# Effective TME-related signature to predict prognosis of patients with head and neck squamous cell carcinoma

**DOI:** 10.3389/fmolb.2023.1232875

**Published:** 2023-08-21

**Authors:** Lingfei Wan, Yuanshuai Li, Wenting Pan, Yuting Yong, Chao Yang, Chen Li, Xingxing Zhao, Ruihong Li, Wen Yue, Xinlong Yan

**Affiliations:** ^1^ College of Life Science and Bioengineering, Faculty of Environmental and Life Sciences, Beijing University of Technology, Beijing, China; ^2^ Stem Cell and Regenerative Medicine Lab, Beijing Institute of Radiation Medicine, Beijing, China; ^3^ Department of Nucleus Radiation-Related Injury Treatment, PLA Rocket Force Characteristic Medical Center, Beijing, China; ^4^ South China Research Center for Stem Cell and Regenerative Medicine, Guangzhou, China

**Keywords:** head and neck squamous cell carcinoma, tumor microenvironment, immune infiltration, tumor mutation burden, prognosis, nomogram

## Abstract

**Introduction:** The tumor microenvironment (TME) is crucial for the development of head and neck squamous cell carcinoma (HNSCC). However, the correlation of the characteristics of the TME and the prognosis of patients with HNSCC remains less known.

**Methods:** In this study, we calculated the immune and stromal cell scores using the “estimate” R package. Kaplan-Meier survival and CIBERSORT algorithm analyses were applied in this study.

**Results:** We identified seven new markers: FCGR3B, IGHV3-64, AC023449.2, IGKV1D-8, FCGR2A, WDFY4, and HBQ1. Subsequently, a risk model was constructed and all HNSCC samples were grouped into low- and high-risk groups. The results of both the Kaplan-Meier survival and receiver operating characteristic curve (ROC) analyses showed that the prognosis indicated by the model was accurate (0.758, 0.756, and 0.666 for 1-, 3- and 5-year survival rates). In addition, we applied the CIBERSORT algorithm to reveal the significant differences in the infiltration levels of immune cells between the two risk groups.

**Discussion:** Our study elucidated the roles of the TME and identified new prognostic biomarkers for patients with HNSCC.

## 1 Introduction

With approximately 600,000 new cases diagnosed annually, squamous cell carcinoma of the head and neck (HNSCC) is the sixth most common malignancy worldwide. More than 50% of HNSCC cases develop to an advanced stage with a 5-year overall survival (OS) rate of approximately 50% (Miyauchi et al., 2019; Yi et al., 2020; Siegel et al., 2021).

Immunotherapy has revolutionized the treatment of cancer, and the clinical application of immune checkpoint inhibitors (ICIs) has provided benefits to patients with various malignant tumors. A known characteristic of HNSCC is severe immunosuppression (Romano and Romero, 2015); therefore, therapy with ICIs play an important role in the treatment of HNSCC patients (Chen et al., 2021). Although many studies have suggested that patients with recurrent and metastatic HNSCC may benefit from ICI immunotherapy, most have shown limited success in the clinical setting, with a 13%–18% overall response rate (Solomon et al., 2018; von Witzleben et al., 2020). The role of immune infiltration in the TME is important for tumorigenesis and tumor progression, both of which affect the clinical prognosis of patients with tumors (Ferris, 2015; Gavrielatou et al., 2020). Furthermore, there is increasing evidence that the tumor mutation burden (TMB) is associated with immunotherapy response (Liu et al., 2019).

Here, we comprehensively analyzed the relationship between the TME, prognosis, TMB, and ICIs in patients with HNSCC. We then established a risk model based on the TME to improve prognostic risk stratification, facilitating better treatment and decision–making for patients. Differentially expressed genes (DEGs) identified here could facilitate a more in-depth understanding of tumor progression and immunotherapy treatment. In addition, this study may help elucidate the mechanism of tumor escape and establish a framework for the development of new prognostic markers.

## 2 Materials and methods

### 2.1 Data download and processing

From The Cancer Genome Atlas (TCGA) database (https://portal.gdc.cancer.gov/), we downloaded the mRNA expression, clinical information, and somatic mutation data of HNSCC samples. After obtaining the somatic mutation data, we used Perl scripts based on the JAVA 8 platform to determine the mutation frequency with number of variants/the length of exons (38 million). Meanwhile, the tumor mutation burden (TMB) value for each sample was calculated.

### 2.2 TME analysis

Using the “estimate” R package, we estimated the infiltration levels of immune and stromal cells, in the form of two scores, immune score and stromal score (Yoshihara et al., 2013). Meanwhile, the sum of immune and stromal score was reflected by the ESTIMATE score. We then explored the correlation between the expression levels of model genes and these scores by performing the Spearman’s rank correlation coefficient test. Additionally, we employed the CIBERSORT algorithm to assess the 22 types of infiltrating immune cells of each sample (Newman et al., 2015).

### 2.3 Identification of differentially expressed genes (DEGs) based on the stromal and immune scores

According to the median stromal and immune scores, we divided 502 HNSCC patients into high- and low-score groups. To identify DEGs between the two score groups, we applied the “limma” R package, with a false-discovery rate (FDR) ≤ 0.05 and |log2 fold change (FC)| ≥ 1.

### 2.4 Construction and validation of the prognostic prediction model in HNSCC

By taking the intersection of the DEGs from the both score groups, the univariate Cox analysis was conducted to primarily screen out immune- and stromal-related genes with prognostic value, using the “survival” R package. A least absolute shrinkage and selection operator (LASSO) analysis was further applied to narrow these prognostic genes. Finally, a multivariate Cox regression model was utilized to select candidate genes related to survival and to construct the prediction model. The risk score was then calculated as follows: risk score = (0.2086 × expression level of FCGR3B) + (−0.0550 × expression level of IGHV3-64) + (−1.8215 × expression level of AC023449.2) + (0.0075 × expression level of IGKV1D-8) + (0.0582 × expression level of FCGR2A) + (−0.5416 × expression level of WDFY4) + (0.0914 × expression level of HBQ1).

Based on the median risk score, we classified all HNSCC patients into low- and high-risk groups. The Kaplan-Meier (KM) survival analysis and the receiver operating characteristic (ROC) curve analyses were used to analyze the OS between the two risk groups and assess the sensitivity and specificity of the signature using the “survivalROC” and “timeROC” R packages.

### 2.5 Functional enrichment analysis

We carried out the Gene Ontology (GO) and the Kyoto Encyclopedia of Genes and Genomes (KEGG) pathway enrichment analysis for the DEGs between the two risk groups, using the “clusterProfiler,” “enrichplot,” and “org.Hs.eg.db” R packages. Furthermore, we used the “GSVA” R package to perform a gene set variation analysis (GSVA) with the purpose of estimating the variation of pathway between the low- and high-risk groups, based on the “c2.cp.kegg.v7.4.symbols.gmt” database, which was downloaded from the Molecular Signatures Database (v7.4, http://www.gsea-msigdb.org/gsea/msigdb/index.jsp) (Hanzelmann et al., 2013).

### 2.6 TMB calculation and visualization

The somatic mutation data were obtained from the TCGA database. The TMB was defined as the total number of somatic gene coding errors, base substitutions, insertions, or deletions detected per megabyte bases of tumor tissue. The value of it for each patient was defined as the total mutation frequency/the length of the human exon (38 Mb) (Lv et al., 2020; Jiang et al., 2021). When calculating TMB, we excluded all synonymous mutations. At the same time, we further studied the mutation status under different risk groups.

### 2.7 Construction of the protein-protein interaction (PPI) network and the competitive endogenous RNA (ceRNA) network

We performed differential analysis for patients between high- and low-risk groups and used the differential genes to construct the PPI network by using the Search Tool for the Retrieval of Interacting Genes (STRING) database.

Construct protein-protein interaction (PPI) network. In addition, we used model genes in the Starbase database (http://starbase.sysu.edu.cn/). The ceRNA regulatory network of model genes was screened and constructed in the database. When predicting the miRNA binding to the model gene through this database, we first ensured that there should be a negative correlation between the expression of miRNA and mRNA. At the same time, miRNA was differentially expressed in normal and tumor. In addition, using the median value of candidate miRNAs, we divided patients into high and low expression groups, and screened miRNAs with survival differences between the two groups through km database. Subsequently, we screened lncRNAs through the Starbase database. According to the theory of ceRNA, there was a positive correlation between lncRNA expression and mRNA. At the same time, the candidate lncRNA should be differentially expressed in normal and tumor tissues, and have survival differences in different expression groups based on the median expression level. According to the theory, the ceRNA networks related to the important model genes was screened and constructed.

### 2.8 Statistical analysis

All statistical analyses were accomplished using the R software (v4.1.1). We followed the methods of Ai-Min Jiang, Yue Zhao, and Ke-Wei Bi et al. (Bi et al., 2020; Jiang et al., 2021; Zhao et al., 2021). To compare the expression level of model genes between the tumor and normal samples, we conducted the Wilcoxon test. To explore the correlation between model gene expression levels and the OS of patients, we used the log-rank test and KM curve analysis. Meanwhile, we performed the univariate and multivariate Cox regression analyses to explore the independent prognostic value of the risk mode. *p*-value ≤0.05 was regarded as significant.

## 3 Results

### 3.1 Acquisition of DEGs based on immune and stromal scores

**FIGURE 1:**
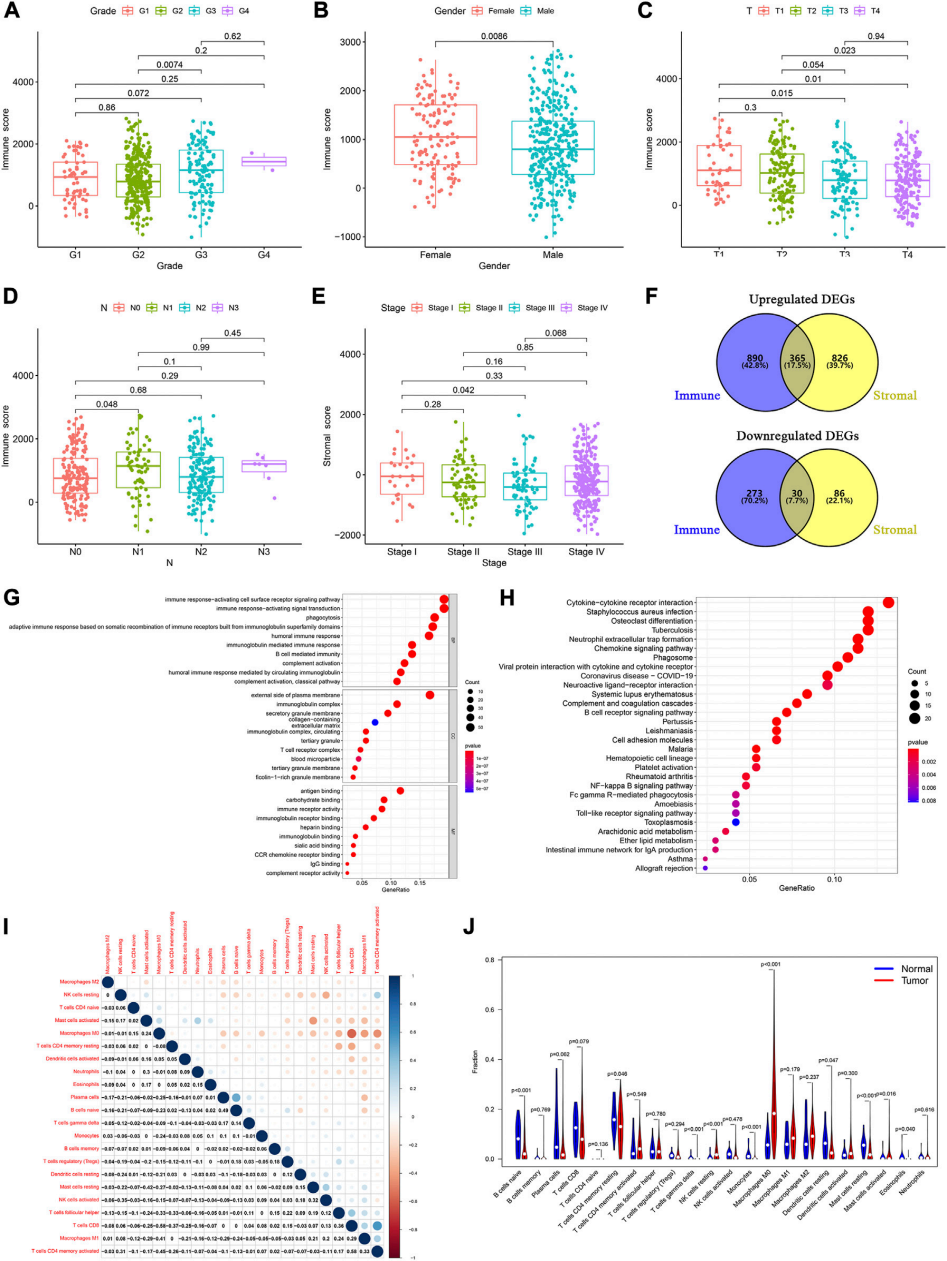
Determination of TME-related DEGs and functional analysis. Distribution of immune and stromal score by clinical characteristics, including **(A)** tumor grades, **(B)** sex, **(C)** T classification, **(D)** N classification, and **(E)** tumor stages. **(F)** Common upregulated and downregulated genes based on immune and stromal scores. **(G)** GO and **(H)** KEGG analyses of 395 common DEGs. **(I)** Correlation between the proportions of 22 types of immune cells in the TME of HNSCC patients. **(J)** Violin plots were used to display the differential infiltration of 22 types of immune cells between tumor and normal samples.

To elucidate the relationship between the immune and stromal scores and clinical features of HNSCC, we used the Wilcoxon test to analyze the differences among patients with different statuses. We found significant differences in immune scores according to tumor grade (Figure 1A), sex (Figure 1B), and T and N stage (Figures 1C, D). Furthermore, stromal scores were significantly different between tumor stage I and III (Figure 1E). These results showed that the immune- and stromal-related activities were associated with HNSCC progression.

Based on the median immune score, we identified 1,558 DEGs, including 1,255 upregulated and 303 downregulated genes (Supplementary Table S1). There were 1,307 DEGs, including 1,191 upregulated and 116 downregulated genes, based on the stromal score (Supplementary Table S2). At the intersection of these two sets of DEGs, 365 upregulated and 30 downregulated genes were identified (Figure 1F; Supplementary Table S3).

We then performed a gene ontology (GO) enrichment analysis on the 395 genes that may be the key factors in the TME. We found that these genes were predominantly associated with the immune responses, such as phagocytosis, activating cell surface receptor signaling pathways, and B cell-mediated immunity (Figure 1G). The Kyoto Encyclopedia of Genes and Genomes (KEGG) enrichment analysis gave similar results, with responses such as phagosome, NF-kappa B signaling pathway, and B cell receptor signaling pathway (Figure 1H). These results indicated that immune-related activities were important characteristics in the TME of HNSCC.

Furthermore, to identify the proportion of the 22 kinds of immune cells in the TME of patients with HNSCC, we conducted a CIBERSORT analysis, using the “CIBERSORT” R package. Using the Pearson analysis, we found that M0 macrophages negatively correlated with CD8^+^ T cells. However, CD8^+^ T cells positively correlated with activated memory CD4^+^ T cells (Figure 1I). These results indicated that there were significant differences between the normal and tumor groups. The normal samples had a higher proportion of native B, resting memory CD4^+^ T, resting mast, and resting dendritic cells than the tumor samples. Moreover, in the tumor patient group, the proportion of resting NK cells and M0 macrophages was higher than that in normal group (Figure 1J).

### 3.2 Establishment of the prognostic prediction model with TCGA cohort

**FIGURE 2:**
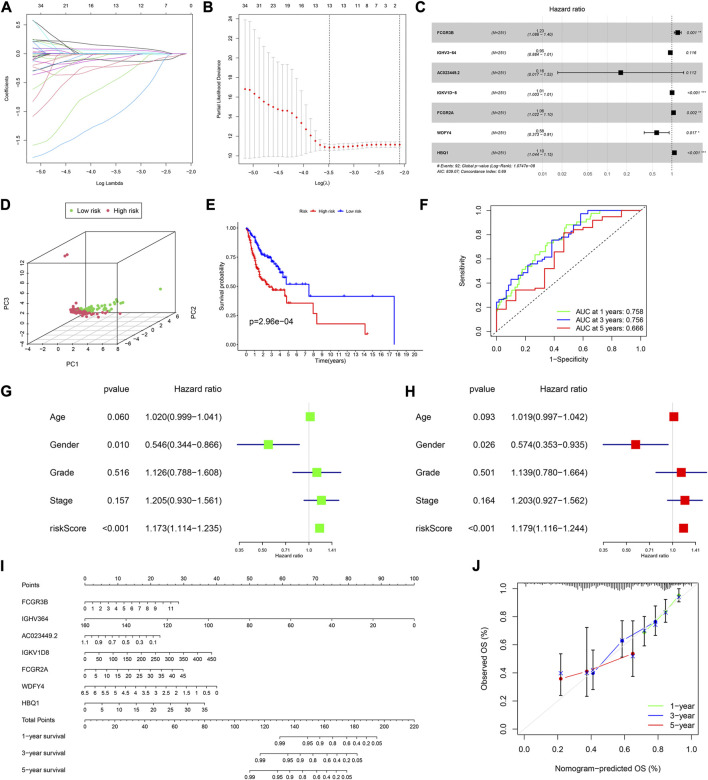
Construction of a prognostic model in the training cohort. **(A,B)** LASSO regression algorithm. **(C)** A prognostic model was constructed by the multivariate Cox regression analysis. **(D)** Principal component analysis. **(E)** Kaplan–Meier (KM) curves of OS for patients in the high- and low-risk groups, respectively. **(F)** Time-dependent ROC curve analysis of the prognostic model. **(G)** Univariate and **(H)** multivariate cox regression analyses to evaluate the prognostic signature. **(I)** Nomogram predicting the survival of HNSCC patients. **(J)** Calibration plot based on the 1-, 3-, and 5-year OS rates of the nomogram. *p < 0.05, **p <0.01, ***p <0.001, and ****p <0.0001.

The univariate Cox analysis of the 395 DEGs identified 50 genes significantly related to OS (Supplementary Table S4). We then used the LASSO regression analysis to screen these genes, and 13 genes were finally identified (Figures 2A, B). All HNSCC samples were then randomly divided into training and validation cohorts, at a ratio of 1:1. The 13 genes were further screened using the multivariate Cox regression analysis. Finally, a set of seven genes, FCGR3B, IGHV3-64, AC023449.2, IGKV1D-8, FCGR2A, WDFY4, and HBQ1, was selected to construct the prognostic model and calculate the risk score (Figure 2C).

Based on the median risk score, 126 and 125 patients were in the low- and high-risk groups, respectively. The results of the principal component analysis (PCA) indicated that patients at different risks were clearly separated into two groups (Figure 2D). Moreover, patients in the high-risk group had a higher death rate and shorter survival time than those in the low-risk group (Supplementary Figure S1A). Based on the KM analysis, we found that patients in the low-risk group had a significantly better OS than those in the high-risk group (*p* < 0.001; Figure 2E). The model had a good predictability for OS, with the area under the curve (AUC) being 0.758, 0.756, and 0.666 for 1, 3, and 5-year OS rates through the time-dependent ROC analysis, respectively (Figure 2F).

To test whether the risk score was independent of other clinical features, such as age, sex, tumor stage, and tumor grade, we performed univariate and multivariable Cox regression analyses. The results showed that it was independent (Figures 2G, H).

Furthermore, we established a nomogram to predict the 1-, 3-, and 5-year survival rates in patients with HNSCC, according to the expression levels of the model genes (Figure 2I). Using the calibration curve, we found that the nomogram had a good predictive value compared to the ideal model (Figure 2J). In the training cohort, the concordance index (C-index) was 0.687.

### 3.3 Validation of the prognostic model

**FIGURE 3:**
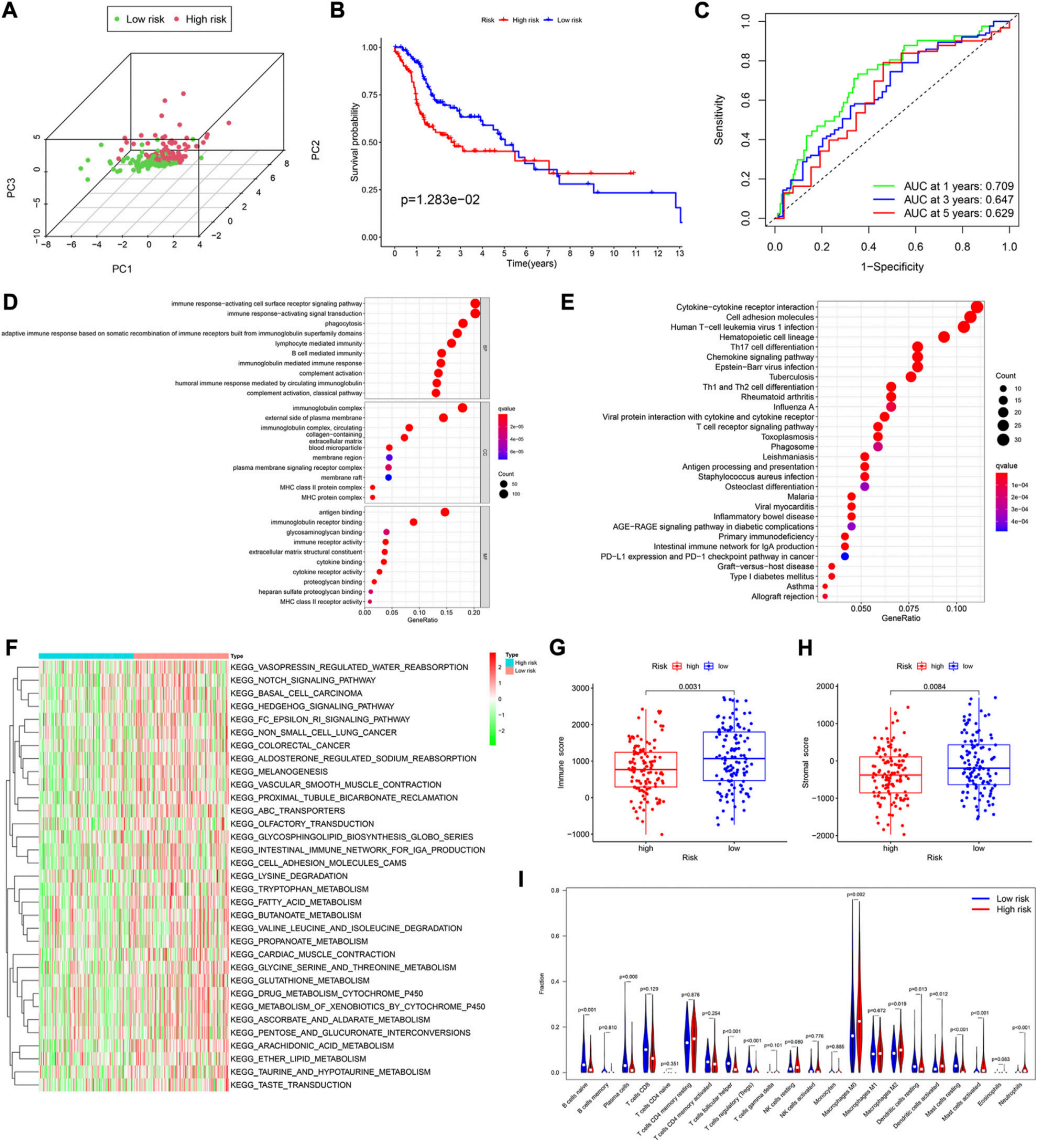
Assessment of the prognostic model in the validation cohort. **(A)** Plot of principal component analysis. **(B)** KM curve of OS for patients in the high- and low-risk groups. **(C)** Time-dependent ROC curve analysis of the prognostic model. **(D,E)** GO and KEGG analyses of DEGs between the high- and low-risk groups in the training cohort. **(F)** GSVA analyses to estimate the variation of pathway between the low- and high-risk groups in the training cohort. **(G,H)** Distribution of immune and stromal scores between the high- and low-risk groups in the training cohort. **(I)** Comparison of infiltration levels of 22 immune cells between the high- and low-risk groups in the training cohort.

According to the median risk score, there were 133 and 115 HNSCC patients in the low and high-risk groups, respectively. The PCA showed a good separation between the risk groups (Figure 3A). The analysis of survival time and patient status in both risk groups showed consistent results (Supplementary Figure S1B). Furthermore, the *p*-value of the KM analysis was 0.01283 (Figure 3B), and the AUC values were 0.709, 0.647, and 0.629 for 1-,3-,5- y survival rates in HNSCC patients, respectively (Figure 3C).

### 3.4 Gene set variation analysis and functional analysis based on the risk model

Using the “limma” R package, we performed a differential analysis of the two risk groups, using the following criteria: FDR ≤0.05 and |log2FC | ≥ 1. In the training and validation cohorts, we identified 750 (Supplementary Table S5), and 755 DEGs (Supplementary Table S7), respectively. Based on these DEGs, GO enrichment and KEGG pathway analyses were performed. We found that in both cohorts, the DEGs were mainly associated with immune-related activities, such as humoral immune response, immune response-activating signal transduction, and immune response-activating cell surface receptor signaling pathway (Figures 3D, E; Supplementary Figures S1C–S1F).

Subsequently, the Gene set variation analysis (GSVA) was used to explore the different biological activities between the two risk groups, with FDR ≤0.05 as the criterion. The results showed that pathways related to metabolism, such as fatty acid metabolism, glycine, serine and threonine metabolism, and ascorbate and aldarate metabolism, were significantly enriched (Figure 3F; Supplementary Figure S2C).

### 3.5 Analysis of immune cell infiltration between the two risk groups

We found that in both cohorts, patients in the low-risk group had a stronger immune response (Figure 3G; Supplementary Figure S2B) and higher stromal scores (Figure 3H; Supplementary Figure S2C) than those in the high-risk groups, using the Wilcoxon signed-rank test. To explore the differences in immune cells, we used the deconvolution algorithm CIBERSORT. The results indicated that in the training cohort, native B (*p* < 0.001), plasma (*p* = 0.006), follicular helper T (*p* < 0.001), regulatory T (*p* < 0.001), resting mast (*p* < 0.001), and resting dendritic cells (*p* < 0.001) were significantly more abundant in the low-risk group than those in the high-risk group, whereas M0 macrophages (*p* = 0.002), M2 macrophages (*p* = 0.019), activated mast cells (*p* < 0.001), neutrophils (*p* < 0.001), and activated dendritic cells (*p* = 0.012) were less abundant (Figure 3I). In the validation cohort, similar results about the immune status were obtained (Supplementary Figure S2D).

**FIGURE 4:**
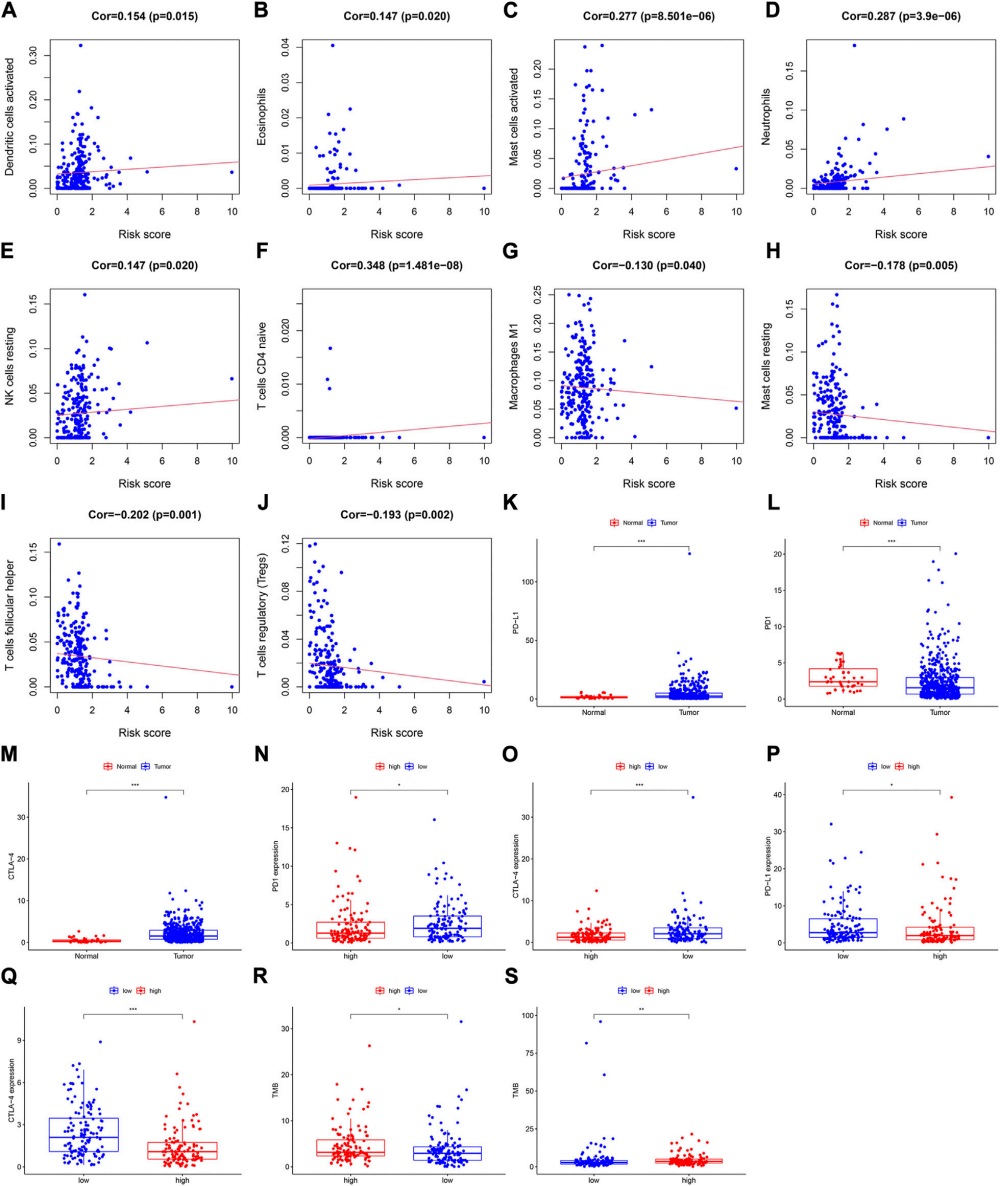
Assessment of the relationships between the risk score and infiltration levels of 22 immune cell types in the training cohort. **(A)** Activated dendritic cells, **(B)** eosinophils, **(C)** activated mast cells, **(D)** neutrophils, **(E)** resting NK cells, **(F)** naive CD4^+^ T cells, **(G)** M1 macrophages, **(H)** resting mast cells, **(I)** follicular helper T cells, and **(J)** regulatory T cells. Differential expression of **(K)** PD-L1, **(L)** PD1, and **(M)** CTLA-4 between the tumor and normal samples. Expression levels of **(N)** PD1, and **(O)** CTLA-4 between the high- and low-risk groups in the training cohort. The differential expression of **(P)** PD-L1, and **(Q)** CTLA-4 between the high- and low-risk groups in the validation cohort. The TMB value in both risk groups in the **(R)** training cohort, and **(S)** validation cohort.

A Pearson analysis was used to analyze the relationship between the risk score and infiltration levels of the 22 immune cell types. In the training cohort, the risk scores had a significantly positive correlation with activated dendritic cells (Figure 4A), eosinophils (Figure 4B), activated mast (Figure 4C), neutrophils (Figure 4D), resting NK (Figure 4E), and naive CD4^+^ T cells (Figure 4F). However, the risk score was negatively correlated with M1 macrophages (Figure 4G), resting mast (Figure 4H), follicular helper T (Figure 4I), and regulatory T cells (Figure 4J).

In the validation cohort, the risk score was positively correlated with eosinophils (Supplementary Figure S2E), activated mast cells (Supplementary Figure S2F), neutrophils (Supplementary Figure S2G), and resting NK cells (Supplementary Figure S2H), whereas it was negatively correlated with naïve B (Supplementary Figure S2I), resting mast (Supplementary Figure S2J), CD8^+^ T (Supplementary Figure S2K), and regulatory T cells (Supplementary Figure S2L).

### 3.6 Association of immune checkpoint molecules with the prognosis prediction model

To explore the relationship between the immune checkpoint molecules and the prognostic model, we evaluated the differential expression of checkpoint molecules in the two risk groups. Compared with the normal tissues, PD-L1 and CTLA-4 expression levels were upregulated in HNSCC tissues (*p* < 0.001; Figures 4K, M), whereas PD1 expression levels were downregulated (*p* < 0.001); (Figure 4L). In both cohorts, the expression level of CTLA-4 in the low-risk group was significantly higher than that in the high-risk group (Figures 4O, Q). In the training cohort, the expression levels of PD1 in the low-risk group were significantly higher than those in the high-risk group (Figure 4N), whereas PD-L1 was more highly expressed in the low-risk group than the high-risk group in the validation cohort (Figure 4P).

These results indicated that the expression levels of immune checkpoint molecules were higher in the low-risk group than those in the high-risk group. Therefore, the prognostic model may provide effective predictive biomarkers, which will enable the optimization of immune checkpoint therapies.

### 3.7 Mutation analysis and visualization

In the different risk groups of both cohorts, we found that there was a difference in TMB. Namely, in both cohorts, the TMB of the high-risk group was higher than that of the low-risk group (Figures 4R, S).

**FIGURE 5:**
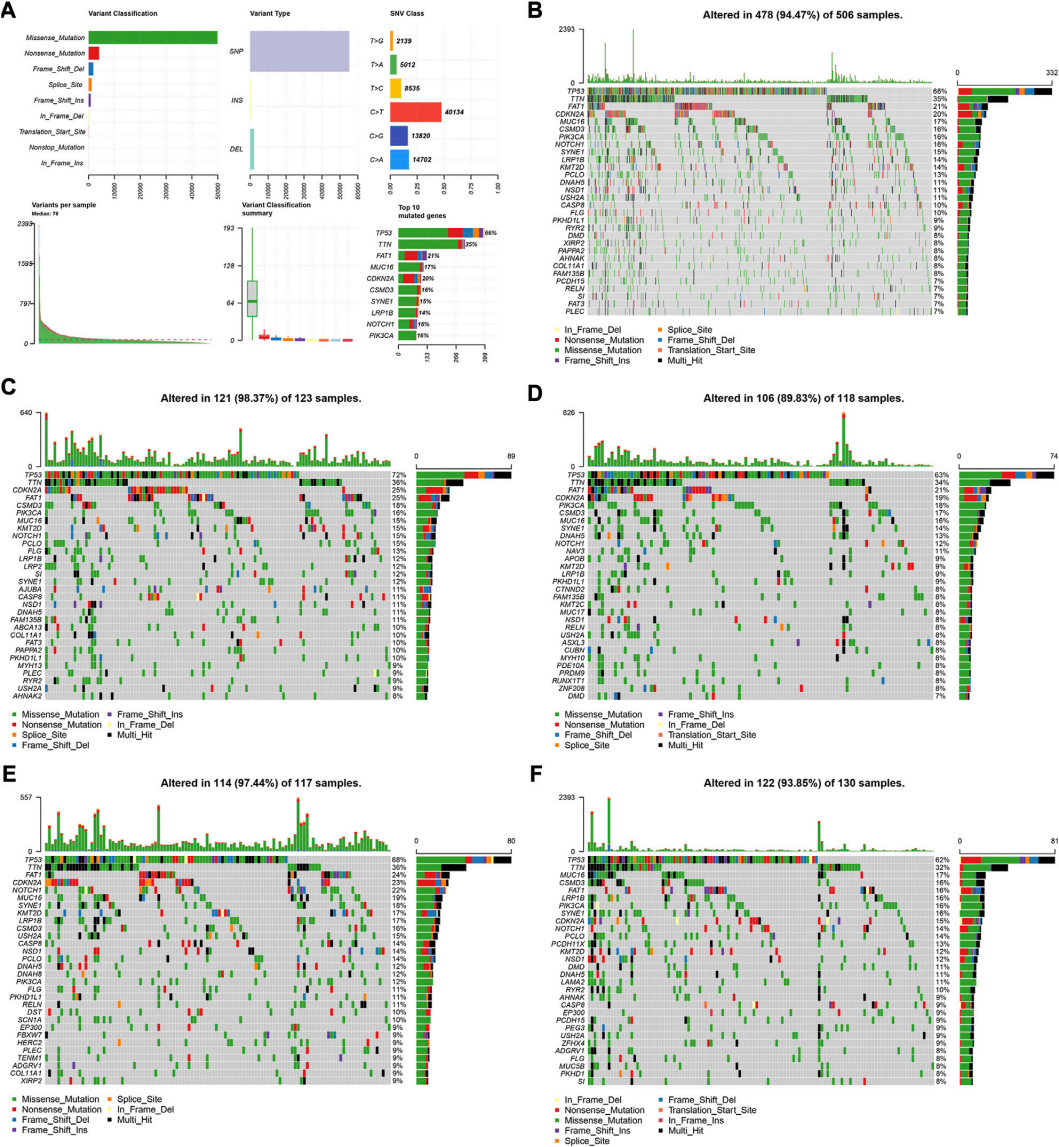
Visualization of mutation profiles. **(A)** Classification of mutation types, including variant classifications, variant types and SNV classes. Waterfall plots displayed the top 30 mutated genes in **(B)** all the TCGA cohorts, **(C)** the high-risk group, and **(D)** low-risk groups of the training cohort, **(E)** and the high-risk group, and **(F)** low-risk groups of the validation cohort.

We utilized the “maftools” R package to analyze and visualize the somatic mutation profiles of 478 HNSCC patients. The detailed mutation information of each sample was illustrated via a waterfall plot, and different mutation types were distinguished by various color annotations. We found that missense mutations, single-nucleotide polymorphism (SNP), and C > T mutations comprised the vast majority of the classification categories. Additionally, the median value of mutations in the samples was 78, and the maximum mutations was 2,393 (Figure 5A). We then presented the number of variant classifications in different samples using box plots. The top 10 mutated genes were TP53 (66%), TTN (35%), FAT1 (21%), CDKN2A (20%), MUC16 (17%), CSMD3 (16%), NOTCH1 (16%), PIK3CA (16%), SYNE1 (15%), and LRP1B (14%) (Figure 5B).

We also investigated the somatic mutation status of different risk groups in the two cohorts. The results showed that the top 10 mutated genes in the four groups differed. In the high-risk group of the training cohort, the top 10 mutated genes were TP53 (72%), TTN (36%), FAT1 (25%), CDKN2A (25%), CSMD3 (18%), PIK3CA (16%), MUC16 (15%), KMT2D (15%), NOTCH1 (15%), and PCLO (15%) (Figure 5C). In the low-risk group, they were TP53 (63%), TTN (34%), FAT1 (21%), CDKN2A (19%), PIK3CA (18%), CSMD3 (17%), MUC16 (16%), SYNE1 (14%), DNAH5 (13%), and NOTCH1 (12%) (Figure 5D). In the high-risk group of the validation cohort, 114 patients possessed somatic mutations, and the top 10 mutated genes were TP53 (68%), TTN (36%), FAT1 (24%), CDKN2A (23%), NOTCH1 (22%), MUC16 (19%), SYNE1 (18%), KMT2D (17%), LRP1B (17%), and CSMD3 (16%) (Figure 5E). In the low-risk group, they were TP53 (62%), TTN (32%), MUC16 (17%), FAT1 (16%), PIK3CA (16%), CSMD3 (16%), LRP1B (16%), SYNE1 (16%), CDKN2A (15%), and NOTCH1 (14%) (Figure 5F).

### 3.8 Acquisition of core genes and establishment of the competitive endogenous RNA (ceRNA) network

**FIGURE 6:**
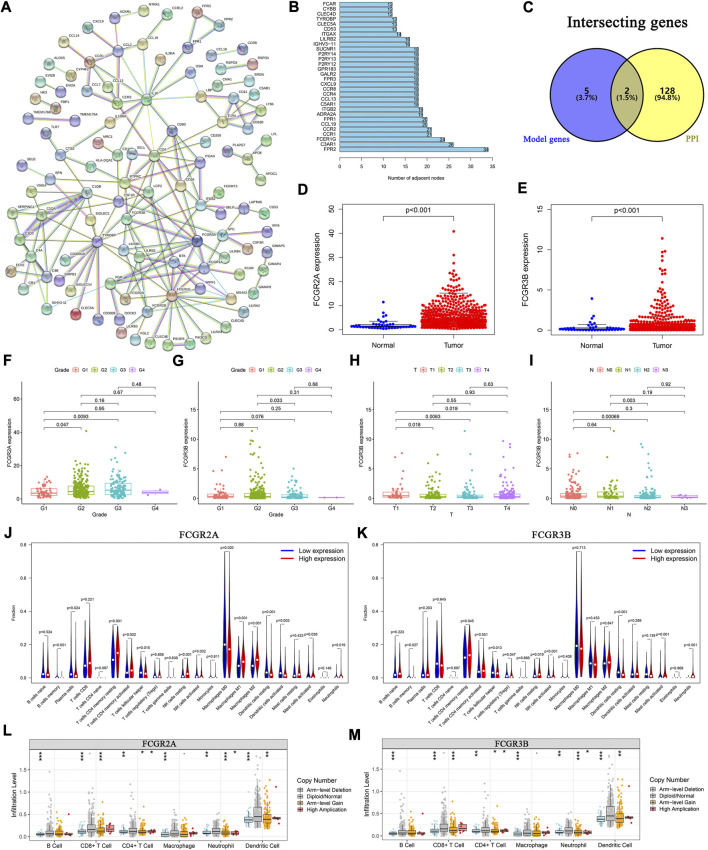
Protein-protein interaction network and the landscape of core genes. **(A)** Interaction network of 395 DEGs. **(B)** The top 30 core elements calculated by the number of degrees. **(C)** Common genes at the intersection of 130 genes and model genes. The differential expression of **(D)** FCGR2A, and **(E)** FCGR3B in tumor and normal samples. Distribution of FCGR2A and FCGR3B in **(F, G)** tumor grades, **(H)** T and **(I)** N classifications. Comparison of infiltration levels of 22 immune cells in the high- and low-expression groups based on the median expression levels of **(J)** FCGR2A, and **(K)** FCGR3B. Analysis of immune cell infiltration levels and somatic copy number alterations in **(L)** FCGR2A, and **(M)** FCGR3B.

To elucidate the biological relationships among the 395 DEGs, we used the Search Tool for the Retrieval of Interacting Genes (STRING) database (https://www.string-db.org/), based on genes with co-expression coefficients higher than 0.9 (Figure 6A). We identified 130 genes with strong mutual correlations, and also identified the top 30 genes according to the number of degrees between the two pairs (Figure 6B).

We identified two common genes, FCGR2A and FCGR3B, which were located at the intersection of the 130 STRING-identified and model genes (Figure 6C). Using the Wilcoxon test, we found that the expression levels of FCGR2A and FCGR3B were significantly higher in HNSCC samples than those in normal samples (Figures 6D, E). Furthermore, using the paired-sample test analysis, we found that the expression of FCGR2A significantly differed between normal and tumor tissue (Supplementary Figure S3A). However, no significant survival differences were observed between the high- and low-expression groups based on the median expression levels of FCGR2A (Supplementary Figure S3B) and FCGR3B (Supplementary Figure S3C).

We then examined the expression of the two genes under different clinical conditions. The results showed that FCGR2A was significantly differentially expressed between tumor grade I and II (Figure 6F). Moreover, the expression level of FCGR3B was significantly different between tumor grades (Figure 6G), T (Figure 6H) and N stages (Figure 6I). In both cohorts, the results indicated that the expression of FCGR3B in the high-risk group was significantly higher than that in the low-risk group (Supplementary Figures S3E, S3F). In the training cohort, the expression of FCGR2A in the high-risk group was higher than that in the low-risk group (Supplementary Figure S3D).

We then used two methods to identify the immune cells associated with FCGR2A and FCGR3B. First, HNSCC samples were divided into low- and high-expression groups based on the median expression of FCGR2A. The Wilcoxon test was used to compare the different infiltration levels of the 22 immune cells in the two groups. The results indicated that the infiltration levels of the resting memory CD4^+^ T cells, activated resting memory CD4^+^ T, resting NK cells, and M1 and M2 macrophages in the high-expression group were higher than those in the low-expression group, whereas plasma, activated dendritic, resting dendritic, follicular helper T cells, and M0 macrophages showed the opposite trend (Figure 6J). Subsequently, using Spearman’s rank correlation analysis, we found that the infiltration levels of plasma, naive CD4^+^ T, activated memory CD4^+^ T, follicular helper T cells, resting dendritic, activated dendritic, activated mast cells, M0 and M1 macrophages, eosinophils, and neutrophils were closely correlated with the expression of FCGR2A. Considering the intersection of the immune cells from the two sets of results, memory B, plasma, resting memory and activated memory CD4^+^ T, follicular helper T, resting dendritic, activated dendritic, activated mast, resting NK, activated NK cells, M0, M1, M2 macrophages, and neutrophils were correlated with the expression of FCGR2A (Supplementary Figure S3G). The immune cells that closely associated with FCGR3B were memory B, resting memory CD4^+^ T, follicular helper T, resting NK, activated NK, resting dendritic, activated mast cells, and neutrophils (Figure 6K; Supplementary Figure S3H).

**FIGURE 7:**
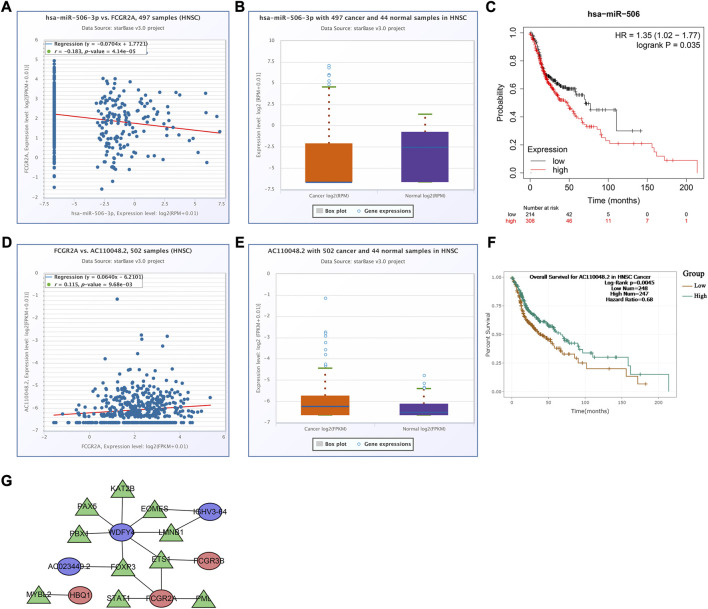
Establishment of the competitive endogenous RNA network constructed using the starBase database. Correlation of expression of **(A)** hsa-miR-506-3p with FCGR2A, and **(D)** AC110048.2 with FCGR2A. The expression levels of **(B)** hsa-miR-506-3p, and **(E)** AC110048.2 in HNSCC and normal samples. KM curves of the prognostic value of **(C)** hsa-miR-506 in the KM plot database, and **(F)** AC110048.2 in the StarBase database. **(G)** The transcription factor regulatory networks associated with the model genes. Red ellipse indicates upregulated model genes; blue ellipse indicates downregulated model genes; green triangle indicates transcription factors.

To analyze the effects of somatic cell copy number alternations (CNAs) of these two genes on infiltration of immune cells, such as B, CD4^+^ T, CD8^+^ T, dendritic cells, neutrophils, and macrophages, we applied the Tumor Immune Estimation Resource (TIMER, https://cistrome.shinyapps.io/timer/). The results showed that the six immune cells were significantly affected by the arm-level deletion and gain of the two genes in HNSCC (Figures 6L, M). It has been widely acknowledged that miRNAs are short noncoding RNAs that can induce mRNA silencing and instability by binding to specific target sites. We predicted that the upstream miRNAs might bind to FCGR2A. These upstream miRNAs, including hsa-miR-124-3p, hsa-miR-145-5p, hsa-miR-299-3p, hsa-miR-513a-5p, hsa-miR-506-3p, and hsa-miR-671-5p, were found through the ENCORI (https://starbase.sysu.edu.cn/) database, which predicted target genes using PITA, RNA22, miRmap, DIANA-microT, miRanda, PicTar, and TargetScan programs. We performed the following analysis only for the predicted miRNAs that appeared in more than two programs. Based on the ceRNA hypothesis, hsa-miR-506-3p was finally chosen (Figures 7A–C). Next, we predicted the upstream lncRNAs. The results showed that there were 33 possible lncRNAs upstream of hsa-miR-506-3p. LncRNAs can competitively bind to shared miRNAs to increase mRNA expression. Therefore, there should be a negative correlation between lncRNAs and miRNAs or a positive correlation between lncRNAs and mRNAs. Based on expression, survival and correlation analysis, we found that AC110048.2 may potentially be the upstream lncRNA of the miR-506-3p/FCGR2A axis in HNSCC (Figures 7D–F).

Finally, we established a transcription factor regulatory network for the model genes, using the Cistrome website (http://cistrome.org/). From this database, 314 transcription factors were identified. There were 59 differentially expressed transcription factors between normal and tumor samples under the criteria FDR <0.05 and |log_2_FC | ≥ 1 (Supplementary Table S7). According to the criteria of |correlation coefficient| > 0.3 and *p*-value <0.001 using Pearson’s correlation analyses, we identified 10 transcription factors associated with the model genes, and constructed the transcription factor regulatory network (Figure 7G; Supplementary Table S8).

## 4 Discussion

Cancer immunotherapy, which differently regulates the immune system, has been widely used in the field of oncology (Baxevanis et al., 2009; Yang, 2015). The TME is closely connected with immunotherapy and plays an important role in tumor genesis and development (Quail and Joyce, 2013). Therefore, it is necessary to explore potential therapeutic targets for early diagnosis and therapy. Thus, immune-based prognostic characteristics have become the focus of cancer risk prediction (Belli et al., 2018; Roma-Rodrigues et al., 2019; Shi et al., 2021).

In this study, based on the transcriptome data of HNSCC, we calculated the scores of immune and stromal cells in the TME, and found that they were significantly different in each phase of tumor development, suggesting that TME played a significant role in tumor growth. Based on the median scores, we obtained 395 DEGs related to the TME. The GO and KEGG enrichment analysis showed that these genes were significantly enriched in immune- and metabolism-related pathways, which preliminarily suggested that immune-related genes and pathways had significant association with the occurrence and development of HNSCC.

Based on these DEGs, a prognostic model consisting of FCGR3B, IGHV3-64, AC023449.2, IGKV1D-8, FCGR2A, WDFY4, and HBQ1 was constructed. Moreover, based on the model genes, a clinical prediction nomogram was constructed and verified to have good predictability. Based on the literature, FCGR3B is a gene that encodes FcγRIIIb and plays an important role in the immune system. Therefore, the biological function of FCGR3B in head and neck squamous cell carcinoma (HNSCC) may be related to the immune system. Another study found that copy number variations of FCGR3B were associated with susceptibility to autoimmune diseases, suggesting that FCGR3B may be involved in regulating immune responses (Leemans et al., 2011; Molokhia et al., 2011; Alberici et al., 2020). The IGH family is involved in the development of B-cell malignancies. Somatic hypermutation of IGHV genes is characteristic of many B-cell lymphomas (Sahota et al., 1997; Ghia et al., 2007; Varettoni et al., 2013). One member of the IGH family, IGHV3-64, was found to be involved in the regulation of immune cells, particularly the positive regulation of B cell activation. Currently, some studies have explored the biological functions of IGHV3-64 in other cancers. For example, in chronic lymphocytic leukemia (CLL), the expression level of IGHV3-64 is closely related to clinical prognosis (Crombie and Davids, 2017). In addition, there are studies suggesting that IGHV3-64 may be associated with the development and prognosis of gastrointestinal (Guan et al., 2020). However, the results of these studies are inconsistent, and more research is needed to determine the biological functions of IGHV3-64 in different cancers. Previous studies have shown that WDFY4 is involved in the function of various immune cells, and it can modulate B cells through noncanonical autophagy, and participates in the regulation of systemic lupus erythematosus (Zhao et al., 2012; Yuan et al., 2018). Furthermore, the deficiency of WDFY4 results in a decrease in CD8^+^ T cells (Li et al., 2021). Hemoglobin subunit theta 1 (HBQ1) is often used as an indicator related to tumor metabolism. When patients with breast cancer were treated with the combination of bevacizumab and doxorubicin, HBQ1 was often differentially expressed (Borgan et al., 2013; Bae et al., 2022) and IGKV1D-8 was primarily involved in immune response (Gaudet et al., 2011). However, there have been a few reports on AC023449.2 and IGKV1D-8 (Alberici et al., 2018; Treffers et al., 2018; Dai et al., 2021). FCGR2A is closely associated with immunity and is considered a cell-surface receptor on phagocytic cells. Although FCGR2A has rarely been reported in HNSCC, our study showed that immune cells, such as memory B, plasma, resting memory CD4^+^ T, activated memory CD4^+^ T, follicular helper T, resting and activated dendritic, activated mast, resting and activated NK cells, M0, M1, and M2 macrophages, and neutrophils, were closely associated with FCGR2A expression in HNSCC. As a product of immune cells, FCGR3B plays an important role in the connection and clearance of neutrophils and other immune complexes (Coxon et al., 2001; Fanciulli et al., 2007). We found that immune cells including memory B, resting memory CD4^+^ T, follicular helper T, resting and activated NK, resting dendritic, activated mast cells, and neutrophils, were closely associated with FCGR3B.

Following GO and KEGG analyses, we found that the DEGs were strongly associated with immunity in the two risk groups. The GSVA results indicated that metabolism-related pathways, such as fatty acid, butanoate, glycine, serine, and threonine metabolism, were significantly different between the two risk groups. Changes in cell metabolism affected tumor progression. Fatty acid metabolism plays a crucial role in tumorigenesis and Epithelial–mesenchymal transition (EMT) regulation.

Furthermore, the infiltration of CD8^+^ T cells was higher in the low-risk group than that in the high-risk group. We also found that in both cohorts, the low-risk group had a higher expression of PD1, PD-L1, and CTLA-4. We therefore speculated that the low-risk group may benefit the most from antibody therapies targeting the PD1, PD-L1, and CTLA-4 immune checkpoints. However, in both cohorts, the TMB was higher in the high-risk group than that in the low-risk group.

## 5 Conclusion

In conclusion, our study highlights the importance of the tumor microenvironment (TME) in the development and prognosis of head and neck squamous cell carcinoma (HNSCC). By analyzing gene expression data from the TCGA database, we identified seven new markers that were found to be associated with HNSCC prognosis. We also constructed a risk model based on the TME that accurately predicted patient outcomes. Our study further revealed significant differences in the infiltration levels of immune cells between low- and high-risk groups. These findings provide a better understanding of the mechanisms of tumor progression and immune infiltration in HNSCC and offer potential biomarkers for prognosis and treatment. Our study may also facilitate the development of new therapeutic strategies for HNSCC patients.

## Data Availability

Publicly available datasets were analyzed in this study. This data can be found here: The TCGA data in the study have been downloaded from https://portal.gdc.cancer.gov/.
