# Supplementary material for: Effective TME-related signature to predict prognosis of patients with head and neck squamous cell carcinoma
Source: Front Mol Biosci. 2023 Aug 21;10:1232875. doi: 10.3389/fmolb.2023.1232875 (PMC10475735; doi:10.3389/fmolb.2023.1232875)
Supplement: Supplementary file 1 [file DataSheet1.zip › Supplementary Material/Supplementary Material.docx]

Supplementary Material

# Supplementary Figures and Tables

## Supplementary Figures

**Supplementary Figure S1: Visualization of the prognostic model.**

The distribution of the expression levels of model genes, risk score, and survival status for each patient in the (A) training cohort, and (B) validation cohort. (C-D) GO and (E-F) KEGG analyses for DEGs between high- and low-risk groups in the validation cohort.

**Supplementary Figure S2: The immune landscape of patients in the validation cohort.**

(A) Gene set variation analysis for estimating the variation of pathway between the low- and high-risk groups. Distribution of (B) immune score, and (C) stromal score between the high- and low-risk groups. (D) Comparison of 22 immune cell infiltration levels in the high- and low-risk groups. The relationships between the risk score and infiltration levels of immune cells, including (E) eosinophils, (F) activated mast cells, (G) neutrophils, (H) resting NK cells, (I) naive B cells, (J) resting mast cells, (K) CD8+ T cells, and (L) regulatory T cells.

**Supplementary Figure S3: The differentiated expression of core genes.**

(A) Paired differentiation analysis for the expression level of FCGR2A. Kaplan–Meier survival analysis of patients in the high- and low-expression levels of (B) FCGR2A, and (C) FCGR3B. Differentiated expression levels of FCGR2A, and FCGR3B in the high- and low-risk groups in (D-E) the training cohort and (F) the validation cohort. Venn plot showing the common immune cells shared by the two methods for (G) FCGR2A, and (H) FCGR3B.

## Supplementary Tables

**Supplementary Table S1.** 1,558 DEGs, including 1,255 upregulated and 303 downregulated genes, based on the median immune score in TCGA cohort.

**Supplementary Table S2.** 1,307 DEGs, including 1,191 upregulated and 116 downregulated genes, according to the median stromal score in TCGA cohort.

**Supplementary Table S3.** 365 upregulated and 30 downregulated genes by taking the intersection of the two sets of DEGs.

**Supplementary Table S4.** 13 genes identified by the univariate Cox analysis.

**Supplementary Table S5.** 750 DEGs between low- and high-risk groups in the training cohort.

**Supplementary Table S6.** 755 DEGs between low- and high-risk groups in the validation cohort.

**Supplementary Table S7.** 59 differentially expressed transcription factors between normal and tumor samples with FDR < 0.05 and |log_2_FC | ≥ 1.

**Supplementary Table S8.** 10 transcription factors associated with the model genes.
