# Supplementary material for: Effective TME-related signature to predict prognosis of patients with head and neck squamous cell carcinoma
Source: Front Mol Biosci. 2023 Aug 21;10:1232875. doi: 10.3389/fmolb.2023.1232875 (PMC10475735; doi:10.3389/fmolb.2023.1232875)
Supplement: Supplementary file 1 [file DataSheet1.zip › Supplementary Material/Supplementary Table S1.docx]

Table S1. 1,558 DEGs, including 1,255 upregulated and 303 downregulated genes, based on the median immune score in TCGA cohort.

| gene | logFC | pValue | fdr | regulated |
| --- | --- | --- | --- | --- |
| KRT36 | 4.23490158 | 6.64E-06 | 3.47E-05 | upregulated |
| FXYD2 | 3.923365452 | 2.16E-24 | 5.35E-23 | upregulated |
| SPIB | 3.713630636 | 1.12E-39 | 5.34E-38 | upregulated |
| IL17REL | 3.663790354 | 9.33E-09 | 7.91E-08 | upregulated |
| PLA2G2D | 3.446947653 | 8.43E-37 | 3.59E-35 | upregulated |
| CXCR2P1 | 3.380638017 | 2.61E-55 | 3.81E-53 | upregulated |
| ODAM | 3.278031659 | 0.008426563 | 0.018855032 | upregulated |
| CRB2 | 3.125607856 | 2.04E-06 | 1.17E-05 | upregulated |
| DRGX | 3.099218733 | 0.006117805 | 0.014343518 | upregulated |
| FCER2 | 3.052898374 | 4.47E-16 | 7.68E-15 | upregulated |
| MS4A1 | 3.034317543 | 2.96E-23 | 7.00E-22 | upregulated |
| IGLV2-34 | 2.974667305 | 7.53E-09 | 6.48E-08 | upregulated |
| LINC02446 | 2.971350781 | 1.15E-43 | 6.86E-42 | upregulated |
| CXCL11 | 2.928622904 | 8.07E-32 | 2.67E-30 | upregulated |
| IGKV1D-12 | 2.876266 | 7.42E-12 | 9.28E-11 | upregulated |
| KEL | 2.874646381 | 8.01E-08 | 5.92E-07 | upregulated |
| TRBV13 | 2.845293839 | 1.99E-29 | 5.88E-28 | upregulated |
| IGLV4-3 | 2.84340391 | 2.48E-08 | 1.98E-07 | upregulated |
| TIMD4 | 2.827516265 | 4.67E-39 | 2.16E-37 | upregulated |
| IGHV3-73 | 2.796757987 | 6.08E-15 | 9.71E-14 | upregulated |
| GZMK | 2.777340483 | 8.63E-53 | 9.66E-51 | upregulated |
| AC093063.1 | 2.765006239 | 6.05E-17 | 1.09E-15 | upregulated |
| GZMH | 2.764753169 | 4.08E-49 | 3.51E-47 | upregulated |
| TRBV7-6 | 2.756641123 | 8.32E-44 | 5.03E-42 | upregulated |
| IGHV3-43 | 2.746556912 | 3.01E-13 | 4.29E-12 | upregulated |
| C7 | 2.728865784 | 2.95E-10 | 3.07E-09 | upregulated |
| TRBV27 | 2.722260067 | 4.95E-34 | 1.83E-32 | upregulated |
| CCL19 | 2.680126242 | 7.84E-28 | 2.18E-26 | upregulated |
| MIR8071-1 | 2.667365317 | 3.35E-18 | 6.45E-17 | upregulated |
| IGHV3-22 | 2.665059262 | 1.03E-11 | 1.26E-10 | upregulated |
| TCL1A | 2.66248739 | 3.69E-36 | 1.50E-34 | upregulated |
| NKG7 | 2.654151884 | 3.97E-57 | 6.80E-55 | upregulated |
| CXCR3 | 2.646847919 | 2.79E-62 | 1.05E-59 | upregulated |
| DNASE2B | 2.640191166 | 7.12E-14 | 1.07E-12 | upregulated |
| IGLV2-8 | 2.635148369 | 2.06E-16 | 3.60E-15 | upregulated |
| SCML4 | 2.634307796 | 3.16E-55 | 4.58E-53 | upregulated |
| CXCL10 | 2.625801788 | 1.40E-36 | 5.90E-35 | upregulated |
| LILRA4 | 2.620679753 | 2.91E-47 | 2.21E-45 | upregulated |
| FCRL3 | 2.611950359 | 1.96E-44 | 1.24E-42 | upregulated |
| IFNG | 2.605671917 | 5.64E-41 | 2.85E-39 | upregulated |
| VCAM1 | 2.604443863 | 4.87E-30 | 1.48E-28 | upregulated |
| IGKV1D-33 | 2.599093106 | 1.69E-09 | 1.59E-08 | upregulated |
| CD8A | 2.591772411 | 2.49E-54 | 3.36E-52 | upregulated |
| AC026369.3 | 2.581441333 | 1.03E-27 | 2.84E-26 | upregulated |
| TRAV24 | 2.576697265 | 7.44E-36 | 2.98E-34 | upregulated |
| IGKV1D-39 | 2.575520353 | 2.28E-11 | 2.71E-10 | upregulated |
| IGKV2OR2-1 | 2.572471523 | 1.13E-07 | 8.12E-07 | upregulated |
| TRBV19 | 2.571155657 | 4.02E-52 | 4.38E-50 | upregulated |
| TNFRSF13B | 2.569890945 | 6.18E-29 | 1.80E-27 | upregulated |
| OR2I1P | 2.563121637 | 4.36E-39 | 2.03E-37 | upregulated |
| SIGLEC8 | 2.555712482 | 5.06E-47 | 3.79E-45 | upregulated |
| GPR174 | 2.548165274 | 8.10E-43 | 4.54E-41 | upregulated |
| TRBV7-4 | 2.541268925 | 1.80E-29 | 5.34E-28 | upregulated |
| CHIT1 | 2.540382571 | 2.40E-16 | 4.17E-15 | upregulated |
| ZNF683 | 2.529950278 | 2.92E-42 | 1.59E-40 | upregulated |
| ADAMDEC1 | 2.528734068 | 8.18E-48 | 6.56E-46 | upregulated |
| LGALS17A | 2.527687368 | 4.66E-26 | 1.20E-24 | upregulated |
| IGHD3-3 | 2.523359316 | 5.72E-12 | 7.23E-11 | upregulated |
| AC135068.8 | 2.522893958 | 7.14E-09 | 6.16E-08 | upregulated |
| TRAV4 | 2.520045824 | 1.33E-46 | 9.71E-45 | upregulated |
| TRAV22 | 2.5166001 | 4.77E-42 | 2.57E-40 | upregulated |
| RORB | 2.515449372 | 4.67E-11 | 5.37E-10 | upregulated |
| GPR25 | 2.509293184 | 5.42E-39 | 2.50E-37 | upregulated |
| TRBV2 | 2.508857763 | 4.79E-47 | 3.60E-45 | upregulated |
| TRBV6-5 | 2.507070933 | 1.67E-48 | 1.38E-46 | upregulated |
| TRAV41 | 2.505592607 | 3.04E-36 | 1.25E-34 | upregulated |
| IGKV2-18 | 2.503098926 | 2.75E-13 | 3.93E-12 | upregulated |
| TRBV5-1 | 2.502169741 | 4.73E-52 | 5.04E-50 | upregulated |
| TRBV15 | 2.500115682 | 1.95E-31 | 6.33E-30 | upregulated |
| TRBV10-3 | 2.494747022 | 3.04E-44 | 1.88E-42 | upregulated |
| TRBV6-6 | 2.490448212 | 9.76E-40 | 4.67E-38 | upregulated |
| TRAV20 | 2.490051583 | 9.81E-37 | 4.15E-35 | upregulated |
| ZNF831 | 2.489440189 | 3.86E-51 | 3.84E-49 | upregulated |
| SIRPG | 2.488565206 | 8.80E-64 | 5.10E-61 | upregulated |
| ASCL4 | 2.485233021 | 0.002526173 | 0.006724977 | upregulated |
| IGHV1-3 | 2.476401778 | 3.99E-13 | 5.63E-12 | upregulated |
| AC022126.1 | 2.476187067 | 1.87E-28 | 5.33E-27 | upregulated |
| TRBV4-2 | 2.471464367 | 1.29E-37 | 5.66E-36 | upregulated |
| CXCL9 | 2.449391359 | 8.65E-44 | 5.16E-42 | upregulated |
| TRAV12-3 | 2.446848934 | 2.06E-47 | 1.58E-45 | upregulated |
| SH2D1A | 2.443342672 | 2.98E-57 | 5.25E-55 | upregulated |
| TRBV7-9 | 2.436912752 | 3.39E-46 | 2.39E-44 | upregulated |
| TLR8 | 2.435754225 | 5.03E-58 | 1.00E-55 | upregulated |
| IGLL1 | 2.429742999 | 3.24E-11 | 3.80E-10 | upregulated |
| IDO1 | 2.427819276 | 1.78E-40 | 8.82E-39 | upregulated |
| IGHV1OR16-3 | 2.424965217 | 1.36E-09 | 1.30E-08 | upregulated |
| TRBV14 | 2.423761231 | 1.49E-36 | 6.25E-35 | upregulated |
| SCIMP | 2.422163755 | 6.00E-54 | 7.89E-52 | upregulated |
| TRBV12-4 | 2.418993483 | 8.41E-39 | 3.85E-37 | upregulated |
| TRBV30 | 2.418461565 | 7.63E-32 | 2.52E-30 | upregulated |
| CCR5 | 2.412399558 | 1.19E-66 | 2.35E-63 | upregulated |
| TRBV3-1 | 2.409269843 | 1.73E-43 | 1.03E-41 | upregulated |
| IGKV2D-24 | 2.409132059 | 1.15E-09 | 1.11E-08 | upregulated |
| CD40LG | 2.408590542 | 5.23E-40 | 2.54E-38 | upregulated |
| TRAV13-1 | 2.407154992 | 1.62E-47 | 1.27E-45 | upregulated |
| TRBV5-5 | 2.404729189 | 1.41E-29 | 4.19E-28 | upregulated |
| CRTAM | 2.402386241 | 1.02E-53 | 1.31E-51 | upregulated |
| LAG3 | 2.401519795 | 1.57E-53 | 1.90E-51 | upregulated |
| IGHV3-20 | 2.393485092 | 4.08E-11 | 4.73E-10 | upregulated |
| SLAMF6 | 2.393476577 | 5.16E-56 | 7.88E-54 | upregulated |
| TRAV35 | 2.389911524 | 6.66E-34 | 2.45E-32 | upregulated |
| TRBV6-1 | 2.386071574 | 4.37E-40 | 2.13E-38 | upregulated |
| TRBV29-1 | 2.385576301 | 1.81E-42 | 9.93E-41 | upregulated |
| LINC00861 | 2.384595219 | 1.93E-41 | 9.97E-40 | upregulated |
| TIFAB | 2.383895156 | 3.12E-53 | 3.70E-51 | upregulated |
| RHOXF1 | 2.382784104 | 0.000250656 | 0.000889501 | upregulated |
| FTH1P22 | 2.38064639 | 1.64E-48 | 1.36E-46 | upregulated |
| FASLG | 2.378822271 | 4.07E-47 | 3.08E-45 | upregulated |
| UBD | 2.377493632 | 3.97E-39 | 1.86E-37 | upregulated |
| TRAV12-1 | 2.370367316 | 4.51E-39 | 2.09E-37 | upregulated |
| GPR18 | 2.367672173 | 1.04E-51 | 1.10E-49 | upregulated |
| IGKV2-28 | 2.366753129 | 7.40E-11 | 8.30E-10 | upregulated |
| TRGC2 | 2.365711414 | 1.96E-47 | 1.51E-45 | upregulated |
| IGKV2-24 | 2.362534686 | 1.32E-16 | 2.34E-15 | upregulated |
| TRAV21 | 2.358111512 | 1.94E-42 | 1.06E-40 | upregulated |
| TRBV18 | 2.348213064 | 4.66E-43 | 2.70E-41 | upregulated |
| IGLV3-16 | 2.347376132 | 1.57E-13 | 2.28E-12 | upregulated |
| IGLV3-6 | 2.343052782 | 5.22E-10 | 5.27E-09 | upregulated |
| TRAV38-1 | 2.342697892 | 7.95E-30 | 2.40E-28 | upregulated |
| TRBC2 | 2.340905755 | 2.71E-62 | 1.05E-59 | upregulated |
| KIF25 | 2.339292451 | 3.78E-11 | 4.39E-10 | upregulated |
| LINC01781 | 2.338624831 | 1.16E-17 | 2.16E-16 | upregulated |
| IGKV1D-17 | 2.33802688 | 1.73E-09 | 1.63E-08 | upregulated |
| CD19 | 2.337838022 | 2.74E-17 | 5.03E-16 | upregulated |
| TRBV28 | 2.336200274 | 2.82E-62 | 1.05E-59 | upregulated |
| TRBV4-1 | 2.336006198 | 9.98E-34 | 3.63E-32 | upregulated |
| CCL25 | 2.335513379 | 3.13E-14 | 4.82E-13 | upregulated |
| PDCD1 | 2.334226488 | 4.60E-59 | 1.04E-56 | upregulated |
| TRGV10 | 2.332230508 | 2.03E-41 | 1.05E-39 | upregulated |
| COL19A1 | 2.331360242 | 2.01E-07 | 1.39E-06 | upregulated |
| TRAV23DV6 | 2.329460166 | 2.79E-36 | 1.15E-34 | upregulated |
| TRBC1 | 2.328167094 | 2.32E-51 | 2.35E-49 | upregulated |
| IGLV6-57 | 2.32702284 | 8.80E-13 | 1.20E-11 | upregulated |
| TRAV26-2 | 2.325505656 | 1.10E-32 | 3.80E-31 | upregulated |
| MIR8071-2 | 2.325406201 | 5.02E-18 | 9.60E-17 | upregulated |
| CD48 | 2.325134285 | 1.73E-67 | 4.26E-64 | upregulated |
| KCNA2 | 2.323660671 | 4.00E-16 | 6.89E-15 | upregulated |
| FCRL6 | 2.322719606 | 3.66E-48 | 2.98E-46 | upregulated |
| CD8B | 2.322164613 | 4.55E-52 | 4.87E-50 | upregulated |
| CD2 | 2.321154351 | 1.07E-65 | 1.25E-62 | upregulated |
| GBP5 | 2.316784207 | 4.51E-38 | 2.01E-36 | upregulated |
| LILRB4 | 2.316378406 | 1.10E-56 | 1.78E-54 | upregulated |
| JAKMIP1 | 2.312084507 | 5.41E-56 | 8.21E-54 | upregulated |
| IGLV2-14 | 2.306644232 | 9.64E-18 | 1.80E-16 | upregulated |
| TRBV9 | 2.305690467 | 2.42E-44 | 1.51E-42 | upregulated |
| TRBV12-3 | 2.30409687 | 5.40E-33 | 1.89E-31 | upregulated |
| IGLV3-12 | 2.297358837 | 1.70E-15 | 2.82E-14 | upregulated |
| CCR2 | 2.296635703 | 7.41E-44 | 4.51E-42 | upregulated |
| TRAV9-2 | 2.29203239 | 1.96E-43 | 1.16E-41 | upregulated |
| IL2RG | 2.291740138 | 1.07E-65 | 1.25E-62 | upregulated |
| CD3G | 2.288622743 | 1.75E-58 | 3.68E-56 | upregulated |
| TRAV13-2 | 2.287719879 | 2.89E-49 | 2.50E-47 | upregulated |
| TRAV3 | 2.286917955 | 7.23E-41 | 3.63E-39 | upregulated |
| TRAV12-2 | 2.28490519 | 7.25E-46 | 5.01E-44 | upregulated |
| TRAV2 | 2.281714382 | 2.23E-45 | 1.50E-43 | upregulated |
| TRBJ2-1 | 2.278534556 | 1.55E-33 | 5.59E-32 | upregulated |
| CD3D | 2.277738006 | 8.44E-63 | 3.70E-60 | upregulated |
| TRBV11-2 | 2.277252947 | 2.79E-45 | 1.85E-43 | upregulated |
| IGKV1-13 | 2.275599842 | 1.24E-12 | 1.67E-11 | upregulated |
| TRAV14DV4 | 2.274644735 | 2.15E-35 | 8.43E-34 | upregulated |
| P2RY10 | 2.269016293 | 1.64E-48 | 1.36E-46 | upregulated |
| TLR10 | 2.268888562 | 1.51E-31 | 4.89E-30 | upregulated |
| ITGAL | 2.264211043 | 2.97E-63 | 1.39E-60 | upregulated |
| TLR7 | 2.264189421 | 1.17E-53 | 1.47E-51 | upregulated |
| TRAV29DV5 | 2.264124225 | 3.21E-43 | 1.87E-41 | upregulated |
| IGKV2OR22-3 | 2.264005842 | 1.21E-09 | 1.16E-08 | upregulated |
| TRGV2 | 2.26234723 | 9.65E-31 | 3.00E-29 | upregulated |
| LINC01857 | 2.2621895 | 9.34E-32 | 3.07E-30 | upregulated |
| IGHM | 2.261402073 | 2.09E-22 | 4.75E-21 | upregulated |
| IL21R | 2.256153645 | 8.32E-67 | 1.82E-63 | upregulated |
| TRAV10 | 2.255487201 | 7.26E-29 | 2.11E-27 | upregulated |
| WDFY4 | 2.255118177 | 1.22E-53 | 1.52E-51 | upregulated |
| BTLA | 2.25488091 | 2.64E-50 | 2.44E-48 | upregulated |
| TRAV16 | 2.250736326 | 1.26E-42 | 7.03E-41 | upregulated |
| IGKV1-16 | 2.249376541 | 3.60E-16 | 6.20E-15 | upregulated |
| TRBV5-6 | 2.247713226 | 1.27E-44 | 8.10E-43 | upregulated |
| AC006369.1 | 2.246635961 | 1.76E-33 | 6.30E-32 | upregulated |
| TRBJ2-2 | 2.246229935 | 4.34E-25 | 1.09E-23 | upregulated |
| TRAV8-6 | 2.242630417 | 1.68E-47 | 1.31E-45 | upregulated |
| TRAV5 | 2.23954224 | 4.82E-36 | 1.95E-34 | upregulated |
| TRBJ2-7 | 2.236826524 | 8.51E-38 | 3.77E-36 | upregulated |
| IGHV2-26 | 2.233170433 | 1.45E-12 | 1.94E-11 | upregulated |
| TRAV30 | 2.233002525 | 1.16E-23 | 2.76E-22 | upregulated |
| IGHV1OR15-2 | 2.232971111 | 7.61E-11 | 8.52E-10 | upregulated |
| CD3E | 2.225749193 | 7.62E-65 | 6.01E-62 | upregulated |
| HMGB2P1 | 2.222521375 | 2.18E-05 | 0.000101358 | upregulated |
| TRAV8-3 | 2.222432302 | 7.00E-43 | 3.97E-41 | upregulated |
| TRDV1 | 2.217710916 | 2.33E-30 | 7.15E-29 | upregulated |
| TRAV25 | 2.214398933 | 7.93E-37 | 3.38E-35 | upregulated |
| TRAV27 | 2.212636663 | 3.18E-36 | 1.30E-34 | upregulated |
| PTPRC | 2.212506827 | 1.72E-55 | 2.55E-53 | upregulated |
| GZMA | 2.211570471 | 3.63E-46 | 2.55E-44 | upregulated |
| IGHV3-21 | 2.209613631 | 3.45E-15 | 5.61E-14 | upregulated |
| IGLV4-69 | 2.209514743 | 9.09E-17 | 1.62E-15 | upregulated |
| TRAV8-4 | 2.209214022 | 3.26E-42 | 1.77E-40 | upregulated |
| IGHV1-69D | 2.207210595 | 1.02E-12 | 1.38E-11 | upregulated |
| UBASH3A | 2.205167366 | 3.25E-58 | 6.54E-56 | upregulated |
| CYBB | 2.205152455 | 8.86E-60 | 2.16E-57 | upregulated |
| GVINP1 | 2.20492341 | 2.72E-46 | 1.94E-44 | upregulated |
| TRBV20-1 | 2.204345283 | 1.41E-51 | 1.47E-49 | upregulated |
| IGLV9-49 | 2.203490372 | 2.03E-10 | 2.16E-09 | upregulated |
| TRAT1 | 2.203436501 | 9.87E-50 | 8.88E-48 | upregulated |
| TRBJ2-2P | 2.202188379 | 8.75E-23 | 2.02E-21 | upregulated |
| IRF8 | 2.202006769 | 1.86E-65 | 2.04E-62 | upregulated |
| TOMM20P2 | 2.19998775 | 7.17E-43 | 4.05E-41 | upregulated |
| ITK | 2.196800078 | 5.28E-55 | 7.49E-53 | upregulated |
| MIR4539 | 2.196618772 | 2.42E-15 | 3.99E-14 | upregulated |
| IGHV1-46 | 2.19627474 | 1.92E-15 | 3.18E-14 | upregulated |
| IGHV1-58 | 2.195961493 | 5.75E-11 | 6.52E-10 | upregulated |
| KCNA3 | 2.195021796 | 2.77E-32 | 9.39E-31 | upregulated |
| SLA2 | 2.18881629 | 1.74E-60 | 4.58E-58 | upregulated |
| CXCR6 | 2.184362803 | 1.80E-61 | 5.30E-59 | upregulated |
| GPR171 | 2.182973227 | 6.80E-50 | 6.21E-48 | upregulated |
| PTCRA | 2.180414214 | 3.67E-45 | 2.41E-43 | upregulated |
| P2RY13 | 2.179898697 | 3.58E-53 | 4.22E-51 | upregulated |
| C1QB | 2.176760274 | 1.86E-63 | 9.65E-61 | upregulated |
| GZMM | 2.17656258 | 9.07E-51 | 8.85E-49 | upregulated |
| IGHV4-61 | 2.174818524 | 6.47E-14 | 9.70E-13 | upregulated |
| TRAV19 | 2.174578359 | 1.70E-36 | 7.07E-35 | upregulated |
| CD27 | 2.173563684 | 2.74E-51 | 2.75E-49 | upregulated |
| GZMB | 2.172981417 | 5.97E-48 | 4.81E-46 | upregulated |
| AC083949.1 | 2.171368338 | 5.23E-43 | 3.01E-41 | upregulated |
| TRAV8-2 | 2.171190981 | 7.65E-42 | 4.06E-40 | upregulated |
| CD84 | 2.167201779 | 3.09E-53 | 3.69E-51 | upregulated |
| LYZ | 2.162531739 | 2.10E-40 | 1.03E-38 | upregulated |
| TBX21 | 2.15760766 | 2.56E-54 | 3.41E-52 | upregulated |
| TRBV24-1 | 2.157260661 | 9.05E-34 | 3.31E-32 | upregulated |
| IGKV6-21 | 2.157178021 | 3.74E-12 | 4.81E-11 | upregulated |
| IGLV5-48 | 2.156589406 | 5.31E-12 | 6.76E-11 | upregulated |
| IGLV2-18 | 2.156449898 | 5.59E-13 | 7.79E-12 | upregulated |
| TRAV17 | 2.156123631 | 2.62E-43 | 1.54E-41 | upregulated |
| IGHV2-70D | 2.155106533 | 5.15E-10 | 5.21E-09 | upregulated |
| SPN | 2.154258908 | 9.36E-59 | 2.05E-56 | upregulated |
| IGKV2D-29 | 2.152348044 | 7.74E-14 | 1.16E-12 | upregulated |
| C1QA | 2.150161069 | 9.08E-64 | 5.11E-61 | upregulated |
| TRDC | 2.150061317 | 5.26E-37 | 2.25E-35 | upregulated |
| AC243829.4 | 2.149410933 | 1.21E-45 | 8.25E-44 | upregulated |
| IGLVI-70 | 2.149184876 | 1.44E-07 | 1.02E-06 | upregulated |
| CD22 | 2.147705548 | 1.67E-21 | 3.67E-20 | upregulated |
| SELL | 2.147699417 | 1.83E-44 | 1.17E-42 | upregulated |
| SIGLEC10 | 2.145334327 | 2.66E-61 | 7.59E-59 | upregulated |
| IGKV3D-7 | 2.144499252 | 1.72E-09 | 1.62E-08 | upregulated |
| IGKV3D-20 | 2.14430438 | 1.07E-13 | 1.58E-12 | upregulated |
| IGHV7-81 | 2.142801808 | 5.15E-10 | 5.21E-09 | upregulated |
| AL928742.1 | 2.141509094 | 6.06E-15 | 9.70E-14 | upregulated |
| PLEK | 2.137403506 | 3.92E-55 | 5.64E-53 | upregulated |
| IGKV6D-21 | 2.133977124 | 1.02E-08 | 8.60E-08 | upregulated |
| TRBV7-3 | 2.130909965 | 8.80E-37 | 3.73E-35 | upregulated |
| P2RY12 | 2.128410003 | 8.27E-39 | 3.79E-37 | upregulated |
| CLECL1 | 2.128100483 | 1.03E-53 | 1.31E-51 | upregulated |
| TRBV5-4 | 2.127792013 | 4.48E-39 | 2.08E-37 | upregulated |
| IGHV1-18 | 2.126962555 | 3.96E-15 | 6.42E-14 | upregulated |
| FGL2 | 2.125784087 | 1.48E-61 | 4.50E-59 | upregulated |
| TRAC | 2.12359432 | 8.45E-62 | 2.79E-59 | upregulated |
| TIGIT | 2.121687818 | 1.62E-64 | 1.14E-61 | upregulated |
| AC015911.7 | 2.12146408 | 2.28E-48 | 1.87E-46 | upregulated |
| AC015911.3 | 2.120768054 | 3.95E-41 | 2.02E-39 | upregulated |
| IGKV1-33 | 2.120020912 | 1.78E-12 | 2.36E-11 | upregulated |
| FAM129C | 2.119874074 | 7.45E-20 | 1.53E-18 | upregulated |
| NAPSB | 2.11928252 | 8.11E-53 | 9.19E-51 | upregulated |
| MIR4538 | 2.118251476 | 8.93E-15 | 1.42E-13 | upregulated |
| LINC01871 | 2.117047589 | 1.19E-39 | 5.64E-38 | upregulated |
| IGHV4-39 | 2.114840212 | 1.11E-14 | 1.76E-13 | upregulated |
| IGLV1-51 | 2.1107767 | 4.32E-15 | 6.97E-14 | upregulated |
| IGKV2-29 | 2.109048225 | 1.15E-07 | 8.29E-07 | upregulated |
| IGLV1-36 | 2.107222947 | 6.93E-10 | 6.87E-09 | upregulated |
| TRAV6 | 2.107022742 | 4.69E-30 | 1.43E-28 | upregulated |
| TRGC1 | 2.105129654 | 1.66E-49 | 1.46E-47 | upregulated |
| IGKV2-30 | 2.10489892 | 7.70E-15 | 1.23E-13 | upregulated |
| IGLV3-10 | 2.102251673 | 2.92E-12 | 3.80E-11 | upregulated |
| TRAV1-1 | 2.096716841 | 3.81E-26 | 9.84E-25 | upregulated |
| IGKV1D-8 | 2.095636909 | 4.40E-11 | 5.07E-10 | upregulated |
| RNU1-61P | 2.094523071 | 2.88E-19 | 5.81E-18 | upregulated |
| SASH3 | 2.0925684 | 1.26E-70 | 2.49E-66 | upregulated |
| CLEC10A | 2.091819423 | 3.72E-44 | 2.30E-42 | upregulated |
| IGHV4-31 | 2.089574844 | 1.90E-12 | 2.52E-11 | upregulated |
| SAMD3 | 2.086535724 | 4.82E-51 | 4.75E-49 | upregulated |
| AL390755.1 | 2.08460878 | 0.000451255 | 0.001488551 | upregulated |
| HLA-DPA3 | 2.083338404 | 2.72E-28 | 7.72E-27 | upregulated |
| AL365361.1 | 2.083242771 | 1.97E-31 | 6.36E-30 | upregulated |
| IKZF1 | 2.081088694 | 1.99E-54 | 2.75E-52 | upregulated |
| IGKV2D-40 | 2.080672307 | 4.87E-09 | 4.31E-08 | upregulated |
| IGLV5-52 | 2.077517567 | 0.001445844 | 0.004131911 | upregulated |
| RUFY4 | 2.07540343 | 1.57E-40 | 7.83E-39 | upregulated |
| TBC1D10C | 2.07486822 | 1.10E-56 | 1.78E-54 | upregulated |
| IL12RB1 | 2.074774366 | 1.30E-61 | 4.07E-59 | upregulated |
| CD74 | 2.073819064 | 2.88E-63 | 1.38E-60 | upregulated |
| IGHV1-24 | 2.073691294 | 4.90E-13 | 6.85E-12 | upregulated |
| TRAV1-2 | 2.072654323 | 6.85E-32 | 2.27E-30 | upregulated |
| TRAV36DV7 | 2.071582639 | 9.68E-36 | 3.86E-34 | upregulated |
| NCKAP1L | 2.071270007 | 2.55E-65 | 2.40E-62 | upregulated |
| CD8B2 | 2.071145019 | 7.03E-09 | 6.07E-08 | upregulated |
| CD37 | 2.071001207 | 4.51E-61 | 1.24E-58 | upregulated |
| IGKV1-17 | 2.069307967 | 8.48E-13 | 1.16E-11 | upregulated |
| CD79A | 2.067088333 | 1.97E-18 | 3.82E-17 | upregulated |
| SLAMF1 | 2.066302261 | 2.04E-59 | 4.79E-57 | upregulated |
| AC246787.2 | 2.062823476 | 2.93E-15 | 4.79E-14 | upregulated |
| CD79B | 2.061678143 | 2.17E-37 | 9.46E-36 | upregulated |
| SIGLEC1 | 2.061437396 | 1.38E-56 | 2.21E-54 | upregulated |
| FCGR1A | 2.059529039 | 1.36E-53 | 1.67E-51 | upregulated |
| LINC02384 | 2.059114669 | 4.67E-28 | 1.32E-26 | upregulated |
| TRG-AS1 | 2.057203372 | 6.11E-55 | 8.60E-53 | upregulated |
| ENPP6 | 2.056150766 | 1.42E-14 | 2.24E-13 | upregulated |
| IGKV1D-43 | 2.054057716 | 4.90E-08 | 3.75E-07 | upregulated |
| HK3 | 2.054056996 | 1.21E-45 | 8.25E-44 | upregulated |
| LILRB1 | 2.047197072 | 1.53E-63 | 8.36E-61 | upregulated |
| IGHJ1 | 2.04666636 | 4.23E-10 | 4.35E-09 | upregulated |
| IGHV1-12 | 2.04593153 | 1.89E-11 | 2.26E-10 | upregulated |
| LINC00426 | 2.044776032 | 3.33E-50 | 3.06E-48 | upregulated |
| PI16 | 2.041512105 | 6.92E-12 | 8.67E-11 | upregulated |
| FCRL2 | 2.041505884 | 3.88E-17 | 7.05E-16 | upregulated |
| DLGAP1-AS5 | 2.041425595 | 1.66E-07 | 1.16E-06 | upregulated |
| TRBV10-2 | 2.039611972 | 1.54E-24 | 3.85E-23 | upregulated |
| DOCK2 | 2.036809049 | 1.78E-57 | 3.23E-55 | upregulated |
| IGHV3OR16-13 | 2.036339678 | 3.76E-13 | 5.30E-12 | upregulated |
| TRBJ2-3 | 2.035959423 | 2.81E-26 | 7.33E-25 | upregulated |
| IGLV7-46 | 2.035563874 | 1.87E-11 | 2.25E-10 | upregulated |
| CD52 | 2.032962928 | 1.02E-55 | 1.52E-53 | upregulated |
| IGLV7-43 | 2.032431086 | 1.96E-14 | 3.07E-13 | upregulated |
| IGHV3-30 | 2.030611886 | 7.82E-16 | 1.33E-14 | upregulated |
| IGKV1OR2-11 | 2.030074055 | 1.02E-08 | 8.59E-08 | upregulated |
| C1QC | 2.029300969 | 3.70E-64 | 2.33E-61 | upregulated |
| EVI2B | 2.029126461 | 2.21E-61 | 6.41E-59 | upregulated |
| IGKV1OR2-3 | 2.028570534 | 7.97E-08 | 5.89E-07 | upregulated |
| IGHV1OR16-1 | 2.028521648 | 9.32E-10 | 9.09E-09 | upregulated |
| IGHV3OR16-9 | 2.027369688 | 1.16E-13 | 1.71E-12 | upregulated |
| IGLV2-11 | 2.025997313 | 3.37E-13 | 4.79E-12 | upregulated |
| AC142381.1 | 2.025065037 | 3.30E-11 | 3.86E-10 | upregulated |
| FUT7 | 2.023311068 | 1.85E-53 | 2.22E-51 | upregulated |
| IGLV3-21 | 2.023294685 | 5.91E-15 | 9.46E-14 | upregulated |
| MPEG1 | 2.022494522 | 1.07E-57 | 2.06E-55 | upregulated |
| USP30-AS1 | 2.021703349 | 2.79E-45 | 1.85E-43 | upregulated |
| TNFRSF17 | 2.019597412 | 6.92E-18 | 1.31E-16 | upregulated |
| TMIGD2 | 2.018318778 | 2.41E-34 | 9.08E-33 | upregulated |
| IGKV3-20 | 2.017330185 | 1.86E-15 | 3.08E-14 | upregulated |
| LTA | 2.013403029 | 8.09E-52 | 8.58E-50 | upregulated |
| CD28 | 2.012976743 | 7.24E-53 | 8.30E-51 | upregulated |
| CR1 | 2.012364552 | 4.84E-21 | 1.05E-19 | upregulated |
| CD53 | 2.010837129 | 1.39E-64 | 1.01E-61 | upregulated |
| IGLV1-41 | 2.00896646 | 8.49E-13 | 1.16E-11 | upregulated |
| AF127936.1 | 2.003570393 | 5.84E-39 | 2.69E-37 | upregulated |
| IGKV1OR2-108 | 2.002382017 | 1.19E-07 | 8.56E-07 | upregulated |
| TFEC | 1.999536085 | 1.25E-53 | 1.54E-51 | upregulated |
| CCL18 | 1.99876497 | 1.60E-30 | 4.95E-29 | upregulated |
| PYHIN1 | 1.998716416 | 4.10E-51 | 4.06E-49 | upregulated |
| IGHV4-59 | 1.998507517 | 1.67E-13 | 2.44E-12 | upregulated |
| FCGR3A | 1.997379485 | 4.96E-53 | 5.82E-51 | upregulated |
| HLA-DQA1 | 1.995863697 | 2.07E-57 | 3.71E-55 | upregulated |
| GRIN2A | 1.993597211 | 6.88E-07 | 4.32E-06 | upregulated |
| IGHG1 | 1.993456766 | 1.97E-17 | 3.63E-16 | upregulated |
| PRKCB | 1.992202138 | 3.06E-51 | 3.06E-49 | upregulated |
| CTSW | 1.990793939 | 5.78E-42 | 3.10E-40 | upregulated |
| FCGR1B | 1.98939485 | 1.89E-47 | 1.47E-45 | upregulated |
| KLRB1 | 1.984817885 | 1.66E-42 | 9.15E-41 | upregulated |
| HCST | 1.982327323 | 4.46E-66 | 6.84E-63 | upregulated |
| HLA-DPA1 | 1.98229401 | 4.04E-57 | 6.87E-55 | upregulated |
| CD226 | 1.982228837 | 1.70E-58 | 3.61E-56 | upregulated |
| AC243960.1 | 1.979171462 | 7.75E-43 | 4.35E-41 | upregulated |
| IGHD3-9 | 1.978833619 | 1.77E-10 | 1.91E-09 | upregulated |
| SNX20 | 1.970507998 | 8.46E-65 | 6.42E-62 | upregulated |
| AL022316.1 | 1.970099998 | 1.61E-20 | 3.41E-19 | upregulated |
| FAM30A | 1.969616197 | 3.33E-19 | 6.70E-18 | upregulated |
| ICOS | 1.969379284 | 9.46E-62 | 3.01E-59 | upregulated |
| GBP4 | 1.968967706 | 2.20E-44 | 1.39E-42 | upregulated |
| APOC1 | 1.968439464 | 6.46E-30 | 1.95E-28 | upregulated |
| FATE1 | 1.967288684 | 6.67E-16 | 1.14E-14 | upregulated |
| TRGV3 | 1.966941076 | 1.07E-27 | 2.94E-26 | upregulated |
| IGLV2-23 | 1.966092539 | 2.30E-15 | 3.79E-14 | upregulated |
| LGI2 | 1.965012903 | 3.90E-11 | 4.52E-10 | upregulated |
| HLA-DPB1 | 1.96387258 | 3.46E-65 | 2.97E-62 | upregulated |
| IGLV1-44 | 1.963829066 | 1.05E-15 | 1.77E-14 | upregulated |
| NCR1 | 1.963392691 | 1.74E-36 | 7.24E-35 | upregulated |
| LINC02195 | 1.962454461 | 5.15E-43 | 2.97E-41 | upregulated |
| TRAV26-1 | 1.96131114 | 6.94E-42 | 3.70E-40 | upregulated |
| AC007991.4 | 1.960059001 | 6.90E-23 | 1.60E-21 | upregulated |
| LINC00996 | 1.958902542 | 1.52E-47 | 1.20E-45 | upregulated |
| SIGLEC14 | 1.958890268 | 2.47E-38 | 1.11E-36 | upregulated |
| LAX1 | 1.958711082 | 1.85E-29 | 5.48E-28 | upregulated |
| SPOCK2 | 1.957444905 | 8.43E-53 | 9.49E-51 | upregulated |
| TRGV4 | 1.949552191 | 1.22E-28 | 3.52E-27 | upregulated |
| PRSS51 | 1.949077785 | 0.012550302 | 0.026451844 | upregulated |
| PCED1B-AS1 | 1.949028761 | 2.77E-61 | 7.79E-59 | upregulated |
| GPR141 | 1.946383045 | 2.94E-44 | 1.83E-42 | upregulated |
| IKZF3 | 1.946227535 | 9.97E-42 | 5.24E-40 | upregulated |
| IGKV1-8 | 1.945539993 | 8.58E-16 | 1.46E-14 | upregulated |
| MMP2-AS1 | 1.944708649 | 1.14E-19 | 2.33E-18 | upregulated |
| CD4 | 1.944598848 | 1.66E-63 | 8.84E-61 | upregulated |
| AC018755.4 | 1.943840695 | 1.41E-33 | 5.10E-32 | upregulated |
| TESPA1 | 1.942723355 | 1.34E-57 | 2.54E-55 | upregulated |
| CCR4 | 1.942353895 | 3.46E-34 | 1.29E-32 | upregulated |
| IGHV2-5 | 1.940575694 | 6.18E-11 | 6.98E-10 | upregulated |
| SIT1 | 1.938979634 | 9.13E-54 | 1.18E-51 | upregulated |
| PIK3CG | 1.938146156 | 1.54E-36 | 6.43E-35 | upregulated |
| IGLC3 | 1.93684197 | 1.80E-18 | 3.51E-17 | upregulated |
| IGHV3-23 | 1.936037933 | 1.20E-15 | 2.02E-14 | upregulated |
| HLA-DMB | 1.935543935 | 1.61E-61 | 4.81E-59 | upregulated |
| CD96 | 1.935348594 | 1.23E-53 | 1.52E-51 | upregulated |
| ITGB2 | 1.934702028 | 1.59E-58 | 3.40E-56 | upregulated |
| LY9 | 1.933207644 | 3.52E-45 | 2.32E-43 | upregulated |
| CCR1 | 1.932994185 | 1.08E-54 | 1.51E-52 | upregulated |
| AC134879.2 | 1.929659871 | 5.44E-11 | 6.20E-10 | upregulated |
| IL10RA | 1.928297164 | 3.59E-64 | 2.33E-61 | upregulated |
| IGHV3-41 | 1.928192207 | 5.72E-10 | 5.76E-09 | upregulated |
| AC103563.3 | 1.926869787 | 3.05E-12 | 3.97E-11 | upregulated |
| IGHV3-76 | 1.925376332 | 1.30E-07 | 9.30E-07 | upregulated |
| IGLV2-28 | 1.924498593 | 2.96E-10 | 3.08E-09 | upregulated |
| IGKV3OR2-268 | 1.923816872 | 4.30E-13 | 6.04E-12 | upregulated |
| MNDA | 1.923502827 | 1.25E-62 | 5.35E-60 | upregulated |
| CXorf65 | 1.923097163 | 1.51E-33 | 5.44E-32 | upregulated |
| IGHJ2 | 1.923035825 | 2.27E-09 | 2.10E-08 | upregulated |
| AC103563.1 | 1.922718237 | 1.44E-12 | 1.93E-11 | upregulated |
| CD5 | 1.922363601 | 4.38E-52 | 4.72E-50 | upregulated |
| IGHV3-66 | 1.920953451 | 3.25E-13 | 4.62E-12 | upregulated |
| NFAM1 | 1.919133987 | 2.69E-58 | 5.47E-56 | upregulated |
| KLRD1 | 1.91646984 | 6.18E-44 | 3.79E-42 | upregulated |
| AC034105.1 | 1.91644081 | 1.37E-10 | 1.50E-09 | upregulated |
| IGKV2-26 | 1.916278964 | 6.57E-11 | 7.40E-10 | upregulated |
| CR2 | 1.913777212 | 6.92E-10 | 6.87E-09 | upregulated |
| TRAV39 | 1.912744871 | 8.50E-32 | 2.80E-30 | upregulated |
| LGALS2 | 1.911222359 | 5.79E-46 | 4.03E-44 | upregulated |
| CST7 | 1.907626228 | 2.94E-62 | 1.07E-59 | upregulated |
| TNFSF13B | 1.90310125 | 6.62E-60 | 1.63E-57 | upregulated |
| LTB | 1.903027967 | 2.82E-44 | 1.76E-42 | upregulated |
| IRF4 | 1.901761419 | 1.14E-37 | 4.98E-36 | upregulated |
| IGLV8-61 | 1.901649179 | 9.45E-13 | 1.29E-11 | upregulated |
| LAIR1 | 1.89936696 | 5.38E-62 | 1.83E-59 | upregulated |
| IGKV3-15 | 1.89917265 | 3.72E-16 | 6.40E-15 | upregulated |
| FCRL5 | 1.8990121 | 5.87E-17 | 1.06E-15 | upregulated |
| IL2RB | 1.898835215 | 1.65E-52 | 1.83E-50 | upregulated |
| IGKC | 1.897139508 | 2.86E-17 | 5.24E-16 | upregulated |
| TRAV8-1 | 1.895658408 | 5.41E-28 | 1.52E-26 | upregulated |
| CCL4 | 1.894887247 | 1.14E-53 | 1.44E-51 | upregulated |
| IGHV3-33 | 1.894311256 | 1.12E-15 | 1.90E-14 | upregulated |
| CYP27A1 | 1.893620451 | 4.42E-19 | 8.84E-18 | upregulated |
| IGLV5-45 | 1.892819606 | 8.08E-13 | 1.11E-11 | upregulated |
| HLA-DRB5 | 1.892682615 | 1.97E-51 | 2.01E-49 | upregulated |
| DNAJC5B | 1.892157606 | 3.88E-32 | 1.31E-30 | upregulated |
| HLA-DRB1 | 1.890448393 | 4.17E-62 | 1.44E-59 | upregulated |
| CD247 | 1.890144585 | 1.79E-58 | 3.71E-56 | upregulated |
| CD163 | 1.888706094 | 5.50E-41 | 2.78E-39 | upregulated |
| SIGLEC12 | 1.887967504 | 9.52E-24 | 2.28E-22 | upregulated |
| CALHM6 | 1.883901023 | 1.40E-48 | 1.18E-46 | upregulated |
| PPP1R16B | 1.882385706 | 8.72E-47 | 6.42E-45 | upregulated |
| IGLV3-9 | 1.880695928 | 1.46E-13 | 2.13E-12 | upregulated |
| APBB1IP | 1.878974153 | 8.13E-59 | 1.80E-56 | upregulated |
| HLA-DPB2 | 1.878247273 | 7.90E-22 | 1.76E-20 | upregulated |
| IGHGP | 1.876698992 | 4.50E-17 | 8.14E-16 | upregulated |
| SLA | 1.876508744 | 3.20E-64 | 2.18E-61 | upregulated |
| CHRNA6 | 1.875875221 | 1.45E-19 | 2.96E-18 | upregulated |
| CD300LF | 1.875820609 | 6.58E-61 | 1.78E-58 | upregulated |
| IGHV1-69 | 1.87441763 | 3.73E-12 | 4.79E-11 | upregulated |
| PRF1 | 1.874133643 | 1.45E-47 | 1.15E-45 | upregulated |
| TNFSF8 | 1.873531811 | 3.68E-43 | 2.14E-41 | upregulated |
| CD6 | 1.872826876 | 1.53E-53 | 1.86E-51 | upregulated |
| STAP1 | 1.872042882 | 1.03E-28 | 2.98E-27 | upregulated |
| ABCD2 | 1.872019309 | 1.13E-44 | 7.23E-43 | upregulated |
| TAGAP | 1.871671838 | 1.42E-49 | 1.26E-47 | upregulated |
| IGKV1-12 | 1.87120646 | 1.80E-12 | 2.39E-11 | upregulated |
| IGKV1OR2-6 | 1.869815651 | 4.26E-10 | 4.38E-09 | upregulated |
| IL4I1 | 1.868714136 | 3.97E-57 | 6.80E-55 | upregulated |
| IGLV1-40 | 1.86658965 | 1.70E-14 | 2.67E-13 | upregulated |
| PKDCC | 1.86658285 | 2.50E-08 | 1.99E-07 | upregulated |
| AC007991.2 | 1.86333364 | 8.40E-18 | 1.58E-16 | upregulated |
| IGLV3-25 | 1.86261741 | 3.09E-15 | 5.04E-14 | upregulated |
| IGKV1OR22-1 | 1.862423066 | 6.04E-10 | 6.04E-09 | upregulated |
| CHRM3-AS2 | 1.862414108 | 1.28E-33 | 4.63E-32 | upregulated |
| CD7 | 1.861982965 | 4.08E-59 | 9.35E-57 | upregulated |
| IGLV1-47 | 1.861478253 | 8.53E-14 | 1.27E-12 | upregulated |
| IGSF6 | 1.859037747 | 3.50E-62 | 1.23E-59 | upregulated |
| IGKV1-5 | 1.857730522 | 2.32E-15 | 3.82E-14 | upregulated |
| IGLL5 | 1.856290594 | 1.41E-17 | 2.62E-16 | upregulated |
| FPR3 | 1.854765684 | 1.68E-52 | 1.86E-50 | upregulated |
| AL139125.2 | 1.85314378 | 6.05E-35 | 2.34E-33 | upregulated |
| IGHV3-15 | 1.84996148 | 2.13E-15 | 3.53E-14 | upregulated |
| KLRC2 | 1.849254671 | 7.24E-18 | 1.37E-16 | upregulated |
| FCGR1CP | 1.848429459 | 1.72E-33 | 6.17E-32 | upregulated |
| IGHV3OR16-10 | 1.848258211 | 6.00E-08 | 4.51E-07 | upregulated |
| IGHV5-51 | 1.847983797 | 2.48E-12 | 3.24E-11 | upregulated |
| CXorf21 | 1.847363091 | 1.78E-56 | 2.83E-54 | upregulated |
| PLD4 | 1.847193835 | 8.43E-44 | 5.08E-42 | upregulated |
| HLA-DQA2 | 1.845853352 | 4.96E-27 | 1.33E-25 | upregulated |
| ARHGAP15 | 1.845510041 | 3.25E-65 | 2.91E-62 | upregulated |
| IGKV4-1 | 1.845180977 | 1.25E-15 | 2.10E-14 | upregulated |
| NUGGC | 1.843517257 | 2.17E-27 | 5.91E-26 | upregulated |
| IGKV1OR22-5 | 1.843276248 | 4.97E-09 | 4.39E-08 | upregulated |
| P2RY8 | 1.842277204 | 3.95E-41 | 2.02E-39 | upregulated |
| AC104699.1 | 1.842270475 | 2.04E-11 | 2.43E-10 | upregulated |
| SLCO2B1 | 1.841351628 | 4.30E-52 | 4.66E-50 | upregulated |
| VSIG4 | 1.840346297 | 4.50E-32 | 1.52E-30 | upregulated |
| CD72 | 1.838946694 | 1.92E-46 | 1.39E-44 | upregulated |
| MS4A6A | 1.838544436 | 5.19E-63 | 2.38E-60 | upregulated |
| IGKV1OR2-9 | 1.838229025 | 1.75E-10 | 1.88E-09 | upregulated |
| HLA-DRA | 1.837948821 | 9.23E-60 | 2.22E-57 | upregulated |
| FLT3 | 1.836285043 | 7.49E-40 | 3.60E-38 | upregulated |
| LILRB2 | 1.835051438 | 3.14E-56 | 4.91E-54 | upregulated |
| ADA2 | 1.83395148 | 5.92E-48 | 4.78E-46 | upregulated |
| IGKV1OR10-1 | 1.833378966 | 2.37E-11 | 2.81E-10 | upregulated |
| WAS | 1.832448587 | 4.26E-70 | 4.20E-66 | upregulated |
| MZB1 | 1.829294761 | 3.70E-19 | 7.44E-18 | upregulated |
| HAVCR2 | 1.829169166 | 6.79E-65 | 5.58E-62 | upregulated |
| IGKV3-11 | 1.828366374 | 7.66E-16 | 1.31E-14 | upregulated |
| CYTIP | 1.82780324 | 9.11E-58 | 1.78E-55 | upregulated |
| CLEC1A | 1.82607211 | 3.50E-23 | 8.24E-22 | upregulated |
| GRAP2 | 1.825168487 | 1.29E-51 | 1.34E-49 | upregulated |
| IGKV1OR9-2 | 1.823836556 | 2.30E-08 | 1.84E-07 | upregulated |
| GIMAP5 | 1.823827928 | 2.39E-44 | 1.50E-42 | upregulated |
| BATF2 | 1.821215326 | 2.59E-23 | 6.13E-22 | upregulated |
| GAPT | 1.820016106 | 4.58E-33 | 1.61E-31 | upregulated |
| C3AR1 | 1.819811235 | 2.65E-59 | 6.15E-57 | upregulated |
| IGKV2OR22-4 | 1.819323522 | 5.11E-10 | 5.17E-09 | upregulated |
| IGLJ2 | 1.818301186 | 1.44E-14 | 2.26E-13 | upregulated |
| SIGLEC7 | 1.81598378 | 2.74E-52 | 3.02E-50 | upregulated |
| PAK3 | 1.814333065 | 7.43E-18 | 1.40E-16 | upregulated |
| HLA-DOA | 1.813377852 | 8.71E-54 | 1.14E-51 | upregulated |
| IGHJ3 | 1.813136517 | 1.05E-12 | 1.42E-11 | upregulated |
| IGKV1-39 | 1.812948013 | 6.06E-15 | 9.69E-14 | upregulated |
| GPR65 | 1.812515489 | 1.77E-62 | 6.99E-60 | upregulated |
| IL18RAP | 1.810976072 | 5.92E-48 | 4.78E-46 | upregulated |
| DBH-AS1 | 1.810769205 | 5.88E-31 | 1.85E-29 | upregulated |
| CD180 | 1.809403241 | 4.99E-45 | 3.26E-43 | upregulated |
| CSF1R | 1.807381063 | 6.42E-60 | 1.60E-57 | upregulated |
| PLA2G2A | 1.805561718 | 8.76E-10 | 8.55E-09 | upregulated |
| IGHG3 | 1.804871296 | 2.97E-17 | 5.42E-16 | upregulated |
| CSF2RB | 1.804137995 | 2.07E-38 | 9.38E-37 | upregulated |
| TNFAIP8L2 | 1.802747333 | 4.07E-64 | 2.43E-61 | upregulated |
| IGHV1-2 | 1.80236725 | 1.08E-11 | 1.32E-10 | upregulated |
| SIRPB1 | 1.797981821 | 3.28E-28 | 9.26E-27 | upregulated |
| GLDC | 1.797321528 | 1.70E-09 | 1.60E-08 | upregulated |
| CD69 | 1.795878344 | 2.86E-46 | 2.03E-44 | upregulated |
| LY86 | 1.793115031 | 1.79E-55 | 2.63E-53 | upregulated |
| GIMAP7 | 1.792881162 | 5.01E-49 | 4.26E-47 | upregulated |
| IGLC7 | 1.787792624 | 1.77E-11 | 2.13E-10 | upregulated |
| AC004687.1 | 1.787412469 | 3.55E-22 | 7.97E-21 | upregulated |
| IGLJ1 | 1.787194577 | 2.24E-10 | 2.37E-09 | upregulated |
| CIITA | 1.786949896 | 2.01E-50 | 1.88E-48 | upregulated |
| LCP2 | 1.784795493 | 8.63E-62 | 2.79E-59 | upregulated |
| SLC7A7 | 1.784690696 | 1.82E-60 | 4.71E-58 | upregulated |
| IGLJ3 | 1.784534502 | 3.75E-09 | 3.36E-08 | upregulated |
| IGHV1OR15-9 | 1.783449148 | 6.16E-10 | 6.14E-09 | upregulated |
| IGKV1D-13 | 1.78269105 | 1.54E-09 | 1.46E-08 | upregulated |
| IGHV4-55 | 1.782630139 | 5.94E-11 | 6.73E-10 | upregulated |
| IGHV6-1 | 1.780912265 | 6.68E-15 | 1.07E-13 | upregulated |
| IGHV3-11 | 1.777910898 | 1.40E-12 | 1.88E-11 | upregulated |
| IGHV1-14 | 1.776666303 | 3.70E-12 | 4.76E-11 | upregulated |
| HLA-DRB6 | 1.776016889 | 8.75E-46 | 6.01E-44 | upregulated |
| ARHGAP9 | 1.775523807 | 3.78E-64 | 2.33E-61 | upregulated |
| CTSS | 1.774433007 | 9.40E-54 | 1.21E-51 | upregulated |
| IGKV3D-11 | 1.774374758 | 2.58E-12 | 3.38E-11 | upregulated |
| CMKLR1 | 1.773506428 | 2.49E-54 | 3.36E-52 | upregulated |
| CLEC4E | 1.773100536 | 2.48E-39 | 1.17E-37 | upregulated |
| IGHV4OR15-8 | 1.77167329 | 1.43E-08 | 1.18E-07 | upregulated |
| IL9R | 1.770623499 | 5.61E-42 | 3.02E-40 | upregulated |
| IGKV1OR1-1 | 1.769828182 | 1.22E-07 | 8.71E-07 | upregulated |
| RHOH | 1.767091435 | 1.42E-57 | 2.67E-55 | upregulated |
| NCR3 | 1.766630499 | 1.23E-35 | 4.87E-34 | upregulated |
| BTNL9 | 1.766526657 | 0.002038818 | 0.00558289 | upregulated |
| IGKV1-6 | 1.766299761 | 1.06E-11 | 1.31E-10 | upregulated |
| GIMAP1 | 1.765801116 | 1.00E-58 | 2.17E-56 | upregulated |
| CORO1A | 1.762755832 | 8.63E-62 | 2.79E-59 | upregulated |
| IGHV3-74 | 1.762720541 | 1.07E-15 | 1.80E-14 | upregulated |
| MS4A4A | 1.762714368 | 1.55E-46 | 1.13E-44 | upregulated |
| IGHV3-13 | 1.759715482 | 1.81E-12 | 2.41E-11 | upregulated |
| GIMAP4 | 1.758484849 | 2.95E-60 | 7.55E-58 | upregulated |
| TYROBP | 1.758315048 | 1.40E-62 | 5.87E-60 | upregulated |
| MYO1F | 1.756980821 | 9.75E-69 | 4.81E-65 | upregulated |
| LRRC25 | 1.755541527 | 8.59E-58 | 1.69E-55 | upregulated |
| IL27 | 1.755325462 | 4.50E-38 | 2.01E-36 | upregulated |
| RGS18 | 1.754030854 | 2.77E-50 | 2.55E-48 | upregulated |
| LINC02285 | 1.753440687 | 9.11E-39 | 4.16E-37 | upregulated |
| THEMIS | 1.75311512 | 1.63E-38 | 7.38E-37 | upregulated |
| FCRLA | 1.752525264 | 2.88E-17 | 5.26E-16 | upregulated |
| AC098613.1 | 1.752505175 | 3.16E-57 | 5.51E-55 | upregulated |
| SPI1 | 1.751494972 | 2.71E-69 | 1.78E-65 | upregulated |
| FERMT3 | 1.751233752 | 6.39E-66 | 9.00E-63 | upregulated |
| IL2RA | 1.750886601 | 2.90E-54 | 3.84E-52 | upregulated |
| LAPTM5 | 1.750123237 | 2.79E-63 | 1.37E-60 | upregulated |
| ZBP1 | 1.749007757 | 2.04E-43 | 1.20E-41 | upregulated |
| AIF1 | 1.747772527 | 2.20E-65 | 2.17E-62 | upregulated |
| MAP4K1 | 1.747338862 | 9.68E-50 | 8.75E-48 | upregulated |
| RNASE6 | 1.747103113 | 2.74E-66 | 4.91E-63 | upregulated |
| IGHV7-27 | 1.742896912 | 5.82E-10 | 5.83E-09 | upregulated |
| CD1B | 1.740361903 | 1.10E-28 | 3.16E-27 | upregulated |
| IGHV3-38 | 1.738991075 | 2.23E-09 | 2.07E-08 | upregulated |
| SLAMF8 | 1.738915482 | 3.11E-56 | 4.90E-54 | upregulated |
| CYTH4 | 1.738668894 | 2.05E-68 | 7.20E-65 | upregulated |
| FCER1G | 1.73638688 | 1.37E-61 | 4.21E-59 | upregulated |
| BTK | 1.735764327 | 1.98E-58 | 4.06E-56 | upregulated |
| CXCL13 | 1.735618972 | 1.06E-32 | 3.67E-31 | upregulated |
| IGLL3P | 1.733994578 | 0.000107505 | 0.000420317 | upregulated |
| KLRC1 | 1.733510522 | 1.10E-34 | 4.19E-33 | upregulated |
| CCR8 | 1.733132045 | 2.39E-33 | 8.54E-32 | upregulated |
| AL590764.1 | 1.729114761 | 7.41E-50 | 6.73E-48 | upregulated |
| IGLV2-33 | 1.728653532 | 9.46E-05 | 0.000375702 | upregulated |
| IGHV7-56 | 1.728560124 | 2.02E-07 | 1.40E-06 | upregulated |
| IGHV3-62 | 1.727848918 | 4.84E-12 | 6.17E-11 | upregulated |
| AC009974.1 | 1.724924972 | 7.86E-08 | 5.82E-07 | upregulated |
| FOXP3 | 1.724629389 | 3.70E-56 | 5.70E-54 | upregulated |
| IGHV1-68 | 1.72362781 | 8.15E-07 | 5.05E-06 | upregulated |
| LINC01402 | 1.723511573 | 0.003279401 | 0.008417557 | upregulated |
| IGLV2-5 | 1.723452601 | 2.19E-11 | 2.60E-10 | upregulated |
| SELPLG | 1.722990172 | 2.19E-68 | 7.20E-65 | upregulated |
| HLA-DQB1 | 1.721583601 | 5.85E-47 | 4.33E-45 | upregulated |
| IGKV2D-28 | 1.720910785 | 9.70E-10 | 9.42E-09 | upregulated |
| CTSE | 1.720604756 | 8.74E-05 | 0.000350595 | upregulated |
| EOMES | 1.718061322 | 4.99E-31 | 1.57E-29 | upregulated |
| ABI3 | 1.717453238 | 4.97E-68 | 1.40E-64 | upregulated |
| RASAL3 | 1.716765701 | 4.42E-57 | 7.32E-55 | upregulated |
| IGHV3-53 | 1.715749844 | 4.13E-12 | 5.29E-11 | upregulated |
| IGHV3OR16-16 | 1.714451728 | 7.72E-08 | 5.73E-07 | upregulated |
| EVI2A | 1.71402912 | 1.49E-57 | 2.78E-55 | upregulated |
| IGHV3-64 | 1.712955479 | 1.12E-11 | 1.37E-10 | upregulated |
| IGHV3-72 | 1.712629933 | 4.50E-13 | 6.31E-12 | upregulated |
| IGLC2 | 1.712411049 | 1.60E-16 | 2.82E-15 | upregulated |
| IGHV4-28 | 1.711831464 | 4.24E-12 | 5.41E-11 | upregulated |
| ICAM3 | 1.711597696 | 7.65E-40 | 3.67E-38 | upregulated |
| PARM1 | 1.70994358 | 4.80E-11 | 5.51E-10 | upregulated |
| TNFRSF9 | 1.709732709 | 1.59E-48 | 1.33E-46 | upregulated |
| CCL5 | 1.709385343 | 2.37E-45 | 1.59E-43 | upregulated |
| IGHV3OR16-8 | 1.7065584 | 1.84E-11 | 2.21E-10 | upregulated |
| CHI3L1 | 1.704531172 | 6.30E-16 | 1.07E-14 | upregulated |
| AC135068.2 | 1.704234122 | 4.97E-13 | 6.94E-12 | upregulated |
| LST1 | 1.702578941 | 8.02E-63 | 3.59E-60 | upregulated |
| LINC01480 | 1.701912951 | 3.21E-27 | 8.67E-26 | upregulated |
| ITGAM | 1.701873028 | 1.59E-28 | 4.54E-27 | upregulated |
| IGLV10-54 | 1.701065666 | 1.17E-11 | 1.43E-10 | upregulated |
| IGHV2-70 | 1.697009803 | 9.27E-12 | 1.14E-10 | upregulated |
| C11orf21 | 1.696817808 | 8.64E-44 | 5.16E-42 | upregulated |
| IGHA1 | 1.694359722 | 4.40E-14 | 6.68E-13 | upregulated |
| IGKV3-7 | 1.693900188 | 1.32E-11 | 1.61E-10 | upregulated |
| IGHV3OR15-7 | 1.69305307 | 6.41E-12 | 8.05E-11 | upregulated |
| CCR7 | 1.692545471 | 5.07E-41 | 2.57E-39 | upregulated |
| GNLY | 1.692112994 | 5.77E-30 | 1.75E-28 | upregulated |
| IGHV5-78 | 1.691951578 | 1.85E-08 | 1.51E-07 | upregulated |
| GPRIN3 | 1.690811399 | 1.88E-50 | 1.78E-48 | upregulated |
| SLAMF7 | 1.68954489 | 1.80E-45 | 1.21E-43 | upregulated |
| CD244 | 1.687572694 | 1.28E-48 | 1.08E-46 | upregulated |
| DOK2 | 1.686937693 | 2.68E-63 | 1.35E-60 | upregulated |
| LBP | 1.686471418 | 0.000496856 | 0.001620001 | upregulated |
| HLA-DQB2 | 1.68355885 | 1.32E-32 | 4.54E-31 | upregulated |
| IGKV1-27 | 1.68292437 | 1.41E-12 | 1.89E-11 | upregulated |
| JCHAIN | 1.681845015 | 1.30E-16 | 2.31E-15 | upregulated |
| LILRB5 | 1.6788184 | 1.16E-35 | 4.61E-34 | upregulated |
| CD209 | 1.677620295 | 1.04E-36 | 4.40E-35 | upregulated |
| IGKJ5 | 1.675346964 | 8.51E-12 | 1.05E-10 | upregulated |
| SUCNR1 | 1.6752635 | 4.22E-41 | 2.15E-39 | upregulated |
| SEPT1 | 1.671360272 | 2.44E-46 | 1.75E-44 | upregulated |
| IGKV1D-27 | 1.670040532 | 4.11E-09 | 3.66E-08 | upregulated |
| TNFRSF1B | 1.669919377 | 4.51E-66 | 6.84E-63 | upregulated |
| TMEM150B | 1.669683055 | 8.85E-45 | 5.70E-43 | upregulated |
| IL18BP | 1.669494999 | 1.83E-51 | 1.88E-49 | upregulated |
| IGHG2 | 1.669115411 | 4.01E-15 | 6.48E-14 | upregulated |
| ABCA3 | 1.668349014 | 0.005072819 | 0.012226492 | upregulated |
| PLA2G7 | 1.667092616 | 1.79E-46 | 1.30E-44 | upregulated |
| RF00397 | 1.66512379 | 4.10E-13 | 5.77E-12 | upregulated |
| IGHV4-34 | 1.663774604 | 2.77E-14 | 4.27E-13 | upregulated |
| LINC02345 | 1.661899454 | 5.41E-21 | 1.17E-19 | upregulated |
| HSH2D | 1.659431756 | 3.49E-39 | 1.63E-37 | upregulated |
| IGLV5-37 | 1.658752387 | 3.49E-07 | 2.32E-06 | upregulated |
| KIR2DL4 | 1.656526682 | 4.30E-27 | 1.16E-25 | upregulated |
| GPR82 | 1.655857031 | 7.73E-44 | 4.69E-42 | upregulated |
| HLA-DMA | 1.655411362 | 2.38E-57 | 4.22E-55 | upregulated |
| IGKV3D-15 | 1.655309755 | 4.83E-10 | 4.91E-09 | upregulated |
| BCL2L14 | 1.65382549 | 2.40E-31 | 7.72E-30 | upregulated |
| LILRA1 | 1.653634298 | 5.38E-43 | 3.08E-41 | upregulated |
| MS4A7 | 1.652721385 | 4.87E-51 | 4.78E-49 | upregulated |
| IGHV3-19 | 1.652700354 | 4.73E-10 | 4.81E-09 | upregulated |
| IGHJ3P | 1.651083552 | 5.03E-11 | 5.75E-10 | upregulated |
| LINC00582 | 1.650812911 | 2.11E-13 | 3.05E-12 | upregulated |
| LILRA6 | 1.648783354 | 4.44E-40 | 2.16E-38 | upregulated |
| CASS4 | 1.646869178 | 1.49E-45 | 1.01E-43 | upregulated |
| GNGT2 | 1.645932476 | 8.88E-66 | 1.17E-62 | upregulated |
| IGLV3-22 | 1.645032517 | 3.35E-07 | 2.23E-06 | upregulated |
| AC104971.3 | 1.644097645 | 1.02E-13 | 1.51E-12 | upregulated |
| ZC3H12D | 1.642438292 | 2.65E-48 | 2.17E-46 | upregulated |
| PTGDS | 1.642428555 | 3.57E-22 | 8.00E-21 | upregulated |
| BIN2 | 1.63865508 | 4.08E-57 | 6.88E-55 | upregulated |
| AC013264.1 | 1.638325181 | 3.23E-20 | 6.77E-19 | upregulated |
| PILRA | 1.636292483 | 1.67E-50 | 1.58E-48 | upregulated |
| TTC24 | 1.635153585 | 8.46E-28 | 2.34E-26 | upregulated |
| MGAT3 | 1.633852232 | 4.70E-06 | 2.51E-05 | upregulated |
| AL031846.1 | 1.632771019 | 1.23E-36 | 5.19E-35 | upregulated |
| IL16 | 1.630760704 | 8.59E-46 | 5.92E-44 | upregulated |
| MMP12 | 1.630586619 | 8.93E-25 | 2.24E-23 | upregulated |
| AC133644.2 | 1.628253025 | 1.26E-29 | 3.75E-28 | upregulated |
| ITGB2-AS1 | 1.627514905 | 2.16E-29 | 6.37E-28 | upregulated |
| SAA2-SAA4 | 1.625324528 | 8.49E-12 | 1.05E-10 | upregulated |
| IGHV3OR16-11 | 1.623090666 | 7.27E-09 | 6.27E-08 | upregulated |
| TMEM176B | 1.622401292 | 3.79E-52 | 4.15E-50 | upregulated |
| IGHV3-47 | 1.622357131 | 1.34E-10 | 1.46E-09 | upregulated |
| RPL32P1 | 1.622272401 | 2.71E-19 | 5.48E-18 | upregulated |
| CCL8 | 1.62162956 | 2.61E-23 | 6.17E-22 | upregulated |
| ZAP70 | 1.61979501 | 8.29E-42 | 4.39E-40 | upregulated |
| AC133065.3 | 1.617625217 | 1.73E-28 | 4.95E-27 | upregulated |
| CFP | 1.617185191 | 9.92E-38 | 4.38E-36 | upregulated |
| IGHV3-6 | 1.614682511 | 3.75E-08 | 2.92E-07 | upregulated |
| AL133467.1 | 1.614103701 | 3.71E-20 | 7.73E-19 | upregulated |
| AL133371.2 | 1.613503586 | 3.34E-37 | 1.43E-35 | upregulated |
| MILR1 | 1.612962856 | 1.12E-51 | 1.17E-49 | upregulated |
| LINCR-0001 | 1.610581414 | 0.000885853 | 0.002696544 | upregulated |
| POU2AF1 | 1.610237469 | 6.03E-13 | 8.39E-12 | upregulated |
| CTLA4 | 1.609120619 | 4.20E-60 | 1.06E-57 | upregulated |
| NCF1B | 1.608607155 | 5.03E-45 | 3.28E-43 | upregulated |
| TRGV5 | 1.608196576 | 1.44E-27 | 3.95E-26 | upregulated |
| CD300A | 1.608052276 | 1.67E-60 | 4.46E-58 | upregulated |
| HLA-DRB9 | 1.607255989 | 2.20E-29 | 6.48E-28 | upregulated |
| RNASE2 | 1.60439661 | 6.99E-35 | 2.69E-33 | upregulated |
| BATF | 1.602728425 | 5.57E-56 | 8.39E-54 | upregulated |
| SSTR2 | 1.602466182 | 2.50E-22 | 5.66E-21 | upregulated |
| IGLV3-19 | 1.601824197 | 3.72E-13 | 5.26E-12 | upregulated |
| BLK | 1.601700704 | 8.87E-18 | 1.66E-16 | upregulated |
| GIMAP6 | 1.600957954 | 4.60E-46 | 3.22E-44 | upregulated |
| IGKV7-3 | 1.599258014 | 7.21E-06 | 3.72E-05 | upregulated |
| AC109446.3 | 1.598282166 | 8.87E-26 | 2.27E-24 | upregulated |
| IGLV3-1 | 1.596099971 | 1.43E-13 | 2.09E-12 | upregulated |
| FCN1 | 1.595784096 | 1.65E-19 | 3.35E-18 | upregulated |
| DOCK8 | 1.594851105 | 4.29E-49 | 3.68E-47 | upregulated |
| IGKV1OR2-118 | 1.592619558 | 6.42E-07 | 4.06E-06 | upregulated |
| APOBEC3H | 1.592523538 | 1.87E-35 | 7.37E-34 | upregulated |
| CLEC9A | 1.590335244 | 8.47E-35 | 3.24E-33 | upregulated |
| PTPN22 | 1.5884098 | 1.85E-49 | 1.62E-47 | upregulated |
| IGHV3-65 | 1.586767144 | 7.45E-12 | 9.31E-11 | upregulated |
| PIK3R5 | 1.586516337 | 3.26E-56 | 5.06E-54 | upregulated |
| FGR | 1.584936175 | 1.87E-51 | 1.91E-49 | upregulated |
| AC136428.1 | 1.582445322 | 2.01E-08 | 1.63E-07 | upregulated |
| CCRL2 | 1.581012208 | 3.50E-62 | 1.23E-59 | upregulated |
| CEACAM4 | 1.579707569 | 9.25E-42 | 4.87E-40 | upregulated |
| CXCR4 | 1.578746828 | 9.78E-35 | 3.73E-33 | upregulated |
| GPR34 | 1.5783485 | 7.08E-46 | 4.92E-44 | upregulated |
| SAMSN1 | 1.576872737 | 4.42E-57 | 7.32E-55 | upregulated |
| LINC02576 | 1.575515603 | 1.05E-10 | 1.16E-09 | upregulated |
| LTF | 1.575280369 | 3.30E-07 | 2.20E-06 | upregulated |
| AC090559.1 | 1.574814864 | 8.07E-36 | 3.23E-34 | upregulated |
| PARP15 | 1.574542506 | 2.48E-33 | 8.82E-32 | upregulated |
| IGKV1D-42 | 1.572410794 | 2.44E-09 | 2.25E-08 | upregulated |
| NLRC3 | 1.571552238 | 1.02E-41 | 5.36E-40 | upregulated |
| AC006033.2 | 1.571300707 | 9.46E-43 | 5.28E-41 | upregulated |
| TENT5C | 1.570888228 | 6.84E-23 | 1.59E-21 | upregulated |
| IGHV3-49 | 1.570053872 | 1.73E-14 | 2.70E-13 | upregulated |
| HNRNPA1P21 | 1.56860498 | 5.88E-53 | 6.82E-51 | upregulated |
| IGLV3-13 | 1.567756885 | 2.86E-09 | 2.60E-08 | upregulated |
| CILP | 1.567436502 | 6.97E-07 | 4.37E-06 | upregulated |
| PDCD1LG2 | 1.566696866 | 1.40E-32 | 4.80E-31 | upregulated |
| CD80 | 1.565196201 | 3.38E-46 | 2.39E-44 | upregulated |
| MSR1 | 1.564870489 | 1.49E-31 | 4.83E-30 | upregulated |
| CEND1 | 1.562396902 | 5.21E-11 | 5.95E-10 | upregulated |
| GALNT17 | 1.561875387 | 1.59E-09 | 1.50E-08 | upregulated |
| CLEC4D | 1.560110166 | 2.39E-29 | 7.03E-28 | upregulated |
| GGTA1P | 1.559426324 | 1.05E-50 | 1.01E-48 | upregulated |
| TMEM178B | 1.558718108 | 3.09E-05 | 0.000138464 | upregulated |
| NCF4 | 1.558540452 | 1.70E-62 | 6.85E-60 | upregulated |
| IGLC6 | 1.557715712 | 2.04E-11 | 2.43E-10 | upregulated |
| IGHV3-71 | 1.554934759 | 1.79E-10 | 1.92E-09 | upregulated |
| CD14 | 1.552662444 | 2.09E-54 | 2.87E-52 | upregulated |
| CX3CR1 | 1.551445647 | 1.41E-31 | 4.60E-30 | upregulated |
| IGHV1-45 | 1.551417466 | 2.01E-09 | 1.88E-08 | upregulated |
| AC109826.1 | 1.550361777 | 1.34E-47 | 1.07E-45 | upregulated |
| LAIR2 | 1.547047486 | 9.49E-34 | 3.46E-32 | upregulated |
| AL139246.3 | 1.545046484 | 1.82E-16 | 3.21E-15 | upregulated |
| MRC1 | 1.544195322 | 1.09E-20 | 2.34E-19 | upregulated |
| AD000864.1 | 1.543611723 | 2.29E-46 | 1.65E-44 | upregulated |
| RASSF2 | 1.543304936 | 1.18E-39 | 5.64E-38 | upregulated |
| SP140 | 1.542245066 | 4.90E-41 | 2.49E-39 | upregulated |
| F5 | 1.54161309 | 4.39E-34 | 1.63E-32 | upregulated |
| C4B | 1.539582598 | 1.26E-21 | 2.80E-20 | upregulated |
| C16orf54 | 1.539276385 | 3.18E-36 | 1.30E-34 | upregulated |
| GPR84 | 1.537546832 | 1.62E-42 | 8.95E-41 | upregulated |
| MARCO | 1.536739453 | 2.93E-08 | 2.31E-07 | upregulated |
| AC119396.1 | 1.535387514 | 1.74E-08 | 1.42E-07 | upregulated |
| CCKAR | 1.534971904 | 5.09E-08 | 3.87E-07 | upregulated |
| CD300LB | 1.534890627 | 4.95E-32 | 1.66E-30 | upregulated |
| ERICH3 | 1.533522204 | 0.000508478 | 0.00165379 | upregulated |
| HLA-DQB1-AS1 | 1.531125654 | 2.36E-29 | 6.95E-28 | upregulated |
| FCGR2B | 1.530760147 | 5.07E-47 | 3.79E-45 | upregulated |
| AC012236.1 | 1.530680575 | 6.27E-12 | 7.87E-11 | upregulated |
| FO393401.1 | 1.530073134 | 1.04E-31 | 3.41E-30 | upregulated |
| GPSM3 | 1.526611474 | 2.00E-65 | 2.08E-62 | upregulated |
| ITGAX | 1.526244868 | 1.41E-41 | 7.33E-40 | upregulated |
| REM1 | 1.525962789 | 3.70E-12 | 4.76E-11 | upregulated |
| BCL2A1 | 1.525298408 | 2.25E-38 | 1.01E-36 | upregulated |
| IGHV3OR16-12 | 1.518728461 | 3.08E-06 | 1.70E-05 | upregulated |
| C19orf38 | 1.517321364 | 1.67E-50 | 1.58E-48 | upregulated |
| AC008105.3 | 1.516625208 | 1.06E-27 | 2.93E-26 | upregulated |
| C5orf56 | 1.514887825 | 1.82E-41 | 9.44E-40 | upregulated |
| AC245884.12 | 1.514415458 | 1.38E-13 | 2.02E-12 | upregulated |
| NLRP3 | 1.514000077 | 7.27E-35 | 2.79E-33 | upregulated |
| AC011899.2 | 1.513522348 | 8.72E-36 | 3.48E-34 | upregulated |
| FDCSP | 1.513254247 | 0.000215353 | 0.000776948 | upregulated |
| C1orf162 | 1.508204798 | 3.33E-61 | 9.23E-59 | upregulated |
| CCL4L2 | 1.505764691 | 1.04E-34 | 3.96E-33 | upregulated |
| AL023653.1 | 1.503095741 | 7.43E-26 | 1.90E-24 | upregulated |
| FAM78A | 1.502514958 | 5.06E-53 | 5.90E-51 | upregulated |
| P2RY14 | 1.502196964 | 1.04E-30 | 3.23E-29 | upregulated |
| CD300C | 1.498983916 | 1.33E-49 | 1.19E-47 | upregulated |
| RGS1 | 1.497504602 | 2.85E-36 | 1.17E-34 | upregulated |
| GMFG | 1.497318448 | 1.47E-62 | 6.05E-60 | upregulated |
| LINC02362 | 1.496409251 | 1.11E-13 | 1.64E-12 | upregulated |
| CMA1 | 1.494346582 | 4.50E-08 | 3.46E-07 | upregulated |
| LILRB3 | 1.494346564 | 1.07E-50 | 1.02E-48 | upregulated |
| NRIR | 1.493739305 | 1.45E-20 | 3.07E-19 | upregulated |
| CLEC4A | 1.493642815 | 1.25E-59 | 2.96E-57 | upregulated |
| CYSLTR1 | 1.493008061 | 1.14E-34 | 4.31E-33 | upregulated |
| SIGLEC9 | 1.492923698 | 1.50E-49 | 1.33E-47 | upregulated |
| LILRA2 | 1.49205685 | 6.08E-42 | 3.25E-40 | upregulated |
| LCP1 | 1.491569349 | 1.60E-48 | 1.34E-46 | upregulated |
| IGKV5-2 | 1.491385758 | 8.02E-14 | 1.20E-12 | upregulated |
| PEAK3 | 1.491298192 | 4.38E-46 | 3.08E-44 | upregulated |
| ARHGAP30 | 1.491025255 | 6.09E-59 | 1.36E-56 | upregulated |
| ADGRG5 | 1.490822165 | 4.49E-33 | 1.58E-31 | upregulated |
| MIR155HG | 1.490538789 | 7.04E-27 | 1.87E-25 | upregulated |
| WARS | 1.490394259 | 5.13E-23 | 1.20E-21 | upregulated |
| GBP1 | 1.490250623 | 9.11E-31 | 2.83E-29 | upregulated |
| AC004921.1 | 1.490031902 | 4.70E-32 | 1.58E-30 | upregulated |
| IGHV3-52 | 1.488046288 | 6.83E-08 | 5.10E-07 | upregulated |
| AQP9 | 1.487786781 | 1.85E-10 | 1.98E-09 | upregulated |
| CD1E | 1.486107898 | 3.96E-20 | 8.23E-19 | upregulated |
| GPR183 | 1.485909236 | 2.00E-43 | 1.18E-41 | upregulated |
| IGHV4-4 | 1.484877151 | 4.06E-12 | 5.21E-11 | upregulated |
| GPBAR1 | 1.484519751 | 6.92E-47 | 5.11E-45 | upregulated |
| SAA1 | 1.48370884 | 1.67E-11 | 2.02E-10 | upregulated |
| IGLV1-50 | 1.483573125 | 1.11E-07 | 8.03E-07 | upregulated |
| GAB3 | 1.481702305 | 9.85E-51 | 9.57E-49 | upregulated |
| ENPP2 | 1.479189244 | 7.38E-33 | 2.58E-31 | upregulated |
| FGD2 | 1.478346704 | 2.31E-50 | 2.14E-48 | upregulated |
| IGHV3-42 | 1.477438889 | 3.14E-09 | 2.84E-08 | upregulated |
| CCL21 | 1.477133897 | 4.79E-15 | 7.69E-14 | upregulated |
| LGALS9 | 1.476119069 | 2.05E-42 | 1.12E-40 | upregulated |
| MGAT3-AS1 | 1.474686315 | 0.00167631 | 0.004704597 | upregulated |
| SRGN | 1.473449587 | 7.45E-53 | 8.49E-51 | upregulated |
| CD274 | 1.472896414 | 3.10E-28 | 8.78E-27 | upregulated |
| APOL3 | 1.47013554 | 1.40E-42 | 7.79E-41 | upregulated |
| IGHV3-75 | 1.464347455 | 1.82E-09 | 1.70E-08 | upregulated |
| ALOX5 | 1.46392565 | 1.72E-41 | 8.93E-40 | upregulated |
| RUBCNL | 1.463396461 | 2.05E-36 | 8.47E-35 | upregulated |
| HS3ST4 | 1.463113818 | 0.000351233 | 0.001196036 | upregulated |
| RASSF4 | 1.46233194 | 1.59E-47 | 1.25E-45 | upregulated |
| IGLV3-29 | 1.459989195 | 3.30E-06 | 1.81E-05 | upregulated |
| RTP3 | 1.458036388 | 6.26E-06 | 3.28E-05 | upregulated |
| APOE | 1.457806368 | 2.15E-26 | 5.64E-25 | upregulated |
| MMRN1 | 1.457474711 | 4.68E-13 | 6.56E-12 | upregulated |
| ALOX5AP | 1.457014567 | 2.51E-54 | 3.37E-52 | upregulated |
| AC104964.3 | 1.454243285 | 0.000347484 | 0.001184087 | upregulated |
| CCL3 | 1.453030045 | 9.92E-33 | 3.44E-31 | upregulated |
| TMEM176A | 1.452948183 | 2.13E-47 | 1.63E-45 | upregulated |
| FMNL1 | 1.450950773 | 1.55E-57 | 2.84E-55 | upregulated |
| RCSD1 | 1.450054856 | 1.11E-45 | 7.59E-44 | upregulated |
| HVCN1 | 1.449612303 | 1.79E-39 | 8.48E-38 | upregulated |
| EXOC3L4 | 1.446998468 | 2.03E-12 | 2.68E-11 | upregulated |
| COL4A4 | 1.443576973 | 7.44E-11 | 8.34E-10 | upregulated |
| HSD11B1 | 1.442276694 | 1.60E-19 | 3.26E-18 | upregulated |
| PARVG | 1.440930976 | 1.09E-57 | 2.08E-55 | upregulated |
| APOBEC3G | 1.440531944 | 2.47E-35 | 9.68E-34 | upregulated |
| IGHV3-63 | 1.439184582 | 3.40E-10 | 3.52E-09 | upregulated |
| C4A | 1.43858765 | 1.07E-20 | 2.28E-19 | upregulated |
| IGHV3-25 | 1.4375114 | 5.54E-12 | 7.02E-11 | upregulated |
| AC015722.2 | 1.437377501 | 0.000281186 | 0.000983502 | upregulated |
| LCK | 1.437103655 | 2.84E-47 | 2.16E-45 | upregulated |
| AC012645.3 | 1.435670935 | 2.25E-22 | 5.10E-21 | upregulated |
| IPCEF1 | 1.435467111 | 8.87E-42 | 4.69E-40 | upregulated |
| AC119044.1 | 1.428577358 | 5.83E-43 | 3.33E-41 | upregulated |
| FOLR2 | 1.425100404 | 2.71E-34 | 1.01E-32 | upregulated |
| PPP1R1A | 1.424922448 | 2.60E-07 | 1.76E-06 | upregulated |
| PRAM1 | 1.424025204 | 7.93E-41 | 3.97E-39 | upregulated |
| ANGPTL7 | 1.422959698 | 4.14E-05 | 0.000179297 | upregulated |
| DERL3 | 1.4229016 | 3.88E-17 | 7.05E-16 | upregulated |
| S100B | 1.421117913 | 1.13E-31 | 3.69E-30 | upregulated |
| FMO2 | 1.419857962 | 4.40E-07 | 2.86E-06 | upregulated |
| NTRK1 | 1.41946398 | 1.84E-16 | 3.23E-15 | upregulated |
| IGKV1-9 | 1.418863532 | 2.66E-13 | 3.82E-12 | upregulated |
| CEACAM21 | 1.414002782 | 5.21E-45 | 3.39E-43 | upregulated |
| RAB42 | 1.412613927 | 2.71E-18 | 5.26E-17 | upregulated |
| CD86 | 1.410838518 | 7.97E-57 | 1.31E-54 | upregulated |
| HAMP | 1.410432355 | 3.24E-26 | 8.39E-25 | upregulated |
| LINC01150 | 1.409151411 | 1.93E-27 | 5.26E-26 | upregulated |
| IGHV3-37 | 1.408990635 | 4.06E-10 | 4.18E-09 | upregulated |
| TBXAS1 | 1.408987963 | 1.43E-54 | 1.98E-52 | upregulated |
| DCSTAMP | 1.408855175 | 3.85E-14 | 5.88E-13 | upregulated |
| TDRD6 | 1.408551823 | 1.05E-34 | 3.98E-33 | upregulated |
| IGHA2 | 1.40835661 | 1.60E-09 | 1.51E-08 | upregulated |
| SLFN12L | 1.405810742 | 7.22E-44 | 4.41E-42 | upregulated |
| CCL13 | 1.399605596 | 1.49E-24 | 3.71E-23 | upregulated |
| CSF3R | 1.399312684 | 1.16E-32 | 3.99E-31 | upregulated |
| NCF1C | 1.397765226 | 7.18E-41 | 3.61E-39 | upregulated |
| PTGES3P2 | 1.397604483 | 5.62E-20 | 1.16E-18 | upregulated |
| UPB1 | 1.396776326 | 2.48E-14 | 3.85E-13 | upregulated |
| ITM2A | 1.396708408 | 1.38E-35 | 5.44E-34 | upregulated |
| CCL23 | 1.396534454 | 2.48E-22 | 5.61E-21 | upregulated |
| IGHV3OR16-7 | 1.393864629 | 7.31E-06 | 3.77E-05 | upregulated |
| RAB37 | 1.390303645 | 6.75E-28 | 1.89E-26 | upregulated |
| FPR1 | 1.388429275 | 1.80E-33 | 6.43E-32 | upregulated |
| AC022182.2 | 1.387517503 | 1.62E-23 | 3.85E-22 | upregulated |
| IGHV1-67 | 1.385054237 | 1.49E-10 | 1.62E-09 | upregulated |
| PLA1A | 1.38459811 | 2.48E-24 | 6.14E-23 | upregulated |
| RASGRP2 | 1.384151587 | 2.40E-30 | 7.36E-29 | upregulated |
| KCNJ10 | 1.383901957 | 2.44E-28 | 6.94E-27 | upregulated |
| ARHGEF6 | 1.381600092 | 5.77E-40 | 2.79E-38 | upregulated |
| AOAH | 1.381295886 | 4.61E-49 | 3.94E-47 | upregulated |
| KLRG1 | 1.37895316 | 5.60E-31 | 1.76E-29 | upregulated |
| C2 | 1.378738402 | 1.71E-47 | 1.33E-45 | upregulated |
| AC022509.1 | 1.37760496 | 0.004210449 | 0.010428518 | upregulated |
| AC006237.1 | 1.377601096 | 4.41E-06 | 2.37E-05 | upregulated |
| RSPO1 | 1.377402824 | 5.42E-11 | 6.18E-10 | upregulated |
| CASP5 | 1.37645347 | 1.79E-30 | 5.51E-29 | upregulated |
| SUSD2 | 1.374770262 | 1.01E-10 | 1.12E-09 | upregulated |
| TRGV7 | 1.373131108 | 3.23E-30 | 9.87E-29 | upregulated |
| TRAF3IP3 | 1.37159204 | 6.65E-43 | 3.78E-41 | upregulated |
| IGHG4 | 1.369528189 | 2.87E-09 | 2.61E-08 | upregulated |
| IL1R2 | 1.367882554 | 0.011494172 | 0.024586004 | upregulated |
| FCGR2C | 1.367423159 | 2.93E-29 | 8.59E-28 | upregulated |
| CSF2RA | 1.36708157 | 1.01E-50 | 9.79E-49 | upregulated |
| TNFRSF4 | 1.367011785 | 1.07E-37 | 4.74E-36 | upregulated |
| ITGB7 | 1.365436643 | 2.07E-33 | 7.40E-32 | upregulated |
| ETV7 | 1.363322863 | 1.51E-32 | 5.15E-31 | upregulated |
| MAJIN | 1.36295758 | 2.31E-07 | 1.58E-06 | upregulated |
| SELP | 1.362580586 | 1.32E-15 | 2.21E-14 | upregulated |
| IGHVIII-51-1 | 1.357522664 | 1.08E-07 | 7.81E-07 | upregulated |
| PIK3R6 | 1.357368216 | 1.99E-47 | 1.53E-45 | upregulated |
| CCL7 | 1.354987927 | 2.30E-16 | 4.00E-15 | upregulated |
| SAMHD1 | 1.353868365 | 1.33E-49 | 1.19E-47 | upregulated |
| ABI3BP | 1.353857349 | 6.79E-12 | 8.52E-11 | upregulated |
| RIPOR2 | 1.353326776 | 2.98E-34 | 1.11E-32 | upregulated |
| TXK | 1.350357784 | 4.33E-29 | 1.26E-27 | upregulated |
| VAV1 | 1.348476594 | 5.34E-44 | 3.29E-42 | upregulated |
| TLR4 | 1.348234142 | 2.07E-31 | 6.68E-30 | upregulated |
| XCL2 | 1.344885707 | 3.98E-24 | 9.76E-23 | upregulated |
| ARMH1 | 1.34133945 | 1.10E-23 | 2.63E-22 | upregulated |
| TM6SF1 | 1.340323474 | 3.37E-36 | 1.37E-34 | upregulated |
| CSF1 | 1.340101697 | 1.95E-50 | 1.83E-48 | upregulated |
| CLEC2D | 1.339898299 | 3.31E-35 | 1.29E-33 | upregulated |
| ADRA2A | 1.339344791 | 2.27E-09 | 2.10E-08 | upregulated |
| LILRA5 | 1.339163027 | 5.33E-32 | 1.78E-30 | upregulated |
| FAM92B | 1.338829346 | 1.95E-09 | 1.82E-08 | upregulated |
| GHRL | 1.337445366 | 1.59E-22 | 3.65E-21 | upregulated |
| GPR55 | 1.335431814 | 1.80E-24 | 4.47E-23 | upregulated |
| PTGIS | 1.335395945 | 5.24E-12 | 6.67E-11 | upregulated |
| LINC01943 | 1.333759667 | 1.67E-46 | 1.21E-44 | upregulated |
| IL22RA2 | 1.332952167 | 2.02E-16 | 3.54E-15 | upregulated |
| ADARB2 | 1.331924818 | 1.99E-06 | 1.14E-05 | upregulated |
| VNN2 | 1.331854936 | 1.86E-26 | 4.89E-25 | upregulated |
| DOK3 | 1.330181481 | 1.92E-49 | 1.68E-47 | upregulated |
| TMEM273 | 1.329978292 | 1.41E-44 | 9.02E-43 | upregulated |
| AC021188.1 | 1.329224232 | 5.65E-32 | 1.88E-30 | upregulated |
| IGHV1-17 | 1.327560898 | 3.43E-11 | 4.00E-10 | upregulated |
| NRROS | 1.326123093 | 6.35E-38 | 2.82E-36 | upregulated |
| IGFLR1 | 1.325075502 | 5.86E-39 | 2.69E-37 | upregulated |
| RARRES3 | 1.32244328 | 1.59E-28 | 4.54E-27 | upregulated |
| C5AR1 | 1.321141552 | 3.99E-31 | 1.27E-29 | upregulated |
| P2RX1 | 1.320114806 | 5.53E-30 | 1.68E-28 | upregulated |
| KCNJ5 | 1.319603808 | 5.43E-19 | 1.08E-17 | upregulated |
| PDE6B | 1.317259111 | 1.12E-12 | 1.51E-11 | upregulated |
| RSAD2 | 1.314689332 | 4.04E-23 | 9.44E-22 | upregulated |
| FYB1 | 1.314606603 | 6.35E-36 | 2.55E-34 | upregulated |
| NEURL3 | 1.313691175 | 7.29E-13 | 1.01E-11 | upregulated |
| PREX1 | 1.313555614 | 1.58E-42 | 8.75E-41 | upregulated |
| AC007728.2 | 1.312733065 | 2.97E-37 | 1.28E-35 | upregulated |
| CYP1B1 | 1.31247768 | 4.23E-10 | 4.35E-09 | upregulated |
| Z95114.3 | 1.311838195 | 1.29E-28 | 3.70E-27 | upregulated |
| MAP1LC3C | 1.306503627 | 9.90E-16 | 1.68E-14 | upregulated |
| CASP17P | 1.303868226 | 2.21E-37 | 9.59E-36 | upregulated |
| AC004988.1 | 1.303336173 | 2.98E-14 | 4.59E-13 | upregulated |
| ADH1B | 1.301951282 | 3.79E-11 | 4.40E-10 | upregulated |
| GPR15 | 1.301411701 | 5.21E-09 | 4.58E-08 | upregulated |
| PSMB9 | 1.301320223 | 1.06E-31 | 3.46E-30 | upregulated |
| PLCB2 | 1.300953744 | 5.83E-45 | 3.78E-43 | upregulated |
| AC008957.1 | 1.299283012 | 4.57E-17 | 8.27E-16 | upregulated |
| ADGRG2 | 1.298939147 | 0.022448214 | 0.042929923 | upregulated |
| CD200R1 | 1.298282555 | 1.04E-29 | 3.12E-28 | upregulated |
| PNOC | 1.296192512 | 3.03E-14 | 4.67E-13 | upregulated |
| FCGBP | 1.296152248 | 3.29E-08 | 2.58E-07 | upregulated |
| SELE | 1.294363762 | 9.11E-07 | 5.59E-06 | upregulated |
| CCDC170 | 1.294200457 | 7.73E-28 | 2.15E-26 | upregulated |
| GFRA2 | 1.292030512 | 3.18E-22 | 7.17E-21 | upregulated |
| TRAF1 | 1.291855323 | 9.15E-33 | 3.19E-31 | upregulated |
| INPP5D | 1.289733097 | 5.40E-40 | 2.62E-38 | upregulated |
| GNG8 | 1.289222386 | 2.53E-21 | 5.53E-20 | upregulated |
| IRF1 | 1.289121422 | 3.14E-39 | 1.47E-37 | upregulated |
| GIMAP2 | 1.287649228 | 2.30E-41 | 1.18E-39 | upregulated |
| AC017002.3 | 1.285487632 | 1.83E-36 | 7.59E-35 | upregulated |
| NCF1 | 1.285318026 | 3.13E-42 | 1.70E-40 | upregulated |
| AKAP5 | 1.284561924 | 3.01E-32 | 1.02E-30 | upregulated |
| IGHV3-35 | 1.283613399 | 3.39E-08 | 2.66E-07 | upregulated |
| Z84484.1 | 1.282381465 | 1.25E-18 | 2.44E-17 | upregulated |
| FCMR | 1.281599267 | 6.06E-30 | 1.83E-28 | upregulated |
| COL6A6 | 1.280141463 | 1.91E-12 | 2.53E-11 | upregulated |
| ANGPTL1 | 1.27985905 | 9.49E-14 | 1.41E-12 | upregulated |
| SOWAHD | 1.277706449 | 4.91E-34 | 1.82E-32 | upregulated |
| SLC8A1 | 1.277624468 | 1.92E-36 | 7.94E-35 | upregulated |
| MIR3945HG | 1.276508014 | 2.78E-10 | 2.91E-09 | upregulated |
| CD33 | 1.27616122 | 8.65E-44 | 5.16E-42 | upregulated |
| IGHV3-16 | 1.275649569 | 1.24E-07 | 8.91E-07 | upregulated |
| STAB1 | 1.275173838 | 4.98E-43 | 2.88E-41 | upregulated |
| AKNA | 1.271576338 | 7.75E-43 | 4.35E-41 | upregulated |
| IGKV1OR-3 | 1.270520073 | 6.25E-08 | 4.69E-07 | upregulated |
| FPR2 | 1.26987857 | 8.80E-13 | 1.20E-11 | upregulated |
| XCR1 | 1.265863951 | 9.36E-21 | 2.00E-19 | upregulated |
| RASGRP4 | 1.263118346 | 2.65E-43 | 1.55E-41 | upregulated |
| AC008750.1 | 1.262933893 | 4.09E-20 | 8.50E-19 | upregulated |
| FCGR2A | 1.262568106 | 8.77E-37 | 3.72E-35 | upregulated |
| SECTM1 | 1.260974948 | 1.47E-33 | 5.31E-32 | upregulated |
| ACAP1 | 1.25937971 | 2.83E-45 | 1.87E-43 | upregulated |
| IFIT3 | 1.258995772 | 9.01E-23 | 2.08E-21 | upregulated |
| DOCK10 | 1.25864575 | 2.45E-37 | 1.06E-35 | upregulated |
| IRX6 | 1.257773695 | 0.001875546 | 0.005182597 | upregulated |
| GIMAP8 | 1.257147771 | 2.78E-37 | 1.20E-35 | upregulated |
| CARMIL2 | 1.257024151 | 1.46E-32 | 4.97E-31 | upregulated |
| HTRA4 | 1.256183591 | 4.24E-19 | 8.50E-18 | upregulated |
| ATP2B2 | 1.255114225 | 8.79E-07 | 5.41E-06 | upregulated |
| IGHV3-7 | 1.253756315 | 3.69E-13 | 5.22E-12 | upregulated |
| TNFRSF14-AS1 | 1.253717893 | 1.73E-26 | 4.56E-25 | upregulated |
| SERPING1 | 1.251795044 | 5.44E-36 | 2.19E-34 | upregulated |
| SDS | 1.250922021 | 2.65E-21 | 5.79E-20 | upregulated |
| PLXNC1 | 1.247914259 | 1.88E-30 | 5.80E-29 | upregulated |
| LAT2 | 1.24782489 | 1.55E-57 | 2.84E-55 | upregulated |
| RGL4 | 1.247128584 | 8.42E-31 | 2.63E-29 | upregulated |
| IGHV3-79 | 1.246221117 | 8.89E-07 | 5.46E-06 | upregulated |
| MS4A14 | 1.2460602 | 2.44E-32 | 8.28E-31 | upregulated |
| ANKRD44 | 1.245692908 | 2.75E-35 | 1.08E-33 | upregulated |
| CYP4Z1 | 1.245311277 | 0.000856305 | 0.002619545 | upregulated |
| 1-Mar | 1.245305574 | 1.18E-41 | 6.16E-40 | upregulated |
| KLRF1 | 1.244992908 | 5.92E-20 | 1.22E-18 | upregulated |
| THBS4 | 1.243336006 | 6.52E-07 | 4.11E-06 | upregulated |
| SUSD3 | 1.243116892 | 3.20E-38 | 1.44E-36 | upregulated |
| TMPRSS3 | 1.243103575 | 4.53E-25 | 1.14E-23 | upregulated |
| CD1C | 1.243101927 | 7.83E-22 | 1.74E-20 | upregulated |
| IGHV3OR16-15 | 1.241415472 | 3.65E-09 | 3.27E-08 | upregulated |
| CEACAM3 | 1.239079896 | 2.05E-24 | 5.10E-23 | upregulated |
| AP002954.1 | 1.238845604 | 2.67E-17 | 4.91E-16 | upregulated |
| THEMIS2 | 1.238062383 | 6.72E-53 | 7.74E-51 | upregulated |
| PIM2 | 1.235783021 | 9.25E-21 | 1.98E-19 | upregulated |
| PTGER2 | 1.235740262 | 3.81E-28 | 1.07E-26 | upregulated |
| AL357054.4 | 1.235664951 | 3.07E-27 | 8.31E-26 | upregulated |
| SIGLEC6 | 1.235536993 | 4.59E-14 | 6.94E-13 | upregulated |
| LINC01914 | 1.234990245 | 8.85E-14 | 1.32E-12 | upregulated |
| RAB33A | 1.234973585 | 2.55E-49 | 2.22E-47 | upregulated |
| APOL4 | 1.234804635 | 3.73E-23 | 8.73E-22 | upregulated |
| TRIM22 | 1.233442274 | 2.91E-31 | 9.31E-30 | upregulated |
| GFRA1 | 1.233272844 | 1.57E-09 | 1.49E-08 | upregulated |
| CELF2 | 1.232431671 | 7.66E-34 | 2.81E-32 | upregulated |
| APOBR | 1.229668539 | 4.48E-55 | 6.41E-53 | upregulated |
| BHLHE22 | 1.228533335 | 2.30E-16 | 4.00E-15 | upregulated |
| ADAM28 | 1.227835959 | 1.61E-22 | 3.67E-21 | upregulated |
| TRPV2 | 1.226113786 | 7.70E-45 | 4.98E-43 | upregulated |
| ZNF366 | 1.224213508 | 1.03E-15 | 1.73E-14 | upregulated |
| EBI3 | 1.223521076 | 2.62E-40 | 1.28E-38 | upregulated |
| RNF150 | 1.222829761 | 1.52E-11 | 1.85E-10 | upregulated |
| CBLN2 | 1.22209258 | 0.013294067 | 0.027746526 | upregulated |
| HS3ST2 | 1.222046641 | 2.25E-09 | 2.08E-08 | upregulated |
| EPSTI1 | 1.221478933 | 6.34E-29 | 1.84E-27 | upregulated |
| DPT | 1.216130193 | 4.76E-15 | 7.66E-14 | upregulated |
| IGHV3-48 | 1.21443958 | 8.52E-15 | 1.36E-13 | upregulated |
| SOX8 | 1.211281476 | 0.001263226 | 0.003667987 | upregulated |
| IQGAP2 | 1.210905025 | 1.94E-34 | 7.31E-33 | upregulated |
| FCAR | 1.207617592 | 2.45E-05 | 0.000112562 | upregulated |
| AC015911.8 | 1.206638871 | 8.52E-28 | 2.36E-26 | upregulated |
| JAK3 | 1.206283549 | 1.57E-32 | 5.34E-31 | upregulated |
| CD163L1 | 1.202714087 | 2.00E-22 | 4.56E-21 | upregulated |
| NFATC2 | 1.201685593 | 1.07E-32 | 3.69E-31 | upregulated |
| SAA2 | 1.200734701 | 3.49E-08 | 2.72E-07 | upregulated |
| CCL24 | 1.198616004 | 4.09E-10 | 4.21E-09 | upregulated |
| TMIGD3 | 1.197152356 | 5.10E-32 | 1.71E-30 | upregulated |
| LINC01315 | 1.197069649 | 0.00265731 | 0.00701534 | upregulated |
| ATP6V0D2 | 1.196236159 | 3.63E-07 | 2.40E-06 | upregulated |
| AC010175.1 | 1.192618578 | 3.46E-09 | 3.12E-08 | upregulated |
| OSM | 1.192457196 | 5.36E-12 | 6.80E-11 | upregulated |
| CD300E | 1.190495599 | 2.19E-13 | 3.17E-12 | upregulated |
| FFAR4 | 1.190408016 | 3.39E-24 | 8.33E-23 | upregulated |
| ISG20 | 1.190362496 | 2.52E-31 | 8.08E-30 | upregulated |
| IL32 | 1.188223137 | 3.82E-27 | 1.03E-25 | upregulated |
| LINC01215 | 1.187983053 | 1.33E-14 | 2.10E-13 | upregulated |
| ANKRD36BP2 | 1.186171843 | 8.99E-09 | 7.66E-08 | upregulated |
| KLHL6 | 1.185540264 | 9.64E-30 | 2.90E-28 | upregulated |
| ATP8B4 | 1.182877868 | 2.42E-44 | 1.51E-42 | upregulated |
| CLC | 1.181970872 | 2.06E-07 | 1.42E-06 | upregulated |
| AL807752.1 | 1.18196609 | 0.001256455 | 0.003649943 | upregulated |
| IGKV1D-16 | 1.181669427 | 7.21E-12 | 9.03E-11 | upregulated |
| LPL | 1.181323503 | 1.08E-07 | 7.80E-07 | upregulated |
| HCK | 1.179803549 | 1.56E-39 | 7.40E-38 | upregulated |
| AL512306.2 | 1.179766058 | 5.06E-09 | 4.47E-08 | upregulated |
| MCOLN2 | 1.179101291 | 3.33E-35 | 1.30E-33 | upregulated |
| FBP1 | 1.178804975 | 6.24E-18 | 1.19E-16 | upregulated |
| AL590648.3 | 1.178150012 | 3.20E-20 | 6.72E-19 | upregulated |
| JAK2 | 1.177606008 | 3.34E-33 | 1.18E-31 | upregulated |
| AP003774.4 | 1.177554099 | 4.11E-20 | 8.53E-19 | upregulated |
| FLI1 | 1.177356157 | 1.89E-40 | 9.32E-39 | upregulated |
| SKAP1 | 1.175939788 | 5.86E-40 | 2.83E-38 | upregulated |
| AL158071.2 | 1.175689412 | 2.54E-08 | 2.02E-07 | upregulated |
| KCTD14 | 1.175029176 | 5.42E-05 | 0.000228389 | upregulated |
| SAMD9L | 1.174307711 | 6.65E-31 | 2.08E-29 | upregulated |
| CHST13 | 1.173469463 | 1.88E-26 | 4.93E-25 | upregulated |
| SFTPA1 | 1.173031396 | 0.00028849 | 0.001005302 | upregulated |
| MEI1 | 1.17269711 | 5.37E-27 | 1.44E-25 | upregulated |
| TMEM156 | 1.171825055 | 4.67E-35 | 1.81E-33 | upregulated |
| FCGR3B | 1.171279262 | 8.27E-09 | 7.09E-08 | upregulated |
| WIPF1 | 1.170242667 | 2.82E-37 | 1.22E-35 | upregulated |
| TDRD10 | 1.170231458 | 4.93E-10 | 5.00E-09 | upregulated |
| DPEP2 | 1.170036819 | 1.63E-30 | 5.05E-29 | upregulated |
| GBP2 | 1.169565378 | 1.07E-35 | 4.24E-34 | upregulated |
| CMPK2 | 1.16944377 | 1.21E-24 | 3.03E-23 | upregulated |
| LSAMP | 1.169150958 | 4.23E-07 | 2.76E-06 | upregulated |
| PRELP | 1.16877298 | 4.20E-12 | 5.37E-11 | upregulated |
| AL161935.3 | 1.168454679 | 4.10E-15 | 6.62E-14 | upregulated |
| XIRP1 | 1.166321028 | 3.18E-10 | 3.30E-09 | upregulated |
| CCL2 | 1.166113905 | 9.14E-21 | 1.96E-19 | upregulated |
| VPREB3 | 1.16544718 | 1.36E-05 | 6.64E-05 | upregulated |
| IGKV2-4 | 1.162709802 | 4.44E-06 | 2.38E-05 | upregulated |
| AL157871.2 | 1.16244303 | 2.02E-08 | 1.63E-07 | upregulated |
| IL10 | 1.157528719 | 2.07E-28 | 5.90E-27 | upregulated |
| APOBEC3D | 1.153833624 | 2.87E-26 | 7.50E-25 | upregulated |
| AC131097.4 | 1.153785015 | 7.97E-17 | 1.43E-15 | upregulated |
| IGHV1OR21-1 | 1.153538681 | 6.62E-10 | 6.58E-09 | upregulated |
| UBA7 | 1.153486308 | 1.77E-40 | 8.77E-39 | upregulated |
| IGHE | 1.152314557 | 3.50E-08 | 2.73E-07 | upregulated |
| IFI44L | 1.15127048 | 5.70E-21 | 1.23E-19 | upregulated |
| C16orf89 | 1.150931647 | 0.000164287 | 0.000613137 | upregulated |
| PSMB10 | 1.149751293 | 2.36E-40 | 1.16E-38 | upregulated |
| TSPOAP1-AS1 | 1.149355175 | 1.66E-21 | 3.65E-20 | upregulated |
| ACKR1 | 1.1484167 | 1.71E-13 | 2.49E-12 | upregulated |
| TNFRSF14 | 1.147959117 | 4.23E-38 | 1.89E-36 | upregulated |
| RIPOR3 | 1.147875594 | 9.30E-11 | 1.03E-09 | upregulated |
| SLC15A3 | 1.146536388 | 4.49E-42 | 2.43E-40 | upregulated |
| CDH23 | 1.145443806 | 1.87E-09 | 1.75E-08 | upregulated |
| CHRDL1 | 1.145000359 | 1.55E-11 | 1.87E-10 | upregulated |
| LINC02416 | 1.141626319 | 4.07E-24 | 9.94E-23 | upregulated |
| MIA | 1.141193102 | 0.002332485 | 0.006273745 | upregulated |
| RLN2 | 1.140577911 | 2.79E-07 | 1.89E-06 | upregulated |
| IGSF21 | 1.140321945 | 3.59E-21 | 7.79E-20 | upregulated |
| ARHGDIB | 1.139882536 | 1.82E-51 | 1.87E-49 | upregulated |
| UCP2 | 1.139196825 | 1.18E-32 | 4.07E-31 | upregulated |
| XXYLT1-AS2 | 1.138767662 | 1.14E-24 | 2.86E-23 | upregulated |
| MMP25 | 1.137816234 | 5.52E-35 | 2.13E-33 | upregulated |
| ENPP3 | 1.13709036 | 2.75E-10 | 2.88E-09 | upregulated |
| IFI30 | 1.136926663 | 3.03E-37 | 1.30E-35 | upregulated |
| IGLV3-27 | 1.134246769 | 1.71E-10 | 1.85E-09 | upregulated |
| ESR1 | 1.132533632 | 3.07E-11 | 3.61E-10 | upregulated |
| AC008760.2 | 1.131161086 | 1.99E-11 | 2.38E-10 | upregulated |
| GYPC | 1.129554008 | 3.31E-31 | 1.06E-29 | upregulated |
| SFRP4 | 1.128838239 | 2.19E-06 | 1.25E-05 | upregulated |
| SIRPB2 | 1.128679175 | 2.97E-31 | 9.49E-30 | upregulated |
| SPNS3 | 1.127911474 | 8.39E-28 | 2.33E-26 | upregulated |
| CTSG | 1.126395401 | 1.48E-08 | 1.22E-07 | upregulated |
| LAP3 | 1.126242064 | 3.82E-35 | 1.48E-33 | upregulated |
| LPXN | 1.12520963 | 1.22E-40 | 6.08E-39 | upregulated |
| IGKV2D-30 | 1.123306216 | 1.53E-10 | 1.66E-09 | upregulated |
| AC116366.1 | 1.121797624 | 2.28E-21 | 5.00E-20 | upregulated |
| MAN1C1 | 1.121029446 | 5.20E-27 | 1.39E-25 | upregulated |
| TACR1 | 1.117088684 | 9.19E-08 | 6.73E-07 | upregulated |
| MGAT4A | 1.115688802 | 9.52E-34 | 3.47E-32 | upregulated |
| LINC00926 | 1.114999893 | 1.14E-08 | 9.57E-08 | upregulated |
| ABCA8 | 1.111100459 | 5.01E-06 | 2.67E-05 | upregulated |
| CD1A | 1.11007549 | 8.98E-12 | 1.11E-10 | upregulated |
| APOL6 | 1.109927454 | 2.77E-33 | 9.81E-32 | upregulated |
| LINC02091 | 1.106758062 | 0.00036641 | 0.001242352 | upregulated |
| MS4A2 | 1.106750548 | 9.62E-12 | 1.18E-10 | upregulated |
| AC022509.2 | 1.105387197 | 7.13E-08 | 5.31E-07 | upregulated |
| LINC01638 | 1.105239217 | 2.58E-05 | 0.000117586 | upregulated |
| ARHGAP25 | 1.10364225 | 5.82E-44 | 3.58E-42 | upregulated |
| CYSLTR2 | 1.103222387 | 2.36E-31 | 7.60E-30 | upregulated |
| NLRC4 | 1.101098098 | 5.69E-47 | 4.24E-45 | upregulated |
| OTOF | 1.097859063 | 2.40E-22 | 5.45E-21 | upregulated |
| TVP23A | 1.097107172 | 7.05E-35 | 2.71E-33 | upregulated |
| IGHV3OR16-6 | 1.096929449 | 5.79E-10 | 5.82E-09 | upregulated |
| AC145098.1 | 1.096832503 | 2.54E-26 | 6.66E-25 | upregulated |
| ZDHHC20P1 | 1.096162164 | 1.35E-07 | 9.59E-07 | upregulated |
| HLA-F | 1.09190269 | 1.71E-29 | 5.09E-28 | upregulated |
| MIR4645 | 1.090841784 | 2.14E-08 | 1.72E-07 | upregulated |
| ZMYND15 | 1.09051635 | 1.39E-38 | 6.30E-37 | upregulated |
| C3 | 1.089451297 | 6.55E-18 | 1.25E-16 | upregulated |
| HAPLN3 | 1.089416561 | 4.88E-38 | 2.17E-36 | upregulated |
| ERAP2 | 1.085781136 | 5.25E-10 | 5.30E-09 | upregulated |
| CCDC69 | 1.085152169 | 5.19E-36 | 2.09E-34 | upregulated |
| SMIM25 | 1.084932657 | 5.16E-24 | 1.26E-22 | upregulated |
| JSRP1 | 1.084210182 | 1.34E-11 | 1.63E-10 | upregulated |
| NXPE4 | 1.081416191 | 0.000369717 | 0.001251415 | upregulated |
| KMO | 1.079819242 | 1.16E-34 | 4.37E-33 | upregulated |
| AC244669.2 | 1.079563118 | 1.98E-05 | 9.32E-05 | upregulated |
| FAM107A | 1.079278328 | 8.26E-13 | 1.14E-11 | upregulated |
| TMEM229B | 1.078922405 | 8.56E-24 | 2.06E-22 | upregulated |
| ACSL5 | 1.078899887 | 1.03E-28 | 2.99E-27 | upregulated |
| AL391056.1 | 1.076815859 | 7.01E-07 | 4.39E-06 | upregulated |
| SYNPO2 | 1.076667946 | 3.07E-06 | 1.70E-05 | upregulated |
| PAX5 | 1.07409351 | 1.37E-07 | 9.75E-07 | upregulated |
| IL15 | 1.073053721 | 1.08E-26 | 2.87E-25 | upregulated |
| CCL3L1 | 1.072927194 | 1.67E-16 | 2.95E-15 | upregulated |
| IFNK | 1.070498911 | 0.017186185 | 0.034377602 | upregulated |
| SEC22B4P | 1.068135783 | 1.47E-11 | 1.78E-10 | upregulated |
| NR1H3 | 1.067356366 | 1.87E-30 | 5.76E-29 | upregulated |
| TRANK1 | 1.064786926 | 5.57E-32 | 1.85E-30 | upregulated |
| SNAI3 | 1.064625386 | 4.02E-36 | 1.63E-34 | upregulated |
| CHI3L2 | 1.0645034 | 1.29E-20 | 2.76E-19 | upregulated |
| ATP8A1 | 1.063432503 | 8.07E-23 | 1.87E-21 | upregulated |
| SYNE1 | 1.05992269 | 2.88E-18 | 5.57E-17 | upregulated |
| F13A1 | 1.058599739 | 2.96E-10 | 3.09E-09 | upregulated |
| AL161669.1 | 1.056931879 | 5.17E-09 | 4.56E-08 | upregulated |
| SERPINB9P1 | 1.055866109 | 2.57E-16 | 4.47E-15 | upregulated |
| AC243960.3 | 1.055537284 | 3.72E-18 | 7.14E-17 | upregulated |
| IGHD | 1.054555515 | 9.47E-10 | 9.22E-09 | upregulated |
| SLC14A1 | 1.054048357 | 9.70E-19 | 1.91E-17 | upregulated |
| PATL2 | 1.053331045 | 3.46E-23 | 8.16E-22 | upregulated |
| AC010247.2 | 1.05097008 | 7.35E-24 | 1.78E-22 | upregulated |
| CCL22 | 1.050770176 | 2.82E-21 | 6.13E-20 | upregulated |
| AL731567.1 | 1.049679806 | 2.39E-14 | 3.72E-13 | upregulated |
| ADGRE1 | 1.049279817 | 4.32E-19 | 8.66E-18 | upregulated |
| FMO1 | 1.04594944 | 3.48E-08 | 2.72E-07 | upregulated |
| CYP4X1 | 1.045865928 | 0.000271135 | 0.000953421 | upregulated |
| HLA-DOB | 1.041612563 | 1.44E-21 | 3.17E-20 | upregulated |
| AC147651.3 | 1.040987737 | 1.83E-35 | 7.21E-34 | upregulated |
| COL23A1 | 1.040105304 | 9.19E-09 | 7.80E-08 | upregulated |
| BIRC3 | 1.039774404 | 9.23E-16 | 1.57E-14 | upregulated |
| ATOH8 | 1.03905601 | 1.37E-11 | 1.67E-10 | upregulated |
| ICAM2 | 1.037500585 | 3.70E-23 | 8.69E-22 | upregulated |
| TLR1 | 1.036745244 | 4.15E-26 | 1.07E-24 | upregulated |
| TLR3 | 1.035370381 | 9.23E-23 | 2.13E-21 | upregulated |
| VWA3B | 1.034926507 | 7.99E-12 | 9.95E-11 | upregulated |
| GBP1P1 | 1.03486947 | 7.32E-20 | 1.50E-18 | upregulated |
| AC093278.2 | 1.03474785 | 3.51E-29 | 1.03E-27 | upregulated |
| ATP2A3 | 1.034159844 | 1.46E-26 | 3.85E-25 | upregulated |
| PRKAR2B | 1.031293673 | 3.78E-21 | 8.20E-20 | upregulated |
| CCL14 | 1.028864914 | 8.43E-10 | 8.25E-09 | upregulated |
| ACKR4 | 1.028310874 | 5.82E-09 | 5.09E-08 | upregulated |
| ACY3 | 1.028308538 | 2.98E-18 | 5.76E-17 | upregulated |
| BANK1 | 1.027765216 | 3.12E-06 | 1.73E-05 | upregulated |
| AL645939.1 | 1.027072857 | 1.34E-12 | 1.80E-11 | upregulated |
| CLEC5A | 1.026079567 | 4.35E-23 | 1.01E-21 | upregulated |
| TNXB | 1.025867707 | 3.29E-07 | 2.19E-06 | upregulated |
| RSPO3 | 1.025797999 | 1.86E-15 | 3.08E-14 | upregulated |
| TAP1 | 1.02533281 | 2.09E-27 | 5.68E-26 | upregulated |
| ART3 | 1.024911328 | 1.48E-18 | 2.89E-17 | upregulated |
| ADAMTS16 | 1.024809008 | 0.003307572 | 0.008483239 | upregulated |
| AL683807.1 | 1.024117135 | 1.55E-15 | 2.59E-14 | upregulated |
| AC245128.3 | 1.023611795 | 2.64E-06 | 1.48E-05 | upregulated |
| ABCB1 | 1.023593661 | 2.20E-21 | 4.84E-20 | upregulated |
| RNU4-62P | 1.022658639 | 4.82E-13 | 6.74E-12 | upregulated |
| RHEX | 1.021803666 | 3.12E-12 | 4.05E-11 | upregulated |
| GNG2 | 1.021329017 | 5.37E-32 | 1.79E-30 | upregulated |
| CYFIP2 | 1.019716313 | 4.64E-31 | 1.47E-29 | upregulated |
| PSTPIP1 | 1.019701379 | 4.05E-33 | 1.43E-31 | upregulated |
| CERKL | 1.019433326 | 1.44E-36 | 6.02E-35 | upregulated |
| LINC02084 | 1.017904824 | 8.04E-16 | 1.37E-14 | upregulated |
| SARDH | 1.016636224 | 6.94E-34 | 2.55E-32 | upregulated |
| AL157394.1 | 1.016224794 | 1.99E-24 | 4.95E-23 | upregulated |
| EPYC | 1.014534902 | 7.34E-07 | 4.58E-06 | upregulated |
| MYCN | 1.01369814 | 4.69E-08 | 3.60E-07 | upregulated |
| GABRP | 1.013430044 | 5.09E-07 | 3.27E-06 | upregulated |
| INSL3 | 1.013325031 | 4.69E-15 | 7.55E-14 | upregulated |
| IFIT2 | 1.013177381 | 2.09E-20 | 4.43E-19 | upregulated |
| CLEC17A | 1.013167247 | 1.09E-10 | 1.19E-09 | upregulated |
| RERGL | 1.008951166 | 0.000447251 | 0.001476827 | upregulated |
| HP | 1.00760936 | 0.025229576 | 0.047366727 | upregulated |
| RNASE1 | 1.007504139 | 3.39E-27 | 9.13E-26 | upregulated |
| FCER1A | 1.007245842 | 1.74E-07 | 1.21E-06 | upregulated |
| IFI35 | 1.007079311 | 1.69E-23 | 4.01E-22 | upregulated |
| AKAP14 | 1.006557512 | 0.000147278 | 0.000555324 | upregulated |
| AC023449.2 | 1.005730118 | 8.29E-24 | 2.00E-22 | upregulated |
| SV2B | 1.005475325 | 8.35E-12 | 1.04E-10 | upregulated |
| NHSL2 | 1.001081997 | 5.06E-07 | 3.26E-06 | upregulated |
| GALM | 1.000931392 | 2.71E-34 | 1.01E-32 | upregulated |
| ENTPD8 | -1.001815108 | 1.49E-05 | 7.22E-05 | downregulated |
| SRMS | -1.00360566 | 6.71E-06 | 3.50E-05 | downregulated |
| KLF14 | -1.003697865 | 0.000465514 | 0.001530721 | downregulated |
| VPS37D | -1.004025474 | 2.49E-06 | 1.40E-05 | downregulated |
| AC092903.1 | -1.005867766 | 0.005850362 | 0.013811759 | downregulated |
| CDH16 | -1.007604566 | 0.000824196 | 0.002536538 | downregulated |
| PNLDC1 | -1.013368392 | 0.013230637 | 0.027640477 | downregulated |
| AC008443.2 | -1.013807309 | 0.000138476 | 0.000525158 | downregulated |
| PIANP | -1.014137098 | 0.006243998 | 0.014594355 | downregulated |
| AC104687.2 | -1.014751426 | 0.001176636 | 0.003452668 | downregulated |
| PURPL | -1.015741175 | 0.013592642 | 0.028273899 | downregulated |
| FAXC | -1.016469935 | 4.17E-08 | 3.21E-07 | downregulated |
| VWA8-AS1 | -1.017546908 | 3.85E-11 | 4.47E-10 | downregulated |
| AC012358.3 | -1.018082408 | 2.39E-07 | 1.64E-06 | downregulated |
| TMEM88B | -1.019115481 | 5.63E-07 | 3.58E-06 | downregulated |
| VWA5B2 | -1.019290642 | 0.006852632 | 0.015814689 | downregulated |
| AC079949.2 | -1.022303823 | 9.42E-06 | 4.75E-05 | downregulated |
| C10orf91 | -1.024519902 | 1.19E-10 | 1.30E-09 | downregulated |
| LINC01518 | -1.028779092 | 0.00026136 | 0.000923002 | downregulated |
| STEAP3-AS1 | -1.029396722 | 3.79E-05 | 0.000165952 | downregulated |
| AC007098.1 | -1.030423539 | 3.71E-13 | 5.25E-12 | downregulated |
| DKK4 | -1.033197135 | 0.017643356 | 0.035128116 | downregulated |
| AL589986.2 | -1.036400221 | 4.04E-09 | 3.60E-08 | downregulated |
| PQLC2L | -1.037741308 | 0.013101651 | 0.027400048 | downregulated |
| P3H2-AS1 | -1.039145105 | 5.59E-05 | 0.000234879 | downregulated |
| AC108174.1 | -1.040552895 | 0.001266382 | 0.003674988 | downregulated |
| AC012512.1 | -1.040693498 | 9.19E-05 | 0.000366037 | downregulated |
| SOX2-OT | -1.043195053 | 0.001079971 | 0.003206244 | downregulated |
| AL122058.1 | -1.044719625 | 0.010693854 | 0.02309798 | downregulated |
| FAM184B | -1.045872326 | 0.001282361 | 0.003715887 | downregulated |
| SCN4B | -1.047343493 | 0.014100548 | 0.029151977 | downregulated |
| AL121790.2 | -1.051078132 | 0.018014988 | 0.03574529 | downregulated |
| C1GALT1C1L | -1.053031947 | 7.25E-08 | 5.39E-07 | downregulated |
| H2BFWT | -1.054630358 | 1.48E-06 | 8.72E-06 | downregulated |
| CYP4F11 | -1.054643925 | 2.96E-08 | 2.34E-07 | downregulated |
| AL353693.1 | -1.055920206 | 0.019450064 | 0.038126708 | downregulated |
| LINC01234 | -1.057242822 | 4.62E-09 | 4.10E-08 | downregulated |
| SNCB | -1.057977178 | 0.000110472 | 0.000430212 | downregulated |
| AC131532.1 | -1.059898489 | 9.45E-07 | 5.79E-06 | downregulated |
| PPP1R1C | -1.060690197 | 3.43E-12 | 4.43E-11 | downregulated |
| PNPLA3 | -1.060994822 | 5.88E-15 | 9.42E-14 | downregulated |
| AC008443.6 | -1.063892464 | 1.47E-05 | 7.12E-05 | downregulated |
| AC093904.2 | -1.066156916 | 8.26E-10 | 8.10E-09 | downregulated |
| YWHAEP1 | -1.068421967 | 0.006075928 | 0.014265693 | downregulated |
| AC006213.3 | -1.070082098 | 4.55E-06 | 2.44E-05 | downregulated |
| AL512324.1 | -1.070222242 | 0.005677135 | 0.013443047 | downregulated |
| RIMKLA | -1.07250746 | 0.00119558 | 0.003500349 | downregulated |
| FSIP2-AS1 | -1.073472325 | 0.000933091 | 0.002819439 | downregulated |
| LINC01995 | -1.073514308 | 2.02E-05 | 9.45E-05 | downregulated |
| EGFR-AS1 | -1.077858127 | 1.45E-06 | 8.57E-06 | downregulated |
| MAGEA3 | -1.078461674 | 5.68E-05 | 0.000237947 | downregulated |
| G2E3-AS1 | -1.080768931 | 7.93E-10 | 7.79E-09 | downregulated |
| MSI1 | -1.082118888 | 0.000279018 | 0.000977306 | downregulated |
| ZDHHC11B | -1.083156858 | 0.001283731 | 0.003719311 | downregulated |
| SMIM24 | -1.083375276 | 0.000107938 | 0.000421842 | downregulated |
| KCNG3 | -1.085498359 | 0.000138794 | 0.000526163 | downregulated |
| REEP1 | -1.085974089 | 4.88E-06 | 2.60E-05 | downregulated |
| AL445250.1 | -1.087082726 | 0.025566836 | 0.047867702 | downregulated |
| CRLF1 | -1.087288693 | 1.64E-05 | 7.86E-05 | downregulated |
| VAX2 | -1.0903793 | 1.51E-09 | 1.43E-08 | downregulated |
| UPK1A | -1.091465185 | 0.001482515 | 0.00422141 | downregulated |
| AC117386.2 | -1.09354515 | 0.002343676 | 0.00629869 | downregulated |
| AC089987.2 | -1.093627664 | 0.000103969 | 0.000408355 | downregulated |
| BRD9P2 | -1.093735163 | 0.010163693 | 0.022132702 | downregulated |
| PRPH | -1.095076996 | 1.08E-05 | 5.39E-05 | downregulated |
| AC105460.2 | -1.097941258 | 3.53E-09 | 3.17E-08 | downregulated |
| SPRR4 | -1.09854559 | 0.002220722 | 0.006017174 | downregulated |
| MRAP2 | -1.099685909 | 0.000762531 | 0.002364975 | downregulated |
| PPP1R1B | -1.102771019 | 0.00122863 | 0.00358602 | downregulated |
| PALM3 | -1.105620205 | 0.010903149 | 0.023487462 | downregulated |
| HOXA13 | -1.106811208 | 0.004490063 | 0.011018626 | downregulated |
| DUSP9 | -1.107152568 | 4.70E-13 | 6.59E-12 | downregulated |
| GDPD2 | -1.109138778 | 4.01E-08 | 3.10E-07 | downregulated |
| SLC13A4 | -1.111519561 | 0.003840358 | 0.009645174 | downregulated |
| ABCC2 | -1.112755599 | 1.72E-06 | 9.98E-06 | downregulated |
| AL390334.1 | -1.113976751 | 4.82E-08 | 3.69E-07 | downregulated |
| AKR1C7P | -1.115796212 | 3.28E-06 | 1.80E-05 | downregulated |
| NPR3 | -1.117829519 | 0.006279145 | 0.014664233 | downregulated |
| BCHE | -1.119191207 | 1.00E-06 | 6.11E-06 | downregulated |
| AC006262.2 | -1.120592932 | 0.000772922 | 0.002394942 | downregulated |
| ODC1 | -1.123561985 | 9.37E-10 | 9.13E-09 | downregulated |
| AC093904.4 | -1.124058859 | 7.26E-08 | 5.40E-07 | downregulated |
| FMO9P | -1.124476908 | 0.002132851 | 0.005809712 | downregulated |
| AC005336.1 | -1.126969127 | 7.03E-08 | 5.24E-07 | downregulated |
| LINC01564 | -1.129180435 | 4.13E-07 | 2.70E-06 | downregulated |
| GTF2IP7 | -1.131518997 | 0.000129725 | 0.000495355 | downregulated |
| LINC01088 | -1.131711373 | 0.014051975 | 0.029064525 | downregulated |
| EVPLL | -1.133648818 | 1.43E-06 | 8.45E-06 | downregulated |
| PLA2G4A | -1.135627948 | 1.38E-08 | 1.15E-07 | downregulated |
| DNAH2 | -1.136367374 | 1.12E-06 | 6.75E-06 | downregulated |
| LINC00518 | -1.13913389 | 0.000169456 | 0.000630518 | downregulated |
| GPX2 | -1.139156847 | 1.77E-11 | 2.13E-10 | downregulated |
| LINC00659 | -1.14043855 | 0.000117548 | 0.000454624 | downregulated |
| AC005722.3 | -1.141393412 | 9.86E-05 | 0.000389328 | downregulated |
| AL596223.1 | -1.141538425 | 9.72E-05 | 0.000384398 | downregulated |
| CCDC151 | -1.143948564 | 0.007071599 | 0.016258739 | downregulated |
| RPL17P43 | -1.144222317 | 0.000890214 | 0.002707312 | downregulated |
| AP005119.2 | -1.144881129 | 0.000114571 | 0.000443894 | downregulated |
| MSLN | -1.146094436 | 2.28E-05 | 0.000105277 | downregulated |
| FOXI3 | -1.14660211 | 0.012603916 | 0.026536682 | downregulated |
| PRSS50 | -1.147280728 | 0.008071005 | 0.018185361 | downregulated |
| SAMD12-AS1 | -1.147528906 | 0.000111613 | 0.000433884 | downregulated |
| RAB3B | -1.148581253 | 1.02E-05 | 5.11E-05 | downregulated |
| GCGR | -1.150236131 | 0.00187906 | 0.005190852 | downregulated |
| GOLGA6L7 | -1.150323319 | 0.00480881 | 0.011681586 | downregulated |
| AC122685.1 | -1.156777847 | 0.000137626 | 0.000522136 | downregulated |
| AC003092.1 | -1.157010634 | 0.013580712 | 0.028255048 | downregulated |
| CASKIN1 | -1.157534304 | 2.31E-11 | 2.74E-10 | downregulated |
| RPL23AP11 | -1.157990102 | 7.15E-08 | 5.33E-07 | downregulated |
| VGLL1 | -1.158100622 | 0.001589395 | 0.004487503 | downregulated |
| RNU6-653P | -1.158358432 | 5.57E-07 | 3.55E-06 | downregulated |
| UGT1A6 | -1.159145953 | 1.04E-06 | 6.33E-06 | downregulated |
| LINC02310 | -1.160888404 | 6.15E-09 | 5.35E-08 | downregulated |
| CLDN8 | -1.160981673 | 0.003116958 | 0.008060422 | downregulated |
| GSTM2 | -1.162268005 | 0.001874422 | 0.005180217 | downregulated |
| ESPNL | -1.163266248 | 0.000543476 | 0.001755169 | downregulated |
| TMEM151A | -1.164282993 | 1.98E-05 | 9.28E-05 | downregulated |
| POU6F2 | -1.165576291 | 7.93E-06 | 4.07E-05 | downregulated |
| KIAA1257 | -1.170564568 | 4.95E-07 | 3.20E-06 | downregulated |
| AC124861.1 | -1.171639453 | 0.002489364 | 0.006638642 | downregulated |
| AC083801.2 | -1.171856054 | 2.09E-07 | 1.44E-06 | downregulated |
| AC004990.1 | -1.173566513 | 9.42E-07 | 5.77E-06 | downregulated |
| TGFBR3L | -1.174460011 | 2.74E-09 | 2.51E-08 | downregulated |
| CHP2 | -1.174526768 | 2.64E-07 | 1.79E-06 | downregulated |
| EFNA2 | -1.177612438 | 3.00E-05 | 0.000135004 | downregulated |
| EPCAM | -1.179895764 | 5.46E-09 | 4.79E-08 | downregulated |
| KRT71 | -1.182684953 | 0.000224959 | 0.000806864 | downregulated |
| LINC02315 | -1.185129066 | 0.000136584 | 0.000518882 | downregulated |
| SLC13A5 | -1.186345653 | 6.79E-06 | 3.53E-05 | downregulated |
| NPW | -1.188404763 | 0.000302179 | 0.001046349 | downregulated |
| OTOP2 | -1.188937024 | 3.22E-08 | 2.53E-07 | downregulated |
| LINC02253 | -1.189820097 | 5.61E-05 | 0.000235493 | downregulated |
| PTGS2 | -1.191815581 | 1.02E-05 | 5.12E-05 | downregulated |
| RN7SL399P | -1.192901094 | 3.39E-05 | 0.000150547 | downregulated |
| HBE1 | -1.198402029 | 0.001286876 | 0.003727327 | downregulated |
| GSTA9P | -1.200243376 | 1.54E-07 | 1.09E-06 | downregulated |
| AC005077.2 | -1.202226928 | 4.89E-05 | 0.000208447 | downregulated |
| AC005863.1 | -1.202910872 | 0.015426618 | 0.031428785 | downregulated |
| FGF13 | -1.203461838 | 0.001292029 | 0.003737858 | downregulated |
| ATP13A5 | -1.205668059 | 0.000106884 | 0.000418307 | downregulated |
| EFCAB10 | -1.207741464 | 2.37E-09 | 2.18E-08 | downregulated |
| AC025244.1 | -1.208936234 | 2.47E-07 | 1.68E-06 | downregulated |
| GLI2 | -1.216077503 | 4.83E-10 | 4.91E-09 | downregulated |
| SULT4A1 | -1.216159493 | 7.93E-06 | 4.07E-05 | downregulated |
| DLX2 | -1.216282588 | 2.26E-09 | 2.10E-08 | downregulated |
| TUBB8P7 | -1.217270679 | 0.001077269 | 0.003199669 | downregulated |
| RCOR2 | -1.220190625 | 1.28E-12 | 1.72E-11 | downregulated |
| NMRAL2P | -1.227327241 | 7.88E-11 | 8.82E-10 | downregulated |
| LINC02570 | -1.229978022 | 1.57E-08 | 1.29E-07 | downregulated |
| LINC02031 | -1.231341653 | 8.41E-05 | 0.000338445 | downregulated |
| AC008440.3 | -1.234168744 | 2.27E-07 | 1.56E-06 | downregulated |
| AC006357.1 | -1.238792513 | 0.000331906 | 0.001137888 | downregulated |
| PCNPP3 | -1.241488314 | 3.61E-05 | 0.000158908 | downregulated |
| FAR2P4 | -1.243063287 | 0.000131959 | 0.000503058 | downregulated |
| GPR149 | -1.24353867 | 0.000703057 | 0.002199549 | downregulated |
| IGF2BP1 | -1.244639612 | 0.002250707 | 0.006082833 | downregulated |
| FOXA3 | -1.25014382 | 0.001160553 | 0.003413096 | downregulated |
| LINC00668 | -1.250292213 | 3.35E-06 | 1.84E-05 | downregulated |
| AC105460.1 | -1.252515643 | 1.57E-08 | 1.29E-07 | downregulated |
| UGT1A7 | -1.254173034 | 0.002434861 | 0.006507193 | downregulated |
| EMILIN3 | -1.255342089 | 5.30E-06 | 2.80E-05 | downregulated |
| FOLR3 | -1.255593155 | 0.022719076 | 0.043364595 | downregulated |
| AKR1C3 | -1.262455054 | 3.41E-08 | 2.66E-07 | downregulated |
| MIR3164 | -1.271853902 | 8.31E-05 | 0.000334577 | downregulated |
| AL645608.3 | -1.280182459 | 2.65E-07 | 1.79E-06 | downregulated |
| KRTAP19-1 | -1.28322844 | 4.67E-06 | 2.50E-05 | downregulated |
| CHCHD2P4 | -1.290079655 | 0.00234125 | 0.006294743 | downregulated |
| CEL | -1.293814488 | 0.001775395 | 0.004943271 | downregulated |
| ACSBG1 | -1.301729783 | 0.000371447 | 0.001257055 | downregulated |
| SLC25A48 | -1.308809592 | 0.000927563 | 0.002804886 | downregulated |
| KRT9 | -1.309367925 | 2.28E-09 | 2.11E-08 | downregulated |
| NGB | -1.313470805 | 9.59E-06 | 4.84E-05 | downregulated |
| AP000619.1 | -1.316721011 | 0.001172723 | 0.003443748 | downregulated |
| AC092969.1 | -1.318902516 | 1.70E-09 | 1.60E-08 | downregulated |
| PRDM13 | -1.323219664 | 4.46E-05 | 0.000192111 | downregulated |
| PAK5 | -1.328300262 | 0.000729338 | 0.002275273 | downregulated |
| CYP1A1 | -1.328320492 | 0.00404859 | 0.010088466 | downregulated |
| AC005077.4 | -1.328694965 | 2.96E-06 | 1.65E-05 | downregulated |
| SNORA80B | -1.329240377 | 3.94E-08 | 3.05E-07 | downregulated |
| DEFB126 | -1.332327881 | 6.54E-09 | 5.67E-08 | downregulated |
| USH1C | -1.332407877 | 0.009201432 | 0.020355497 | downregulated |
| AC006058.3 | -1.333183646 | 4.83E-05 | 0.000206251 | downregulated |
| MTCO3P12 | -1.333409008 | 0.007866139 | 0.017794949 | downregulated |
| OTOP3 | -1.33357816 | 2.21E-07 | 1.52E-06 | downregulated |
| DLX2-DT | -1.340417186 | 9.67E-10 | 9.39E-09 | downregulated |
| LINC00871 | -1.340714656 | 1.39E-08 | 1.15E-07 | downregulated |
| TMEM139 | -1.351158176 | 0.026318675 | 0.049011111 | downregulated |
| SLC35G1 | -1.352664372 | 3.64E-14 | 5.56E-13 | downregulated |
| POU6F2-AS2 | -1.367341334 | 1.77E-09 | 1.66E-08 | downregulated |
| LINC02522 | -1.368197528 | 2.55E-05 | 0.000116507 | downregulated |
| RPL21P13 | -1.3708815 | 1.22E-05 | 6.00E-05 | downregulated |
| AC012363.1 | -1.377281901 | 2.23E-06 | 1.27E-05 | downregulated |
| PTGER1 | -1.378012132 | 0.000264924 | 0.000933581 | downregulated |
| AC128709.3 | -1.38380008 | 2.32E-09 | 2.14E-08 | downregulated |
| AC089983.1 | -1.387930044 | 1.01E-05 | 5.09E-05 | downregulated |
| AC005845.1 | -1.388442235 | 0.026836299 | 0.049776436 | downregulated |
| AC128709.1 | -1.393162773 | 4.10E-12 | 5.25E-11 | downregulated |
| OR7E158P | -1.396144053 | 0.011951657 | 0.025404682 | downregulated |
| AC133561.1 | -1.397111895 | 4.83E-05 | 0.000206316 | downregulated |
| JAKMIP3 | -1.399053391 | 3.72E-05 | 0.000163465 | downregulated |
| AF165147.1 | -1.399484757 | 8.61E-07 | 5.31E-06 | downregulated |
| SHH | -1.401446697 | 0.000393407 | 0.001320937 | downregulated |
| UGT1A10 | -1.40266851 | 3.01E-05 | 0.000135256 | downregulated |
| AC128709.2 | -1.403005628 | 1.12E-14 | 1.78E-13 | downregulated |
| NPM1P21 | -1.405885881 | 0.001904511 | 0.005255267 | downregulated |
| AKR1C1 | -1.405988139 | 9.44E-10 | 9.20E-09 | downregulated |
| AL139327.2 | -1.408537185 | 3.73E-11 | 4.34E-10 | downregulated |
| AL138881.1 | -1.411110383 | 7.69E-09 | 6.61E-08 | downregulated |
| SLC8A2 | -1.412747474 | 0.0032725 | 0.00840203 | downregulated |
| AC019080.4 | -1.415314668 | 3.64E-06 | 1.98E-05 | downregulated |
| KHDC1L | -1.420109536 | 5.91E-08 | 4.46E-07 | downregulated |
| SLC30A3 | -1.421771841 | 0.001242943 | 0.003618868 | downregulated |
| AL391427.1 | -1.422356153 | 5.06E-07 | 3.26E-06 | downregulated |
| CHL1-AS2 | -1.428444489 | 0.00309384 | 0.008007993 | downregulated |
| UPK1B | -1.430122892 | 3.64E-05 | 0.000160143 | downregulated |
| FTCD | -1.440028495 | 0.00051289 | 0.001667315 | downregulated |
| AKR1C2 | -1.440625309 | 8.53E-11 | 9.50E-10 | downregulated |
| FAR2P1 | -1.444032229 | 5.06E-07 | 3.26E-06 | downregulated |
| CASC8 | -1.444184783 | 9.82E-07 | 5.99E-06 | downregulated |
| KRT42P | -1.449724668 | 1.12E-07 | 8.09E-07 | downregulated |
| AL033397.1 | -1.450711338 | 1.17E-06 | 7.03E-06 | downregulated |
| MLXIPL | -1.462371302 | 7.43E-05 | 0.000303004 | downregulated |
| AC253536.4 | -1.466790629 | 0.022478941 | 0.042976178 | downregulated |
| AADAC | -1.467227356 | 0.000551414 | 0.00177702 | downregulated |
| AL645608.6 | -1.467676583 | 0.000901997 | 0.002737234 | downregulated |
| NTS | -1.471471416 | 0.001060535 | 0.003157101 | downregulated |
| LINC02561 | -1.482090484 | 1.61E-05 | 7.73E-05 | downregulated |
| PLPPR1 | -1.490170892 | 0.000323646 | 0.001113249 | downregulated |
| RGL3 | -1.492403285 | 0.023978869 | 0.045364302 | downregulated |
| HKDC1 | -1.501341619 | 6.83E-07 | 4.30E-06 | downregulated |
| CALB1 | -1.502918042 | 9.37E-07 | 5.74E-06 | downregulated |
| AC007249.1 | -1.523443921 | 0.000214565 | 0.000774392 | downregulated |
| DMRT1 | -1.542360837 | 3.07E-06 | 1.70E-05 | downregulated |
| FREM2 | -1.556246978 | 0.02237302 | 0.042819354 | downregulated |
| TPSP2 | -1.567346818 | 0.000514632 | 0.001671325 | downregulated |
| HOXB-AS3 | -1.567633466 | 0.000394752 | 0.001324777 | downregulated |
| SDAD1P2 | -1.573910838 | 2.71E-08 | 2.15E-07 | downregulated |
| NYAP1 | -1.579718074 | 6.87E-11 | 7.72E-10 | downregulated |
| AADACL2-AS1 | -1.582716877 | 0.009303279 | 0.020532415 | downregulated |
| CALHM3 | -1.589498322 | 0.009772413 | 0.021381086 | downregulated |
| C11orf87 | -1.591822662 | 0.009878896 | 0.021580526 | downregulated |
| KLF2P1 | -1.594514786 | 1.06E-05 | 5.29E-05 | downregulated |
| AP001547.1 | -1.615123864 | 4.50E-05 | 0.000193257 | downregulated |
| CYP4F2 | -1.61745491 | 0.001770943 | 0.004934359 | downregulated |
| SLC6A10P | -1.626907168 | 0.000749211 | 0.002328059 | downregulated |
| GSTA3 | -1.635691395 | 1.34E-08 | 1.12E-07 | downregulated |
| CLEC2L | -1.64787805 | 4.28E-05 | 0.000185281 | downregulated |
| LINC00556 | -1.650090548 | 0.000324819 | 0.001117091 | downregulated |
| SLC9A4 | -1.685013958 | 0.004260081 | 0.010536884 | downregulated |
| AC120498.4 | -1.686246529 | 5.93E-06 | 3.12E-05 | downregulated |
| CPA2 | -1.705777889 | 0.012174953 | 0.025773484 | downregulated |
| BTBD16 | -1.713163862 | 2.01E-10 | 2.14E-09 | downregulated |
| MAGEA12 | -1.719871276 | 0.002415946 | 0.006462082 | downregulated |
| TBX4 | -1.721841589 | 0.000114926 | 0.000445184 | downregulated |
| SYNGR4 | -1.726098651 | 0.000178366 | 0.000658699 | downregulated |
| NUPR2 | -1.73976824 | 7.21E-07 | 4.50E-06 | downregulated |
| MAGEA8 | -1.741059139 | 0.017652958 | 0.035140135 | downregulated |
| GALR2 | -1.746174393 | 0.00073248 | 0.002283267 | downregulated |
| AC026471.3 | -1.762512574 | 1.20E-06 | 7.24E-06 | downregulated |
| FOXH1 | -1.776956698 | 1.29E-05 | 6.33E-05 | downregulated |
| AC012501.2 | -1.791564211 | 0.000118294 | 0.000456614 | downregulated |
| JPH3 | -1.795012271 | 0.004040349 | 0.010077107 | downregulated |
| AL355796.1 | -1.795914464 | 3.24E-07 | 2.16E-06 | downregulated |
| AC073365.1 | -1.815419993 | 1.52E-08 | 1.25E-07 | downregulated |
| LINC01694 | -1.819451446 | 0.000126212 | 0.000483955 | downregulated |
| MPPED1 | -1.828133634 | 0.00022898 | 0.000819663 | downregulated |
| CYP2W1 | -1.847186918 | 1.22E-08 | 1.02E-07 | downregulated |
| SYT13 | -1.857203651 | 0.008290085 | 0.018593976 | downregulated |
| FGF19 | -1.864662518 | 8.35E-06 | 4.26E-05 | downregulated |
| CYP2AB1P | -1.867295599 | 6.64E-08 | 4.96E-07 | downregulated |
| LINC00648 | -1.868315021 | 0.000671453 | 0.002114435 | downregulated |
| C10orf82 | -1.875401102 | 9.15E-06 | 4.63E-05 | downregulated |
| SMCP | -1.886042219 | 1.84E-10 | 1.98E-09 | downregulated |
| RNF183 | -1.888815495 | 0.01743484 | 0.034765629 | downregulated |
| CLDN20 | -1.889957753 | 3.34E-06 | 1.83E-05 | downregulated |
| AC009502.2 | -1.918838853 | 1.21E-07 | 8.63E-07 | downregulated |
| LHX9 | -1.920264871 | 0.002807894 | 0.007357705 | downregulated |
| SPTLC1P4 | -1.92549634 | 2.45E-07 | 1.67E-06 | downregulated |
| SELENOOLP | -1.93189132 | 6.94E-05 | 0.000285014 | downregulated |
| RIPPLY2 | -1.974958922 | 3.98E-08 | 3.08E-07 | downregulated |
| LY6G6D | -1.990325253 | 0.000431034 | 0.001429985 | downregulated |
| BOK-AS1 | -2.065570751 | 0.026709837 | 0.049593201 | downregulated |
| STRA8 | -2.08849716 | 0.009842892 | 0.021520956 | downregulated |
| NR5A1 | -2.088756505 | 0.003588852 | 0.009100468 | downregulated |
| SPDYC | -2.102553891 | 8.42E-05 | 0.000338466 | downregulated |
| LINC00393 | -2.103353958 | 1.64E-07 | 1.15E-06 | downregulated |
| PDIA2 | -2.12922346 | 4.26E-06 | 2.30E-05 | downregulated |
| AC139769.2 | -2.129726032 | 0.005020119 | 0.012114288 | downregulated |
| AC087491.1 | -2.184693021 | 2.57E-05 | 0.000117234 | downregulated |
| LINC02582 | -2.218392597 | 1.21E-06 | 7.24E-06 | downregulated |
| AC087783.2 | -2.32472532 | 4.88E-07 | 3.15E-06 | downregulated |
| ARHGDIG | -2.336020408 | 0.005617481 | 0.013324688 | downregulated |
| BRDT | -2.407653149 | 0.017862598 | 0.035492933 | downregulated |
| AC073347.1 | -2.447167424 | 2.76E-06 | 1.54E-05 | downregulated |
| OR51B5 | -2.461193919 | 0.003362504 | 0.008603977 | downregulated |
| LINC00392 | -2.520690163 | 3.27E-05 | 0.000145661 | downregulated |
| FGFBP2 | -2.520873019 | 0.011214922 | 0.024088693 | downregulated |
| DDC | -2.540569964 | 0.007470431 | 0.017032686 | downregulated |
| HBQ1 | -2.557939922 | 3.49E-05 | 0.000154198 | downregulated |
| C11orf86 | -2.652056681 | 0.011080195 | 0.023837595 | downregulated |
| TRBV20OR9-2 | -2.908493694 | 6.98E-07 | 4.37E-06 | downregulated |
| LINC00974 | -3.143177185 | 0.000340711 | 0.001164027 | downregulated |
| KRT20 | -4.153574749 | 0.000277561 | 0.000973069 | downregulated |
