# Supplementary material for: Effective TME-related signature to predict prognosis of patients with head and neck squamous cell carcinoma
Source: Front Mol Biosci. 2023 Aug 21;10:1232875. doi: 10.3389/fmolb.2023.1232875 (PMC10475735; doi:10.3389/fmolb.2023.1232875)
Supplement: Supplementary file 1 [file DataSheet1.zip › Supplementary Material/Supplementary Table S2.docx]

Table S2. 1,307 DEGs, including 1,191 upregulated and 116 downregulated genes, according to the median stromal score in TCGA cohort.

| gene | logFC | pValue | fdr | regulated |
| --- | --- | --- | --- | --- |
| OGN | 3.429250321 | 4.48E-39 | 3.86E-37 | upregulated |
| PLA2G2A | 3.373100477 | 1.35E-14 | 1.64E-13 | upregulated |
| SFRP4 | 3.248855121 | 4.33E-46 | 7.28E-44 | upregulated |
| EPYC | 3.166128134 | 1.91E-21 | 3.88E-20 | upregulated |
| COL11A1 | 3.112974376 | 1.05E-40 | 1.04E-38 | upregulated |
| MYL3 | 3.027507941 | 1.50E-29 | 5.57E-28 | upregulated |
| FBXO40 | 3.012079058 | 5.04E-10 | 3.98E-09 | upregulated |
| AL669970.3 | 2.98326356 | 1.07E-05 | 4.12E-05 | upregulated |
| PPP1R1A | 2.973735182 | 5.80E-12 | 5.58E-11 | upregulated |
| AGTR1 | 2.970836603 | 9.78E-48 | 1.98E-45 | upregulated |
| CILP | 2.961361824 | 1.70E-32 | 8.07E-31 | upregulated |
| LRRC15 | 2.899981335 | 1.88E-52 | 8.43E-50 | upregulated |
| MYH8 | 2.889278424 | 1.55E-10 | 1.29E-09 | upregulated |
| PRG4 | 2.875124352 | 5.90E-17 | 8.73E-16 | upregulated |
| DWORF | 2.848591303 | 1.08E-06 | 5.14E-06 | upregulated |
| PI16 | 2.836489526 | 9.46E-16 | 1.27E-14 | upregulated |
| OMD | 2.836485161 | 8.96E-45 | 1.42E-42 | upregulated |
| PPP1R3A | 2.835885072 | 1.43E-11 | 1.33E-10 | upregulated |
| DHRS7C | 2.819171061 | 3.37E-06 | 1.45E-05 | upregulated |
| PYGM | 2.804805586 | 6.75E-13 | 7.16E-12 | upregulated |
| MYH7 | 2.777926526 | 1.79E-09 | 1.32E-08 | upregulated |
| FNDC1 | 2.74518226 | 5.05E-51 | 1.70E-48 | upregulated |
| COL10A1 | 2.722495032 | 1.61E-42 | 2.03E-40 | upregulated |
| PPDPFL | 2.722297217 | 3.26E-06 | 1.41E-05 | upregulated |
| LINC01614 | 2.717842812 | 5.15E-42 | 5.93E-40 | upregulated |
| DNASE2B | 2.680156122 | 3.62E-18 | 5.86E-17 | upregulated |
| NPY6R | 2.678643646 | 1.39E-07 | 7.82E-07 | upregulated |
| MYLK3 | 2.658788703 | 3.70E-11 | 3.29E-10 | upregulated |
| ASPN | 2.629778661 | 9.57E-45 | 1.51E-42 | upregulated |
| ADAMTS16 | 2.625762323 | 7.02E-35 | 4.09E-33 | upregulated |
| COMP | 2.620640164 | 6.76E-33 | 3.32E-31 | upregulated |
| COL3A1 | 2.620212626 | 6.11E-60 | 1.23E-56 | upregulated |
| ANGPTL7 | 2.619891886 | 8.91E-21 | 1.73E-19 | upregulated |
| AC092484.1 | 2.608106603 | 8.70E-07 | 4.22E-06 | upregulated |
| SGCD | 2.587895344 | 1.48E-49 | 3.82E-47 | upregulated |
| ITGBL1 | 2.583158675 | 2.32E-40 | 2.25E-38 | upregulated |
| ASB11 | 2.579474202 | 1.40E-09 | 1.05E-08 | upregulated |
| THBS4 | 2.571836129 | 4.90E-29 | 1.74E-27 | upregulated |
| SFRP2 | 2.56893005 | 2.04E-56 | 1.79E-53 | upregulated |
| PTGIS | 2.561022161 | 5.22E-37 | 3.51E-35 | upregulated |
| ITGA11 | 2.555570896 | 1.41E-49 | 3.69E-47 | upregulated |
| WISP2 | 2.552430881 | 9.24E-30 | 3.50E-28 | upregulated |
| F13A1 | 2.545851611 | 8.91E-50 | 2.43E-47 | upregulated |
| POSTN | 2.537111025 | 1.03E-50 | 3.31E-48 | upregulated |
| ACTA1 | 2.529001905 | 6.02E-11 | 5.24E-10 | upregulated |
| MAGEL2 | 2.523033928 | 5.15E-47 | 9.80E-45 | upregulated |
| C10orf71 | 2.520397617 | 3.05E-12 | 3.03E-11 | upregulated |
| HJV | 2.509888076 | 4.95E-10 | 3.91E-09 | upregulated |
| CASQ1 | 2.508302037 | 4.87E-09 | 3.41E-08 | upregulated |
| MYL2 | 2.498608223 | 8.53E-10 | 6.55E-09 | upregulated |
| KLHL41 | 2.491411921 | 3.98E-10 | 3.19E-09 | upregulated |
| COL1A2 | 2.489908817 | 1.30E-57 | 1.46E-54 | upregulated |
| LINC01638 | 2.484459057 | 1.09E-32 | 5.25E-31 | upregulated |
| SMYD1 | 2.476014172 | 1.40E-11 | 1.30E-10 | upregulated |
| LMOD2 | 2.469069352 | 3.28E-09 | 2.35E-08 | upregulated |
| NRAP | 2.464800316 | 5.40E-09 | 3.75E-08 | upregulated |
| ASB10 | 2.447318832 | 2.49E-11 | 2.26E-10 | upregulated |
| COL1A1 | 2.440963327 | 2.54E-54 | 1.50E-51 | upregulated |
| STAC2 | 2.439012163 | 2.08E-09 | 1.53E-08 | upregulated |
| PODN | 2.436934368 | 4.59E-52 | 1.89E-49 | upregulated |
| CACNA1S | 2.435891646 | 6.94E-11 | 6.00E-10 | upregulated |
| XIRP2 | 2.430591219 | 2.79E-10 | 2.27E-09 | upregulated |
| LRRC17 | 2.418827548 | 3.20E-37 | 2.23E-35 | upregulated |
| ARPP21 | 2.402029695 | 7.99E-10 | 6.16E-09 | upregulated |
| VCAN | 2.39683341 | 1.12E-59 | 1.88E-56 | upregulated |
| FIBIN | 2.376412094 | 8.54E-54 | 4.79E-51 | upregulated |
| MYH2 | 2.37029054 | 2.49E-10 | 2.04E-09 | upregulated |
| C10orf71-AS1 | 2.362951557 | 5.22E-10 | 4.12E-09 | upregulated |
| CLC | 2.362316036 | 7.53E-07 | 3.71E-06 | upregulated |
| ACTN2 | 2.35714471 | 3.98E-11 | 3.53E-10 | upregulated |
| ATP1A2 | 2.355374529 | 1.46E-15 | 1.94E-14 | upregulated |
| UNC45B | 2.343320805 | 7.43E-10 | 5.74E-09 | upregulated |
| ATP1B4 | 2.340988695 | 1.34E-12 | 1.38E-11 | upregulated |
| MYOT | 2.339726526 | 8.15E-09 | 5.51E-08 | upregulated |
| ART1 | 2.339560897 | 5.92E-11 | 5.16E-10 | upregulated |
| ATP2A1 | 2.335055264 | 0.000534864 | 0.001383653 | upregulated |
| AGBL1 | 2.33014213 | 7.51E-09 | 5.10E-08 | upregulated |
| NEB | 2.329750589 | 9.76E-11 | 8.33E-10 | upregulated |
| MYOZ1 | 2.325358009 | 7.61E-11 | 6.56E-10 | upregulated |
| SCN4A | 2.323291321 | 1.45E-13 | 1.63E-12 | upregulated |
| MMP8 | 2.322359281 | 6.77E-22 | 1.42E-20 | upregulated |
| LMOD3 | 2.318020914 | 4.26E-10 | 3.39E-09 | upregulated |
| ASB15 | 2.312119653 | 1.33E-05 | 5.02E-05 | upregulated |
| RN7SL431P | 2.305145662 | 5.49E-07 | 2.78E-06 | upregulated |
| LDB3 | 2.299864536 | 1.59E-13 | 1.77E-12 | upregulated |
| CKM | 2.298565318 | 2.15E-08 | 1.37E-07 | upregulated |
| IGF2-AS | 2.298046773 | 5.94E-22 | 1.25E-20 | upregulated |
| PEBP4 | 2.297378846 | 6.74E-15 | 8.41E-14 | upregulated |
| MYPN | 2.294911535 | 2.04E-10 | 1.68E-09 | upregulated |
| LINC01929 | 2.292271508 | 6.32E-30 | 2.41E-28 | upregulated |
| CSRP3 | 2.289198801 | 6.12E-09 | 4.22E-08 | upregulated |
| LBP | 2.287854016 | 1.55E-08 | 1.01E-07 | upregulated |
| AL356417.2 | 2.287656202 | 8.57E-32 | 3.82E-30 | upregulated |
| MYH6 | 2.285199739 | 9.09E-08 | 5.26E-07 | upregulated |
| EXTL1 | 2.283642222 | 1.04E-23 | 2.50E-22 | upregulated |
| COL6A3 | 2.282692129 | 2.53E-58 | 3.41E-55 | upregulated |
| MYOZ3 | 2.282419975 | 1.97E-15 | 2.58E-14 | upregulated |
| CCKAR | 2.278737184 | 7.29E-15 | 9.07E-14 | upregulated |
| APOBEC2 | 2.275068551 | 1.51E-08 | 9.84E-08 | upregulated |
| CHRND | 2.274837088 | 5.14E-11 | 4.52E-10 | upregulated |
| CHRNG | 2.274595568 | 1.68E-08 | 1.09E-07 | upregulated |
| TRDN | 2.270698181 | 9.27E-11 | 7.93E-10 | upregulated |
| DUSP27 | 2.263358966 | 5.16E-12 | 5.01E-11 | upregulated |
| NDNF | 2.260113469 | 2.87E-44 | 4.32E-42 | upregulated |
| HHIP | 2.253586423 | 9.50E-19 | 1.61E-17 | upregulated |
| FGF10 | 2.252231176 | 3.28E-29 | 1.19E-27 | upregulated |
| MB | 2.249907652 | 2.34E-07 | 1.27E-06 | upregulated |
| COL8A1 | 2.24981237 | 1.74E-52 | 8.18E-50 | upregulated |
| FBN1 | 2.242944956 | 6.14E-56 | 4.77E-53 | upregulated |
| BNC2 | 2.242849698 | 1.66E-52 | 7.99E-50 | upregulated |
| RBFOX1 | 2.242604512 | 4.54E-10 | 3.61E-09 | upregulated |
| COX6A2 | 2.242353618 | 3.39E-07 | 1.79E-06 | upregulated |
| LINC02016 | 2.236395207 | 3.87E-09 | 2.75E-08 | upregulated |
| MIR133A1HG | 2.233004966 | 9.51E-06 | 3.72E-05 | upregulated |
| ANGPTL1 | 2.21607027 | 1.18E-31 | 5.15E-30 | upregulated |
| METTL21EP | 2.210077436 | 0.007710786 | 0.014592781 | upregulated |
| DACT1 | 2.20841122 | 4.70E-49 | 1.14E-46 | upregulated |
| DIRC1 | 2.208030748 | 1.30E-36 | 8.33E-35 | upregulated |
| TNN | 2.204718278 | 1.73E-41 | 1.89E-39 | upregulated |
| CMYA5 | 2.202967522 | 1.58E-09 | 1.18E-08 | upregulated |
| TNNC1 | 2.202767942 | 1.57E-07 | 8.75E-07 | upregulated |
| MYOZ2 | 2.201868305 | 1.38E-10 | 1.16E-09 | upregulated |
| DDIT4L | 2.201496305 | 2.81E-17 | 4.28E-16 | upregulated |
| GPC6 | 2.19528907 | 6.11E-47 | 1.13E-44 | upregulated |
| VGLL2 | 2.183598674 | 4.57E-12 | 4.47E-11 | upregulated |
| PDGFRL | 2.183401161 | 9.79E-48 | 1.98E-45 | upregulated |
| TNNI1 | 2.182638468 | 1.54E-07 | 8.58E-07 | upregulated |
| CAVIN4 | 2.181515188 | 1.28E-14 | 1.56E-13 | upregulated |
| AL499627.1 | 2.177937134 | 2.45E-08 | 1.55E-07 | upregulated |
| HSPB7 | 2.172538617 | 1.60E-17 | 2.47E-16 | upregulated |
| HHATL | 2.171634697 | 3.90E-08 | 2.39E-07 | upregulated |
| FN1 | 2.1697991 | 1.47E-48 | 3.38E-46 | upregulated |
| ADAMTS12 | 2.165641024 | 2.27E-51 | 8.04E-49 | upregulated |
| TMEM119 | 2.155875462 | 2.55E-50 | 7.76E-48 | upregulated |
| COLEC12 | 2.1554384 | 8.38E-59 | 1.30E-55 | upregulated |
| AC099786.2 | 2.154686858 | 4.19E-20 | 7.79E-19 | upregulated |
| CTNNA3 | 2.152231662 | 2.01E-16 | 2.87E-15 | upregulated |
| KLHL40 | 2.15135043 | 3.53E-10 | 2.85E-09 | upregulated |
| CHIT1 | 2.145362527 | 9.91E-12 | 9.36E-11 | upregulated |
| CHRNA1 | 2.144091026 | 1.23E-19 | 2.22E-18 | upregulated |
| C4orf54 | 2.137687847 | 7.38E-11 | 6.37E-10 | upregulated |
| PLPP4 | 2.134344388 | 7.37E-34 | 3.97E-32 | upregulated |
| MYOM1 | 2.130825188 | 8.07E-12 | 7.68E-11 | upregulated |
| MFAP4 | 2.127496459 | 1.06E-36 | 6.89E-35 | upregulated |
| CASQ2 | 2.127436653 | 1.08E-15 | 1.45E-14 | upregulated |
| AC087286.2 | 2.124928863 | 8.94E-32 | 3.97E-30 | upregulated |
| LINC01561 | 2.115320875 | 6.43E-26 | 1.79E-24 | upregulated |
| MYBPC2 | 2.11444738 | 8.64E-11 | 7.41E-10 | upregulated |
| PRUNE2 | 2.114124593 | 4.92E-25 | 1.29E-23 | upregulated |
| GALNT15 | 2.111698727 | 4.68E-48 | 9.95E-46 | upregulated |
| C1QTNF3 | 2.109153783 | 1.34E-15 | 1.79E-14 | upregulated |
| TTN | 2.107046895 | 8.00E-09 | 5.42E-08 | upregulated |
| PRR32 | 2.102825215 | 1.50E-07 | 8.35E-07 | upregulated |
| PRND | 2.098786715 | 1.29E-23 | 3.08E-22 | upregulated |
| MYF5 | 2.098639826 | 8.29E-12 | 7.88E-11 | upregulated |
| AC068506.1 | 2.095650799 | 3.15E-08 | 1.96E-07 | upregulated |
| TNFAIP6 | 2.094841315 | 1.01E-51 | 3.91E-49 | upregulated |
| FGF7 | 2.094802557 | 2.18E-38 | 1.72E-36 | upregulated |
| DNM3OS | 2.094736729 | 2.17E-33 | 1.11E-31 | upregulated |
| MYH1 | 2.094175576 | 8.20E-11 | 7.06E-10 | upregulated |
| OLFML2B | 2.093445688 | 2.74E-66 | 5.52E-62 | upregulated |
| LRRC2 | 2.092212174 | 7.20E-16 | 9.80E-15 | upregulated |
| ADAMTS2 | 2.091183287 | 4.04E-61 | 1.16E-57 | upregulated |
| CTSK | 2.088289746 | 7.77E-64 | 3.92E-60 | upregulated |
| SLN | 2.086456285 | 9.51E-11 | 8.13E-10 | upregulated |
| CMA1 | 2.08096803 | 3.86E-17 | 5.81E-16 | upregulated |
| YIPF7 | 2.078743104 | 7.37E-12 | 7.03E-11 | upregulated |
| SMPX | 2.077967859 | 2.18E-09 | 1.59E-08 | upregulated |
| EMILIN1 | 2.072643032 | 3.70E-57 | 3.93E-54 | upregulated |
| TCAP | 2.071262869 | 2.86E-07 | 1.53E-06 | upregulated |
| MYMK | 2.06417133 | 1.86E-11 | 1.71E-10 | upregulated |
| OLFML3 | 2.064087277 | 2.49E-63 | 1.00E-59 | upregulated |
| XIRP1 | 2.063918157 | 1.44E-25 | 3.90E-24 | upregulated |
| HSPB6 | 2.062982722 | 7.06E-18 | 1.12E-16 | upregulated |
| MYBPH | 2.058672611 | 7.15E-10 | 5.53E-09 | upregulated |
| WNT2 | 2.057494368 | 3.08E-36 | 1.96E-34 | upregulated |
| HRC | 2.056915726 | 2.22E-13 | 2.45E-12 | upregulated |
| ISLR | 2.055854781 | 3.68E-49 | 9.04E-47 | upregulated |
| MYOD1 | 2.055472527 | 1.58E-07 | 8.77E-07 | upregulated |
| CHRDL2 | 2.054326677 | 8.00E-21 | 1.55E-19 | upregulated |
| ASB5 | 2.052336306 | 2.90E-09 | 2.09E-08 | upregulated |
| GRP | 2.048875013 | 1.65E-21 | 3.36E-20 | upregulated |
| MXRA5Y | 2.040817842 | 7.93E-15 | 9.85E-14 | upregulated |
| JAML | 2.039527634 | 3.90E-13 | 4.22E-12 | upregulated |
| DES | 2.035515493 | 1.08E-12 | 1.12E-11 | upregulated |
| BICC1 | 2.03419968 | 7.21E-61 | 1.82E-57 | upregulated |
| MRC1 | 2.032527656 | 6.03E-41 | 6.36E-39 | upregulated |
| SGCA | 2.030859606 | 1.01E-12 | 1.05E-11 | upregulated |
| MMP11 | 2.030189042 | 7.95E-33 | 3.88E-31 | upregulated |
| DUPD1 | 2.026170303 | 7.06E-10 | 5.47E-09 | upregulated |
| MYBPC1 | 2.021927428 | 1.08E-08 | 7.16E-08 | upregulated |
| NT5C1A | 2.020695322 | 5.02E-07 | 2.56E-06 | upregulated |
| HMCN1 | 2.017192847 | 1.12E-40 | 1.11E-38 | upregulated |
| NEGR1 | 2.014406322 | 2.14E-43 | 2.94E-41 | upregulated |
| MYL4 | 2.01357211 | 1.26E-10 | 1.06E-09 | upregulated |
| MAB21L2 | 2.011371209 | 3.50E-09 | 2.51E-08 | upregulated |
| AC099786.1 | 2.009935432 | 1.53E-19 | 2.75E-18 | upregulated |
| MXRA5 | 1.998979375 | 3.09E-49 | 7.80E-47 | upregulated |
| C1QTNF7 | 1.991439344 | 3.74E-35 | 2.22E-33 | upregulated |
| CAV3 | 1.991229334 | 4.66E-08 | 2.82E-07 | upregulated |
| CKMT2 | 1.989519774 | 6.08E-07 | 3.05E-06 | upregulated |
| AP000851.2 | 1.986407274 | 0.002561747 | 0.005522788 | upregulated |
| AC245297.1 | 1.983109968 | 1.12E-28 | 3.86E-27 | upregulated |
| NID2 | 1.983107608 | 1.59E-48 | 3.59E-46 | upregulated |
| AEBP1 | 1.980712883 | 5.97E-56 | 4.77E-53 | upregulated |
| SYPL2 | 1.980454255 | 5.17E-19 | 8.99E-18 | upregulated |
| AP000904.1 | 1.971537562 | 3.54E-10 | 2.86E-09 | upregulated |
| AC053503.6 | 1.971130093 | 1.71E-09 | 1.27E-08 | upregulated |
| MYL1 | 1.970263856 | 7.16E-09 | 4.89E-08 | upregulated |
| AC079298.3 | 1.968728115 | 6.86E-38 | 5.07E-36 | upregulated |
| DPT | 1.964852582 | 2.04E-35 | 1.23E-33 | upregulated |
| AC002398.2 | 1.964406176 | 6.28E-12 | 6.03E-11 | upregulated |
| PTGFR | 1.960183213 | 9.99E-31 | 4.05E-29 | upregulated |
| ZNF469 | 1.959660229 | 3.61E-49 | 8.99E-47 | upregulated |
| AMPD1 | 1.955904783 | 1.91E-13 | 2.12E-12 | upregulated |
| CNTN3 | 1.95430004 | 1.66E-22 | 3.63E-21 | upregulated |
| IGLV3-16 | 1.953383425 | 0.00031152 | 0.000854287 | upregulated |
| OLFML1 | 1.953240512 | 6.78E-64 | 3.92E-60 | upregulated |
| TXLNB | 1.952819664 | 1.13E-19 | 2.05E-18 | upregulated |
| LMOD1 | 1.945338854 | 1.69E-50 | 5.31E-48 | upregulated |
| COL26A1 | 1.941957368 | 2.58E-07 | 1.39E-06 | upregulated |
| TIMP2 | 1.941156801 | 4.85E-65 | 4.89E-61 | upregulated |
| AC112721.2 | 1.940429107 | 1.80E-33 | 9.29E-32 | upregulated |
| NMRK2 | 1.928154557 | 2.37E-09 | 1.73E-08 | upregulated |
| COL5A2 | 1.926040709 | 2.01E-48 | 4.50E-46 | upregulated |
| PTPRN | 1.925750592 | 2.25E-15 | 2.94E-14 | upregulated |
| ADAM12 | 1.924150498 | 1.66E-42 | 2.07E-40 | upregulated |
| SULF1 | 1.922761271 | 3.49E-51 | 1.21E-48 | upregulated |
| CAPN6 | 1.922443914 | 2.77E-14 | 3.31E-13 | upregulated |
| EBF2 | 1.921133041 | 5.10E-42 | 5.91E-40 | upregulated |
| KCND2 | 1.92070106 | 5.59E-41 | 5.94E-39 | upregulated |
| DCN | 1.919529507 | 6.73E-55 | 4.68E-52 | upregulated |
| NCAM1 | 1.918662635 | 9.05E-16 | 1.22E-14 | upregulated |
| SPON1 | 1.916497162 | 1.41E-43 | 2.00E-41 | upregulated |
| NAP1L3 | 1.914151403 | 1.15E-44 | 1.75E-42 | upregulated |
| PCOLCE | 1.911231166 | 1.26E-54 | 8.19E-52 | upregulated |
| IGKV1D-12 | 1.9096366 | 0.000628308 | 0.001595119 | upregulated |
| ABCA8 | 1.909237401 | 1.05E-24 | 2.71E-23 | upregulated |
| CCL18 | 1.905380058 | 2.52E-30 | 9.92E-29 | upregulated |
| SSC5D | 1.903063775 | 5.12E-44 | 7.54E-42 | upregulated |
| SGCG | 1.902739166 | 2.44E-12 | 2.45E-11 | upregulated |
| MIR1-1HG | 1.901968048 | 1.37E-09 | 1.03E-08 | upregulated |
| ADAMTS5 | 1.900830641 | 9.50E-43 | 1.24E-40 | upregulated |
| MYOG | 1.900785262 | 1.42E-10 | 1.19E-09 | upregulated |
| MEOX2 | 1.898002598 | 3.54E-35 | 2.11E-33 | upregulated |
| ACTG2 | 1.895367574 | 1.52E-21 | 3.10E-20 | upregulated |
| PLN | 1.893587854 | 1.65E-30 | 6.59E-29 | upregulated |
| ABRA | 1.893419472 | 2.97E-05 | 0.00010406 | upregulated |
| P4HA3 | 1.888848861 | 1.75E-40 | 1.72E-38 | upregulated |
| CPXM1 | 1.886475736 | 1.67E-46 | 2.92E-44 | upregulated |
| ACTC1 | 1.88606086 | 2.31E-13 | 2.54E-12 | upregulated |
| ANO5 | 1.884181023 | 2.58E-14 | 3.09E-13 | upregulated |
| ZNF423 | 1.880567958 | 7.13E-51 | 2.32E-48 | upregulated |
| RBM24 | 1.879130254 | 9.78E-17 | 1.43E-15 | upregulated |
| CD163 | 1.87824229 | 5.45E-42 | 6.25E-40 | upregulated |
| SPARC | 1.876354957 | 1.56E-53 | 8.43E-51 | upregulated |
| MYF6 | 1.875741336 | 3.64E-10 | 2.93E-09 | upregulated |
| STAC3 | 1.87499584 | 6.90E-15 | 8.61E-14 | upregulated |
| GREM1 | 1.874698259 | 5.11E-41 | 5.45E-39 | upregulated |
| TRIM55 | 1.869074922 | 3.19E-12 | 3.16E-11 | upregulated |
| DUSP26 | 1.868514965 | 1.25E-11 | 1.17E-10 | upregulated |
| CACNG1 | 1.867925364 | 2.30E-08 | 1.46E-07 | upregulated |
| CXCL12 | 1.866519767 | 7.37E-42 | 8.31E-40 | upregulated |
| MYH3 | 1.866333958 | 1.71E-06 | 7.84E-06 | upregulated |
| KLHL33 | 1.865292077 | 6.74E-16 | 9.20E-15 | upregulated |
| FAM198B | 1.863447707 | 5.22E-62 | 1.76E-58 | upregulated |
| NKX3-2 | 1.862234223 | 9.09E-28 | 2.95E-26 | upregulated |
| PTH1R | 1.861144317 | 1.08E-40 | 1.07E-38 | upregulated |
| MMP2 | 1.860886505 | 1.42E-51 | 5.32E-49 | upregulated |
| FLNC | 1.860735143 | 5.74E-15 | 7.23E-14 | upregulated |
| EPHA3 | 1.858673795 | 1.64E-36 | 1.05E-34 | upregulated |
| IGDCC4 | 1.855269182 | 4.52E-47 | 8.68E-45 | upregulated |
| CNN1 | 1.854711516 | 3.84E-36 | 2.43E-34 | upregulated |
| ADRA2A | 1.853677966 | 1.40E-39 | 1.28E-37 | upregulated |
| AC090125.1 | 1.851691684 | 3.33E-15 | 4.29E-14 | upregulated |
| ZNF521 | 1.85083315 | 4.00E-58 | 5.05E-55 | upregulated |
| TLR8 | 1.850656997 | 3.36E-37 | 2.33E-35 | upregulated |
| MYLPF | 1.850247088 | 3.05E-08 | 1.90E-07 | upregulated |
| ECM2 | 1.8458035 | 2.74E-52 | 1.18E-49 | upregulated |
| PDK4 | 1.845631659 | 5.49E-23 | 1.24E-21 | upregulated |
| SPTB | 1.843321042 | 1.03E-10 | 8.79E-10 | upregulated |
| SUSD2 | 1.843232129 | 3.17E-26 | 9.09E-25 | upregulated |
| CTGF | 1.841751079 | 2.13E-40 | 2.08E-38 | upregulated |
| COL5A1 | 1.840923637 | 1.04E-44 | 1.62E-42 | upregulated |
| SMTNL1 | 1.8391941 | 0.001608446 | 0.003655391 | upregulated |
| AQP9 | 1.838745711 | 4.59E-21 | 9.08E-20 | upregulated |
| COL14A1 | 1.837589996 | 1.95E-42 | 2.42E-40 | upregulated |
| RUNX1T1 | 1.837194805 | 1.58E-46 | 2.82E-44 | upregulated |
| GREM2 | 1.835220014 | 1.52E-28 | 5.17E-27 | upregulated |
| FCN1 | 1.830273669 | 1.63E-26 | 4.76E-25 | upregulated |
| FSD2 | 1.815444251 | 0.000181849 | 0.000530407 | upregulated |
| MEF2C | 1.814460532 | 1.40E-34 | 8.00E-33 | upregulated |
| AP001189.3 | 1.80788414 | 6.60E-39 | 5.48E-37 | upregulated |
| CACNG6 | 1.806583793 | 6.47E-10 | 5.05E-09 | upregulated |
| LHX8 | 1.804235509 | 2.13E-27 | 6.66E-26 | upregulated |
| DDR2 | 1.803740571 | 9.89E-52 | 3.91E-49 | upregulated |
| RARRES2 | 1.802623279 | 1.78E-43 | 2.45E-41 | upregulated |
| ZNF385D | 1.80033905 | 2.45E-42 | 3.01E-40 | upregulated |
| MMP16 | 1.798335169 | 2.04E-37 | 1.44E-35 | upregulated |
| MUSTN1 | 1.798280275 | 4.88E-08 | 2.94E-07 | upregulated |
| CTD-2201I18.1 | 1.795456076 | 3.97E-18 | 6.40E-17 | upregulated |
| PRELP | 1.795188321 | 4.04E-34 | 2.21E-32 | upregulated |
| COL6A2 | 1.793269609 | 5.54E-52 | 2.23E-49 | upregulated |
| APOD | 1.792032254 | 6.99E-31 | 2.88E-29 | upregulated |
| PPP1R27 | 1.790550523 | 8.07E-06 | 3.21E-05 | upregulated |
| TMEM130 | 1.786439672 | 3.19E-33 | 1.61E-31 | upregulated |
| AC134312.5 | 1.784849389 | 2.13E-39 | 1.88E-37 | upregulated |
| MYADML2 | 1.779104085 | 7.61E-06 | 3.04E-05 | upregulated |
| SNED1 | 1.776711307 | 2.62E-40 | 2.52E-38 | upregulated |
| SERPINA5 | 1.775817898 | 2.25E-18 | 3.71E-17 | upregulated |
| COL6A1 | 1.774673276 | 4.08E-51 | 1.40E-48 | upregulated |
| THBS2 | 1.768690103 | 1.16E-45 | 1.92E-43 | upregulated |
| GDF10 | 1.767769497 | 3.08E-22 | 6.62E-21 | upregulated |
| SBK2 | 1.758239522 | 8.60E-07 | 4.18E-06 | upregulated |
| AC026310.2 | 1.75602787 | 8.85E-12 | 8.40E-11 | upregulated |
| PADI2 | 1.750895728 | 6.80E-23 | 1.53E-21 | upregulated |
| MYO18B | 1.745811282 | 2.27E-09 | 1.66E-08 | upregulated |
| MSR1 | 1.745071157 | 1.47E-41 | 1.62E-39 | upregulated |
| TNNI2 | 1.743400089 | 2.99E-05 | 0.00010471 | upregulated |
| COL24A1 | 1.743227181 | 5.16E-43 | 6.85E-41 | upregulated |
| MGP | 1.742003267 | 3.24E-34 | 1.79E-32 | upregulated |
| FNDC5 | 1.740495677 | 6.96E-12 | 6.66E-11 | upregulated |
| LY6H | 1.738334404 | 1.18E-11 | 1.10E-10 | upregulated |
| AC112721.1 | 1.736248838 | 1.64E-25 | 4.42E-24 | upregulated |
| SLC8A3 | 1.735429359 | 1.76E-18 | 2.93E-17 | upregulated |
| CDH15 | 1.734119386 | 2.64E-16 | 3.74E-15 | upregulated |
| PDGFRB | 1.733651354 | 9.48E-58 | 1.13E-54 | upregulated |
| AK5 | 1.732965537 | 1.62E-30 | 6.51E-29 | upregulated |
| FGF18 | 1.732168561 | 7.89E-24 | 1.92E-22 | upregulated |
| DKK2 | 1.732141861 | 5.73E-38 | 4.29E-36 | upregulated |
| CDH11 | 1.731347865 | 6.92E-44 | 1.01E-41 | upregulated |
| CLSTN2 | 1.731103386 | 3.58E-31 | 1.52E-29 | upregulated |
| TAGLN | 1.729595494 | 7.70E-45 | 1.24E-42 | upregulated |
| ACTA2 | 1.727161988 | 2.06E-48 | 4.57E-46 | upregulated |
| ENPP1 | 1.726414038 | 7.52E-39 | 6.20E-37 | upregulated |
| ST6GAL2 | 1.723175285 | 2.05E-29 | 7.54E-28 | upregulated |
| DDN | 1.723018814 | 0.000548478 | 0.001415783 | upregulated |
| ADH1B | 1.71586897 | 6.34E-07 | 3.17E-06 | upregulated |
| SCARF2 | 1.715327945 | 1.29E-43 | 1.85E-41 | upregulated |
| ABI3BP | 1.714962506 | 3.86E-30 | 1.49E-28 | upregulated |
| FOLR2 | 1.714930649 | 4.17E-43 | 5.57E-41 | upregulated |
| LAMA2 | 1.714505421 | 5.02E-41 | 5.39E-39 | upregulated |
| CD209 | 1.714255827 | 4.73E-33 | 2.36E-31 | upregulated |
| GLT8D2 | 1.71060572 | 1.59E-53 | 8.43E-51 | upregulated |
| LUM | 1.710190893 | 1.38E-44 | 2.09E-42 | upregulated |
| CORIN | 1.709599186 | 1.41E-34 | 8.03E-33 | upregulated |
| CHI3L1 | 1.706397089 | 1.49E-19 | 2.69E-18 | upregulated |
| NTM | 1.703655336 | 8.67E-50 | 2.40E-47 | upregulated |
| MXRA8 | 1.702672302 | 1.72E-47 | 3.41E-45 | upregulated |
| SPATA20P1 | 1.7023468 | 1.27E-36 | 8.22E-35 | upregulated |
| RCAN2 | 1.697115287 | 5.19E-49 | 1.25E-46 | upregulated |
| CTHRC1 | 1.69686525 | 3.08E-42 | 3.74E-40 | upregulated |
| TGFB3 | 1.696775196 | 4.75E-55 | 3.42E-52 | upregulated |
| DOK5 | 1.694039929 | 3.08E-47 | 5.97E-45 | upregulated |
| PGM5 | 1.690754289 | 1.38E-29 | 5.17E-28 | upregulated |
| IP6K3 | 1.689632537 | 1.10E-07 | 6.24E-07 | upregulated |
| LGR5 | 1.68961987 | 5.02E-16 | 6.95E-15 | upregulated |
| ABCC9 | 1.689072469 | 5.91E-39 | 4.99E-37 | upregulated |
| VSIG4 | 1.687435407 | 1.49E-37 | 1.07E-35 | upregulated |
| SMTNL2 | 1.687026862 | 8.39E-16 | 1.14E-14 | upregulated |
| IGHV7-81 | 1.686536533 | 0.000260975 | 0.000731384 | upregulated |
| HEPH | 1.686184889 | 8.26E-49 | 1.91E-46 | upregulated |
| TCEAL7 | 1.685436271 | 1.06E-38 | 8.59E-37 | upregulated |
| HS3ST2 | 1.684545934 | 1.16E-17 | 1.81E-16 | upregulated |
| RERGL | 1.67690716 | 1.83E-10 | 1.51E-09 | upregulated |
| TMEM132C | 1.676668264 | 8.22E-10 | 6.32E-09 | upregulated |
| DCLK1 | 1.674318494 | 1.16E-28 | 3.99E-27 | upregulated |
| CTSG | 1.673317045 | 1.64E-16 | 2.36E-15 | upregulated |
| CR1 | 1.672035636 | 1.46E-28 | 4.99E-27 | upregulated |
| ATP10A | 1.672031271 | 9.90E-56 | 7.40E-53 | upregulated |
| JCAD | 1.67056321 | 1.46E-56 | 1.34E-53 | upregulated |
| KCNE4 | 1.669177551 | 2.67E-49 | 6.82E-47 | upregulated |
| TNXB | 1.668552112 | 6.05E-16 | 8.29E-15 | upregulated |
| TRIM63 | 1.66793751 | 3.61E-10 | 2.90E-09 | upregulated |
| BEST3 | 1.666314884 | 9.62E-12 | 9.10E-11 | upregulated |
| TLR4 | 1.666067813 | 1.09E-49 | 2.93E-47 | upregulated |
| PLPP7 | 1.66299795 | 7.04E-37 | 4.67E-35 | upregulated |
| PRKAG3 | 1.662773905 | 3.35E-08 | 2.07E-07 | upregulated |
| RGS4 | 1.659840265 | 6.16E-36 | 3.87E-34 | upregulated |
| PLXNC1 | 1.659628804 | 2.39E-53 | 1.24E-50 | upregulated |
| SCARA5 | 1.656974637 | 2.45E-23 | 5.71E-22 | upregulated |
| IGLV2-34 | 1.656855516 | 0.000698348 | 0.001750903 | upregulated |
| AP001434.1 | 1.654316791 | 1.81E-32 | 8.57E-31 | upregulated |
| SLC36A2 | 1.652964265 | 0.000270407 | 0.000754358 | upregulated |
| FHL1 | 1.652445398 | 1.26E-12 | 1.30E-11 | upregulated |
| PDLIM3 | 1.651692943 | 2.05E-23 | 4.79E-22 | upregulated |
| CYS1 | 1.648127646 | 5.86E-38 | 4.38E-36 | upregulated |
| CA3 | 1.647114597 | 6.42E-08 | 3.80E-07 | upregulated |
| LINCMD1 | 1.646848855 | 1.17E-08 | 7.75E-08 | upregulated |
| KCNA7 | 1.646196428 | 3.73E-05 | 0.000127627 | upregulated |
| FPR2 | 1.645707078 | 1.49E-14 | 1.81E-13 | upregulated |
| PEG3 | 1.645004486 | 1.86E-29 | 6.86E-28 | upregulated |
| LILRB4 | 1.643678334 | 1.78E-31 | 7.67E-30 | upregulated |
| CDH2 | 1.643363205 | 1.76E-26 | 5.14E-25 | upregulated |
| RSPO3 | 1.643284999 | 3.86E-42 | 4.53E-40 | upregulated |
| CADM3 | 1.642489797 | 1.41E-25 | 3.83E-24 | upregulated |
| SPX | 1.641890927 | 8.80E-09 | 5.93E-08 | upregulated |
| ADGRA2 | 1.640925998 | 4.64E-50 | 1.36E-47 | upregulated |
| THY1 | 1.639324684 | 4.44E-48 | 9.63E-46 | upregulated |
| LRTM1 | 1.637689655 | 0.003423001 | 0.007135996 | upregulated |
| CLEC4G | 1.637342257 | 1.90E-13 | 2.11E-12 | upregulated |
| DHH | 1.636445463 | 3.04E-28 | 1.01E-26 | upregulated |
| CSDC2 | 1.636218366 | 1.45E-21 | 2.97E-20 | upregulated |
| TCEAL5 | 1.63575363 | 2.20E-11 | 2.01E-10 | upregulated |
| IGKV1D-33 | 1.634810431 | 0.001313298 | 0.003054723 | upregulated |
| TNNC2 | 1.63405781 | 2.37E-06 | 1.05E-05 | upregulated |
| ZEB2 | 1.633126476 | 3.23E-60 | 7.25E-57 | upregulated |
| FPR3 | 1.632267497 | 3.52E-42 | 4.23E-40 | upregulated |
| ENO3 | 1.627806703 | 6.19E-07 | 3.10E-06 | upregulated |
| CD84 | 1.62672675 | 3.36E-35 | 2.01E-33 | upregulated |
| CYBB | 1.622487162 | 2.23E-37 | 1.57E-35 | upregulated |
| CCR2 | 1.620608819 | 1.79E-28 | 6.05E-27 | upregulated |
| DAB2 | 1.620551643 | 9.17E-59 | 1.32E-55 | upregulated |
| SLC24A2 | 1.620066987 | 2.55E-28 | 8.49E-27 | upregulated |
| IGF2 | 1.618712637 | 3.49E-32 | 1.61E-30 | upregulated |
| PPEF1 | 1.61766304 | 4.35E-30 | 1.67E-28 | upregulated |
| FBLN5 | 1.615332683 | 2.24E-54 | 1.37E-51 | upregulated |
| SYNDIG1 | 1.614798064 | 7.24E-32 | 3.26E-30 | upregulated |
| AC009093.1 | 1.614548359 | 1.50E-41 | 1.66E-39 | upregulated |
| HSD11B1 | 1.613996503 | 4.60E-37 | 3.15E-35 | upregulated |
| RASGRF2 | 1.61244889 | 6.24E-54 | 3.59E-51 | upregulated |
| CPZ | 1.610455411 | 7.48E-34 | 4.02E-32 | upregulated |
| ELN | 1.610107036 | 3.50E-23 | 8.09E-22 | upregulated |
| SYNPO2L | 1.610042417 | 4.08E-07 | 2.12E-06 | upregulated |
| AL136084.3 | 1.608237438 | 4.56E-32 | 2.08E-30 | upregulated |
| LINC02544 | 1.607748058 | 7.61E-22 | 1.59E-20 | upregulated |
| CHRDL1 | 1.606856893 | 5.49E-19 | 9.52E-18 | upregulated |
| FHL5 | 1.606611747 | 3.37E-28 | 1.12E-26 | upregulated |
| LINC00702 | 1.603368599 | 1.76E-23 | 4.15E-22 | upregulated |
| S100A1 | 1.602795536 | 7.67E-06 | 3.06E-05 | upregulated |
| DAAM2 | 1.601008729 | 6.29E-49 | 1.49E-46 | upregulated |
| PCDH10 | 1.598450129 | 2.67E-08 | 1.68E-07 | upregulated |
| ANGPTL2 | 1.598106512 | 2.58E-50 | 7.76E-48 | upregulated |
| HK3 | 1.597720586 | 5.59E-29 | 1.98E-27 | upregulated |
| MAGI2-AS3 | 1.597068563 | 1.52E-51 | 5.58E-49 | upregulated |
| IGLV2-8 | 1.596709225 | 4.70E-06 | 1.96E-05 | upregulated |
| ANK2 | 1.596405124 | 2.33E-30 | 9.21E-29 | upregulated |
| FAP | 1.596030259 | 3.85E-39 | 3.33E-37 | upregulated |
| IGLV3-12 | 1.595378478 | 0.000292562 | 0.000808232 | upregulated |
| FABP3 | 1.594082102 | 5.35E-21 | 1.05E-19 | upregulated |
| AP000892.3 | 1.592446221 | 5.67E-39 | 4.83E-37 | upregulated |
| ATP6V0D2 | 1.591494474 | 1.03E-18 | 1.74E-17 | upregulated |
| LRRC14B | 1.590422806 | 2.62E-09 | 1.90E-08 | upregulated |
| NOX4 | 1.58875789 | 1.88E-52 | 8.43E-50 | upregulated |
| CACNA1G | 1.588123516 | 3.08E-24 | 7.70E-23 | upregulated |
| DLK1 | 1.582789466 | 1.65E-05 | 6.14E-05 | upregulated |
| FITM1 | 1.582107921 | 3.11E-05 | 0.000108477 | upregulated |
| LAMA4 | 1.580182009 | 6.05E-57 | 6.11E-54 | upregulated |
| MEDAG | 1.580110178 | 1.47E-37 | 1.07E-35 | upregulated |
| LINC01050 | 1.578756789 | 2.71E-13 | 2.96E-12 | upregulated |
| TLR7 | 1.578562113 | 3.56E-29 | 1.28E-27 | upregulated |
| PCDH18 | 1.572553679 | 7.47E-49 | 1.75E-46 | upregulated |
| BGN | 1.571967487 | 1.15E-43 | 1.65E-41 | upregulated |
| AC112715.1 | 1.571399831 | 4.47E-14 | 5.22E-13 | upregulated |
| FCAR | 1.570731548 | 1.28E-11 | 1.19E-10 | upregulated |
| THSD7A | 1.570059504 | 2.47E-34 | 1.38E-32 | upregulated |
| DCHS1 | 1.569902475 | 8.33E-40 | 7.68E-38 | upregulated |
| AMPH | 1.567962174 | 6.90E-48 | 1.43E-45 | upregulated |
| MYLK2 | 1.567203538 | 3.96E-09 | 2.81E-08 | upregulated |
| CCDC80 | 1.566513357 | 4.58E-40 | 4.34E-38 | upregulated |
| SOX11 | 1.565330882 | 3.84E-24 | 9.59E-23 | upregulated |
| VGLL3 | 1.562066318 | 6.21E-36 | 3.89E-34 | upregulated |
| SGIP1 | 1.56169124 | 7.90E-45 | 1.27E-42 | upregulated |
| GUCY1A2 | 1.558185531 | 5.50E-42 | 6.27E-40 | upregulated |
| PDE3A | 1.557571922 | 6.01E-40 | 5.64E-38 | upregulated |
| APELA | 1.556260727 | 5.40E-17 | 8.02E-16 | upregulated |
| PDZRN4 | 1.555634888 | 2.94E-30 | 1.15E-28 | upregulated |
| MMRN1 | 1.553766558 | 3.06E-16 | 4.31E-15 | upregulated |
| LAMP5 | 1.551775028 | 3.19E-34 | 1.77E-32 | upregulated |
| ADAMDEC1 | 1.551521589 | 1.40E-22 | 3.07E-21 | upregulated |
| DCSTAMP | 1.550866893 | 1.40E-25 | 3.81E-24 | upregulated |
| SLCO2B1 | 1.550577696 | 4.12E-41 | 4.44E-39 | upregulated |
| IGKV1-17 | 1.54966514 | 7.62E-06 | 3.05E-05 | upregulated |
| MS4A4A | 1.549384125 | 9.99E-41 | 1.00E-38 | upregulated |
| FAM13C | 1.548838185 | 3.38E-29 | 1.22E-27 | upregulated |
| AC129507.1 | 1.548607548 | 5.38E-15 | 6.79E-14 | upregulated |
| UNC5C | 1.548371242 | 5.95E-47 | 1.11E-44 | upregulated |
| KLHL30 | 1.547116841 | 9.81E-09 | 6.56E-08 | upregulated |
| GFRA1 | 1.544977258 | 4.30E-25 | 1.14E-23 | upregulated |
| PTGER2 | 1.544736102 | 3.80E-39 | 3.32E-37 | upregulated |
| LRRC39 | 1.543659722 | 0.000528779 | 0.001368965 | upregulated |
| HTR1B | 1.542564787 | 4.34E-27 | 1.32E-25 | upregulated |
| CCL13 | 1.541819784 | 1.98E-27 | 6.22E-26 | upregulated |
| JAM2 | 1.53816143 | 7.02E-50 | 1.97E-47 | upregulated |
| PLPPR3 | 1.537894583 | 1.68E-09 | 1.25E-08 | upregulated |
| CACNA1C | 1.537501259 | 1.96E-45 | 3.21E-43 | upregulated |
| DUSP13 | 1.536731884 | 2.64E-05 | 9.39E-05 | upregulated |
| LINC00565 | 1.534561954 | 1.82E-28 | 6.13E-27 | upregulated |
| ABCA6 | 1.533131878 | 6.94E-43 | 9.10E-41 | upregulated |
| LINC02104 | 1.53038736 | 6.20E-18 | 9.85E-17 | upregulated |
| NHSL2 | 1.530293909 | 5.04E-29 | 1.79E-27 | upregulated |
| AOC3 | 1.529460248 | 1.56E-43 | 2.19E-41 | upregulated |
| PLAC9 | 1.52655214 | 1.50E-28 | 5.11E-27 | upregulated |
| LINC01854 | 1.522776902 | 1.80E-05 | 6.64E-05 | upregulated |
| FMOD | 1.520704829 | 2.29E-50 | 7.09E-48 | upregulated |
| MAP1A | 1.516054462 | 2.32E-38 | 1.82E-36 | upregulated |
| IGKV1OR2-108 | 1.515178959 | 1.42E-05 | 5.35E-05 | upregulated |
| AKAP12 | 1.515080868 | 1.82E-38 | 1.44E-36 | upregulated |
| CCL7 | 1.512595744 | 2.52E-13 | 2.76E-12 | upregulated |
| SDC2 | 1.511434743 | 6.51E-39 | 5.43E-37 | upregulated |
| CRISPLD2 | 1.51112469 | 2.73E-48 | 5.98E-46 | upregulated |
| CALHM5 | 1.509409919 | 2.19E-47 | 4.29E-45 | upregulated |
| LILRA4 | 1.509124445 | 3.29E-15 | 4.24E-14 | upregulated |
| LRRC32 | 1.508927434 | 7.01E-57 | 6.74E-54 | upregulated |
| ZEB1 | 1.508435429 | 4.38E-52 | 1.84E-49 | upregulated |
| MYHAS | 1.507625473 | 3.07E-08 | 1.91E-07 | upregulated |
| METTL11B | 1.507473738 | 2.16E-18 | 3.58E-17 | upregulated |
| COL15A1 | 1.506205248 | 8.33E-41 | 8.53E-39 | upregulated |
| MYL9 | 1.505482527 | 1.47E-39 | 1.33E-37 | upregulated |
| WDR86 | 1.505189792 | 7.15E-37 | 4.73E-35 | upregulated |
| AC244669.2 | 1.50283548 | 4.15E-10 | 3.31E-09 | upregulated |
| FBLN2 | 1.502315465 | 2.56E-42 | 3.13E-40 | upregulated |
| FXYD1 | 1.500912279 | 3.38E-12 | 3.34E-11 | upregulated |
| LPL | 1.499156335 | 1.23E-31 | 5.37E-30 | upregulated |
| HSPB3 | 1.495820418 | 1.40E-07 | 7.87E-07 | upregulated |
| FSTL1 | 1.49476704 | 4.73E-50 | 1.36E-47 | upregulated |
| STMN2 | 1.494095511 | 6.35E-07 | 3.17E-06 | upregulated |
| HTRA3 | 1.492340073 | 1.08E-38 | 8.69E-37 | upregulated |
| KISS1 | 1.492283016 | 0.014043427 | 0.024662164 | upregulated |
| IGKV1-16 | 1.492159362 | 4.21E-06 | 1.78E-05 | upregulated |
| PDGFRA | 1.486688634 | 3.96E-46 | 6.77E-44 | upregulated |
| LINC00968 | 1.485603578 | 6.51E-28 | 2.14E-26 | upregulated |
| C1orf105 | 1.48355653 | 6.79E-05 | 0.000220016 | upregulated |
| C3orf80 | 1.482764935 | 9.43E-41 | 9.52E-39 | upregulated |
| TNNT3 | 1.482727765 | 2.03E-05 | 7.39E-05 | upregulated |
| CSF1R | 1.482701669 | 3.61E-42 | 4.29E-40 | upregulated |
| CCL11 | 1.482018548 | 1.81E-20 | 3.44E-19 | upregulated |
| FMO1 | 1.481201493 | 1.07E-26 | 3.19E-25 | upregulated |
| SYNE1 | 1.480648238 | 6.08E-41 | 6.36E-39 | upregulated |
| GPD1 | 1.480437359 | 2.53E-05 | 9.03E-05 | upregulated |
| C2orf40 | 1.480201978 | 1.45E-12 | 1.49E-11 | upregulated |
| SERPINA9 | 1.479883149 | 0.00536299 | 0.010606137 | upregulated |
| SIGLEC1 | 1.479525128 | 2.76E-28 | 9.20E-27 | upregulated |
| ANKRD1 | 1.47905613 | 1.85E-11 | 1.70E-10 | upregulated |
| SIGLEC7 | 1.477218971 | 1.77E-37 | 1.27E-35 | upregulated |
| TRIM54 | 1.477079282 | 0.000380969 | 0.001023458 | upregulated |
| RETN | 1.476887056 | 6.14E-14 | 7.09E-13 | upregulated |
| APLNR | 1.473989115 | 1.52E-35 | 9.33E-34 | upregulated |
| ACAN | 1.473702626 | 5.23E-23 | 1.19E-21 | upregulated |
| PRRX1 | 1.472686575 | 1.39E-51 | 5.27E-49 | upregulated |
| TBX5-AS1 | 1.471752278 | 4.78E-12 | 4.66E-11 | upregulated |
| AL354732.1 | 1.469540571 | 4.62E-38 | 3.49E-36 | upregulated |
| CES5A | 1.468271759 | 0.001930403 | 0.004306907 | upregulated |
| MUSK | 1.466685543 | 6.10E-11 | 5.31E-10 | upregulated |
| ADCY2 | 1.466194997 | 2.78E-15 | 3.60E-14 | upregulated |
| A2M | 1.461658847 | 8.08E-55 | 5.43E-52 | upregulated |
| OIT3 | 1.461246839 | 6.31E-22 | 1.33E-20 | upregulated |
| AC090559.1 | 1.459913758 | 2.84E-34 | 1.58E-32 | upregulated |
| RF00265 | 1.45763409 | 8.42E-07 | 4.10E-06 | upregulated |
| CMKLR1 | 1.457478354 | 7.36E-41 | 7.61E-39 | upregulated |
| DNAJC5B | 1.457338393 | 1.41E-18 | 2.36E-17 | upregulated |
| AP003071.4 | 1.453383967 | 4.12E-33 | 2.07E-31 | upregulated |
| SCN1B | 1.452605038 | 4.47E-32 | 2.04E-30 | upregulated |
| C4A | 1.452505831 | 4.72E-26 | 1.33E-24 | upregulated |
| GJA5 | 1.447152899 | 2.18E-32 | 1.02E-30 | upregulated |
| CXCL5 | 1.445691864 | 0.000986467 | 0.002375574 | upregulated |
| PPIAP39 | 1.445099179 | 1.01E-18 | 1.71E-17 | upregulated |
| TSPAN11 | 1.444094574 | 1.26E-28 | 4.34E-27 | upregulated |
| IGHV1OR16-3 | 1.443264829 | 0.000568114 | 0.001461981 | upregulated |
| HMGN1P13 | 1.4413568 | 2.35E-12 | 2.37E-11 | upregulated |
| PROK2 | 1.441057943 | 0.002499201 | 0.005405025 | upregulated |
| PLA2G2D | 1.441013389 | 1.23E-12 | 1.28E-11 | upregulated |
| C3AR1 | 1.43609909 | 8.29E-39 | 6.74E-37 | upregulated |
| ITGB3 | 1.430945179 | 2.94E-34 | 1.63E-32 | upregulated |
| ADAMTSL1 | 1.430305861 | 5.06E-39 | 4.33E-37 | upregulated |
| GLIS3 | 1.428953287 | 1.62E-41 | 1.78E-39 | upregulated |
| SCT | 1.427944736 | 4.09E-22 | 8.75E-21 | upregulated |
| SRL | 1.426064931 | 1.39E-08 | 9.08E-08 | upregulated |
| MRGPRF | 1.42593786 | 1.58E-43 | 2.19E-41 | upregulated |
| SYNPO2 | 1.425382087 | 2.48E-16 | 3.52E-15 | upregulated |
| TMEM26 | 1.422185944 | 3.26E-42 | 3.94E-40 | upregulated |
| IGHV3-43 | 1.418721622 | 0.000431048 | 0.001143681 | upregulated |
| AL109924.2 | 1.418712442 | 4.11E-15 | 5.23E-14 | upregulated |
| SIGLEC8 | 1.41862727 | 3.75E-23 | 8.63E-22 | upregulated |
| MSRB3 | 1.417689559 | 7.64E-47 | 1.39E-44 | upregulated |
| MS4A7 | 1.417265096 | 1.17E-37 | 8.57E-36 | upregulated |
| CCL23 | 1.417256779 | 6.06E-21 | 1.19E-19 | upregulated |
| HAS1 | 1.416678149 | 1.18E-06 | 5.61E-06 | upregulated |
| TM6SF1 | 1.41212851 | 1.52E-40 | 1.50E-38 | upregulated |
| MPEG1 | 1.412104803 | 1.35E-29 | 5.08E-28 | upregulated |
| AC008687.6 | 1.411567294 | 0.022912087 | 0.037857852 | upregulated |
| F2RL2 | 1.406610552 | 1.07E-30 | 4.33E-29 | upregulated |
| OSM | 1.405608169 | 8.96E-13 | 9.39E-12 | upregulated |
| ABCA9 | 1.40550968 | 1.12E-28 | 3.86E-27 | upregulated |
| LINC02345 | 1.404227221 | 3.08E-14 | 3.66E-13 | upregulated |
| IGKV1D-16 | 1.40106574 | 0.000105368 | 0.000325792 | upregulated |
| IGKV2-28 | 1.399685367 | 0.00039356 | 0.001052799 | upregulated |
| CCR1 | 1.398871463 | 9.07E-32 | 4.01E-30 | upregulated |
| AC068700.1 | 1.396269445 | 1.44E-13 | 1.61E-12 | upregulated |
| GUCY1A1 | 1.3957314 | 6.77E-39 | 5.60E-37 | upregulated |
| TMEM233 | 1.395564675 | 5.88E-24 | 1.44E-22 | upregulated |
| PIK3CG | 1.395112628 | 2.02E-28 | 6.76E-27 | upregulated |
| ODAM | 1.394399704 | 0.008923828 | 0.016601973 | upregulated |
| FGL2 | 1.39337861 | 5.11E-28 | 1.68E-26 | upregulated |
| GXYLT2 | 1.393193759 | 4.86E-37 | 3.29E-35 | upregulated |
| CASS4 | 1.392341362 | 3.05E-33 | 1.54E-31 | upregulated |
| CREB3L1 | 1.390202738 | 3.51E-35 | 2.10E-33 | upregulated |
| GGT5 | 1.389295559 | 3.21E-53 | 1.62E-50 | upregulated |
| CD1B | 1.388266081 | 1.28E-17 | 1.99E-16 | upregulated |
| AL139147.1 | 1.387323722 | 1.22E-18 | 2.05E-17 | upregulated |
| SIRPB1 | 1.387208216 | 1.05E-18 | 1.77E-17 | upregulated |
| SLAMF8 | 1.386512017 | 9.05E-41 | 9.22E-39 | upregulated |
| FBXL7 | 1.386462489 | 4.14E-46 | 7.02E-44 | upregulated |
| FCGR3A | 1.384046861 | 2.51E-32 | 1.17E-30 | upregulated |
| GPR34 | 1.383611062 | 1.80E-33 | 9.28E-32 | upregulated |
| FAT4 | 1.383460338 | 1.32E-29 | 4.99E-28 | upregulated |
| LINC00654 | 1.382517272 | 2.84E-43 | 3.87E-41 | upregulated |
| TPM2 | 1.382503177 | 5.70E-21 | 1.12E-19 | upregulated |
| MYOM2 | 1.382244029 | 0.000186146 | 0.000540983 | upregulated |
| AC005165.1 | 1.38196998 | 1.38E-06 | 6.44E-06 | upregulated |
| CGB8 | 1.381728764 | 0.025141205 | 0.041160215 | upregulated |
| ANPEP | 1.381678699 | 3.99E-44 | 5.92E-42 | upregulated |
| GPIHBP1 | 1.381363286 | 3.34E-21 | 6.70E-20 | upregulated |
| TM6SF2 | 1.379877105 | 1.89E-28 | 6.35E-27 | upregulated |
| FPR1 | 1.379708773 | 6.00E-33 | 2.96E-31 | upregulated |
| COL12A1 | 1.379297385 | 2.39E-33 | 1.22E-31 | upregulated |
| ANTXR1 | 1.378511492 | 1.04E-39 | 9.48E-38 | upregulated |
| ASB12 | 1.378456728 | 0.003798156 | 0.00781873 | upregulated |
| STARD8 | 1.37739888 | 1.55E-51 | 5.58E-49 | upregulated |
| SUCNR1 | 1.377047404 | 4.43E-28 | 1.46E-26 | upregulated |
| PDZRN3 | 1.375027281 | 1.68E-47 | 3.35E-45 | upregulated |
| CD300LB | 1.374524611 | 6.22E-27 | 1.88E-25 | upregulated |
| WISP1 | 1.373451094 | 3.58E-42 | 4.27E-40 | upregulated |
| BMP8A | 1.369651467 | 8.09E-26 | 2.25E-24 | upregulated |
| TFEC | 1.369303544 | 6.88E-30 | 2.61E-28 | upregulated |
| HHIPL1 | 1.368897681 | 6.30E-39 | 5.27E-37 | upregulated |
| ZCCHC24 | 1.368034003 | 2.17E-54 | 1.37E-51 | upregulated |
| ADAMTS4 | 1.367288288 | 2.78E-37 | 1.94E-35 | upregulated |
| CST1 | 1.366528178 | 2.81E-14 | 3.35E-13 | upregulated |
| PXDN | 1.365767611 | 5.29E-33 | 2.62E-31 | upregulated |
| ITGB2 | 1.365651676 | 8.43E-32 | 3.76E-30 | upregulated |
| C5AR1 | 1.365539109 | 2.01E-35 | 1.22E-33 | upregulated |
| DACT3 | 1.364656449 | 3.17E-34 | 1.76E-32 | upregulated |
| PHKG1 | 1.364593196 | 1.91E-07 | 1.05E-06 | upregulated |
| LILRB5 | 1.363093448 | 4.86E-25 | 1.28E-23 | upregulated |
| PRKG1 | 1.362937564 | 3.73E-41 | 4.04E-39 | upregulated |
| C4B | 1.361612928 | 9.11E-21 | 1.76E-19 | upregulated |
| IGHV3OR16-9 | 1.360549435 | 1.45E-05 | 5.44E-05 | upregulated |
| EPDR1 | 1.359799263 | 8.82E-40 | 8.09E-38 | upregulated |
| KIF26B | 1.359159464 | 1.45E-35 | 8.93E-34 | upregulated |
| CAMK2A | 1.358152829 | 1.88E-13 | 2.08E-12 | upregulated |
| IGKV1D-17 | 1.356951314 | 0.000728721 | 0.001818017 | upregulated |
| FCGR3B | 1.35620792 | 7.46E-09 | 5.08E-08 | upregulated |
| BHMT2 | 1.355389782 | 9.37E-31 | 3.83E-29 | upregulated |
| RELN | 1.354186171 | 6.87E-15 | 8.57E-14 | upregulated |
| SIGLEC15 | 1.35381009 | 1.35E-18 | 2.26E-17 | upregulated |
| CD163L1 | 1.352713756 | 2.51E-28 | 8.39E-27 | upregulated |
| TEK | 1.352615754 | 1.75E-39 | 1.57E-37 | upregulated |
| CD300C | 1.351702522 | 4.55E-34 | 2.49E-32 | upregulated |
| HHIP-AS1 | 1.350511584 | 4.48E-21 | 8.88E-20 | upregulated |
| ADAMTS8 | 1.349850245 | 2.47E-18 | 4.07E-17 | upregulated |
| LRCH2 | 1.349501568 | 7.80E-44 | 1.13E-41 | upregulated |
| MMP12 | 1.346252158 | 9.23E-16 | 1.25E-14 | upregulated |
| SCG2 | 1.344165548 | 9.82E-23 | 2.19E-21 | upregulated |
| LRRC37A7P | 1.342512869 | 4.45E-15 | 5.65E-14 | upregulated |
| HAND2 | 1.342296429 | 2.59E-22 | 5.60E-21 | upregulated |
| PLXDC1 | 1.341063374 | 3.96E-46 | 6.77E-44 | upregulated |
| IGHV1OR15-2 | 1.339040318 | 1.82E-06 | 8.26E-06 | upregulated |
| GFRA2 | 1.338607651 | 5.15E-25 | 1.35E-23 | upregulated |
| BNC2-AS1 | 1.33754527 | 3.59E-15 | 4.61E-14 | upregulated |
| AOX1 | 1.336854783 | 7.99E-18 | 1.26E-16 | upregulated |
| NTRK1 | 1.335102858 | 4.24E-16 | 5.92E-15 | upregulated |
| KCNJ8 | 1.334720656 | 3.01E-46 | 5.24E-44 | upregulated |
| CCL2 | 1.33336354 | 8.64E-26 | 2.38E-24 | upregulated |
| CCL14 | 1.329852832 | 7.96E-15 | 9.88E-14 | upregulated |
| JPH2 | 1.329574857 | 1.77E-14 | 2.15E-13 | upregulated |
| AP001528.3 | 1.327908689 | 4.96E-26 | 1.39E-24 | upregulated |
| PMP22 | 1.327562877 | 7.62E-60 | 1.40E-56 | upregulated |
| IGLV6-57 | 1.32756093 | 0.000654667 | 0.001653715 | upregulated |
| CD93 | 1.327000021 | 6.47E-45 | 1.05E-42 | upregulated |
| P2RY12 | 1.325516983 | 1.73E-19 | 3.11E-18 | upregulated |
| SELP | 1.324598017 | 4.16E-13 | 4.48E-12 | upregulated |
| PKNOX2 | 1.322800892 | 8.84E-17 | 1.30E-15 | upregulated |
| HNMT | 1.32177341 | 8.33E-48 | 1.71E-45 | upregulated |
| TMOD1 | 1.32086124 | 1.30E-07 | 7.31E-07 | upregulated |
| TBX15 | 1.32007567 | 9.64E-23 | 2.15E-21 | upregulated |
| MNDA | 1.319469811 | 5.06E-33 | 2.51E-31 | upregulated |
| CCR8 | 1.319058745 | 1.54E-27 | 4.90E-26 | upregulated |
| AC023449.2 | 1.318408792 | 5.49E-34 | 2.98E-32 | upregulated |
| AKAP6 | 1.314905067 | 1.91E-08 | 1.23E-07 | upregulated |
| C5orf46 | 1.314444055 | 1.32E-10 | 1.10E-09 | upregulated |
| KIAA1755 | 1.314218657 | 3.24E-44 | 4.85E-42 | upregulated |
| MAP1LC3C | 1.314206605 | 4.36E-24 | 1.08E-22 | upregulated |
| PDE7B | 1.314115024 | 1.49E-42 | 1.89E-40 | upregulated |
| TRBV6-6 | 1.313798321 | 2.83E-17 | 4.30E-16 | upregulated |
| TMEM176B | 1.313369217 | 6.88E-40 | 6.42E-38 | upregulated |
| BST1 | 1.312005729 | 6.80E-51 | 2.25E-48 | upregulated |
| LAIR1 | 1.310968207 | 2.17E-34 | 1.23E-32 | upregulated |
| CHSY3 | 1.310040845 | 1.77E-36 | 1.13E-34 | upregulated |
| ALPK3 | 1.309505524 | 1.27E-18 | 2.13E-17 | upregulated |
| NEXN | 1.309362086 | 5.37E-22 | 1.14E-20 | upregulated |
| LILRA2 | 1.309229175 | 4.81E-31 | 2.01E-29 | upregulated |
| SLC7A7 | 1.307557434 | 2.47E-38 | 1.93E-36 | upregulated |
| CCL24 | 1.305891442 | 1.32E-12 | 1.37E-11 | upregulated |
| GLIS1 | 1.305008597 | 2.26E-22 | 4.91E-21 | upregulated |
| RGS18 | 1.303915288 | 9.36E-29 | 3.26E-27 | upregulated |
| ZNF366 | 1.30388073 | 1.44E-24 | 3.69E-23 | upregulated |
| ENPP2 | 1.303267018 | 6.49E-43 | 8.55E-41 | upregulated |
| CD4 | 1.303230274 | 7.27E-35 | 4.23E-33 | upregulated |
| IGHV3-13 | 1.3029345 | 1.61E-06 | 7.40E-06 | upregulated |
| IGKV2D-28 | 1.302105154 | 0.003042886 | 0.006423871 | upregulated |
| TMEM176A | 1.300850551 | 3.02E-40 | 2.87E-38 | upregulated |
| EBF1 | 1.30026139 | 3.30E-50 | 9.80E-48 | upregulated |
| IGHV3-64 | 1.300170996 | 6.46E-05 | 0.000210459 | upregulated |
| SCN5A | 1.299479523 | 1.52E-08 | 9.91E-08 | upregulated |
| ADAMTS7 | 1.299275224 | 6.82E-38 | 5.06E-36 | upregulated |
| EMX2OS | 1.299247085 | 1.38E-19 | 2.50E-18 | upregulated |
| GALNT16 | 1.298931456 | 1.68E-16 | 2.41E-15 | upregulated |
| EFEMP2 | 1.298889989 | 1.38E-38 | 1.09E-36 | upregulated |
| AC011352.3 | 1.298604617 | 2.12E-11 | 1.93E-10 | upregulated |
| GGTA1P | 1.298190667 | 4.53E-33 | 2.27E-31 | upregulated |
| C11orf96 | 1.297180171 | 1.20E-22 | 2.66E-21 | upregulated |
| GRIA3 | 1.296335067 | 2.74E-19 | 4.87E-18 | upregulated |
| COL5A3 | 1.296240879 | 1.28E-31 | 5.56E-30 | upregulated |
| IGKV2OR2-1 | 1.295367805 | 0.003577596 | 0.007411573 | upregulated |
| KRTAP2-3 | 1.294427851 | 0.002104679 | 0.004648956 | upregulated |
| CLEC11A | 1.293664311 | 9.71E-31 | 3.96E-29 | upregulated |
| IGHV1-18 | 1.293485766 | 5.49E-07 | 2.78E-06 | upregulated |
| RNU6-403P | 1.292339648 | 6.85E-12 | 6.56E-11 | upregulated |
| AL161935.3 | 1.292133595 | 1.27E-20 | 2.44E-19 | upregulated |
| RHOJ | 1.291618836 | 4.56E-48 | 9.79E-46 | upregulated |
| DRP2 | 1.28966505 | 5.49E-24 | 1.35E-22 | upregulated |
| APOC1 | 1.289555479 | 4.92E-18 | 7.90E-17 | upregulated |
| IGHV3-21 | 1.289266637 | 6.06E-08 | 3.60E-07 | upregulated |
| IGHV1-69D | 1.288815282 | 1.19E-06 | 5.63E-06 | upregulated |
| MS4A6A | 1.286619873 | 2.45E-34 | 1.38E-32 | upregulated |
| COL6A6 | 1.286238652 | 1.79E-17 | 2.75E-16 | upregulated |
| AC103740.2 | 1.285209585 | 0.008010073 | 0.015084424 | upregulated |
| CLIC5 | 1.283643835 | 3.33E-17 | 5.04E-16 | upregulated |
| LILRA6 | 1.283331845 | 1.57E-28 | 5.33E-27 | upregulated |
| ARHGAP6 | 1.282960785 | 1.36E-42 | 1.73E-40 | upregulated |
| FILIP1L | 1.282755456 | 8.04E-37 | 5.30E-35 | upregulated |
| FCGR2A | 1.280058828 | 7.77E-39 | 6.37E-37 | upregulated |
| LINC01711 | 1.279902047 | 1.21E-12 | 1.26E-11 | upregulated |
| ROS1 | 1.27863927 | 7.17E-08 | 4.22E-07 | upregulated |
| HRH2 | 1.276359029 | 7.84E-31 | 3.21E-29 | upregulated |
| CYTL1 | 1.274718152 | 9.66E-29 | 3.36E-27 | upregulated |
| AL031651.2 | 1.27444955 | 4.24E-26 | 1.20E-24 | upregulated |
| MRAS | 1.274107459 | 1.03E-41 | 1.16E-39 | upregulated |
| TDO2 | 1.273871214 | 8.19E-15 | 1.01E-13 | upregulated |
| ITIH3 | 1.27377161 | 3.20E-19 | 5.67E-18 | upregulated |
| RCN3 | 1.273029352 | 1.98E-34 | 1.12E-32 | upregulated |
| RSPO1 | 1.271913327 | 5.75E-10 | 4.52E-09 | upregulated |
| AP001189.1 | 1.271739138 | 3.00E-20 | 5.62E-19 | upregulated |
| KCNK3 | 1.269114378 | 1.16E-17 | 1.81E-16 | upregulated |
| OLR1 | 1.267785847 | 1.60E-24 | 4.10E-23 | upregulated |
| PTGER3 | 1.267017098 | 8.36E-26 | 2.31E-24 | upregulated |
| RGS16 | 1.266057618 | 1.71E-28 | 5.78E-27 | upregulated |
| FGD5 | 1.266026367 | 6.41E-50 | 1.82E-47 | upregulated |
| DIO2 | 1.265450135 | 5.26E-29 | 1.86E-27 | upregulated |
| CRTAC1 | 1.26538359 | 1.08E-15 | 1.46E-14 | upregulated |
| ISM1 | 1.264463795 | 1.58E-25 | 4.29E-24 | upregulated |
| SIGLEC9 | 1.263874638 | 2.87E-39 | 2.52E-37 | upregulated |
| MMP9 | 1.262927562 | 8.84E-24 | 2.13E-22 | upregulated |
| AL592424.1 | 1.262503066 | 1.03E-07 | 5.90E-07 | upregulated |
| LILRB1 | 1.262328116 | 1.85E-27 | 5.84E-26 | upregulated |
| LILRB2 | 1.261227027 | 3.49E-28 | 1.15E-26 | upregulated |
| BHLHE22 | 1.260182212 | 4.46E-24 | 1.11E-22 | upregulated |
| LOXL1 | 1.259759093 | 2.66E-30 | 1.04E-28 | upregulated |
| PLA2G7 | 1.259486938 | 8.59E-29 | 3.00E-27 | upregulated |
| RECK | 1.259246986 | 3.83E-42 | 4.52E-40 | upregulated |
| HSPA12B | 1.256592633 | 5.45E-47 | 1.03E-44 | upregulated |
| UTS2R | 1.255000115 | 3.19E-09 | 2.30E-08 | upregulated |
| C1QC | 1.25471786 | 1.71E-29 | 6.35E-28 | upregulated |
| P2RY13 | 1.254498287 | 8.33E-21 | 1.61E-19 | upregulated |
| PCDHGA12 | 1.253538212 | 3.60E-37 | 2.49E-35 | upregulated |
| PDE1A | 1.253404724 | 1.18E-34 | 6.78E-33 | upregulated |
| SORBS1 | 1.253069223 | 1.36E-21 | 2.81E-20 | upregulated |
| DLC1 | 1.25195586 | 7.97E-40 | 7.37E-38 | upregulated |
| DYSF | 1.25152311 | 8.50E-35 | 4.93E-33 | upregulated |
| NCKAP1L | 1.251271052 | 6.23E-31 | 2.58E-29 | upregulated |
| SOD3 | 1.250950074 | 5.76E-39 | 4.89E-37 | upregulated |
| IGHV2-5 | 1.250766268 | 3.77E-05 | 0.000129176 | upregulated |
| ADAMTS14 | 1.249612698 | 7.04E-31 | 2.90E-29 | upregulated |
| DOCK2 | 1.249285054 | 1.02E-30 | 4.13E-29 | upregulated |
| PDGFD | 1.249109193 | 2.94E-25 | 7.83E-24 | upregulated |
| ACHE | 1.248967698 | 2.80E-09 | 2.02E-08 | upregulated |
| AC104083.1 | 1.248678957 | 1.90E-37 | 1.35E-35 | upregulated |
| SIGLEC14 | 1.24837121 | 3.95E-19 | 6.94E-18 | upregulated |
| SH3RF3-AS1 | 1.247687088 | 1.20E-32 | 5.76E-31 | upregulated |
| AC010175.1 | 1.247640245 | 4.41E-08 | 2.68E-07 | upregulated |
| TMEM47 | 1.245826279 | 1.27E-42 | 1.63E-40 | upregulated |
| IGLV3-21 | 1.244607733 | 1.73E-07 | 9.56E-07 | upregulated |
| MS4A2 | 1.243504651 | 4.65E-16 | 6.46E-15 | upregulated |
| TCTEX1D1 | 1.241691722 | 3.69E-26 | 1.05E-24 | upregulated |
| IGHV3OR16-10 | 1.240406394 | 0.000511536 | 0.001330982 | upregulated |
| MAPT | 1.239811412 | 1.19E-05 | 4.53E-05 | upregulated |
| PILRA | 1.239515315 | 4.59E-33 | 2.29E-31 | upregulated |
| AC021087.3 | 1.238361933 | 2.88E-08 | 1.80E-07 | upregulated |
| IGHV1-69 | 1.236642255 | 0.000277466 | 0.00077149 | upregulated |
| TMEM100 | 1.236282974 | 1.06E-25 | 2.92E-24 | upregulated |
| P2RY14 | 1.236064107 | 1.66E-24 | 4.22E-23 | upregulated |
| LRRC25 | 1.235974052 | 9.00E-32 | 3.99E-30 | upregulated |
| GPR141 | 1.235670607 | 3.68E-29 | 1.32E-27 | upregulated |
| HEYL | 1.235479123 | 2.44E-39 | 2.15E-37 | upregulated |
| TNS1 | 1.235323915 | 8.38E-34 | 4.48E-32 | upregulated |
| LDB2 | 1.235167708 | 1.07E-46 | 1.93E-44 | upregulated |
| UCP3 | 1.235102198 | 0.0007867 | 0.00194869 | upregulated |
| SYT11 | 1.235078302 | 9.35E-53 | 4.60E-50 | upregulated |
| KLHL38 | 1.234036711 | 3.42E-14 | 4.05E-13 | upregulated |
| IGSF21 | 1.233643524 | 1.63E-24 | 4.17E-23 | upregulated |
| IGHV1-2 | 1.23222965 | 1.52E-05 | 5.67E-05 | upregulated |
| CNRIP1 | 1.232094103 | 2.93E-56 | 2.46E-53 | upregulated |
| TRAV21 | 1.231547488 | 3.43E-17 | 5.17E-16 | upregulated |
| TDRD6 | 1.230292504 | 3.02E-27 | 9.34E-26 | upregulated |
| EVI2A | 1.229962383 | 1.69E-35 | 1.04E-33 | upregulated |
| FKBP7 | 1.229025739 | 1.84E-37 | 1.31E-35 | upregulated |
| LILRA1 | 1.228463518 | 2.91E-24 | 7.31E-23 | upregulated |
| AMTN | 1.227951524 | 0.000512055 | 0.001331907 | upregulated |
| SLCO5A1 | 1.226781621 | 3.54E-18 | 5.74E-17 | upregulated |
| PTGDR | 1.225957053 | 5.87E-37 | 3.92E-35 | upregulated |
| PLPPR4 | 1.225475814 | 2.10E-32 | 9.91E-31 | upregulated |
| CHRD | 1.224436741 | 4.81E-46 | 8.02E-44 | upregulated |
| IGKV1D-8 | 1.22421019 | 0.000128964 | 0.000390141 | upregulated |
| IGHV3-20 | 1.223590316 | 3.03E-07 | 1.61E-06 | upregulated |
| KLHL31 | 1.222858554 | 0.001591617 | 0.003622338 | upregulated |
| MMP13 | 1.22235505 | 5.49E-19 | 9.52E-18 | upregulated |
| CCR4 | 1.221746933 | 1.36E-23 | 3.24E-22 | upregulated |
| HIC1 | 1.220846061 | 1.47E-43 | 2.08E-41 | upregulated |
| TMEM200A | 1.219696495 | 9.63E-33 | 4.66E-31 | upregulated |
| SIGLEC12 | 1.218795385 | 3.19E-10 | 2.58E-09 | upregulated |
| AC131097.4 | 1.218321561 | 1.99E-17 | 3.05E-16 | upregulated |
| IGHV4-4 | 1.217855336 | 0.000434312 | 0.001151584 | upregulated |
| IGHV1-46 | 1.217478781 | 2.45E-06 | 1.09E-05 | upregulated |
| IGF1 | 1.216574002 | 3.91E-26 | 1.11E-24 | upregulated |
| NNMT | 1.216479856 | 2.38E-34 | 1.34E-32 | upregulated |
| IBSP | 1.216136269 | 3.96E-11 | 3.51E-10 | upregulated |
| TSPAN8 | 1.215582117 | 0.004972752 | 0.009948463 | upregulated |
| KLHL34 | 1.212654154 | 3.13E-14 | 3.72E-13 | upregulated |
| SLC14A1 | 1.212196719 | 2.12E-09 | 1.56E-08 | upregulated |
| WDFY4 | 1.211238434 | 4.30E-25 | 1.14E-23 | upregulated |
| PTCRA | 1.210927288 | 7.82E-18 | 1.23E-16 | upregulated |
| ITGA7 | 1.210584903 | 3.48E-18 | 5.66E-17 | upregulated |
| IGHV2-70 | 1.210220384 | 4.71E-06 | 1.97E-05 | upregulated |
| AC004988.1 | 1.210012396 | 1.46E-15 | 1.94E-14 | upregulated |
| PTCH2 | 1.209661081 | 0.002371376 | 0.005163443 | upregulated |
| WIPF1 | 1.208848188 | 1.04E-44 | 1.62E-42 | upregulated |
| SIGLEC10 | 1.208738768 | 4.95E-25 | 1.30E-23 | upregulated |
| CD248 | 1.207477462 | 2.11E-39 | 1.88E-37 | upregulated |
| LMCD1 | 1.207159957 | 1.37E-35 | 8.42E-34 | upregulated |
| SHD | 1.207128451 | 4.79E-11 | 4.22E-10 | upregulated |
| LANCL1-AS1 | 1.207095853 | 3.01E-05 | 0.000105141 | upregulated |
| C1QB | 1.204742424 | 6.07E-26 | 1.70E-24 | upregulated |
| SMOC2 | 1.204359364 | 1.66E-26 | 4.85E-25 | upregulated |
| COL8A2 | 1.203749271 | 6.11E-26 | 1.71E-24 | upregulated |
| EMX2 | 1.20310599 | 4.84E-17 | 7.20E-16 | upregulated |
| CLEC10A | 1.203025678 | 5.23E-18 | 8.36E-17 | upregulated |
| RFLNA | 1.202889841 | 1.21E-41 | 1.35E-39 | upregulated |
| SLC8A1 | 1.201353204 | 8.25E-32 | 3.70E-30 | upregulated |
| AC079780.1 | 1.200897897 | 2.86E-07 | 1.53E-06 | upregulated |
| ACTA2-AS1 | 1.19967928 | 5.84E-19 | 1.01E-17 | upregulated |
| LINC01563 | 1.199164173 | 2.38E-06 | 1.06E-05 | upregulated |
| ARHGEF6 | 1.198236767 | 1.77E-37 | 1.27E-35 | upregulated |
| CAMK2B | 1.197562008 | 0.000268875 | 0.000750707 | upregulated |
| GAS1 | 1.19599501 | 5.37E-38 | 4.04E-36 | upregulated |
| F2R | 1.195229747 | 7.93E-41 | 8.16E-39 | upregulated |
| KCNA3 | 1.193897841 | 2.03E-18 | 3.37E-17 | upregulated |
| LINC01150 | 1.193359566 | 1.67E-24 | 4.26E-23 | upregulated |
| ADA2 | 1.192797432 | 2.98E-24 | 7.48E-23 | upregulated |
| AL133371.2 | 1.190713236 | 4.48E-23 | 1.02E-21 | upregulated |
| CDH5 | 1.190446759 | 1.81E-39 | 1.61E-37 | upregulated |
| SLC40A1 | 1.190361146 | 7.75E-27 | 2.32E-25 | upregulated |
| MCEMP1 | 1.188553809 | 2.88E-14 | 3.43E-13 | upregulated |
| THPO | 1.188459338 | 1.57E-32 | 7.49E-31 | upregulated |
| PTPRC | 1.187967099 | 8.64E-26 | 2.38E-24 | upregulated |
| ENPEP | 1.185915242 | 1.29E-36 | 8.30E-35 | upregulated |
| MAMDC2 | 1.18563286 | 2.46E-16 | 3.48E-15 | upregulated |
| EDNRA | 1.185267967 | 1.20E-35 | 7.43E-34 | upregulated |
| IGHV3-76 | 1.185100578 | 0.001458602 | 0.003355949 | upregulated |
| LINC02257 | 1.184705177 | 2.90E-19 | 5.15E-18 | upregulated |
| S1PR1 | 1.184645554 | 9.12E-41 | 9.25E-39 | upregulated |
| PLEK | 1.184587746 | 1.72E-25 | 4.64E-24 | upregulated |
| ABCB4 | 1.184108964 | 1.12E-33 | 5.93E-32 | upregulated |
| IGKV6-21 | 1.183222758 | 1.38E-05 | 5.21E-05 | upregulated |
| AC106872.5 | 1.183121424 | 2.35E-33 | 1.20E-31 | upregulated |
| IRF8 | 1.18067566 | 1.38E-26 | 4.07E-25 | upregulated |
| TSPAN2 | 1.179892491 | 3.43E-25 | 9.11E-24 | upregulated |
| RASGRP4 | 1.179270897 | 4.79E-39 | 4.11E-37 | upregulated |
| EDA2R | 1.178907345 | 1.79E-35 | 1.09E-33 | upregulated |
| PLA2G5 | 1.178068233 | 1.07E-21 | 2.23E-20 | upregulated |
| IGHV3-65 | 1.178053552 | 0.00035366 | 0.000957615 | upregulated |
| ADAM33 | 1.17741392 | 1.73E-25 | 4.66E-24 | upregulated |
| ADCYAP1 | 1.177338928 | 2.15E-08 | 1.37E-07 | upregulated |
| TWIST1 | 1.176555027 | 8.11E-28 | 2.65E-26 | upregulated |
| FAM19A5 | 1.176238225 | 1.77E-33 | 9.17E-32 | upregulated |
| EMCN | 1.176210121 | 2.16E-31 | 9.27E-30 | upregulated |
| AC009093.2 | 1.176027428 | 2.14E-28 | 7.18E-27 | upregulated |
| IGKV1OR2-3 | 1.175650913 | 0.014683348 | 0.025618729 | upregulated |
| FCGR1A | 1.174936557 | 2.20E-29 | 8.07E-28 | upregulated |
| AC093627.6 | 1.174766015 | 1.92E-10 | 1.58E-09 | upregulated |
| LRRN4CL | 1.174536509 | 8.09E-21 | 1.57E-19 | upregulated |
| AL009178.2 | 1.174207743 | 2.83E-24 | 7.13E-23 | upregulated |
| CD80 | 1.174141735 | 6.19E-26 | 1.73E-24 | upregulated |
| LYVE1 | 1.17356876 | 1.84E-26 | 5.33E-25 | upregulated |
| CTSS | 1.172185659 | 1.14E-24 | 2.93E-23 | upregulated |
| ALPK2 | 1.17196914 | 4.80E-34 | 2.62E-32 | upregulated |
| TCL1A | 1.171727197 | 5.79E-08 | 3.45E-07 | upregulated |
| NKAPL | 1.171726304 | 1.69E-30 | 6.73E-29 | upregulated |
| VIM | 1.170750654 | 5.03E-48 | 1.06E-45 | upregulated |
| ACKR1 | 1.170749296 | 1.32E-11 | 1.23E-10 | upregulated |
| TIE1 | 1.17050713 | 4.20E-42 | 4.90E-40 | upregulated |
| IGSF6 | 1.170386638 | 1.47E-29 | 5.48E-28 | upregulated |
| TMEM59L | 1.17021684 | 1.43E-15 | 1.90E-14 | upregulated |
| IL2RA | 1.169753865 | 1.46E-31 | 6.35E-30 | upregulated |
| ITGA4 | 1.169746768 | 7.85E-29 | 2.76E-27 | upregulated |
| LYZ | 1.168983914 | 6.15E-24 | 1.50E-22 | upregulated |
| FERMT2 | 1.167795737 | 5.84E-34 | 3.16E-32 | upregulated |
| TIMP3 | 1.167035042 | 2.64E-24 | 6.69E-23 | upregulated |
| ADAMTS10 | 1.165515508 | 5.11E-35 | 3.01E-33 | upregulated |
| CYP1B1 | 1.165197891 | 1.55E-23 | 3.67E-22 | upregulated |
| IGLV3-13 | 1.164257581 | 0.000335213 | 0.000912421 | upregulated |
| RASL12 | 1.164046297 | 5.98E-42 | 6.78E-40 | upregulated |
| AL049838.1 | 1.163732336 | 1.01E-15 | 1.36E-14 | upregulated |
| IL10 | 1.163046913 | 4.11E-27 | 1.26E-25 | upregulated |
| GPRIN3 | 1.162305909 | 1.83E-31 | 7.88E-30 | upregulated |
| IGHV1OR16-1 | 1.16022588 | 0.000471586 | 0.001238374 | upregulated |
| PDE4B | 1.159732262 | 2.17E-32 | 1.02E-30 | upregulated |
| DGKI | 1.159508708 | 3.87E-23 | 8.88E-22 | upregulated |
| TNFSF8 | 1.159422751 | 1.47E-26 | 4.33E-25 | upregulated |
| SH3BGR | 1.159132264 | 1.01E-07 | 5.78E-07 | upregulated |
| IGHV7-56 | 1.158325327 | 0.000994416 | 0.002391291 | upregulated |
| PECAM1 | 1.157566987 | 1.65E-42 | 2.06E-40 | upregulated |
| CXCL9 | 1.156624734 | 1.83E-11 | 1.68E-10 | upregulated |
| CALD1 | 1.155689623 | 3.93E-43 | 5.28E-41 | upregulated |
| ANXA6 | 1.155548592 | 1.14E-49 | 3.03E-47 | upregulated |
| GSC | 1.1554928 | 1.69E-27 | 5.37E-26 | upregulated |
| CD53 | 1.154937902 | 1.54E-29 | 5.73E-28 | upregulated |
| SEC14L6 | 1.154245345 | 1.96E-21 | 3.97E-20 | upregulated |
| FGR | 1.151356169 | 3.08E-29 | 1.12E-27 | upregulated |
| GIMAP6 | 1.150626298 | 5.48E-31 | 2.28E-29 | upregulated |
| GPC4 | 1.14993656 | 1.31E-15 | 1.75E-14 | upregulated |
| SCIMP | 1.149556928 | 1.75E-23 | 4.13E-22 | upregulated |
| ST6GALNAC5 | 1.149167047 | 1.01E-18 | 1.71E-17 | upregulated |
| AC092376.2 | 1.148645662 | 2.68E-24 | 6.76E-23 | upregulated |
| SALL1 | 1.147938375 | 5.89E-18 | 9.37E-17 | upregulated |
| CDH19 | 1.147830253 | 8.39E-13 | 8.82E-12 | upregulated |
| AC011899.2 | 1.147601122 | 6.17E-20 | 1.14E-18 | upregulated |
| IGHV3-71 | 1.147445112 | 7.35E-05 | 0.00023623 | upregulated |
| AC093535.2 | 1.145888411 | 7.71E-25 | 2.01E-23 | upregulated |
| SLITRK4 | 1.145852626 | 2.89E-25 | 7.71E-24 | upregulated |
| SHROOM4 | 1.145568807 | 5.10E-31 | 2.13E-29 | upregulated |
| CLEC4D | 1.144583251 | 2.40E-15 | 3.11E-14 | upregulated |
| CXorf21 | 1.144228807 | 4.05E-26 | 1.15E-24 | upregulated |
| ITGAX | 1.14266922 | 1.86E-23 | 4.38E-22 | upregulated |
| CPA3 | 1.141445398 | 1.76E-17 | 2.71E-16 | upregulated |
| IGKV1-33 | 1.140146213 | 7.97E-05 | 0.000254037 | upregulated |
| TRABD2B | 1.139313373 | 1.35E-24 | 3.48E-23 | upregulated |
| AC109479.1 | 1.139152286 | 1.43E-16 | 2.06E-15 | upregulated |
| CHN1 | 1.139006573 | 2.38E-40 | 2.29E-38 | upregulated |
| CD28 | 1.137621139 | 8.91E-28 | 2.91E-26 | upregulated |
| LAPTM5 | 1.137026995 | 9.35E-33 | 4.54E-31 | upregulated |
| CXCR2P1 | 1.136445017 | 1.12E-07 | 6.36E-07 | upregulated |
| KCNN3 | 1.13495458 | 5.20E-27 | 1.57E-25 | upregulated |
| ADGRD1 | 1.134477945 | 1.65E-30 | 6.58E-29 | upregulated |
| CLEC4E | 1.134158641 | 1.29E-17 | 2.01E-16 | upregulated |
| IGKV2-18 | 1.133158961 | 0.01031636 | 0.018851975 | upregulated |
| TMEM273 | 1.132899021 | 1.87E-34 | 1.06E-32 | upregulated |
| CSMD2 | 1.131984738 | 2.42E-27 | 7.55E-26 | upregulated |
| ITGA8 | 1.131601227 | 2.52E-27 | 7.85E-26 | upregulated |
| CLEC4A | 1.131585352 | 5.57E-32 | 2.52E-30 | upregulated |
| FCGR1CP | 1.130934056 | 6.07E-19 | 1.05E-17 | upregulated |
| IGKV1OR2-11 | 1.130753998 | 0.003124779 | 0.006578174 | upregulated |
| CNTN4 | 1.127943244 | 2.02E-27 | 6.33E-26 | upregulated |
| PDE1B | 1.127500363 | 1.98E-37 | 1.40E-35 | upregulated |
| FAM131B | 1.127077808 | 2.24E-20 | 4.22E-19 | upregulated |
| MYOM3 | 1.127076463 | 3.56E-08 | 2.19E-07 | upregulated |
| F10 | 1.126329873 | 4.94E-23 | 1.13E-21 | upregulated |
| RAB3IL1 | 1.125690642 | 3.72E-38 | 2.87E-36 | upregulated |
| TIFAB | 1.125639349 | 2.71E-15 | 3.50E-14 | upregulated |
| GPR183 | 1.125145664 | 4.64E-30 | 1.77E-28 | upregulated |
| AVPR1A | 1.124940933 | 8.00E-24 | 1.94E-22 | upregulated |
| AQP1 | 1.123753891 | 5.15E-36 | 3.26E-34 | upregulated |
| IGHV3OR16-15 | 1.12308862 | 0.000552374 | 0.001424747 | upregulated |
| VASH1 | 1.122771747 | 1.14E-42 | 1.47E-40 | upregulated |
| AC134879.2 | 1.122348181 | 0.000313877 | 0.00086005 | upregulated |
| MITF | 1.122164746 | 5.50E-34 | 2.98E-32 | upregulated |
| ZFHX4-AS1 | 1.121824829 | 1.45E-06 | 6.73E-06 | upregulated |
| CD300LF | 1.120207394 | 6.65E-27 | 2.00E-25 | upregulated |
| CPQ | 1.119276534 | 1.27E-33 | 6.70E-32 | upregulated |
| ITGA1 | 1.11892586 | 1.59E-46 | 2.82E-44 | upregulated |
| IGHV1-68 | 1.118147055 | 0.000790193 | 0.001956183 | upregulated |
| PXDNL | 1.117278935 | 9.72E-18 | 1.52E-16 | upregulated |
| AC109826.1 | 1.11694737 | 3.35E-23 | 7.76E-22 | upregulated |
| TRBV24-1 | 1.116654616 | 3.25E-13 | 3.53E-12 | upregulated |
| RAPSN | 1.116213074 | 0.000326742 | 0.000891548 | upregulated |
| SERPING1 | 1.116034331 | 1.15E-34 | 6.60E-33 | upregulated |
| LILRB3 | 1.115343327 | 3.64E-29 | 1.31E-27 | upregulated |
| TYROBP | 1.114767339 | 4.20E-31 | 1.76E-29 | upregulated |
| PCDH17 | 1.1146877 | 5.76E-31 | 2.39E-29 | upregulated |
| LBX1 | 1.112295208 | 8.29E-05 | 0.000263445 | upregulated |
| CCRL2 | 1.111864982 | 7.49E-33 | 3.67E-31 | upregulated |
| CACNB1 | 1.111161889 | 1.00E-06 | 4.82E-06 | upregulated |
| SH3RF3 | 1.110844926 | 1.15E-38 | 9.23E-37 | upregulated |
| DSEL | 1.109623796 | 8.70E-26 | 2.40E-24 | upregulated |
| ADGRF5 | 1.108685149 | 3.78E-37 | 2.59E-35 | upregulated |
| SLIT3 | 1.108634436 | 1.51E-26 | 4.42E-25 | upregulated |
| C16orf45 | 1.10788198 | 9.90E-39 | 8.02E-37 | upregulated |
| ARHGAP31 | 1.107550003 | 6.90E-40 | 6.42E-38 | upregulated |
| LOXL2 | 1.106479343 | 2.26E-27 | 7.07E-26 | upregulated |
| FCGR1B | 1.105998744 | 1.51E-22 | 3.31E-21 | upregulated |
| ARSE | 1.105990773 | 8.40E-24 | 2.04E-22 | upregulated |
| PCDH12 | 1.105514392 | 4.98E-40 | 4.69E-38 | upregulated |
| PAPPA | 1.105380842 | 2.10E-20 | 3.98E-19 | upregulated |
| IGHV3OR16-16 | 1.105307597 | 0.000203134 | 0.000585466 | upregulated |
| GRID1 | 1.105082619 | 2.13E-30 | 8.43E-29 | upregulated |
| EDIL3 | 1.10465848 | 6.64E-21 | 1.30E-19 | upregulated |
| MRVI1 | 1.104527877 | 8.22E-39 | 6.72E-37 | upregulated |
| HLA-DQA1 | 1.104259715 | 5.59E-24 | 1.37E-22 | upregulated |
| ACE | 1.104236654 | 2.90E-38 | 2.26E-36 | upregulated |
| FCGR2B | 1.104082163 | 5.87E-29 | 2.07E-27 | upregulated |
| TRAV35 | 1.103879131 | 2.50E-11 | 2.27E-10 | upregulated |
| LCP2 | 1.102699805 | 6.56E-29 | 2.31E-27 | upregulated |
| IGKV1D-42 | 1.102457007 | 0.00010659 | 0.000328782 | upregulated |
| CLEC5A | 1.102176332 | 9.28E-27 | 2.76E-25 | upregulated |
| IGHV1-17 | 1.101632538 | 3.46E-05 | 0.000119468 | upregulated |
| ACVRL1 | 1.101249557 | 2.14E-52 | 9.40E-50 | upregulated |
| NFAM1 | 1.100051306 | 1.43E-29 | 5.35E-28 | upregulated |
| LY86 | 1.099870159 | 6.14E-28 | 2.02E-26 | upregulated |
| LTBP2 | 1.098303795 | 2.16E-31 | 9.27E-30 | upregulated |
| RNASE6 | 1.098165736 | 3.30E-30 | 1.28E-28 | upregulated |
| ADGRL4 | 1.097953209 | 3.34E-43 | 4.52E-41 | upregulated |
| CD300E | 1.097717945 | 2.34E-13 | 2.57E-12 | upregulated |
| STARD13 | 1.094015192 | 6.25E-38 | 4.65E-36 | upregulated |
| SIGLEC6 | 1.09391468 | 1.01E-16 | 1.47E-15 | upregulated |
| LINC00924 | 1.093407031 | 8.25E-21 | 1.60E-19 | upregulated |
| SPI1 | 1.093120509 | 2.57E-31 | 1.10E-29 | upregulated |
| IQGAP2 | 1.09000076 | 3.04E-27 | 9.37E-26 | upregulated |
| TMEM150B | 1.089740463 | 5.13E-23 | 1.17E-21 | upregulated |
| PLA1A | 1.08947558 | 3.72E-19 | 6.55E-18 | upregulated |
| IL10RA | 1.089439924 | 4.37E-27 | 1.33E-25 | upregulated |
| STAB1 | 1.089330812 | 3.12E-32 | 1.45E-30 | upregulated |
| IGHV2-70D | 1.088842698 | 0.000350572 | 0.000950207 | upregulated |
| FAM189A1 | 1.088802271 | 4.56E-20 | 8.46E-19 | upregulated |
| S1PR3 | 1.08871769 | 1.51E-28 | 5.13E-27 | upregulated |
| SRGN | 1.088336959 | 2.31E-29 | 8.46E-28 | upregulated |
| TRAV9-2 | 1.088250808 | 5.56E-17 | 8.25E-16 | upregulated |
| MARCO | 1.088248988 | 4.07E-08 | 2.48E-07 | upregulated |
| CYSLTR1 | 1.086935943 | 1.57E-26 | 4.59E-25 | upregulated |
| CLEC3B | 1.086175942 | 2.74E-22 | 5.91E-21 | upregulated |
| AC134043.2 | 1.085771934 | 4.73E-37 | 3.22E-35 | upregulated |
| SSPN | 1.084727018 | 8.20E-30 | 3.11E-28 | upregulated |
| PLD4 | 1.084723425 | 1.25E-17 | 1.94E-16 | upregulated |
| IGHE | 1.084697073 | 0.008809341 | 0.016416224 | upregulated |
| CILP2 | 1.084480541 | 1.02E-09 | 7.78E-09 | upregulated |
| RCSD1 | 1.082450556 | 3.69E-29 | 1.32E-27 | upregulated |
| CRABP1 | 1.082043043 | 0.010174374 | 0.018626248 | upregulated |
| LDLRAD4 | 1.081596002 | 1.75E-29 | 6.48E-28 | upregulated |
| SELE | 1.081224757 | 1.78E-15 | 2.34E-14 | upregulated |
| ALOX5 | 1.081089116 | 1.63E-27 | 5.16E-26 | upregulated |
| AC108463.2 | 1.081015383 | 7.25E-27 | 2.18E-25 | upregulated |
| ST8SIA2 | 1.080474122 | 3.91E-23 | 8.98E-22 | upregulated |
| TRAV8-1 | 1.080217546 | 3.33E-13 | 3.61E-12 | upregulated |
| TACR1 | 1.079502562 | 5.11E-08 | 3.07E-07 | upregulated |
| IGHV3-66 | 1.07898699 | 3.62E-05 | 0.000124381 | upregulated |
| AOAH | 1.078434872 | 1.42E-24 | 3.65E-23 | upregulated |
| AC245128.3 | 1.077687002 | 9.66E-09 | 6.48E-08 | upregulated |
| APOE | 1.077061812 | 9.14E-21 | 1.77E-19 | upregulated |
| SLC18A2 | 1.076243197 | 1.66E-16 | 2.39E-15 | upregulated |
| TRAV20 | 1.075399333 | 2.98E-14 | 3.55E-13 | upregulated |
| COX7A1 | 1.075257052 | 4.43E-24 | 1.10E-22 | upregulated |
| IL17D | 1.074825603 | 1.16E-10 | 9.83E-10 | upregulated |
| MRC2 | 1.073450804 | 2.16E-29 | 7.91E-28 | upregulated |
| RPL3L | 1.073224466 | 0.001835749 | 0.004116665 | upregulated |
| DIRAS1 | 1.073028849 | 1.38E-15 | 1.83E-14 | upregulated |
| KDR | 1.072685539 | 1.26E-28 | 4.31E-27 | upregulated |
| TYRP1 | 1.072223873 | 4.57E-10 | 3.63E-09 | upregulated |
| GUCA1A | 1.071810894 | 4.34E-27 | 1.32E-25 | upregulated |
| FBP1 | 1.071644302 | 1.90E-12 | 1.93E-11 | upregulated |
| IGHV3-11 | 1.071599817 | 2.36E-06 | 1.05E-05 | upregulated |
| CSF3R | 1.070876999 | 5.74E-22 | 1.22E-20 | upregulated |
| AFF3 | 1.070577201 | 5.01E-16 | 6.95E-15 | upregulated |
| TRAV14DV4 | 1.068992269 | 5.01E-14 | 5.82E-13 | upregulated |
| ADAMTS6 | 1.067275788 | 4.95E-21 | 9.76E-20 | upregulated |
| HAVCR2 | 1.066960051 | 4.23E-30 | 1.63E-28 | upregulated |
| EVI2B | 1.064736729 | 2.92E-27 | 9.04E-26 | upregulated |
| FCER1G | 1.064629454 | 4.38E-31 | 1.84E-29 | upregulated |
| TNNT1 | 1.064095999 | 0.008536711 | 0.015958287 | upregulated |
| IGKV1OR2-6 | 1.063911238 | 0.000878872 | 0.002144486 | upregulated |
| MMP2-AS1 | 1.062316674 | 1.74E-07 | 9.62E-07 | upregulated |
| FERMT3 | 1.06191686 | 1.55E-26 | 4.53E-25 | upregulated |
| TNFSF13B | 1.061600404 | 1.38E-23 | 3.29E-22 | upregulated |
| BMP5 | 1.059976252 | 1.44E-19 | 2.60E-18 | upregulated |
| SMIM25 | 1.059553058 | 1.18E-26 | 3.48E-25 | upregulated |
| PCDHB5 | 1.059516764 | 2.98E-23 | 6.94E-22 | upregulated |
| INMT | 1.058651115 | 1.88E-16 | 2.69E-15 | upregulated |
| AL590648.3 | 1.05837505 | 2.83E-15 | 3.66E-14 | upregulated |
| SAMD11 | 1.058060749 | 1.27E-11 | 1.18E-10 | upregulated |
| TNS3 | 1.056943505 | 9.41E-37 | 6.14E-35 | upregulated |
| AP001528.2 | 1.056095813 | 1.26E-19 | 2.29E-18 | upregulated |
| BIRC7 | 1.05573175 | 1.56E-06 | 7.20E-06 | upregulated |
| ARHGAP20 | 1.05540823 | 5.08E-19 | 8.85E-18 | upregulated |
| SYNC | 1.053674688 | 7.32E-19 | 1.26E-17 | upregulated |
| JAM3 | 1.053496137 | 2.89E-40 | 2.77E-38 | upregulated |
| SRPX | 1.053371567 | 7.53E-18 | 1.19E-16 | upregulated |
| AL133415.1 | 1.052452225 | 1.55E-22 | 3.40E-21 | upregulated |
| GIMAP8 | 1.052264783 | 3.04E-27 | 9.37E-26 | upregulated |
| IGLV3-22 | 1.052218621 | 0.0281087 | 0.045432341 | upregulated |
| ENOX1 | 1.051809375 | 2.76E-34 | 1.54E-32 | upregulated |
| SOX17 | 1.051709066 | 1.07E-27 | 3.44E-26 | upregulated |
| NRROS | 1.051704005 | 1.22E-28 | 4.20E-27 | upregulated |
| MEIS3P2 | 1.050858304 | 1.24E-21 | 2.56E-20 | upregulated |
| C1QA | 1.05067249 | 9.89E-24 | 2.38E-22 | upregulated |
| SPN | 1.049414909 | 4.35E-21 | 8.66E-20 | upregulated |
| AC098613.1 | 1.049038832 | 1.14E-24 | 2.93E-23 | upregulated |
| TRAV2 | 1.048807746 | 8.66E-19 | 1.47E-17 | upregulated |
| MYH11 | 1.048160965 | 8.27E-16 | 1.12E-14 | upregulated |
| HMCN2 | 1.048020343 | 4.25E-10 | 3.38E-09 | upregulated |
| CD14 | 1.047817856 | 1.99E-30 | 7.92E-29 | upregulated |
| ITM2A | 1.047294919 | 4.80E-26 | 1.35E-24 | upregulated |
| NTNG2 | 1.046900988 | 4.03E-38 | 3.07E-36 | upregulated |
| IGHV3OR16-6 | 1.046790124 | 2.77E-05 | 9.80E-05 | upregulated |
| CHST13 | 1.045377144 | 3.36E-21 | 6.73E-20 | upregulated |
| NFATC4 | 1.045374864 | 1.46E-33 | 7.59E-32 | upregulated |
| FAM110B | 1.045369862 | 1.95E-28 | 6.54E-27 | upregulated |
| GIMAP4 | 1.044934432 | 1.16E-30 | 4.68E-29 | upregulated |
| CD34 | 1.044637854 | 6.08E-41 | 6.36E-39 | upregulated |
| TMEM52B | 1.042315034 | 2.21E-10 | 1.82E-09 | upregulated |
| CCL19 | 1.042248009 | 3.55E-14 | 4.20E-13 | upregulated |
| AC083949.1 | 1.041405734 | 3.80E-14 | 4.48E-13 | upregulated |
| IGHV3-23 | 1.041235129 | 1.43E-07 | 8.01E-07 | upregulated |
| PIK3R5 | 1.040495231 | 6.66E-28 | 2.18E-26 | upregulated |
| LHFPL6 | 1.0397214 | 7.06E-41 | 7.34E-39 | upregulated |
| TRBV3-1 | 1.039412913 | 5.77E-16 | 7.93E-15 | upregulated |
| MYCT1 | 1.03916621 | 7.53E-36 | 4.70E-34 | upregulated |
| PRKCB | 1.039158956 | 1.69E-23 | 3.99E-22 | upregulated |
| TIMD4 | 1.03856654 | 1.09E-14 | 1.33E-13 | upregulated |
| BRINP1 | 1.038497063 | 5.38E-14 | 6.23E-13 | upregulated |
| GPR65 | 1.038014291 | 9.54E-28 | 3.09E-26 | upregulated |
| KCNMB1 | 1.037476605 | 5.06E-32 | 2.30E-30 | upregulated |
| MMP7 | 1.036565187 | 7.90E-13 | 8.33E-12 | upregulated |
| RF00397 | 1.036332749 | 6.55E-07 | 3.26E-06 | upregulated |
| PLXDC2 | 1.035710899 | 4.83E-26 | 1.36E-24 | upregulated |
| IGKV1OR22-1 | 1.034744856 | 9.59E-05 | 0.000299253 | upregulated |
| AF127936.1 | 1.034642093 | 3.29E-13 | 3.56E-12 | upregulated |
| SIRPB2 | 1.034611017 | 2.60E-26 | 7.52E-25 | upregulated |
| AL109741.1 | 1.034282554 | 1.54E-20 | 2.94E-19 | upregulated |
| TRAV24 | 1.033135822 | 4.87E-11 | 4.29E-10 | upregulated |
| ZBTB16 | 1.033004721 | 7.46E-13 | 7.89E-12 | upregulated |
| ACTN3 | 1.031967715 | 3.64E-07 | 1.91E-06 | upregulated |
| IGFBP5 | 1.031914781 | 5.15E-30 | 1.97E-28 | upregulated |
| VENTX | 1.030883484 | 1.95E-21 | 3.96E-20 | upregulated |
| ADORA1 | 1.030339164 | 1.63E-30 | 6.54E-29 | upregulated |
| RNASE1 | 1.030291576 | 2.61E-31 | 1.11E-29 | upregulated |
| CXorf36 | 1.029514322 | 3.84E-38 | 2.94E-36 | upregulated |
| RPLP0P2 | 1.028524311 | 3.40E-21 | 6.81E-20 | upregulated |
| LINC01655 | 1.028039077 | 3.41E-11 | 3.04E-10 | upregulated |
| BTK | 1.027606555 | 3.55E-27 | 1.09E-25 | upregulated |
| VSTM4 | 1.027335372 | 1.08E-35 | 6.75E-34 | upregulated |
| MMP19 | 1.027238455 | 9.93E-35 | 5.73E-33 | upregulated |
| ST6GALNAC3 | 1.025576661 | 3.64E-32 | 1.67E-30 | upregulated |
| GJA4 | 1.024413104 | 3.77E-31 | 1.59E-29 | upregulated |
| ITGA9 | 1.024402359 | 4.75E-37 | 3.23E-35 | upregulated |
| TRBV5-1 | 1.023083132 | 3.85E-17 | 5.80E-16 | upregulated |
| TCF4 | 1.021263274 | 2.13E-35 | 1.29E-33 | upregulated |
| ADAMTS9 | 1.020081402 | 9.24E-34 | 4.93E-32 | upregulated |
| SELL | 1.019516539 | 4.87E-21 | 9.61E-20 | upregulated |
| FOXS1 | 1.019299283 | 1.60E-23 | 3.78E-22 | upregulated |
| AC004921.1 | 1.018418588 | 4.85E-14 | 5.65E-13 | upregulated |
| FLI1 | 1.018348807 | 1.38E-37 | 1.01E-35 | upregulated |
| PREX1 | 1.016383446 | 8.31E-32 | 3.72E-30 | upregulated |
| CYR61 | 1.016002222 | 5.46E-19 | 9.48E-18 | upregulated |
| TBXAS1 | 1.015501278 | 2.67E-30 | 1.05E-28 | upregulated |
| RYR1 | 1.015449935 | 0.003071184 | 0.00647582 | upregulated |
| CARNS1 | 1.014819124 | 3.81E-07 | 1.99E-06 | upregulated |
| SDS | 1.014151881 | 4.31E-18 | 6.94E-17 | upregulated |
| P2RY10 | 1.013450896 | 5.93E-20 | 1.10E-18 | upregulated |
| AC008957.1 | 1.013434508 | 1.66E-16 | 2.38E-15 | upregulated |
| SLC2A5 | 1.013053686 | 1.22E-16 | 1.77E-15 | upregulated |
| TRAV5 | 1.012215503 | 2.31E-14 | 2.78E-13 | upregulated |
| CD180 | 1.012136302 | 5.69E-23 | 1.29E-21 | upregulated |
| AGT | 1.011251953 | 7.59E-23 | 1.71E-21 | upregulated |
| CDH6 | 1.009879552 | 1.38E-28 | 4.73E-27 | upregulated |
| IGHV3-75 | 1.008681249 | 0.000200024 | 0.000577409 | upregulated |
| GIMAP5 | 1.008271186 | 1.26E-21 | 2.60E-20 | upregulated |
| TRAV26-1 | 1.008180477 | 4.37E-16 | 6.09E-15 | upregulated |
| EFHD1 | 1.007906738 | 2.93E-24 | 7.35E-23 | upregulated |
| ARHGEF15 | 1.007770682 | 8.31E-33 | 4.05E-31 | upregulated |
| RASSF4 | 1.0071175 | 5.73E-30 | 2.18E-28 | upregulated |
| LOXL3 | 1.006733653 | 3.69E-37 | 2.54E-35 | upregulated |
| ENG | 1.006581482 | 7.17E-47 | 1.32E-44 | upregulated |
| P2RY8 | 1.006283162 | 2.89E-25 | 7.70E-24 | upregulated |
| IGHV4-61 | 1.005995362 | 3.75E-06 | 1.60E-05 | upregulated |
| CPXM2 | 1.005230276 | 8.98E-28 | 2.92E-26 | upregulated |
| SNAI1 | 1.004706403 | 2.24E-31 | 9.59E-30 | upregulated |
| PTPRB | 1.004130757 | 2.93E-31 | 1.24E-29 | upregulated |
| LINC02285 | 1.002607771 | 2.64E-16 | 3.74E-15 | upregulated |
| IGLV3-10 | 1.001928526 | 0.000308586 | 0.000846703 | upregulated |
| SLIT2 | 1.000667293 | 1.40E-20 | 2.68E-19 | upregulated |
| PMCH | 1.000533918 | 0.00946153 | 0.01748784 | upregulated |
| SLC6A1 | 1.000438674 | 5.52E-15 | 6.95E-14 | upregulated |
| TMEM204 | 1.000094675 | 4.26E-38 | 3.23E-36 | upregulated |
| KRT32 | -1.003346478 | 0.003780163 | 0.007784073 | downregulated |
| AC005077.4 | -1.003438174 | 0.008739667 | 0.016295521 | downregulated |
| AL138789.1 | -1.00623099 | 0.000182124 | 0.000530978 | downregulated |
| PI3 | -1.00869011 | 4.87E-06 | 2.03E-05 | downregulated |
| AC078881.1 | -1.010430497 | 2.41E-05 | 8.65E-05 | downregulated |
| MUC4 | -1.010818206 | 0.002871806 | 0.006100359 | downregulated |
| TMPRSS11B | -1.019278348 | 0.000174294 | 0.000510289 | downregulated |
| SLC13A4 | -1.019354754 | 4.17E-06 | 1.76E-05 | downregulated |
| ZYG11A | -1.020180188 | 3.85E-05 | 0.000131407 | downregulated |
| AL136982.5 | -1.022729658 | 0.000510899 | 0.001329541 | downregulated |
| TMPRSS11BNL | -1.023338077 | 0.000424143 | 0.001126842 | downregulated |
| AC083801.2 | -1.023987047 | 7.66E-07 | 3.77E-06 | downregulated |
| TMPRSS11GP | -1.025922294 | 5.28E-07 | 2.68E-06 | downregulated |
| CDKN2A | -1.027768583 | 0.011117088 | 0.020089611 | downregulated |
| AL365226.2 | -1.028303906 | 0.002474671 | 0.005361002 | downregulated |
| AL391427.1 | -1.028594865 | 0.000681442 | 0.001712346 | downregulated |
| S100A12 | -1.030169527 | 0.000108547 | 0.000334289 | downregulated |
| OR2B6 | -1.031842057 | 0.0002716 | 0.000757164 | downregulated |
| CYP4F29P | -1.033747326 | 0.016930529 | 0.028956113 | downregulated |
| CEACAM7 | -1.034707545 | 0.016639575 | 0.028528622 | downregulated |
| ASPG | -1.035689556 | 2.43E-12 | 2.44E-11 | downregulated |
| GCNT3 | -1.041905519 | 2.78E-05 | 9.83E-05 | downregulated |
| AC009065.2 | -1.042312041 | 0.002469424 | 0.005350949 | downregulated |
| LINC02004 | -1.045665178 | 3.19E-08 | 1.98E-07 | downregulated |
| AC011473.3 | -1.045995353 | 0.006776839 | 0.013030957 | downregulated |
| C5orf66-AS1 | -1.048412797 | 5.01E-06 | 2.08E-05 | downregulated |
| DEFB4A | -1.050398094 | 0.014709616 | 0.025653469 | downregulated |
| AC026333.4 | -1.056767281 | 1.68E-09 | 1.25E-08 | downregulated |
| LINC01451 | -1.056789268 | 1.73E-08 | 1.12E-07 | downregulated |
| AC024592.2 | -1.058166563 | 3.67E-07 | 1.92E-06 | downregulated |
| C9orf24 | -1.065328267 | 0.022104767 | 0.036672956 | downregulated |
| MAP7D2 | -1.067275752 | 0.02531554 | 0.041405283 | downregulated |
| NMU | -1.06780111 | 3.47E-08 | 2.14E-07 | downregulated |
| PDCL2 | -1.073125536 | 0.000459443 | 0.001210108 | downregulated |
| RPL41P2 | -1.074105625 | 0.000152881 | 0.000453853 | downregulated |
| CYP4F2 | -1.081977951 | 0.000587177 | 0.001504353 | downregulated |
| LDLRAD1 | -1.087179042 | 2.04E-06 | 9.18E-06 | downregulated |
| AL136418.1 | -1.087219493 | 0.001842278 | 0.004129469 | downregulated |
| SLC25A41 | -1.098508927 | 1.20E-05 | 4.60E-05 | downregulated |
| BEST2 | -1.10111513 | 0.026684508 | 0.043405079 | downregulated |
| AC087783.2 | -1.104029086 | 0.000286087 | 0.000792746 | downregulated |
| LINC02178 | -1.106093573 | 7.34E-05 | 0.000236183 | downregulated |
| DMRTA2 | -1.106144319 | 0.002208632 | 0.004845159 | downregulated |
| AC015818.3 | -1.107439216 | 2.81E-06 | 1.23E-05 | downregulated |
| LDHC | -1.107682255 | 0.001194012 | 0.00280867 | downregulated |
| GSTA3 | -1.11935257 | 0.016459061 | 0.028264761 | downregulated |
| CASC8 | -1.128635809 | 0.001653152 | 0.003746748 | downregulated |
| HRK | -1.128884443 | 0.007923722 | 0.014945402 | downregulated |
| PRDM13 | -1.132383312 | 0.002673964 | 0.005729558 | downregulated |
| HMSD | -1.138778453 | 0.005847343 | 0.011434024 | downregulated |
| AC093904.3 | -1.141736636 | 0.00013684 | 0.0004115 | downregulated |
| SHISA8 | -1.142694227 | 0.013878083 | 0.024412165 | downregulated |
| AC106795.2 | -1.143797557 | 9.78E-06 | 3.81E-05 | downregulated |
| YBX2 | -1.153837581 | 0.029557254 | 0.047499704 | downregulated |
| AC009065.5 | -1.159346105 | 0.000509305 | 0.00132706 | downregulated |
| OR7E14P | -1.167942311 | 4.92E-07 | 2.52E-06 | downregulated |
| LINC00330 | -1.171134522 | 0.00710594 | 0.013580569 | downregulated |
| REN | -1.175182448 | 0.001005614 | 0.002413617 | downregulated |
| LYPD2 | -1.178068633 | 0.000850057 | 0.002082365 | downregulated |
| UGT1A8 | -1.181759494 | 0.024525954 | 0.040273819 | downregulated |
| LINC00393 | -1.182487338 | 0.000423327 | 0.001124968 | downregulated |
| SP9 | -1.182495919 | 0.002444746 | 0.005303296 | downregulated |
| AL356867.1 | -1.19379121 | 4.65E-07 | 2.39E-06 | downregulated |
| AC129926.1 | -1.195830709 | 7.06E-05 | 0.000228055 | downregulated |
| FUT6 | -1.197653624 | 3.28E-05 | 0.000113868 | downregulated |
| FOXH1 | -1.200588814 | 2.25E-05 | 8.12E-05 | downregulated |
| TLX3 | -1.214317668 | 0.000770411 | 0.001912804 | downregulated |
| AC105460.2 | -1.218885555 | 0.00795238 | 0.014986016 | downregulated |
| CCDC187 | -1.22755375 | 6.92E-06 | 2.79E-05 | downregulated |
| FAM3B | -1.231866052 | 0.022357516 | 0.037029759 | downregulated |
| MAJIN | -1.246962066 | 0.009080101 | 0.016851585 | downregulated |
| AC105460.1 | -1.247766578 | 0.02769268 | 0.044831747 | downregulated |
| CHCHD2P4 | -1.260603965 | 0.02504391 | 0.041024194 | downregulated |
| WFDC21P | -1.263534981 | 3.53E-05 | 0.000121539 | downregulated |
| LINC02448 | -1.271773906 | 0.000231184 | 0.000657668 | downregulated |
| MIR9-3HG | -1.278646018 | 8.65E-06 | 3.41E-05 | downregulated |
| AL136131.2 | -1.288535906 | 5.86E-07 | 2.95E-06 | downregulated |
| KCNJ18 | -1.292339731 | 0.015476416 | 0.026798517 | downregulated |
| AC087491.1 | -1.293050451 | 0.028037066 | 0.045345617 | downregulated |
| DCAF4L1 | -1.294219161 | 0.020195395 | 0.033842927 | downregulated |
| AACSP1 | -1.298210781 | 0.001115153 | 0.00264382 | downregulated |
| FTLP10 | -1.310173665 | 0.00020681 | 0.000594446 | downregulated |
| SYCP2 | -1.347622531 | 0.015353844 | 0.026611395 | downregulated |
| DKK4 | -1.371474371 | 0.000108815 | 0.000334929 | downregulated |
| GALR2 | -1.378651008 | 0.000247823 | 0.000699282 | downregulated |
| HMX2 | -1.388310804 | 0.000105154 | 0.000325279 | downregulated |
| TCAM1P | -1.396814494 | 0.010646263 | 0.019363646 | downregulated |
| C1orf194 | -1.3984383 | 0.003730711 | 0.007696379 | downregulated |
| SLC8A2 | -1.41263649 | 0.010338534 | 0.018880881 | downregulated |
| AL138760.1 | -1.424127549 | 0.000225883 | 0.000644038 | downregulated |
| SPTLC1P4 | -1.437473452 | 0.004099595 | 0.008366707 | downregulated |
| MIOX | -1.480570068 | 0.001416039 | 0.003266587 | downregulated |
| SPDYC | -1.5009351 | 0.016526559 | 0.028363775 | downregulated |
| LINC00556 | -1.513325998 | 0.006798171 | 0.013066632 | downregulated |
| LGALS4 | -1.53619312 | 0.000328254 | 0.000895417 | downregulated |
| BTBD16 | -1.541945395 | 0.002536248 | 0.00547461 | downregulated |
| LINC01983 | -1.575475824 | 0.000224069 | 0.000639589 | downregulated |
| NR5A1 | -1.656462953 | 0.011292789 | 0.020365172 | downregulated |
| SLC9A4 | -1.744217486 | 0.020606754 | 0.034446448 | downregulated |
| KRT24 | -1.7567107 | 0.00046493 | 0.001222963 | downregulated |
| KRT8P40 | -1.765029523 | 0.001935691 | 0.004317274 | downregulated |
| STOML3 | -1.766781277 | 0.007599023 | 0.014408296 | downregulated |
| SFTA2 | -1.780127744 | 0.000213295 | 0.000610912 | downregulated |
| LCN12 | -1.786284686 | 2.90E-05 | 0.000101929 | downregulated |
| TUBB8P7 | -1.828770072 | 0.000149943 | 0.000445984 | downregulated |
| LINC01305 | -1.83804033 | 0.000257944 | 0.000723794 | downregulated |
| AC025181.1 | -1.867801835 | 0.000698447 | 0.001750933 | downregulated |
| HBQ1 | -1.876623992 | 0.006156441 | 0.011975771 | downregulated |
| PROSER2-AS1 | -1.920348762 | 0.000463307 | 0.001219012 | downregulated |
| C11orf16 | -1.971200533 | 0.005248513 | 0.010407664 | downregulated |
| CYP2AB1P | -2.008051241 | 7.17E-07 | 3.55E-06 | downregulated |
| C8G | -2.109905978 | 4.52E-05 | 0.000152093 | downregulated |
| TOB1-AS1 | -2.340146668 | 0.00799635 | 0.01506258 | downregulated |
| ATP13A5 | -2.346403035 | 0.026657369 | 0.043371423 | downregulated |
| BOK-AS1 | -2.438357763 | 0.007254397 | 0.013844695 | downregulated |
| LINC00974 | -3.176591292 | 0.01224115 | 0.021889572 | downregulated |
