# Supplementary material for: Effective TME-related signature to predict prognosis of patients with head and neck squamous cell carcinoma
Source: Front Mol Biosci. 2023 Aug 21;10:1232875. doi: 10.3389/fmolb.2023.1232875 (PMC10475735; doi:10.3389/fmolb.2023.1232875)
Supplement: Supplementary file 1 [file DataSheet1.zip › Supplementary Material/Supplementary Table S3.docx]

Table S3. 365 upregulated and 30 downregulated genes by taking the intersection of the two sets of DEGs.

| Gene | Log_2_FC | regulated |
| --- | --- | --- |
| DNASE2B | 2.660173644 | upregulated |
| PLA2G2A | 2.589331098 | upregulated |
| PLA2G2D | 2.443980521 | upregulated |
| PI16 | 2.439000816 | upregulated |
| IGKV1D-12 | 2.3929513 | upregulated |
| CHIT1 | 2.342872549 | upregulated |
| ODAM | 2.336215682 | upregulated |
| IGLV2-34 | 2.31576141 | upregulated |
| CILP | 2.264399163 | upregulated |
| CXCR2P1 | 2.258541517 | upregulated |
| PPP1R1A | 2.199328815 | upregulated |
| SFRP4 | 2.18884668 | upregulated |
| IGLV3-16 | 2.150379778 | upregulated |
| TLR8 | 2.143205611 | upregulated |
| IGKV1D-33 | 2.116951769 | upregulated |
| IGLV2-8 | 2.115928797 | upregulated |
| EPYC | 2.090331518 | upregulated |
| IGHV3-43 | 2.082639267 | upregulated |
| LILRA4 | 2.064902099 | upregulated |
| ADAMDEC1 | 2.040127828 | upregulated |
| ANGPTL7 | 2.021425792 | upregulated |
| SIGLEC8 | 1.987169876 | upregulated |
| LBP | 1.987162717 | upregulated |
| LILRB4 | 1.98002837 | upregulated |
| CCR2 | 1.958622261 | upregulated |
| CCL18 | 1.952072514 | upregulated |
| PTGIS | 1.948209053 | upregulated |
| IGLV3-12 | 1.946368657 | upregulated |
| IGHV1OR16-3 | 1.934115023 | upregulated |
| IGKV2OR2-1 | 1.933919664 | upregulated |
| TIMD4 | 1.933041402 | upregulated |
| TLR7 | 1.921375767 | upregulated |
| TCL1A | 1.917107293 | upregulated |
| IGHV7-81 | 1.914669171 | upregulated |
| CYBB | 1.913819809 | upregulated |
| THBS4 | 1.907586068 | upregulated |
| CCKAR | 1.906854544 | upregulated |
| TRBV6-6 | 1.902123267 | upregulated |
| CD84 | 1.896964264 | upregulated |
| CD163 | 1.883474192 | upregulated |
| IGKV2-28 | 1.883219248 | upregulated |
| IGKV1-16 | 1.870767952 | upregulated |
| CCL19 | 1.861187126 | upregulated |
| IGKV1D-17 | 1.847489097 | upregulated |
| CR1 | 1.842200094 | upregulated |
| IGLV6-57 | 1.827291885 | upregulated |
| HK3 | 1.825888791 | upregulated |
| ADAMTS16 | 1.825285665 | upregulated |
| IGKV2-18 | 1.818128943 | upregulated |
| IGKV1-17 | 1.809486553 | upregulated |
| IGHV3-20 | 1.808537704 | upregulated |
| TRAV24 | 1.804916543 | upregulated |
| CXCL9 | 1.803008047 | upregulated |
| F13A1 | 1.802225675 | upregulated |
| LINC01638 | 1.794849137 | upregulated |
| TRAV21 | 1.7948295 | upregulated |
| MRC1 | 1.788361489 | upregulated |
| CMA1 | 1.787657306 | upregulated |
| IGHV1OR15-2 | 1.786005714 | upregulated |
| SCIMP | 1.785860341 | upregulated |
| TRAV20 | 1.782725458 | upregulated |
| CLC | 1.772143454 | upregulated |
| SIGLEC1 | 1.770481262 | upregulated |
| VSIG4 | 1.763890852 | upregulated |
| TRBV5-1 | 1.762626437 | upregulated |
| FGL2 | 1.759581349 | upregulated |
| IGKV1OR2-108 | 1.758780488 | upregulated |
| TIFAB | 1.754767252 | upregulated |
| IGHV3-21 | 1.749440134 | upregulated |
| IGHV1-69D | 1.748012938 | upregulated |
| ANGPTL1 | 1.74796466 | upregulated |
| TRAV35 | 1.746895327 | upregulated |
| FPR3 | 1.74351659 | upregulated |
| WDFY4 | 1.733178306 | upregulated |
| P2RY12 | 1.726963493 | upregulated |
| TRBV3-1 | 1.724341378 | upregulated |
| MPEG1 | 1.717299663 | upregulated |
| P2RY13 | 1.717198492 | upregulated |
| FCN1 | 1.713028883 | upregulated |
| IGHV1-18 | 1.710224161 | upregulated |
| IGHV1-46 | 1.70687676 | upregulated |
| CHI3L1 | 1.705464131 | upregulated |
| PTPRC | 1.700236963 | upregulated |
| SLCO2B1 | 1.695964662 | upregulated |
| CD209 | 1.695938061 | upregulated |
| PTCRA | 1.695670751 | upregulated |
| KCNA3 | 1.694459818 | upregulated |
| IGHV3OR16-9 | 1.693959561 | upregulated |
| IRF8 | 1.691341214 | upregulated |
| C1QB | 1.690751349 | upregulated |
| FCGR3A | 1.690713173 | upregulated |
| TRAV9-2 | 1.690141599 | upregulated |
| TFEC | 1.684419814 | upregulated |
| SIGLEC10 | 1.677036547 | upregulated |
| DNAJC5B | 1.674748 | upregulated |
| TRAV14DV4 | 1.671818502 | upregulated |
| IGKV6-21 | 1.67020039 | upregulated |
| PIK3CG | 1.666629392 | upregulated |
| CCR1 | 1.665932824 | upregulated |
| LYZ | 1.665757826 | upregulated |
| TRAV2 | 1.665261064 | upregulated |
| AQP9 | 1.663266246 | upregulated |
| NCKAP1L | 1.66127053 | upregulated |
| PLEK | 1.660995626 | upregulated |
| IGKV1D-8 | 1.65992355 | upregulated |
| MS4A4A | 1.656049246 | upregulated |
| MSR1 | 1.654970823 | upregulated |
| LILRB1 | 1.654762594 | upregulated |
| ITGB2 | 1.650176852 | upregulated |
| CLEC10A | 1.64742255 | upregulated |
| SIGLEC7 | 1.646601376 | upregulated |
| CSF1R | 1.645041366 | upregulated |
| DOCK2 | 1.643047051 | upregulated |
| C1QC | 1.642009415 | upregulated |
| P2RY10 | 1.641233595 | upregulated |
| TRBV24-1 | 1.636957639 | upregulated |
| IGLV3-21 | 1.633951209 | upregulated |
| IGKV1-33 | 1.630083563 | upregulated |
| APOC1 | 1.628997471 | upregulated |
| C3AR1 | 1.627955163 | upregulated |
| TRAV5 | 1.625878871 | upregulated |
| CD4 | 1.623914561 | upregulated |
| IGHV2-70D | 1.621974615 | upregulated |
| MNDA | 1.621486319 | upregulated |
| FCGR1A | 1.617232798 | upregulated |
| CMKLR1 | 1.615492391 | upregulated |
| XIRP1 | 1.615119593 | upregulated |
| SUSD2 | 1.609001195 | upregulated |
| AC083949.1 | 1.606387036 | upregulated |
| LAIR1 | 1.605167584 | upregulated |
| SIGLEC14 | 1.603630739 | upregulated |
| IGKV1OR2-3 | 1.602110724 | upregulated |
| SPN | 1.601836909 | upregulated |
| C1QA | 1.600416779 | upregulated |
| ADRA2A | 1.596511378 | upregulated |
| IGHV2-5 | 1.595670981 | upregulated |
| IGHV1OR16-1 | 1.594373764 | upregulated |
| SIRPB1 | 1.592595018 | upregulated |
| GPR141 | 1.591026826 | upregulated |
| DPT | 1.590491388 | upregulated |
| IGHV4-61 | 1.590406943 | upregulated |
| SELL | 1.583607978 | upregulated |
| CD53 | 1.582887515 | upregulated |
| CCR4 | 1.582050414 | upregulated |
| IGKV1OR2-11 | 1.580414027 | upregulated |
| CD28 | 1.575298941 | upregulated |
| FOLR2 | 1.570015527 | upregulated |
| CD1B | 1.564313992 | upregulated |
| SLAMF8 | 1.56271375 | upregulated |
| MS4A6A | 1.562582154 | upregulated |
| IGHV1-69 | 1.555529943 | upregulated |
| IGHV3-76 | 1.555238455 | upregulated |
| SIGLEC12 | 1.553381445 | upregulated |
| IGLV3-10 | 1.552090099 | upregulated |
| HLA-DQA1 | 1.550061706 | upregulated |
| LILRB2 | 1.548139233 | upregulated |
| FCGR1B | 1.547696797 | upregulated |
| EVI2B | 1.546931595 | upregulated |
| SLC7A7 | 1.546124065 | upregulated |
| IGHV3OR16-10 | 1.544332303 | upregulated |
| MS4A7 | 1.53499324 | upregulated |
| ABI3BP | 1.534409927 | upregulated |
| LINC02345 | 1.533063338 | upregulated |
| IGHV3-13 | 1.531324991 | upregulated |
| RGS18 | 1.528973071 | upregulated |
| HSD11B1 | 1.528136598 | upregulated |
| SUCNR1 | 1.526155452 | upregulated |
| CCR8 | 1.526095395 | upregulated |
| AC134879.2 | 1.526004026 | upregulated |
| LILRB5 | 1.520955924 | upregulated |
| CASS4 | 1.51960527 | upregulated |
| AF127936.1 | 1.519106243 | upregulated |
| AC090559.1 | 1.517364311 | upregulated |
| IGHV1-2 | 1.51729845 | upregulated |
| TNFSF8 | 1.516477281 | upregulated |
| PRKCB | 1.515680547 | upregulated |
| IGSF6 | 1.514712193 | upregulated |
| ADA2 | 1.513374456 | upregulated |
| IGKV2D-28 | 1.51150797 | upregulated |
| ABCA8 | 1.51016893 | upregulated |
| NFAM1 | 1.509592646 | upregulated |
| ADH1B | 1.508910126 | upregulated |
| IL10RA | 1.508868544 | upregulated |
| TLR4 | 1.507150978 | upregulated |
| IGHV3-64 | 1.506563238 | upregulated |
| MMRN1 | 1.505620634 | upregulated |
| MMP2-AS1 | 1.503512662 | upregulated |
| IGHV3-66 | 1.499970221 | upregulated |
| CD300LF | 1.498014002 | upregulated |
| CXorf21 | 1.495795949 | upregulated |
| LRRC25 | 1.495757789 | upregulated |
| FCGR1CP | 1.489681758 | upregulated |
| IGHV3-23 | 1.488636531 | upregulated |
| MMP12 | 1.488419389 | upregulated |
| TRAV8-1 | 1.487937977 | upregulated |
| TRAV26-1 | 1.484745809 | upregulated |
| TNFSF13B | 1.482350827 | upregulated |
| PRELP | 1.481980651 | upregulated |
| GPR34 | 1.480979781 | upregulated |
| DCSTAMP | 1.479861034 | upregulated |
| CTSS | 1.473309333 | upregulated |
| EVI2A | 1.471995751 | upregulated |
| CCL13 | 1.47071269 | upregulated |
| TMEM176B | 1.467885255 | upregulated |
| IGKV1OR2-6 | 1.466863444 | upregulated |
| LILRA6 | 1.4660576 | upregulated |
| PLD4 | 1.46595863 | upregulated |
| PLA2G7 | 1.463289777 | upregulated |
| IL2RA | 1.460320233 | upregulated |
| FPR2 | 1.457792824 | upregulated |
| CD300LB | 1.454707619 | upregulated |
| PLXNC1 | 1.453771531 | upregulated |
| CLEC4E | 1.453629589 | upregulated |
| IGHV2-70 | 1.453615093 | upregulated |
| HS3ST2 | 1.453296287 | upregulated |
| C4B | 1.450597763 | upregulated |
| IGKV1OR22-1 | 1.448583961 | upregulated |
| HAVCR2 | 1.448064609 | upregulated |
| LY86 | 1.446492595 | upregulated |
| C4A | 1.44554674 | upregulated |
| LCP2 | 1.443747649 | upregulated |
| LAPTM5 | 1.443575116 | upregulated |
| IGHV7-56 | 1.443442725 | upregulated |
| LILRA1 | 1.441048908 | upregulated |
| PILRA | 1.437903899 | upregulated |
| TYROBP | 1.436541194 | upregulated |
| CCL7 | 1.433791835 | upregulated |
| GGTA1P | 1.428808495 | upregulated |
| GPRIN3 | 1.426558654 | upregulated |
| CD300C | 1.425343219 | upregulated |
| GPR65 | 1.42526489 | upregulated |
| IGHV3-11 | 1.424755358 | upregulated |
| P2RY8 | 1.424280183 | upregulated |
| RNASE6 | 1.422634424 | upregulated |
| SPI1 | 1.422307741 | upregulated |
| IGHV1-68 | 1.420887433 | upregulated |
| GIMAP5 | 1.416049557 | upregulated |
| CD180 | 1.410769771 | upregulated |
| IGHV3OR16-16 | 1.409879663 | upregulated |
| CCL23 | 1.406895617 | upregulated |
| FERMT3 | 1.406575306 | upregulated |
| AL133371.2 | 1.402108411 | upregulated |
| GIMAP4 | 1.40170964 | upregulated |
| AC098613.1 | 1.400772003 | upregulated |
| LILRA2 | 1.400643013 | upregulated |
| FCER1G | 1.400508167 | upregulated |
| CTSG | 1.399856223 | upregulated |
| ATP6V0D2 | 1.393865317 | upregulated |
| ENPP2 | 1.391228131 | upregulated |
| PTGER2 | 1.390238182 | upregulated |
| FCAR | 1.38917457 | upregulated |
| GFRA1 | 1.389125051 | upregulated |
| FPR1 | 1.384069024 | upregulated |
| IGHV3-65 | 1.382410348 | upregulated |
| BTK | 1.381685441 | upregulated |
| TMEM150B | 1.379711759 | upregulated |
| SIGLEC9 | 1.378399168 | upregulated |
| LINC02285 | 1.378024229 | upregulated |
| NTRK1 | 1.377283419 | upregulated |
| TMEM176A | 1.376899367 | upregulated |
| TM6SF1 | 1.376225992 | upregulated |
| CHRDL1 | 1.375928626 | upregulated |
| GIMAP6 | 1.375792126 | upregulated |
| CD80 | 1.369668968 | upregulated |
| P2RY14 | 1.369130535 | upregulated |
| FGR | 1.368146172 | upregulated |
| IGLV3-13 | 1.366007233 | upregulated |
| CLEC4D | 1.352346709 | upregulated |
| IGHV4-4 | 1.351366244 | upregulated |
| IGHV3-71 | 1.351189935 | upregulated |
| RF00397 | 1.350728269 | upregulated |
| IGLV3-22 | 1.348625569 | upregulated |
| TNXB | 1.347209909 | upregulated |
| CCRL2 | 1.346438595 | upregulated |
| SELP | 1.343589301 | upregulated |
| C5AR1 | 1.34334033 | upregulated |
| RERGL | 1.342929163 | upregulated |
| LPL | 1.340239919 | upregulated |
| IGKV1D-42 | 1.337433901 | upregulated |
| RSPO3 | 1.334541499 | upregulated |
| ITGAX | 1.334457044 | upregulated |
| AC109826.1 | 1.333654574 | upregulated |
| AC011899.2 | 1.330561735 | upregulated |
| RSPO1 | 1.324658075 | upregulated |
| TDRD6 | 1.319422163 | upregulated |
| FCGR2B | 1.317421155 | upregulated |
| GFRA2 | 1.315319082 | upregulated |
| PIK3R5 | 1.313505784 | upregulated |
| CLEC4A | 1.312614084 | upregulated |
| MARCO | 1.31249422 | upregulated |
| MAP1LC3C | 1.310355116 | upregulated |
| GPR183 | 1.30552745 | upregulated |
| LILRB3 | 1.304844946 | upregulated |
| LINC01150 | 1.301255489 | upregulated |
| CD14 | 1.30024015 | upregulated |
| OSM | 1.299032683 | upregulated |
| IGKV1D-16 | 1.291367584 | upregulated |
| AC244669.2 | 1.291199299 | upregulated |
| CYSLTR1 | 1.289972002 | upregulated |
| ARHGEF6 | 1.289918429 | upregulated |
| COL6A6 | 1.283190057 | upregulated |
| SRGN | 1.280893273 | upregulated |
| CD163L1 | 1.277713921 | upregulated |
| ALOX5 | 1.272507383 | upregulated |
| FCGR2A | 1.271313467 | upregulated |
| SYNE1 | 1.270285464 | upregulated |
| APOE | 1.26743409 | upregulated |
| RCSD1 | 1.266252706 | upregulated |
| NHSL2 | 1.265687953 | upregulated |
| ZNF366 | 1.264047119 | upregulated |
| FCGR3B | 1.263743591 | upregulated |
| FMO1 | 1.263575466 | upregulated |
| AC004988.1 | 1.256674284 | upregulated |
| AC004921.1 | 1.254225245 | upregulated |
| CCL24 | 1.252253723 | upregulated |
| SYNPO2 | 1.251025016 | upregulated |
| CCL2 | 1.249738723 | upregulated |
| BHLHE22 | 1.244357773 | upregulated |
| SLC8A1 | 1.239488836 | upregulated |
| CYP1B1 | 1.238837786 | upregulated |
| PLA1A | 1.237036845 | upregulated |
| IGHV3-75 | 1.236514352 | upregulated |
| CSF3R | 1.235094841 | upregulated |
| RASSF4 | 1.23472472 | upregulated |
| TMEM273 | 1.231438656 | upregulated |
| AL161935.3 | 1.230294137 | upregulated |
| AOAH | 1.229865379 | upregulated |
| ITM2A | 1.222001664 | upregulated |
| RASGRP4 | 1.221194622 | upregulated |
| AC010175.1 | 1.220129412 | upregulated |
| IGHV1-17 | 1.214596718 | upregulated |
| TBXAS1 | 1.21224462 | upregulated |
| WIPF1 | 1.189545427 | upregulated |
| NRROS | 1.188913549 | upregulated |
| SELE | 1.187794259 | upregulated |
| IGSF21 | 1.186982734 | upregulated |
| AC131097.4 | 1.186053288 | upregulated |
| SERPING1 | 1.183914687 | upregulated |
| STAB1 | 1.182252325 | upregulated |
| IGHV3OR16-15 | 1.182252046 | upregulated |
| CCL14 | 1.179358873 | upregulated |
| MS4A2 | 1.175127599 | upregulated |
| PREX1 | 1.16496953 | upregulated |
| SIGLEC6 | 1.164725837 | upregulated |
| AC023449.2 | 1.162069455 | upregulated |
| IL10 | 1.160287816 | upregulated |
| ACKR1 | 1.159582998 | upregulated |
| AC008957.1 | 1.15635876 | upregulated |
| GIMAP8 | 1.154706277 | upregulated |
| IQGAP2 | 1.150452892 | upregulated |
| CD300E | 1.144106772 | upregulated |
| SLC14A1 | 1.133122538 | upregulated |
| SDS | 1.132536951 | upregulated |
| FBP1 | 1.125224638 | upregulated |
| IGHE | 1.118505815 | upregulated |
| AL590648.3 | 1.118262531 | upregulated |
| CHST13 | 1.109423303 | upregulated |
| TACR1 | 1.098295623 | upregulated |
| FLI1 | 1.097852482 | upregulated |
| SIRPB2 | 1.081645096 | upregulated |
| SMIM25 | 1.072242857 | upregulated |
| IGHV3OR16-6 | 1.071859786 | upregulated |
| CLEC5A | 1.064127949 | upregulated |
| AC245128.3 | 1.050649398 | upregulated |
| RNASE1 | 1.018897857 | upregulated |
| SLC13A4 | -1.065437158 | downregulated |
| AC083801.2 | -1.09792155 | downregulated |
| AC105460.2 | -1.158413407 | downregulated |
| AC005077.4 | -1.166066569 | downregulated |
| DKK4 | -1.202335753 | downregulated |
| AL391427.1 | -1.225475509 | downregulated |
| PRDM13 | -1.227801488 | downregulated |
| AC105460.1 | -1.25014111 | downregulated |
| CHCHD2P4 | -1.27534181 | downregulated |
| CASC8 | -1.286410296 | downregulated |
| CYP4F2 | -1.34971643 | downregulated |
| GSTA3 | -1.377521983 | downregulated |
| SLC8A2 | -1.412691982 | downregulated |
| FOXH1 | -1.488772756 | downregulated |
| TUBB8P7 | -1.523020375 | downregulated |
| GALR2 | -1.5624127 | downregulated |
| LINC00556 | -1.581708273 | downregulated |
| BTBD16 | -1.627554629 | downregulated |
| LINC00393 | -1.642920648 | downregulated |
| SPTLC1P4 | -1.681484896 | downregulated |
| AC087783.2 | -1.714377203 | downregulated |
| SLC9A4 | -1.714615722 | downregulated |
| AC087491.1 | -1.738871736 | downregulated |
| ATP13A5 | -1.776035547 | downregulated |
| SPDYC | -1.801744496 | downregulated |
| NR5A1 | -1.872609729 | downregulated |
| CYP2AB1P | -1.93767342 | downregulated |
| HBQ1 | -2.217281957 | downregulated |
| BOK-AS1 | -2.251964257 | downregulated |
| LINC00974 | -3.159884238 | downregulated |
