# Supplementary material for: Effective TME-related signature to predict prognosis of patients with head and neck squamous cell carcinoma
Source: Front Mol Biosci. 2023 Aug 21;10:1232875. doi: 10.3389/fmolb.2023.1232875 (PMC10475735; doi:10.3389/fmolb.2023.1232875)
Supplement: Supplementary file 1 [file DataSheet1.zip › Supplementary Material/Supplementary Table S4.docx]

Table S4. 13 genes identified by the univariate Cox analysis.

| ID | HR | HR.95L | HR.95H | pvalue |
| --- | --- | --- | --- | --- |
| IGHV3-20 | 0.849551235 | 0.76315484 | 0.945728524 | 0.002885176 |
| CXorf21 | 0.75370711 | 0.573988449 | 0.989696586 | 0.041902736 |
| IGHV3-66 | 0.907991762 | 0.831008954 | 0.992106085 | 0.03273713 |
| IGHV1-69D | 0.92583813 | 0.873613487 | 0.981184764 | 0.009291001 |
| SYNE1 | 0.634843377 | 0.416500872 | 0.967647704 | 0.034609985 |
| IGLV2-34 | 0.809648424 | 0.668118832 | 0.981158649 | 0.031240268 |
| RCSD1 | 0.75778991 | 0.61751166 | 0.929934743 | 0.007920065 |
| AC010175.1 | 0.488010494 | 0.252040932 | 0.944903039 | 0.033331069 |
| GIMAP6 | 0.79537486 | 0.667912187 | 0.947162186 | 0.010195163 |
| TRAV21 | 0.681271422 | 0.517427856 | 0.896996065 | 0.006248344 |
| TNFSF8 | 0.539524981 | 0.362218112 | 0.803624102 | 0.002402268 |
| IGLV3-21 | 0.919053543 | 0.873843314 | 0.966602824 | 0.001038925 |
| IGHV3-23 | 0.908444156 | 0.86105666 | 0.958439581 | 0.000443169 |
| CCR8 | 0.683606177 | 0.519156216 | 0.900147954 | 0.006743976 |
| WIPF1 | 0.775097883 | 0.660350973 | 0.909783968 | 0.001829607 |
| IGKV1OR2-108 | 0.888891169 | 0.810242343 | 0.975174302 | 0.012709142 |
| IGHV1-17 | 0.65772528 | 0.476029624 | 0.908772316 | 0.011089006 |
| NCKAP1L | 0.806293412 | 0.676872396 | 0.960460302 | 0.015870345 |
| IGLV3-16 | 0.823412608 | 0.711290096 | 0.953209284 | 0.009278588 |
| IGKV1D-12 | 0.809813969 | 0.67762939 | 0.967783679 | 0.02033406 |
| CD180 | 0.70602801 | 0.522818148 | 0.953439649 | 0.023145447 |
| CCR4 | 0.612056069 | 0.480140619 | 0.780214413 | 7.37E-05 |
| BHLHE22 | 0.479962227 | 0.269281019 | 0.855477079 | 0.012798759 |
| TRAV14DV4 | 0.505292708 | 0.320565408 | 0.796469971 | 0.003280866 |
| IGHV3-11 | 0.900818421 | 0.848260881 | 0.956632382 | 0.000660508 |
| P2RY10 | 0.665638958 | 0.524414807 | 0.84489457 | 0.000822114 |
| TRBV3-1 | 0.707531347 | 0.514241292 | 0.973474153 | 0.033578652 |
| ARHGEF6 | 0.76371474 | 0.622858062 | 0.936425553 | 0.009557775 |
| IGKV1OR2-6 | 0.849924884 | 0.734032134 | 0.984115374 | 0.02970177 |
| RF00397 | 0.334055318 | 0.146941268 | 0.759439175 | 0.008879282 |
| CXCR2P1 | 0.795843641 | 0.64386433 | 0.98369652 | 0.034686888 |
| IGHV1-46 | 0.907350989 | 0.852844438 | 0.965341134 | 0.002098618 |
| AC011899.2 | 0.404710037 | 0.182278292 | 0.898572244 | 0.026232078 |
| PREX1 | 0.80161843 | 0.678084405 | 0.947657996 | 0.009609697 |
| NFAM1 | 0.780730343 | 0.638309925 | 0.954927762 | 0.016006261 |
| FERMT3 | 0.859008673 | 0.749588802 | 0.984400912 | 0.028806877 |
| FLI1 | 0.714331911 | 0.553623941 | 0.921690774 | 0.009679515 |
| ITM2A | 0.822356427 | 0.707493246 | 0.955867913 | 0.010834975 |
| IGKV1D-16 | 0.900442181 | 0.813823092 | 0.996280555 | 0.042136009 |
| TRAV9-2 | 0.611235026 | 0.439390754 | 0.850287026 | 0.003467486 |
| LILRA4 | 0.640060392 | 0.449550345 | 0.911304618 | 0.013316453 |
| EVI2B | 0.863639756 | 0.755898534 | 0.986737763 | 0.031056783 |
| KCNA3 | 0.480704629 | 0.317895452 | 0.726896025 | 0.00051706 |
| CLEC10A | 0.775747671 | 0.645727024 | 0.931948682 | 0.006668959 |
| GPR65 | 0.687363623 | 0.47462526 | 0.995456395 | 0.047248571 |
| SELP | 0.800509872 | 0.673397079 | 0.95161692 | 0.011665859 |
| IL10 | 0.569280666 | 0.351629666 | 0.921652829 | 0.021913594 |
| IGHV1-68 | 0.615170762 | 0.397979704 | 0.950890366 | 0.028772206 |
| IGKV6-21 | 0.864692298 | 0.784667145 | 0.952878904 | 0.003345151 |
| IGHV3-75 | 0.694502487 | 0.487431845 | 0.989540813 | 0.043573552 |
| IGHV4-4 | 0.878986615 | 0.788560247 | 0.979782423 | 0.019874244 |
| TCL1A | 0.628863535 | 0.465731111 | 0.849136629 | 0.002467603 |
| ZNF366 | 0.526667313 | 0.333895196 | 0.830735099 | 0.005824772 |
| IGHE | 0.786461185 | 0.624995949 | 0.989640327 | 0.040483321 |
| LYZ | 0.922200441 | 0.858203405 | 0.990969797 | 0.027302329 |
| IGLV6-57 | 0.916624077 | 0.863043215 | 0.973531434 | 0.00461348 |
| TRBV6-6 | 0.539418173 | 0.36345594 | 0.800570119 | 0.002183117 |
| IGHV3-64 | 0.800722795 | 0.707723565 | 0.905942696 | 0.000418558 |
| CCR2 | 0.687063981 | 0.504595674 | 0.935515181 | 0.017161832 |
| PIK3CG | 0.66535556 | 0.501327465 | 0.883051602 | 0.004785454 |
| CTSG | 0.744905954 | 0.606560843 | 0.914804982 | 0.004963045 |
| IGKV1OR2-3 | 0.712027788 | 0.535160345 | 0.947348914 | 0.019742166 |
| IGHV1OR15-2 | 0.863738629 | 0.767422603 | 0.97214288 | 0.015169334 |
| GFRA1 | 0.691184183 | 0.488349398 | 0.978265923 | 0.03716522 |
| AC023449.2 | 0.235242345 | 0.075065654 | 0.73720747 | 0.013024078 |
| PLA2G2D | 0.756239544 | 0.63045893 | 0.907114199 | 0.002609705 |
| CD4 | 0.879520611 | 0.777063974 | 0.995486254 | 0.042198768 |
| IGKV2-28 | 0.819417796 | 0.713966334 | 0.940444237 | 0.004603012 |
| CCL19 | 0.918659638 | 0.851413509 | 0.991216985 | 0.02871213 |
| IL10RA | 0.799989498 | 0.681677354 | 0.938835937 | 0.006277953 |
| TRAV35 | 0.520131858 | 0.270924588 | 0.998569939 | 0.049499444 |
| IGKV1-16 | 0.900953163 | 0.84675147 | 0.958624379 | 0.000984982 |
| IGKV2D-28 | 0.823264281 | 0.679828015 | 0.996964028 | 0.046470556 |
| TRBV5-1 | 0.593068171 | 0.449366127 | 0.782724453 | 0.000223918 |
| MPEG1 | 0.858725429 | 0.75260074 | 0.979814826 | 0.023639358 |
| IGHV1-2 | 0.901088501 | 0.851008343 | 0.954115778 | 0.000357085 |
| NRROS | 0.781230598 | 0.617792007 | 0.987907323 | 0.039249964 |
| RSPO1 | 0.31066377 | 0.141713061 | 0.681037991 | 0.003509538 |
| IGHV2-5 | 0.861800715 | 0.78707318 | 0.943623149 | 0.001309552 |
| CD28 | 0.575816764 | 0.418680337 | 0.791928631 | 0.000687007 |
| P2RY14 | 0.428853378 | 0.258570765 | 0.711276156 | 0.001038926 |
| PIK3R5 | 0.717060173 | 0.539593323 | 0.952894097 | 0.021873444 |
| IGHV3-21 | 0.916701086 | 0.865031973 | 0.97145644 | 0.003300146 |
| PTPRC | 0.841659236 | 0.743265384 | 0.953078517 | 0.006575559 |
| SIGLEC6 | 0.244435768 | 0.095674655 | 0.624500239 | 0.00324298 |
| TIFAB | 0.389081993 | 0.195899989 | 0.772765723 | 0.007012219 |
| SELL | 0.837560244 | 0.737727748 | 0.950902503 | 0.006192542 |
| IRF8 | 0.800581355 | 0.683058126 | 0.938324985 | 0.006035047 |
| IGHV7-81 | 0.800862693 | 0.649546167 | 0.98742951 | 0.037676258 |
| P2RY8 | 0.63755824 | 0.506305964 | 0.802835713 | 0.000129582 |
| PLA1A | 0.772067048 | 0.602344425 | 0.989612425 | 0.041111202 |
| IGKV1OR22-1 | 0.800943433 | 0.657761984 | 0.975292581 | 0.027179237 |
| GPRIN3 | 0.585715373 | 0.409926434 | 0.836887962 | 0.003303882 |
| TRAV5 | 0.472039363 | 0.285475091 | 0.780527504 | 0.003437387 |
| GIMAP5 | 0.252281812 | 0.101537097 | 0.626826202 | 0.003018562 |
| IGHV3-43 | 0.902213592 | 0.829205229 | 0.981650063 | 0.016842231 |
| IGHV3OR16-9 | 0.822356938 | 0.694363606 | 0.973943519 | 0.023461481 |
| TRAV2 | 0.653107775 | 0.475076985 | 0.897853987 | 0.008703035 |
| MS4A2 | 0.432263344 | 0.241078767 | 0.775064519 | 0.004873792 |
| IGKV1D-8 | 0.883938616 | 0.786360018 | 0.993625642 | 0.038723531 |
| IGHV4-61 | 0.869628841 | 0.794382899 | 0.952002268 | 0.002484542 |
| PTCRA | 0.387937536 | 0.185779357 | 0.810076722 | 0.011714033 |
| CHIT1 | 0.863054527 | 0.750988172 | 0.991844005 | 0.037952648 |
| IGHV3OR16-6 | 0.748059371 | 0.580581306 | 0.963849191 | 0.02478748 |
| PLD4 | 0.564896494 | 0.381222138 | 0.837065891 | 0.004422212 |
| BTK | 0.71658185 | 0.557155073 | 0.921627699 | 0.009442233 |
| IGHV3-13 | 0.853351288 | 0.776865096 | 0.937367923 | 0.000933146 |
| IGLV3-10 | 0.909865058 | 0.858146153 | 0.964700966 | 0.001558638 |
| IGHV3-71 | 0.80095354 | 0.670278491 | 0.957104521 | 0.014589165 |
| PLXNC1 | 0.811384251 | 0.664336134 | 0.990980874 | 0.04048447 |
| WDFY4 | 0.591040863 | 0.446811113 | 0.781827693 | 0.000229311 |
| SPN | 0.765919394 | 0.632914441 | 0.926874914 | 0.006139242 |
| IGHV1OR16-1 | 0.648136863 | 0.435553037 | 0.964478163 | 0.032491953 |
| IGHV2-70 | 0.919328941 | 0.855341737 | 0.98810296 | 0.022305886 |
| CD84 | 0.790608301 | 0.637091446 | 0.981117372 | 0.032922257 |
| AC134879.2 | 0.784234403 | 0.653734237 | 0.940785359 | 0.008863279 |
| PRKCB | 0.651840423 | 0.492488838 | 0.862752419 | 0.002770396 |
| TRAV26-1 | 0.503842833 | 0.323866148 | 0.783834934 | 0.002364753 |
| IGKV2OR2-1 | 0.633778188 | 0.44621332 | 0.900185568 | 0.010855881 |
| IGLV2-8 | 0.89508965 | 0.839181121 | 0.954722958 | 0.000757217 |
| DOCK2 | 0.731803696 | 0.588675291 | 0.909731829 | 0.004924282 |
| IGHV1-18 | 0.926142032 | 0.878869942 | 0.975956762 | 0.004099213 |
| IGLV3-13 | 0.618002359 | 0.425181344 | 0.898268282 | 0.011661042 |
| IGLV3-22 | 0.558000763 | 0.34298618 | 0.907805823 | 0.018798487 |
| IGKV1-17 | 0.904329156 | 0.847917768 | 0.964493556 | 0.002212931 |
| COL6A6 | 0.52028016 | 0.273385638 | 0.990145082 | 0.046576661 |
| HBQ1 | 1.362519216 | 1.010679325 | 1.836842377 | 0.042390759 |
| CASC8 | 1.221118319 | 1.02860916 | 1.449656494 | 0.022476455 |
