# Supplementary material for: Effective TME-related signature to predict prognosis of patients with head and neck squamous cell carcinoma
Source: Front Mol Biosci. 2023 Aug 21;10:1232875. doi: 10.3389/fmolb.2023.1232875 (PMC10475735; doi:10.3389/fmolb.2023.1232875)
Supplement: Supplementary file 1 [file DataSheet1.zip › Supplementary Material/Supplementary Table S5.docx]

Table S5. 750 DEGs between low- and high-risk groups in the training cohort.

| ID |
| --- |
| NUTF2 |
| AREG |
| RGS20 |
| TUBB6 |
| SH2D5 |
| GJB3 |
| RHOD |
| PYGL |
| ANXA3 |
| SLC25A39 |
| DUS2 |
| C16orf74 |
| TPD52L2 |
| IL1A |
| FGFBP1 |
| LRRC42 |
| F3 |
| MRTO4 |
| CSF2 |
| UPP1 |
| NIP7 |
| FSCN1 |
| RAB38 |
| HMOX2 |
| CAV1 |
| RRAS2 |
| MYOSLID |
| FJX1 |
| ITGA3 |
| MIR193BHG |
| APLN |
| PLEK2 |
| AC002401.4 |
| SLC16A1 |
| FOSL1 |
| RNASE7 |
| PLCB3 |
| LINC00941 |
| EREG |
| PLS3 |
| LINC02178 |
| AL365356.5 |
| PXN |
| PNP |
| LDHA |
| FAM83A |
| TGFA |
| S100A10 |
| CIAPIN1 |
| CXCL5 |
| AL161431.1 |
| CARHSP1 |
| MT2A |
| EBNA1BP2 |
| LRRC59 |
| IL24 |
| MPHOSPH6 |
| CITED4 |
| CDH3 |
| GJB5 |
| WDR66 |
| FSTL3 |
| HMGA2 |
| LINC02323 |
| RPS6KA4 |
| IL1B |
| PTHLH |
| EIF3I |
| CTPS1 |
| PPBP |
| LINC00958 |
| NDFIP2 |
| PHLDA2 |
| TPBG |
| UCHL3 |
| MDFI |
| ACOT7 |
| KRT14 |
| LYAR |
| C1orf116 |
| MT1E |
| POLR2L |
| PPP4R1 |
| IFNK |
| VEGFC |
| CYP27B1 |
| ENO1 |
| KHDC1L |
| KARS |
| PAQR5 |
| EFNB1 |
| PWP1 |
| NOP14-AS1 |
| GRPEL1 |
| GNA15 |
| EIF2S1 |
| PHB |
| MT2P1 |
| LINC01322 |
| NRG1 |
| SMTN |
| AP001453.2 |
| BOP1 |
| KRT17 |
| FAM83A-AS1 |
| S100A2 |
| CCT3 |
| LPAR3 |
| SERPINB2 |
| BNIP3 |
| MYL12B |
| MTHFD1L |
| SLC7A5 |
| JPT1 |
| C19orf33 |
| TLDC1 |
| SFN |
| CHMP1A |
| TNFRSF12A |
| MAP7D1 |
| SLC22A1 |
| CDK6 |
| PLCD3 |
| ANGPTL4 |
| STK17A |
| WNT7A |
| SERINC2 |
| LINC00707 |
| GSDMA |
| MRPL15 |
| PRELP |
| ZBTB18 |
| PLA2G2D |
| PARM1 |
| METTL7A |
| IRX6 |
| PBX1 |
| SMOC2 |
| COLCA2 |
| MYLIP |
| SKAP1 |
| RAI2 |
| ELN |
| RRAGD |
| CBX7 |
| TMEM150C |
| VPREB3 |
| MGAT3 |
| HLF |
| PLPP3 |
| LRIG1 |
| FRZB |
| EYA2 |
| AC093010.3 |
| LINC00885 |
| CLIC6 |
| DYRK1B |
| ADRA2A |
| MIAT |
| RGS5 |
| REPIN1 |
| RNF150 |
| PGPEP1 |
| AQP1 |
| DPT |
| SEMA4D |
| SCUBE2 |
| ITM2A |
| POU2AF1 |
| NAPSB |
| NCOA1 |
| BMF |
| P2RY8 |
| FXYD6 |
| CHRDL1 |
| ID2 |
| CYFIP2 |
| BCL2 |
| MAP4K1 |
| MFAP4 |
| CCL19 |
| TCF4 |
| NTRK2 |
| LEF1 |
| ALDH1A1 |
| KLHL24 |
| ZDHHC2 |
| SMARCA2 |
| EPHB6 |
| RASSF2 |
| AC004656.1 |
| CD5 |
| COMP |
| PODN |
| ITPKB |
| VASH1 |
| TP53INP1 |
| PTN |
| SLC6A6 |
| FGD5 |
| LMO4 |
| MS4A1 |
| ATP6V0E2 |
| SELP |
| GPD1L |
| CD200 |
| SPIB |
| UBL3 |
| ZBTB7C |
| KLRB1 |
| ST3GAL1 |
| CYP27A1 |
| TSPAN33 |
| PPP1R16B |
| KIT |
| PI16 |
| C7 |
| ADA2 |
| APBB1IP |
| MGP |
| CELF2 |
| SPN |
| ACO2 |
| NEURL1B |
| OMD |
| CLEC3B |
| ADCY6 |
| FAM117B |
| TNS1 |
| GSTM2 |
| PRKX |
| PPM1M |
| PLEKHG1 |
| ABCA3 |
| NEFH |
| SBK1 |
| CD79B |
| NFIC |
| AMOT |
| LTBP3 |
| RAMP2 |
| ABHD4 |
| PBXIP1 |
| IKZF1 |
| CEP68 |
| CD27 |
| ETV6 |
| SDK1 |
| ADAM23 |
| CXCR4 |
| CD6 |
| SUSD4 |
| TBC1D9 |
| RARB |
| ID4 |
| FAM172A |
| TSHZ1 |
| MAN2A2 |
| ICK |
| DNMT3A |
| IGFBP5 |
| GSTM4 |
| ZNF302 |
| KLF2 |
| CCDC97 |
| NISCH |
| USP54 |
| CCR7 |
| FBLN5 |
| FAM84A |
| SPOCK2 |
| CUL9 |
| NFIX |
| ARHGEF6 |
| CACNA2D1 |
| VCAM1 |
| ZNF703 |
| EEPD1 |
| CD19 |
| POMC |
| IKZF3 |
| TNFRSF13C |
| PTGDS |
| ARHGEF26 |
| DOK1 |
| SLC37A1 |
| NOTCH3 |
| GATM |
| SYNGR1 |
| SUN2 |
| LGALS9 |
| ASB8 |
| USP19 |
| DERL3 |
| VWA5A |
| TLE2 |
| TENT5C |
| LZTS1 |
| TNS2 |
| TOP2B |
| RNF130 |
| CD37 |
| AC005332.6 |
| PGAP1 |
| TRBC2 |
| PXYLP1 |
| BEX4 |
| FAM171A1 |
| SCUBE3 |
| GYPC |
| SOX21-AS1 |
| PIK3R1 |
| TRAC |
| EIF4EBP3 |
| WIPF1 |
| TMEM178B |
| SVIP |
| AC104083.1 |
| CBFA2T2 |
| EPYC |
| CHPT1 |
| RAMP3 |
| SAMD12 |
| CLIC2 |
| LGALS2 |
| VAV1 |
| HHEX |
| GZMM |
| IGLV8-61 |
| UTRN |
| GIMAP7 |
| TRBV20-1 |
| LIMD2 |
| CD1C |
| BMP7 |
| EHD3 |
| SAP30L |
| NR1D2 |
| IQSEC1 |
| FMO3 |
| MYH11 |
| TRAK1 |
| CLIP3 |
| TMEM119 |
| SOX21 |
| MYH14 |
| ST6GAL1 |
| CD52 |
| NOTCH1 |
| ENTPD1 |
| UCP2 |
| LTB |
| APBB1 |
| IGKV2D-29 |
| LINC00963 |
| AL109918.1 |
| KIAA1147 |
| ALOX5 |
| GZMK |
| GUCY1B1 |
| CLEC10A |
| IGKV2D-24 |
| PELI2 |
| BICRAL |
| IGHV3OR16-9 |
| ATP2A3 |
| CD79A |
| FGFR2 |
| KIAA0232 |
| TBC1D10C |
| ACKR1 |
| GUCY1A1 |
| IRF8 |
| EMP2 |
| ABCA2 |
| HEY1 |
| RGL1 |
| APPL1 |
| STAG3 |
| CBX6 |
| FAM117A |
| IGHV3-64 |
| PLAC8 |
| GPRC5B |
| DIO2 |
| PLPP1 |
| XPC |
| SLAMF6 |
| SYT11 |
| CPXM2 |
| SLC9A9 |
| ZMAT3 |
| TRBV28 |
| NUP210 |
| JCHAIN |
| ASAH1 |
| FAM102A |
| MTUS1 |
| LBH |
| SOX2 |
| ARL15 |
| FZD7 |
| LINC00847 |
| CYP4X1 |
| KCNJ2 |
| SLC25A29 |
| CD48 |
| EVL |
| DNASE2 |
| RUNX1 |
| FBLN1 |
| CD3E |
| IGLV2-14 |
| PKDCC |
| CD247 |
| ZKSCAN8 |
| RTL6 |
| RGMA |
| WDR48 |
| SOSTDC1 |
| UBE2J1 |
| TBC1D17 |
| HLA-DOA |
| ARHGAP30 |
| IGHV3OR15-7 |
| JAML |
| DPYSL2 |
| IGLC3 |
| SOX4 |
| ADH7 |
| RCSD1 |
| DBP |
| TRIM2 |
| IL2RB |
| CADM1 |
| PIM2 |
| ITGAL |
| PREX1 |
| LYZ |
| SH2D3C |
| NUPR1 |
| IL34 |
| IRF4 |
| CA11 |
| ZSWIM8 |
| FOXN3 |
| ZFP62 |
| IGHV3-20 |
| IGHV3-7 |
| RAB15 |
| ZNF672 |
| ENTPD4 |
| EPS8 |
| MLLT6 |
| IL2RG |
| RERE |
| RASL11A |
| GJA4 |
| TFDP2 |
| WNK2 |
| IGHV1-2 |
| NCKAP1L |
| RNF38 |
| APOD |
| KCNE3 |
| ARHGAP31 |
| WDR82 |
| ZNF362 |
| ZBTB47 |
| BCOR |
| TBC1D5 |
| IRF2BP1 |
| GRB10 |
| CXCR3 |
| SLC35E2B |
| AL035661.1 |
| MIA3 |
| GIMAP6 |
| PER2 |
| FAM13B |
| CNOT6L |
| CALHM2 |
| IGKV3D-11 |
| IGHV1OR15-2 |
| CRTAC1 |
| BCL2L11 |
| MANSC1 |
| ARHGAP26 |
| DENND1C |
| SLIT3 |
| IGHV3-35 |
| FAM3B |
| SNTB1 |
| PTPRS |
| TXNDC15 |
| ASPN |
| RMND5B |
| ZNF24 |
| SIT1 |
| FUCA1 |
| EPC2 |
| EVI2B |
| IGLV2-28 |
| PATZ1 |
| ANXA6 |
| ADD3 |
| DOC2B |
| PTGER4 |
| SPARCL1 |
| H6PD |
| POLDIP3 |
| CXCL12 |
| MAP1B |
| ZNF740 |
| CD4 |
| GABARAP |
| SASH3 |
| SNX31 |
| IGLV1-41 |
| IGHV3-11 |
| AL022323.1 |
| RASAL3 |
| FZD10 |
| TMX2P1 |
| C3orf58 |
| CDKN1B |
| IGHV4-39 |
| IL17RB |
| PI4KA |
| LONRF1 |
| PCM1 |
| MT-ND5 |
| IGKV3-15 |
| SERTAD4 |
| TRIM24 |
| TNFSF12 |
| PTPN6 |
| PLEKHO1 |
| RSBN1L |
| IGKV2OR22-4 |
| IGHV3OR16-13 |
| MAU2 |
| ALDH3A2 |
| PEPD |
| CD34 |
| RAB42 |
| MFSD6 |
| ZBED1 |
| PIK3IP1 |
| SELENBP1 |
| DYRK2 |
| SLC44A2 |
| IGKV2-24 |
| TMEM168 |
| MIR8071-2 |
| FECH |
| AC104794.2 |
| LOXL4 |
| IGHV3OR16-8 |
| FOXP3 |
| ELK4 |
| GPC4 |
| BRD1 |
| PDIK1L |
| TACC1 |
| GPCPD1 |
| IGHV3-63 |
| POGZ |
| VGLL4 |
| TTC3 |
| NUDT16 |
| FAM53B |
| GALM |
| IGHV3-73 |
| GAS7 |
| GSN |
| CX3CL1 |
| IGHV3-38 |
| CCR5 |
| EFHD1 |
| CLEC14A |
| SVIL |
| ENPP2 |
| CYP1B1 |
| FADS2 |
| CD2 |
| KIAA1324 |
| HLA-DQA2 |
| IGHV4-59 |
| RCAN2 |
| SLC25A20 |
| FNBP1 |
| IP6K1 |
| NR1H2 |
| MADD |
| USP4 |
| IGKV3-7 |
| CLSTN3 |
| CHD6 |
| IGLV2-11 |
| CHL1 |
| TNFSF4 |
| PGAP3 |
| VSTM4 |
| PPM1B |
| IGKV1-12 |
| PIK3R3 |
| IGHJ3 |
| TCF7L2 |
| PKN1 |
| PLXNC1 |
| IGKV1D-16 |
| CABIN1 |
| IGLV1-40 |
| UBD |
| IGLV2-34 |
| PLXND1 |
| ABHD2 |
| RALGAPA2 |
| TCN2 |
| IGHM |
| EPHX1 |
| DAPL1 |
| HCLS1 |
| PKD2 |
| OR2I1P |
| PHF2 |
| ATF5 |
| COL14A1 |
| DPYSL3 |
| ZNF33A |
| PTPRC |
| SGPL1 |
| ALDH9A1 |
| MPC1 |
| HERPUD1 |
| UNC119B |
| HNRNPUL1 |
| CYTIP |
| GABRP |
| HCK |
| IGLV1-51 |
| FYCO1 |
| KCNQ1 |
| A2M |
| EFS |
| IGHV1-45 |
| PCP4L1 |
| AC103563.1 |
| MRAP2 |
| IGKV3-20 |
| LZTS2 |
| IGHV3-53 |
| IGLV2-23 |
| RASSF5 |
| DAG1 |
| MAGEH1 |
| NPPC |
| TMEM47 |
| ABI3 |
| FRG1BP |
| IGLV3-9 |
| KCTD20 |
| SEMA4A |
| LDOC1 |
| IGHV3-49 |
| IGHV3-52 |
| FUZ |
| PTPRM |
| UGT1A7 |
| PHF8 |
| HLA-DMB |
| HLA-DPB1 |
| AC136428.1 |
| EEF2K |
| DNASE1L3 |
| IL10RA |
| MAN2B1 |
| CMTM8 |
| IGLL5 |
| IGLV1-47 |
| ADAMDEC1 |
| IGLV1-50 |
| ERO1B |
| FAM83E |
| IGHV3-48 |
| PHIP |
| IGHV1-18 |
| IGKV1-39 |
| TBC1D8 |
| CSTF2T |
| CUEDC1 |
| NFE2L2 |
| HLA-DQB2 |
| IGLC2 |
| PHF10 |
| ZMIZ1 |
| SMDT1 |
| ALAD |
| PLA2G16 |
| OIP5-AS1 |
| FCER1A |
| IGHV3-19 |
| FBXW4 |
| GIMAP4 |
| BBC3 |
| IGLV5-45 |
| IGLV5-37 |
| EGR2 |
| MEGF9 |
| MAOB |
| TRBV7-9 |
| BHLHE41 |
| APLNR |
| IGKV1OR22-1 |
| RNF24 |
| RNF146 |
| IGHV3-71 |
| DGLUCY |
| AL591895.1 |
| CAMK1D |
| LCK |
| ACOX1 |
| PECAM1 |
| ZMYM2 |
| IGLV3-10 |
| SPAST |
| ZDHHC3 |
| IGKV6-21 |
| GGA2 |
| CTSO |
| LRP3 |
| IGHA1 |
| FGD5-AS1 |
| NDUFS1 |
| CPE |
| AKT3 |
| IGKC |
| FOXO1 |
| UBP1 |
| TNFRSF17 |
| TMPRSS2 |
| WDTC1 |
| IGKV3-11 |
| S100B |
| IGHV1OR15-9 |
| IGHV2-5 |
| SOS1 |
| PLSCR4 |
| TSPYL2 |
