# Supplementary material for: Effective TME-related signature to predict prognosis of patients with head and neck squamous cell carcinoma
Source: Front Mol Biosci. 2023 Aug 21;10:1232875. doi: 10.3389/fmolb.2023.1232875 (PMC10475735; doi:10.3389/fmolb.2023.1232875)
Supplement: Supplementary file 1 [file DataSheet1.zip › Supplementary Material/Supplementary Table S6.docx]

Table S6. 755 DEGs between low- and high-risk groups in the validation cohort.

| ID |
| --- |
| UPP1 |
| EIF3I |
| S100A2 |
| SLC25A39 |
| KRT17 |
| PHB |
| ATP5MPL |
| LINP1 |
| CXCL8 |
| AREG |
| MYOSLID |
| CINP |
| PPP1R14C |
| NEDD8 |
| GJB3 |
| WDR66 |
| EIF5A |
| PLEK2 |
| PYGL |
| SH2D5 |
| LINC01322 |
| NPM3 |
| SLIRP |
| FOSL1 |
| MRPS15 |
| IL1A |
| S100A10 |
| GJB5 |
| NOP10 |
| C19orf33 |
| NUTF2 |
| FGFBP1 |
| EBNA1BP2 |
| NME1 |
| RHOD |
| MRTO4 |
| FST |
| AHSA1 |
| MDFI |
| TXNDC17 |
| INF2 |
| RAN |
| RPL36AL |
| ZNF165 |
| RGS20 |
| JPT1 |
| TPD52L2 |
| PTRH2 |
| LINC00958 |
| NDUFS5 |
| TRMT61A |
| AP1M2 |
| F3 |
| COX16 |
| GSTP1 |
| RRAS2 |
| MRPL14 |
| ZNF114 |
| MFSD2A |
| CSF2 |
| ITM2A |
| CELF2 |
| P2RY8 |
| CD5 |
| IKZF1 |
| PPP1R16B |
| RASSF2 |
| ARHGEF6 |
| SLAMF6 |
| SPN |
| ELN |
| TENT5C |
| PRELP |
| DOCK2 |
| APBB1IP |
| RAI2 |
| VCAM1 |
| IGHV1-18 |
| TRBV20-1 |
| CYTIP |
| RGS5 |
| RCSD1 |
| TRAC |
| EVI2B |
| CXCR4 |
| PLPP3 |
| TRBJ2-7 |
| BCL2 |
| ADA2 |
| PARM1 |
| DOCK8 |
| ADRA2A |
| UBL3 |
| DERL3 |
| CD37 |
| CD27 |
| SYT11 |
| IGLV2-14 |
| CD96 |
| PTPRC |
| PLXNC1 |
| CD79B |
| SPOCK2 |
| ALOX5 |
| NCKAP1L |
| PPM1M |
| CD79A |
| ENPP2 |
| IGHJ3 |
| IRF4 |
| MS4A1 |
| ST6GAL1 |
| ARHGAP30 |
| AKNA |
| SKAP1 |
| AC093010.3 |
| IGHV3-49 |
| NAPSB |
| WIPF1 |
| IRF8 |
| PODN |
| POU2AF1 |
| IGHV3-64 |
| GIMAP6 |
| IL10RA |
| FAM171A1 |
| CD3G |
| IGKV3-20 |
| TAGAP |
| MFAP4 |
| CD3E |
| LZTS1 |
| IGKV3D-11 |
| IGHV1-24 |
| PTGDS |
| IGHV3-35 |
| MAP4K1 |
| ITGAL |
| BMF |
| IL2RB |
| IL21R |
| IGKV1-8 |
| IGHV3-21 |
| IGHV4-34 |
| IGHV1OR15-2 |
| SH3BP5 |
| IGHV3-74 |
| GYPC |
| CCL19 |
| TIGIT |
| EVI2A |
| PLA2G2D |
| ARHGAP31 |
| CYP27A1 |
| IGHV1-2 |
| MIR8071-2 |
| IGHV3-15 |
| SPIB |
| SASH3 |
| IGHGP |
| VASH1 |
| IGKV1-39 |
| SELL |
| ITGB2 |
| IGKV1-6 |
| IGHV4-31 |
| FOXP3 |
| IGHV4-61 |
| IGHV4-28 |
| IGHV3-33 |
| IGKV1-5 |
| IGHV3-48 |
| GZMK |
| IGHV3-19 |
| TCF4 |
| IGKV3-15 |
| FBLN5 |
| CCR7 |
| SUN2 |
| IGKV1-16 |
| IGHV3-23 |
| IGHV3OR16-13 |
| IGHV3-30 |
| CCR5 |
| IGLV1-51 |
| HLA-DOA |
| ANXA6 |
| IGHV4-55 |
| IGLV1-40 |
| SELP |
| IGHG2 |
| CD48 |
| IGKV2-30 |
| IGKV3D-15 |
| OMD |
| IGKV3-11 |
| SH2D3C |
| PREX1 |
| SIT1 |
| IGLV2-11 |
| FNBP1 |
| IGHV3-7 |
| CD6 |
| CD52 |
| DPT |
| IGLV3-21 |
| GIMAP7 |
| IGKV3D-20 |
| COMP |
| IGHV4-59 |
| CADPS2 |
| AC135068.2 |
| IGHJ2 |
| ADAMDEC1 |
| IGHV3-72 |
| IGHG1 |
| ZEB1 |
| TNFRSF17 |
| IGLV7-46 |
| CD2 |
| CD53 |
| IGHV1-69D |
| IGHV2-70 |
| AC142381.1 |
| AC134879.2 |
| BIN2 |
| TRBC2 |
| IGHV1-69 |
| TRBV28 |
| IGHV1-12 |
| CSF2RB |
| IGLL5 |
| IGKV1D-27 |
| PCED1B-AS1 |
| TNS2 |
| NFIC |
| SLA |
| PTPRJ |
| TRBV7-9 |
| IGHV3-71 |
| IGKV1D-39 |
| IGLV2-8 |
| AC034105.1 |
| IGLV3-1 |
| IKZF3 |
| CYP7B1 |
| IGKC |
| IGHV5-51 |
| IGKV1D-8 |
| GZMM |
| IGHV2-26 |
| IGKV4-1 |
| ABCA3 |
| IGHV3OR16-9 |
| IGHV3-63 |
| IGLC2 |
| PIM2 |
| RASAL3 |
| IGHV3-20 |
| IL2RG |
| CTSO |
| IGHG3 |
| IGLV7-43 |
| IGLV1-47 |
| IGKV3-7 |
| CD4 |
| SELPLG |
| CXCR3 |
| IGLV3-25 |
| AC105942.1 |
| IGLV2-23 |
| JCHAIN |
| MAP3K3 |
| IGKV1OR2-108 |
| TMEM150C |
| ENTPD1 |
| HLA-DQA1 |
| AQP1 |
| CTSS |
| IGHV1-46 |
| IGHV3-73 |
| FMOD |
| IGKV3OR2-268 |
| IGKV1-9 |
| IGKV2-24 |
| TNS1 |
| CD200 |
| IGHJ3P |
| CLEC10A |
| MEI1 |
| ATP2A3 |
| CCDC80 |
| IGKV1D-12 |
| IGHM |
| IGHV3-13 |
| IGHV3-53 |
| PRRX1 |
| GPR183 |
| SUSD3 |
| IGKV1D-42 |
| IGLV4-69 |
| IGKV1OR2-6 |
| CXCR6 |
| FERMT3 |
| AC103563.1 |
| IGLV2-18 |
| NFAM1 |
| SMARCA2 |
| IGKV2D-24 |
| FYN |
| GIMAP4 |
| TBC1D10C |
| IGLC3 |
| CLIP3 |
| AC136428.1 |
| CHIT1 |
| LCK |
| PLPP1 |
| METTL7A |
| SIRPG |
| ORAI2 |
| CST7 |
| WAS |
| IGKJ5 |
| IGLV3-12 |
| MZB1 |
| IGKV2D-28 |
| A2M |
| TNFSF12 |
| CXCL12 |
| ADCY6 |
| MGAT3 |
| SLA2 |
| FBLN1 |
| PECAM1 |
| IGHV2-70D |
| HCLS1 |
| LYZ |
| STAG3 |
| PBX1 |
| FAM13B |
| IGKV6D-21 |
| OLFML1 |
| S1PR1 |
| IGLV3-19 |
| IGKV1OR22-1 |
| IGHV3-11 |
| IGHV4-4 |
| CNOT6L |
| IGKV1D-43 |
| IGKV2D-29 |
| RGS1 |
| IGKV1-33 |
| CYBB |
| FXYD6 |
| IGHV1OR15-9 |
| IGHV4-39 |
| IGHV5-78 |
| IGLV1-44 |
| IGKV1-17 |
| IGHV6-1 |
| IGHJ1 |
| FMNL1 |
| IGHV1-67 |
| IGLV8-61 |
| FMNL3 |
| IGLV10-54 |
| IGHV3-66 |
| CLEC3B |
| SMOC2 |
| IGLV3-10 |
| ABI3 |
| ARHGAP9 |
| IQSEC1 |
| PDGFRL |
| IGLV6-57 |
| IGLV3-16 |
| NCOA1 |
| IGHV3-43 |
| HERC1 |
| EYA2 |
| LPXN |
| CD247 |
| IL34 |
| FCMR |
| FGR |
| TNFRSF1B |
| GPSM3 |
| GXYLT2 |
| IGFBP5 |
| SLC6A6 |
| MEGF6 |
| HNRNPA1P21 |
| RAB42 |
| INPP5D |
| IGKV2OR22-4 |
| IGKV1D-33 |
| IGHV3OR15-7 |
| LCP1 |
| FGL2 |
| KCNAB2 |
| PDGFRA |
| BCL2L11 |
| MGP |
| SELE |
| PTPRM |
| IGHV1-45 |
| CD8A |
| LGALS2 |
| IGHV2-5 |
| IL17REL |
| IGKV1-27 |
| LRP1 |
| AC103563.3 |
| PLEK |
| AKT3 |
| COLEC12 |
| UCP2 |
| MYO1F |
| IGKV5-2 |
| GAS7 |
| RAMP2 |
| PLEKHG1 |
| AP001972.5 |
| IGHD3-9 |
| TLR1 |
| IGHV1-3 |
| ASPN |
| HLA-DQB2 |
| IGLVI-70 |
| CSF1R |
| JAK3 |
| CBX7 |
| KIAA0355 |
| CREBL2 |
| NR1D2 |
| PTPN7 |
| CORO1A |
| LAPTM5 |
| SLC7A7 |
| IGLV1-41 |
| VAV1 |
| TRAF1 |
| SAP30L |
| LGI2 |
| HLA-DMB |
| JCAD |
| IGKV2-29 |
| TMEM119 |
| NFIX |
| PTN |
| LTBP3 |
| HLA-DPB1 |
| DPYSL2 |
| TSHZ1 |
| ADAM19 |
| BCOR |
| IGLV3-27 |
| LRRC15 |
| MEF2C |
| SAMSN1 |
| HLA-DPA1 |
| IGHG4 |
| IGLV1-50 |
| CIITA |
| CD3D |
| FZD1 |
| H6PD |
| FBLN2 |
| FAM107B |
| PAPSS2 |
| GLIPR2 |
| DIO2 |
| UBE2J1 |
| DYRK2 |
| SEMA4D |
| CTDSP2 |
| LAMA2 |
| KANK2 |
| CCDC69 |
| PDZRN3 |
| PKDCC |
| JSRP1 |
| XPC |
| LTB |
| SLCO2B1 |
| TMEM178B |
| IGKV6-21 |
| PDCD1 |
| LRIG1 |
| TLE2 |
| HERPUD1 |
| LCP2 |
| ABCA2 |
| SPI1 |
| CLEC14A |
| IGKV1D-13 |
| IRX6 |
| CPA3 |
| CD8B |
| IGLV2-34 |
| MAN2B1 |
| CCL22 |
| TP53INP1 |
| TLR5 |
| SELENBP1 |
| ADGRA2 |
| RCAN2 |
| FCER1A |
| PIK3R1 |
| LINC01315 |
| TMEM176A |
| TRPV2 |
| MPEG1 |
| IGKV2-28 |
| SPARCL1 |
| SH3BGRL |
| CXCL13 |
| IGLV4-60 |
| C16orf54 |
| CPXM2 |
| IGLV3-9 |
| IGKV2D-40 |
| IGHA1 |
| ABR |
| ANTXR1 |
| DOK1 |
| INSR |
| SIGLEC10 |
| HAVCR2 |
| ZCCHC24 |
| HLA-DRA |
| PADI2 |
| ASAH1 |
| FOXO1 |
| ZSWIM4 |
| ZBTB18 |
| IGKV1D-16 |
| IGLV9-49 |
| XYLT1 |
| MDFIC |
| ID2 |
| LNPEP |
| CEP68 |
| THBS4 |
| KAT2B |
| CD74 |
| COL8A1 |
| FMO2 |
| INSM1 |
| IGHV1-58 |
| ALOX5AP |
| TMEM47 |
| CACNA2D1 |
| RNF38 |
| FGFR1 |
| DDR2 |
| SLAMF7 |
| KCTD20 |
| ETV6 |
| TBC1D1 |
| C1QTNF3 |
| LGR5 |
| MRAS |
| PIK3IP1 |
| OR2I1P |
| TACC1 |
| FBN1 |
| MARF1 |
| CD34 |
| RASSF4 |
| ISLR |
| SLC40A1 |
| SOD3 |
| DAB2 |
| EVL |
| TRDC |
| UTRN |
| LEF1 |
| IGKV1-13 |
| TCF12 |
| SPON1 |
| FPR3 |
| RNASE6 |
| LY86 |
| TMEM140 |
| ARID5B |
| LILRB4 |
| LOXL1 |
| IGHA2 |
| FOXO4 |
| CSF1 |
| CYP1B1 |
| CD8B2 |
| MED12 |
| HEG1 |
| CDKN1B |
| RUSC2 |
| SLAMF8 |
| VSTM4 |
| PTGER4 |
| IGLV5-37 |
| NTN1 |
| SERPINF1 |
| LAIR1 |
| TNS3 |
| IGLV5-45 |
| GUCY1B1 |
| IGLV5-48 |
| IL4I1 |
| PIP4K2A |
| ACKR1 |
| MIR8071-1 |
| ARHGDIB |
| CTSK |
| JAK2 |
| AOAH |
| LMOD1 |
| IGLC6 |
| PCGF5 |
| HLA-DQA2 |
| TMC8 |
| TPSAB1 |
| IGKV1-12 |
| SLCO2A1 |
| FNDC1 |
| BHLHE41 |
| KMT2E |
| CREBBP |
| RGL1 |
| SETD1B |
| TMEM176B |
| IGHD |
| COL8A2 |
| PARP4 |
| S100B |
| GNS |
| AKAP13 |
| CLIC2 |
| ARNT2 |
| IL3RA |
| NR2F2 |
| GATM |
| ITGAM |
| VEZF1 |
| HLF |
| SSC5D |
| LPL |
| COL14A1 |
| GAL3ST4 |
| FAM198B |
| SYNPO2 |
| CALHM2 |
| EBF4 |
| ID4 |
| JAK1 |
| TXNDC15 |
| APPL1 |
| ARHGAP45 |
| PTPN18 |
| CD7 |
| ENG |
| CXXC5 |
| ITGA11 |
| RERE |
| HCK |
| KLF2 |
| CMKLR1 |
| COL15A1 |
| RUNX1 |
| TMX4 |
| GALM |
| MYLIP |
| COLCA1 |
| CIC |
| CTSW |
| NKG7 |
| ABL1 |
| FAM117A |
| LGALS9 |
| AC004656.1 |
| TBC1D5 |
| RAMP3 |
| PPFIBP2 |
| APOBR |
| MS4A6A |
| FIBIN |
| IGLV1-36 |
| LIMD2 |
| NEURL1B |
| UBD |
| CCL2 |
| CDH5 |
| TP53I11 |
| NEO1 |
| PI4KA |
| LPIN2 |
| TNFRSF19 |
| PALM |
| FMO1 |
| GTPBP1 |
| FAM3B |
| ZNF532 |
| CASC4 |
| CCR1 |
| MLLT6 |
| ARHGEF10L |
| PRR12 |
| OLFML3 |
| TMEM204 |
| ASB8 |
| MXRA5 |
| IGSF6 |
| SRGN |
| TNFSF13B |
| SFRP2 |
| LMO4 |
| IL18BP |
| HSPG2 |
| MNDA |
| PLEKHO1 |
| BTNL9 |
| HLA-DRB6 |
| SOCS5 |
| FAM19A5 |
| ARL6IP5 |
| NCF4 |
| FAS |
| LMCD1 |
| KLF9 |
| SVIL |
| TRANK1 |
| CD93 |
| MEGF8 |
| DENND1C |
| LRRC32 |
| CH25H |
| TLN1 |
| SLC44A2 |
| ZMYM3 |
| EPB41L2 |
| PLVAP |
| ANGPTL2 |
| RAPGEF1 |
| RRAGD |
| VGLL4 |
| WDTC1 |
| PKIA |
| ZBTB47 |
| ABHD2 |
| LBH |
| KDM3B |
| CYP4X1 |
| HLA-DMA |
| SEMA5A |
