# Supplementary material for: Effective TME-related signature to predict prognosis of patients with head and neck squamous cell carcinoma
Source: Front Mol Biosci. 2023 Aug 21;10:1232875. doi: 10.3389/fmolb.2023.1232875 (PMC10475735; doi:10.3389/fmolb.2023.1232875)
Supplement: Supplementary file 1 [file DataSheet1.zip › Supplementary Material/Supplementary Table S7.docx]

Table S7. 59 differentially expressed transcription factors between normal and tumor samples with FDR < 0.05 and |log_2_FC | ≥ 1.

| ID | logFC | pValue | FDR |
| --- | --- | --- | --- |
| MYBL2 | 2.420930041 | 9.34E-26 | 2.93E-23 |
| E2F1 | 2.411076886 | 1.12E-24 | 3.50E-22 |
| CENPA | 2.116534048 | 4.94E-24 | 1.54E-21 |
| CBX3 | 1.204701667 | 6.24E-24 | 1.94E-21 |
| HOXC9 | 3.914620971 | 4.19E-23 | 1.30E-20 |
| FOXM1 | 2.214295567 | 5.36E-23 | 1.65E-20 |
| SNAI2 | 1.924518772 | 5.63E-23 | 1.73E-20 |
| HOXC11 | 4.007518355 | 4.79E-21 | 1.46E-18 |
| NCAPG | 1.623038529 | 9.95E-21 | 3.01E-18 |
| E2F7 | 2.294876624 | 1.38E-20 | 4.17E-18 |
| CDK2 | 1.209585084 | 1.83E-20 | 5.51E-18 |
| TBL1XR1 | 1.153704994 | 2.30E-19 | 6.89E-17 |
| STAT2 | 1.310925875 | 5.48E-18 | 1.63E-15 |
| HOXB7 | 3.811208617 | 1.42E-17 | 4.21E-15 |
| ARID3A | 1.236064126 | 2.09E-17 | 6.13E-15 |
| LHX2 | 1.22168301 | 3.74E-17 | 1.09E-14 |
| CBX8 | 1.180921864 | 3.90E-17 | 1.13E-14 |
| DNMT1 | 1.250066033 | 4.00E-17 | 1.16E-14 |
| BRCA1 | 1.422527133 | 4.43E-17 | 1.28E-14 |
| SALL4 | 2.833367664 | 5.24E-16 | 1.48E-13 |
| LIN9 | 1.106194973 | 4.59E-15 | 1.28E-12 |
| CBX2 | 1.82132147 | 3.28E-14 | 8.97E-12 |
| STAT1 | 1.59598764 | 6.10E-14 | 1.65E-11 |
| TP63 | 1.217583253 | 2.05E-13 | 5.50E-11 |
| PML | 1.075200643 | 4.77E-13 | 1.26E-10 |
| FOXP3 | 1.812132306 | 1.07E-12 | 2.80E-10 |
| PRKDC | 1.022629343 | 1.15E-12 | 3.00E-10 |
| PAX5 | 2.43447203 | 1.43E-12 | 3.71E-10 |
| EPO | 2.321166485 | 1.90E-12 | 4.92E-10 |
| RAG1 | 2.156472815 | 2.84E-12 | 7.29E-10 |
| HIF1A | 1.03540995 | 2.96E-12 | 7.58E-10 |
| EMX1 | 3.09120881 | 4.07E-12 | 1.04E-09 |
| H2AFX | 1.046287386 | 1.33E-11 | 3.31E-09 |
| POU5F1 | 1.682374086 | 1.76E-11 | 4.37E-09 |
| STAT4 | 1.507203263 | 8.91E-11 | 2.16E-08 |
| LMNB1 | 1.125372173 | 1.54E-10 | 3.67E-08 |
| ETS1 | 1.158359142 | 2.60E-10 | 6.19E-08 |
| MEF2B | 1.286971223 | 2.88E-09 | 6.45E-07 |
| EZH2 | 1.065588078 | 1.20E-08 | 2.60E-06 |
| HOXA9 | 2.420637882 | 2.82E-08 | 5.93E-06 |
| EOMES | 1.593718915 | 1.34E-07 | 2.68E-05 |
| PDX1 | 3.815887898 | 3.46E-07 | 6.79E-05 |
| HNF4A | 2.407507653 | 1.64E-06 | 0.000304882 |
| HOXB13 | 4.826805046 | 2.49E-06 | 0.000454635 |
| HEY1 | 1.567824245 | 1.41E-05 | 0.002421171 |
| NANOG | 1.764114454 | 0.000311196 | 0.045745803 |
| KAT2B | -1.663449189 | 2.77E-21 | 8.50E-19 |
| PPARG | -1.935753037 | 2.08E-17 | 6.10E-15 |
| EHF | -1.613026274 | 4.82E-14 | 1.31E-11 |
| AR | -1.592576039 | 2.16E-13 | 5.77E-11 |
| FOS | -1.300217234 | 2.41E-12 | 6.21E-10 |
| EGR1 | -1.226051175 | 1.81E-11 | 4.47E-09 |
| PBX1 | -1.012816292 | 2.90E-10 | 6.85E-08 |
| ELF5 | -2.175375178 | 7.02E-10 | 1.63E-07 |
| MYH11 | -1.83234291 | 1.21E-08 | 2.61E-06 |
| MITF | -1.617841211 | 7.04E-08 | 1.43E-05 |
| KLF4 | -1.045394047 | 1.13E-06 | 0.000213151 |
| RXRG | -1.264942132 | 5.72E-06 | 0.001018478 |
| NR4A1 | -1.637737816 | 7.35E-06 | 0.00128691 |
