# Supplementary material for: Effective TME-related signature to predict prognosis of patients with head and neck squamous cell carcinoma
Source: Front Mol Biosci. 2023 Aug 21;10:1232875. doi: 10.3389/fmolb.2023.1232875 (PMC10475735; doi:10.3389/fmolb.2023.1232875)
Supplement: Supplementary file 1 [file DataSheet1.zip › Supplementary Material/Supplementary Table S8.docx]

Table S8. 10 transcription factors associated with the model genes.

| Transcription factors | model gene | correlation coefficient | pValue |
| --- | --- | --- | --- |
| PAX5 | WDFY4 | 0.701920208 | 1.45E-38 |
| FOXP3 | WDFY4 | 0.721288898 | 1.35E-41 |
| FOXP3 | AC023449.2 | 0.46920093 | 3.82E-15 |
| EOMES | WDFY4 | 0.495817755 | 5.59E-17 |
| EOMES | IGHV3-64 | 0.510024668 | 5.03E-18 |
| ETS1 | WDFY4 | 0.503905555 | 1.44E-17 |
| ETS1 | FCGR2A | 0.554707868 | 1.18E-21 |
| KAT2B | WDFY4 | 0.418351498 | 4.69E-12 |
