# Supplementary figures and images for: Effective TME-related signature to predict prognosis of patients with head and neck squamous cell carcinoma
Source: Front Mol Biosci. 2023 Aug 21;10:1232875. doi: 10.3389/fmolb.2023.1232875 (PMC10475735; doi:10.3389/fmolb.2023.1232875)

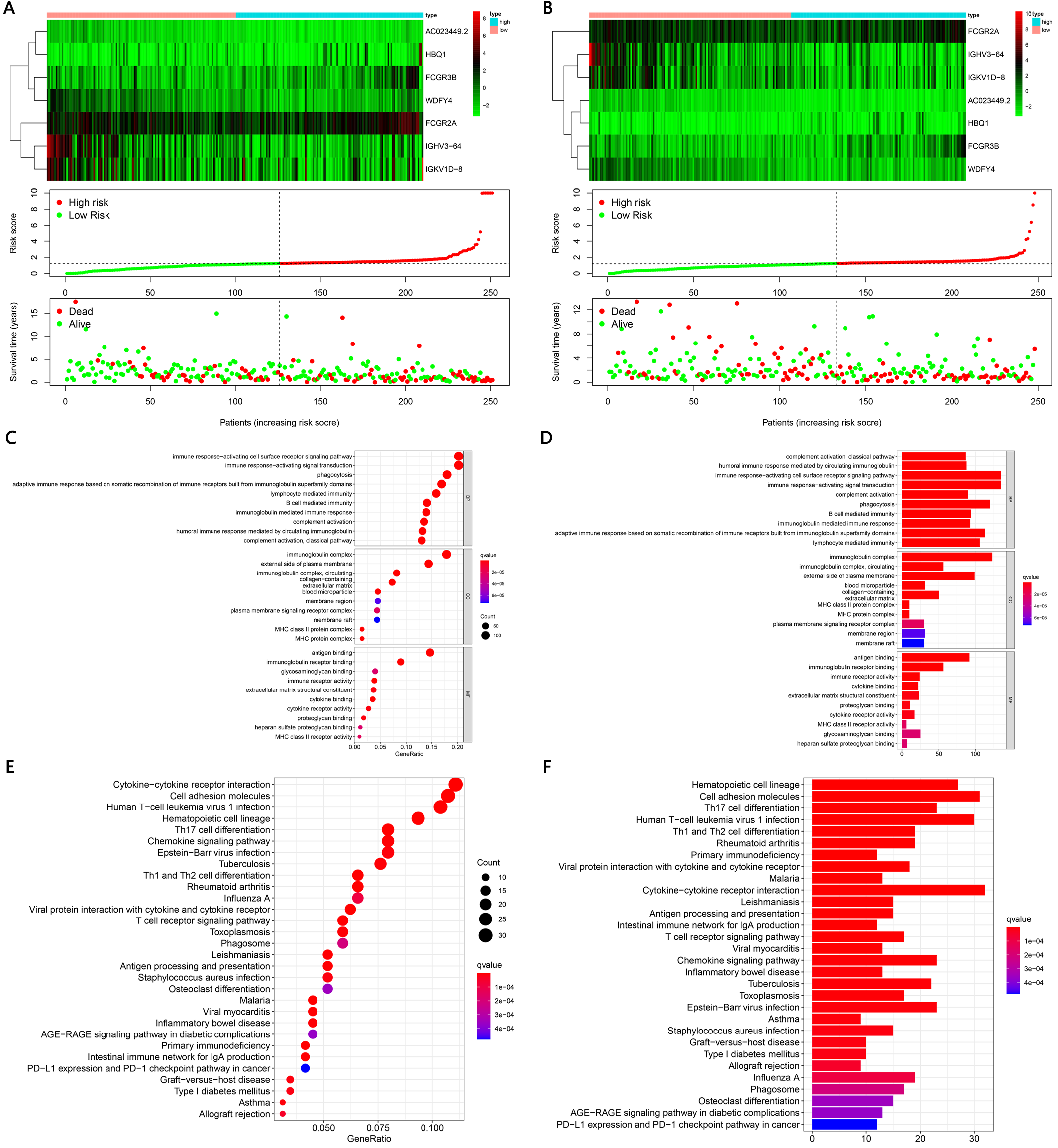

Supplement: Supplementary file 1 [file DataSheet1.zip › Supplementary Material/Supplementary Figure S1.tif]

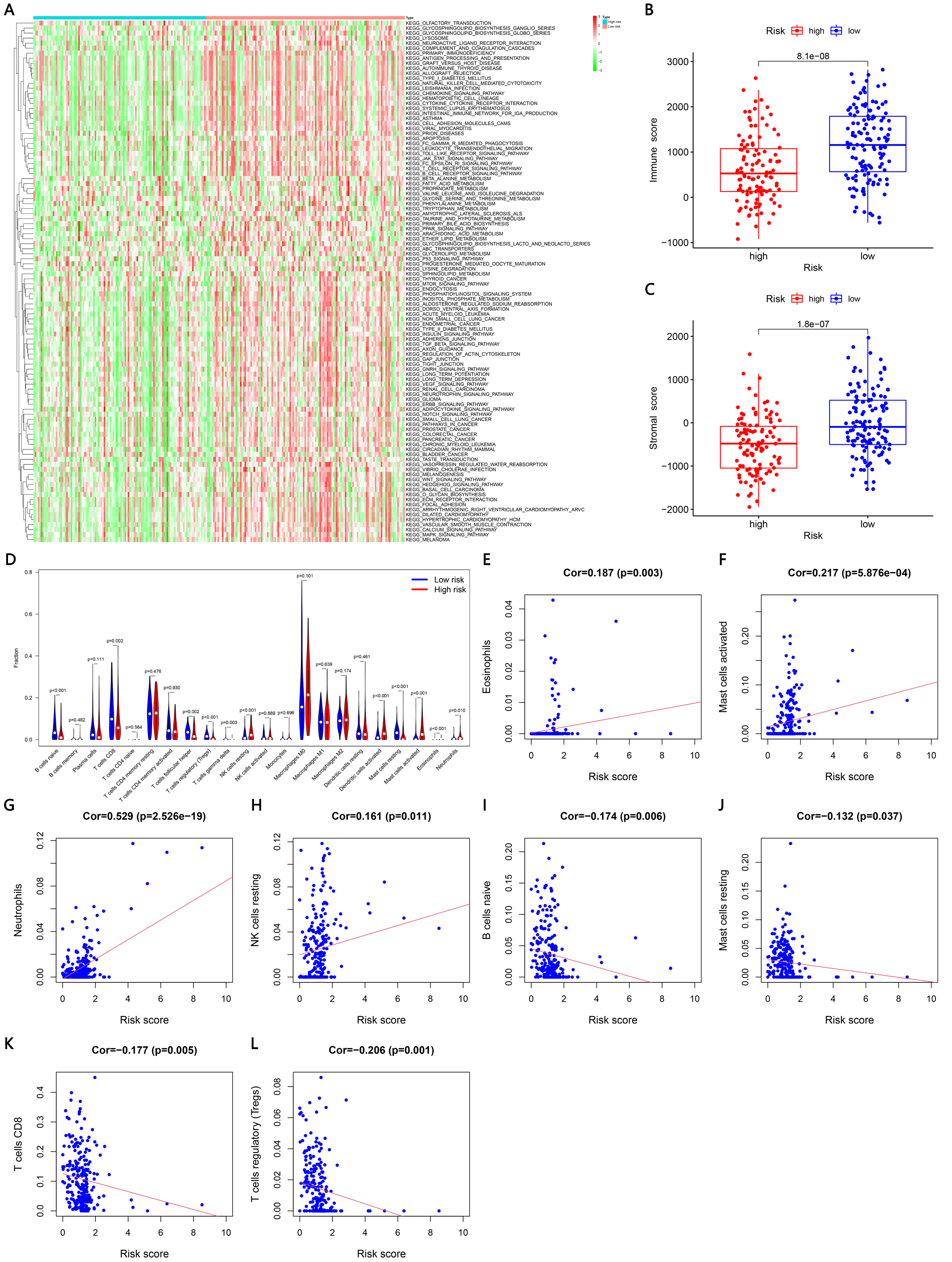

Supplement: Supplementary file 1 [file DataSheet1.zip › Supplementary Material/Supplementary Figure S2.tif]

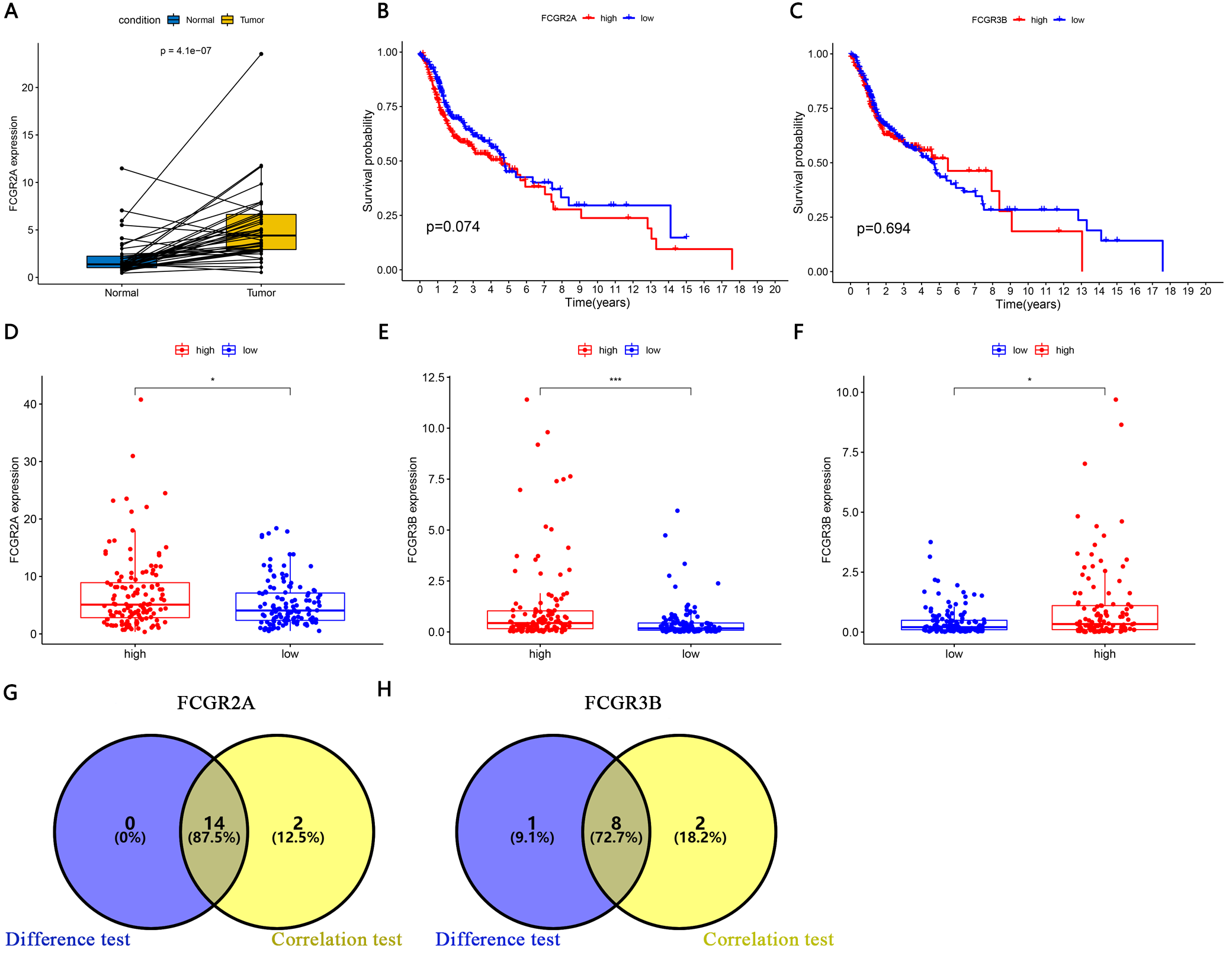

Supplement: Supplementary file 1 [file DataSheet1.zip › Supplementary Material/Supplementary Figure S3.tif]
